# Discovery of SKP2‐Recruiting PROTACs for Target Protein Degradation

**DOI:** 10.1002/advs.202515159

**Published:** 2026-02-04

**Authors:** Guanjun Dong, Aima Huang, Ziqing Zhao, Bikai Lai, Xin Pan, Huiyu Yang, Xiaohan Xu, Tianwei Wang, Fangchen Zhao, Zhimin Zhang, Yongbo Xue, Guanjun Deng, Wenbin Deng, Jianwei Chen

**Affiliations:** ^1^ School of Pharmaceutical Sciences (Shenzhen) Shenzhen Campus of Sun Yat‐Sen University Shenzhen P. R. China; ^2^ Shenzhen Key Laboratory of Neural Cell Reprogramming and Drug Research Shenzhen Campus of Sun Yat‐Sen University Shenzhen P. R. China; ^3^ School of Pharmaceutical Sciences (Shenzhen) Sun Yat‐sen University Shenzhen P. R. China; ^4^ Sun Yat‐sen University School of Medicine (Shenzhen) Shenzhen P. R. China; ^5^ College of Pharmacy Jinan University Guangzhou P. R. China

**Keywords:** BRD4, E3 ligase, PROTAC, SKP2, targeted protein degradation

## Abstract

Proteolysis targeting chimeras (PROTACs) have emerged as an intriguing therapeutic strategy for targeted protein degradation (TPD), functioning as heterobifunctional compounds that induce the redirection of E3 ligases to ubiquitinate neo‐substrates for proteasomal degradation. Despite the presence of over 600 E3 ligases, only a limited subset has been successfully harnessed for TPD. This study demonstrates that S‐phase kinase‐associated protein 2 (SKP2), the substrate receptor of the Cullin RING ligase 1 (CRL1) subfamily, can be employed for TPD using a selective, non‐covalent SKP2 recruiter, **SL1**. We designed and synthesized SKP2‐recruiting degraders by linking **SL1** to the BRD4 inhibitor **JQ1**. These compounds effectively induce BRD4 degradation in MV‐4‐11 cells, with the most potent compound **2‐1** exhibiting a half‐maximal degradation (DC_50_) of 298 nM, validating their potential as PROTACs. Mechanistic investigations show that **2‐1** promotes BRD4 ubiquitination and subsequent degradation in a proteasome‐ and neddylation‐dependent manner, which can be rescued by SKP2 knockdown and knockout. We further demonstrate that SKP2‐directed PROTACs effectively degrade Androgen receptor (AR) in 22RV1 cells. These findings emphasize that SKP2, frequently overexpressed in various tumor cells, can be successfully exploited for TPD through non‐covalent PROTACs, expanding the pool of E3 ligases available for potential therapeutic applications.

## Graphical abstract

Based on the SKP2‐targeting ligand **SL1**, we designed non‐covalent PROTACs by linking it with the BRD4 inhibitor **JQ1** and the AR antagonist **AL** through a linker. These PROTACs successfully induced the ubiquitination of BRD4 and AR, followed by proteasome‐mediated degradation.

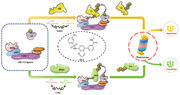

## 1 Introduction

Traditional small molecule drugs are generally thought to exert pharmacological effects by modulating protein function through binding‐mediated antagonism or agonism. However, these small molecules are less effective against proteins that lack active binding pockets or possess multiple functional domains. In recent years, the use of small molecules to direct specific proteins into the cellular proteasome system for targeted protein degradation (TPD) has emerged as a promising alternative approach [1]. The TPD strategy utilizes two types of molecules: monofunctional compounds, known as “molecular glues” and heterobifunctional compounds, referred to as proteolysis‐targeting chimeras (PROTACs). Molecular glue is a monovalent molecule that binds and reshapes the surface of E3 ligase, enabling a stabilizing and novel protein‐protein interaction with the protein of interest (POI) [2, 3, 4]. In contrast, PROTACs consist of two independent binding moieties (one targeting an E3 ligase and the other recognizing the POI) connected by a linker to form a ternary complex that brings the POI into proximity of the E3 ligase, enabling its ubiquitination and subsequent proteasome‐mediated degradation [2, 5].

Compared to traditional small molecule inhibitors, PROTACs offer significant advantages, particularly for multidomain proteins. Inhibitors typically block only specific functions and cannot disrupt scaffold roles. Whereas PROTACs induce complete protein degradation, effectively eliminating the target from the cell [6]. PROTACs can also confer ligand‐binding functionality to “silent” sites on proteins, potentially overcoming challenges associated with target proteins that lack active binding pockets and were previously deemed “undruggable” [7]. Additionally, by functioning catalytically, PROTACs may enable lower drug concentrations to achieve the desired pharmacological effect [8]. Despite the significant therapeutic potential and rapid development of PROTACs, a critical issue persists, as only a handful of the over 600 predicted E3 ligases have been confirmed to be effectively utilized by small molecule degraders, with most PROTACs designed based on ligands of the von‐Hippel Lindau (VHL) and cereblon (CRBN) [8, 9, 10]. Recent studies have demonstrated that VHL and CRBN exhibit markedly different and restrictive substrate specificities in targeted protein degradation [11, 12, 13, 14]. To fully harness the therapeutic potential of TPD, it is essential to expand the repertoire of available E3 ligases.

So far, aside from CRBN and VHL E3 ligase binders, few other non‐covalent ligase binders in PROTACs exhibit comparable efficacy in achieving low to sub‐nanomolar potency [5, 15]. Other E3 ligases, such as IAP [16], KEAP1 [17], and MDM2 [18], have been reported to have promising non‐covalent binders for TPD, but their efficacy remains relatively weak. In recent years, through sustained research efforts, several novel E3 ligases, including DCAF1 [19, 20], DCAF11 [21], DCAF15 [22], and TRIM21 [23], have been identified for TPD, concurrent with the development of non‐covalent PROTACs exhibiting nanomolar‐level potency. Additionally, various chemical proteomics approaches employing electrophilic probes have enabled the identification of additional tractable ligases for TPD, including RNF4 [24], RNF114 [25], DCAF1 [26], DCAF11 [27], DCAF16 [28], DDB1 [29], SKP1 [30], and FEM1B [31]. Notably, a structure‐based fragment‐growing strategy was employed, starting from a phosphotyrosine (pY) core fragment, to rationally design and optimize the covalent ligand **MN551** targeting the E3 ligase SOCS2, which holds promise as an E3 ligase recruiter for PROTAC‐mediated targeted protein degradation [32]. However, these covalent ligands for E3 ligases present limitations that may compromise the catalytic properties of PROTACs, as their efficacy relies on the half‐life of the E3 receptor domains. Moreover, covalent binding may interfere with the degradation of natural substrates, potentially increasing the risk of on‐target toxicity. Ultimately, it is noteworthy that most PROTACs are developed from substrate receptors associated with Cullin‐Ring Ligase (CRL) E3 complexes. Specifically, VHL and FEM1B function as substrate receptors for CRL2, KEAP1 for CRL3, and CRBN along with DCAF family proteins for CRL4.

Here, we present the discovery of non‐covalent PROTACs based on S‐phase kinase‐associated protein 2 (SKP2), a substrate receptor of CRL1. SKP2 expression is known to be upregulated in various cancers, where it ubiquitinates and promotes the degradation of multiple cell cycle regulators, including p21 [33], p27 [34], and p57 [35]. In this study, starting from a previously reported SKP2 inhibitor, we designed and synthesized non‐covalent PROTACs targeting BRD4 and AR, demonstrating the viability of SKP2 in TPD. Our research shows that the BRD4 degraders designed with SKP2 recruiter significantly enhance BRD4 degradation in various tumor cells, with this effect rescued by SKP2 knockdown and knockout. Additionally, we demonstrate that PROTACs utilizing SKP2 can effectively degrade AR in 22RV1 cells. Overall, the SKP2‐based PROTACs effectively promote the degradation of nuclear proteins (BRD4) and cytoplasmic proteins (AR).

## 2 Results and Discussion

### 2.1 Selection and Optimization of SKP2 Recruiters

We conducted a literature review of reported SKP2 ligands or inhibitors and performed a preliminary screening to identify optimal candidates. **SZL‐P1–41** [36] and **AAA‐237** [37] were deemed unsuitable as SKP2 ligands for TPD due to their inhibition of SKP1‐SKP2 interaction, which disrupts the formation of the CRL1 E3 complex and abolishes E3 enzymatic activity. **SMIP004,** identified by E. Rico‐Bautista et al., elevates the levels of p27 (a SKP2 substrate) in treated cells [38]. This compound also lacks SKP2 binding affinity data from a mechanistic standpoint; it inhibits the E3 enzymatic activity of SKP2, making it unsuitable for our research. Cyclopeptide **M1**, a SKP2‐P300 inhibitor, has a Kd value of 3.85 µM and does not affect SKP2's E3 activity [39]. It is not prioritized due to peptides` limitations in cell permeability and stability. Notably, compounds **SKPin C1** [40], **SYK‐031** [41], **14i** [42], and **22d** [43] are all SKP2‐CKS1 inhibitors that exhibit strong binding affinity to SKP2 without disrupting the assembly of the CRL1 E3 complex, making them ideal ligands candidates for our purposes.

**FIGURE 1:**
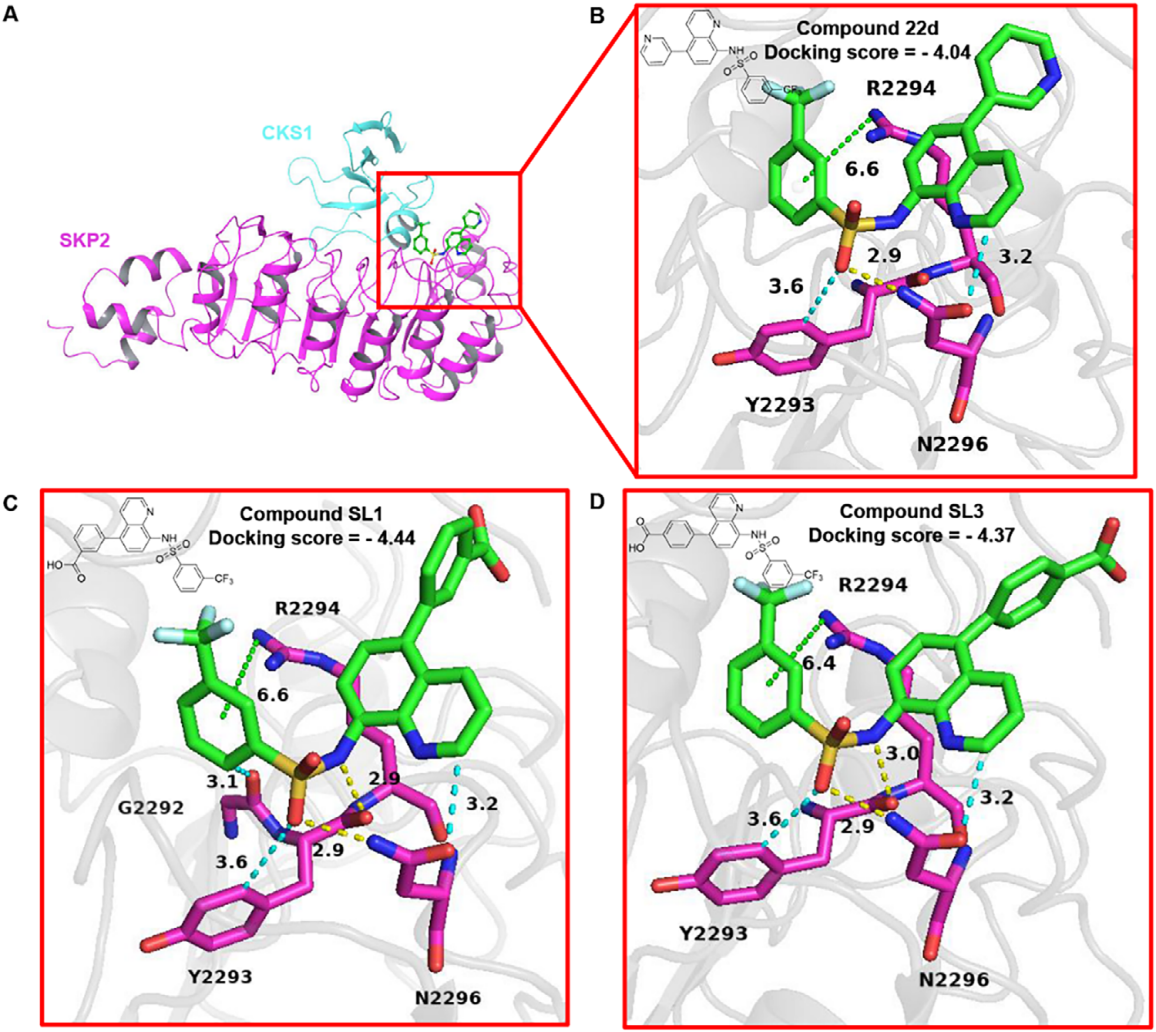
Predicted binding models of compounds **22d**, **SL1** and **SL3** with the SKP2 protein. (A) Predicted binding models of SKP2‐CKS1(2AST) with compounds **22d**. SKP2 and CKS1 are presented in cyan and purple, respectively. (B) Predicted binding models of compounds **22d**. (C) Predicted binding models of compounds **SL1**. (D) Predicted binding models of compounds **SL3**. Hydrogen bonds are represented by yellow dashed lines, arene C‐H···O hydrogen bonds by cyan dashed lines and cation‐π interactions by green dashed lines. G2292, Y2293, R2294 and N2296 are amino acids of SKP2 protein.

The reported crystal structure of the SKP1−SKP2−CKS1 complex bound to phosphorylated p27 reveals a pocket at the interface between SKP2 and CKS1, flanked by residues essential for p27 binding [44]. To identify the most suitable SKP2 recruiter, we conducted molecular docking studies to predict their binding models with SKP2, as illustrated in Figure 1 and Figure S1.

Our computational analyses demonstrate that compound **14i** forms a cation‐π interaction with R2294, a halogen bond and a hydrogen bond with E3040, resulting in a docking score of −3.57 (Figure S1B). **SYK‐031** establishes a hydrogen bond with R2294 and a cation‐π interaction with K2295, yielding a docking score of −3.84, while **SKPin C1** forms a hydrogen bond and a salt bridge with K2295, producing a docking score of −3.67 (Figure S1C,D).

In comparison, compound **22d** forms an arene C‐H···O hydrogen bond with Y2293 [45], as well as a hydrogen bond and an arene C‐H···O hydrogen bond with N2296, and a cation‐π interaction with R2294, resulting in a docking score of −4.04 (Figure 1B). The computational prediction for compound **22d** demonstrates a relatively strong binding affinity for SKP2, consistent with previously reported activity.

**FIGURE 2:**
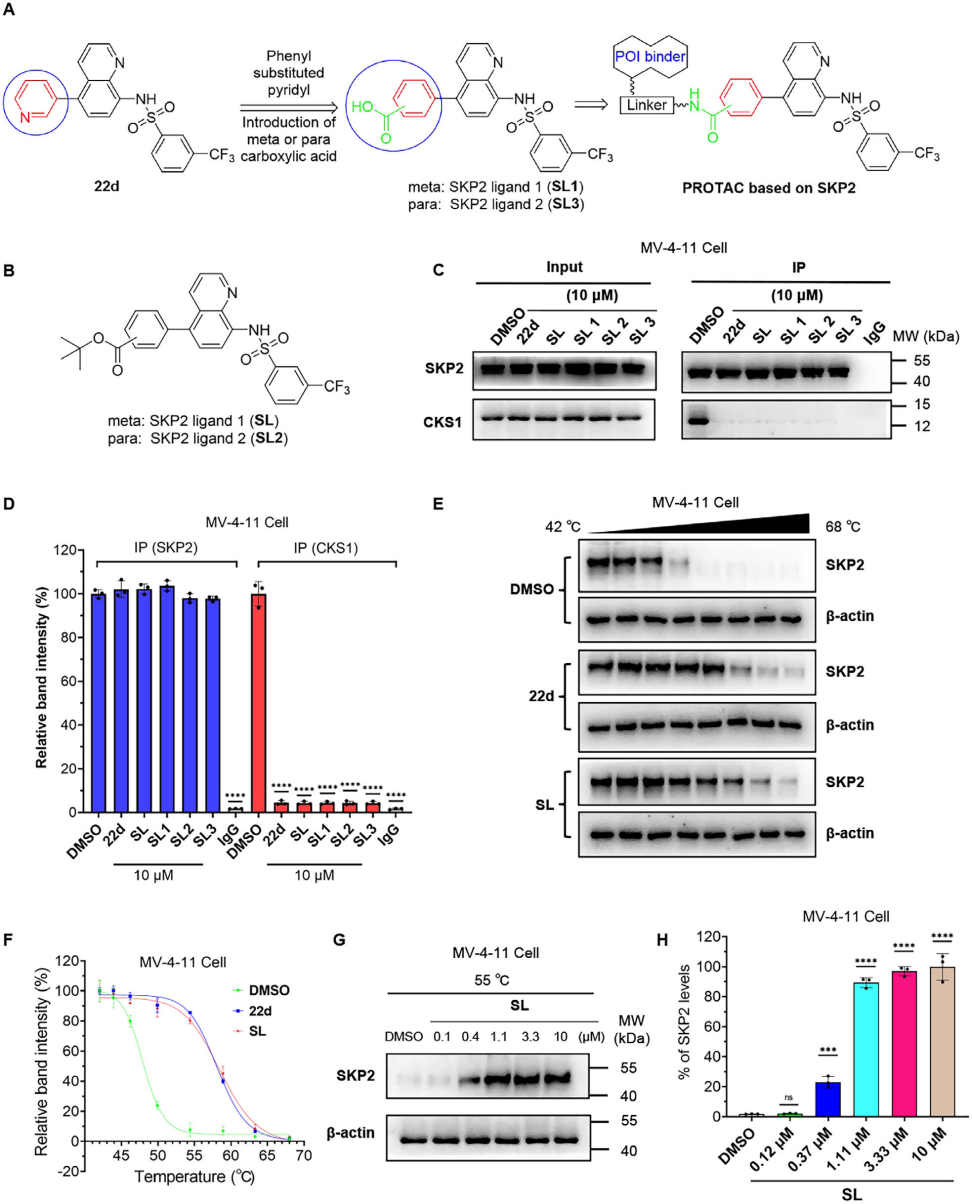
Optimization of SKP2 ligand and validation of its binding activity with SKP2 protein. (A) Design optimization of SKP2 recruiters. (B) Structures of compounds **SL1** and **SL2**. (C) Compounds **22d**, **SL**, **SL1**, **SL2** and **SL3** effectively inhibited the binding of SKP2 and CKS1 at the cellular level by binding to SKP2. After treating MV‐4‐11 cells with 10 µM of **22d**, **SL**, **SL1**, **SL2**, **SL3** or an equivalent volume of DMSO for 8 h, Western blot analysis was used to measure the protein levels of SKP2 and CKS1 pulled down by the SKP2 antibody (right panel). The baseline levels of SKP2 and CKS1 in the whole‐cell lysates were determined by Western blot (left panel). (D) Quantitative analysis of SKP2 and CKS1 abundance in the right panel of Figure C, at least three individual experiments were performed for each group. (E) MV‐4‐11 cells were treated with 10 µM of compound **22d**, **SL**, or an equivalent volume of DMSO for 1 h. The cells were then collected, heated from 42 to 68°C, lysed, and proteins were extracted for Western blot analysis to determine the SKP2 expression levels. (F) The relative bands intensities of proteins in E, at least three individual experiments were performed for each group. (G) MV‐4‐11 cells were treated with a concentration gradient of compound **SL** or an equivalent volume of DMSO for 1 h. Then the cells were collected and heated at 55°C for 10 min, lysed, and proteins were extracted for Western blot analysis to determine the SKP2 expression levels. (H) The relative bands intensities of proteins in G, at least three individual experiments were performed for each group. All data are presented as mean ± standard deviation (SD), with each group consisting of three independent experiments. *P*‐values were determined by one‐way ANOVA with Dunnett's post hoc test for multiple comparisons. ns: *p* > 0.05, ***p < 0.001, ****p < 0.0001 as compared with the DMSO controls.

Additionally, the results indicate that the pyridine ring of **22d** is solvent‐exposed and does not interact with SKP2. To facilitate the synthesis of PROTACs, we optimized the structure by replacing the pyridine ring with a phenyl ring and introducing carboxyl groups at the meta and para positions, yielding compounds **SL1** and **SL3**. (Figure 2A).

We then predicted the binding models of **SL1** and **SL3** with SKP2 by docking (Figure 1C,D). As our expectations, substituting the pyridine ring with a phenyl ring did not affect the binding mode of the compounds with SKP2. The interaction profiles remain mainly similar to those of **22d**, **SL1** and **SL3** still form an arene C‐H···O hydrogen bond with Y2293, a hydrogen bond and an arene C‐H···O hydrogen bond with N2296, and a cation‐π interaction with R2294. Both compounds form an extra hydrogen bond with Y2293, improving docking scores. Notably, **SL1** establishes an additional arene C‐H···O hydrogen bond with G2292 compared to **SL3**.

### 2.2 Compound SL Effectively Binds to the SKP2 Protein

Considering that these compounds function as inhibitors of SKP2‐CKS1 interaction, we employed co‐immunoprecipitation (CO‐IP) assays for additional validation. To mimic the SKP2 recruiter with a linker, we introduced a t‐butyl to the carboxyl group, forming esters **SL** and **SL2**, respectively (Figure 2C). The results indicate that compounds **22d**, **SL**, **SL1**, **SL2**, and **SL3** disrupt the SKP2‐CKS1 interaction, presumably by binding to the SKP2 protein intracellularly. The CO‐IP results provided preliminary evidence for the binding of these compounds to SKP2 in cells, which was consistent with our molecular docking predictions suggesting that modifications to the pyridine ring of **22d** may not critically affect SKP2 binding. Based on these combined findings, we selected the meta‐substituted small molecule **SL** and its deprotected derivative **SL1** for further investigation (Figure 2C,D).

**FIGURE 3:**
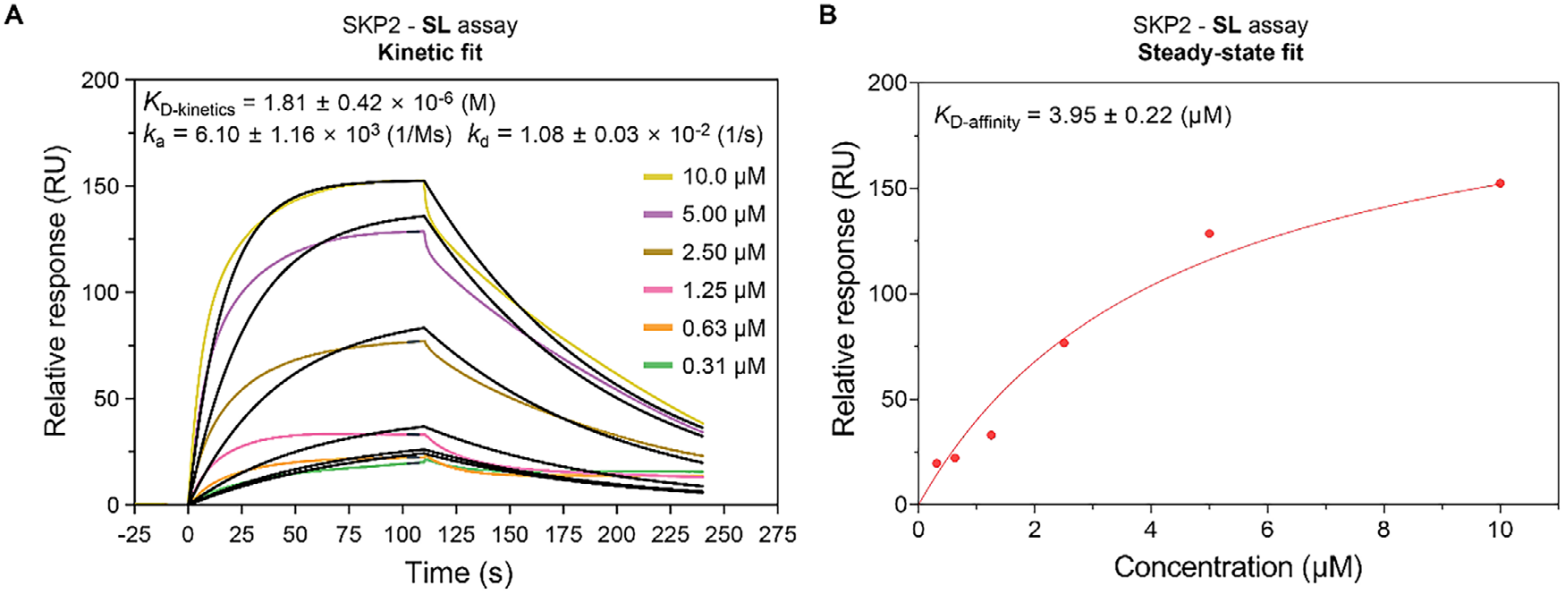
SPR graph showing the interaction of **SL** and SKP2 protein. (A) Sensorgrams and fitting curve for the binding of **SL** to SKP2. GST‐SKP2 protein was immobilized on a CM5 sensor chip, and then SPR analysis was performed on a Biacore 1K system (cytiva) instrument. The figure includes both experimental data (colored lines) and fitted curves (black lines). (B) Steady‐state plots for the binding of **SL** to SKP2. The affinity (*K*_D_) of SKP2 binding to **SL** obtained based on the “Kinetic” and “Steady‐state” methods were shown in Figure 3A,B above. The association rate (*k*_a_) and dissociation rate (*k*_d_) of the binding between SKP2 and **SL** were presented in Figure 3A. All data are expressed as mean ± SD, with each dataset consisting of three independent experiments. The three repeated SPR experiments can be viewed in the Figure S3.

Next, we further validated the binding of compound **22d** and **SL** to intracellular SKP2 protein. The cellular thermal shift assay (CETSA) demonstrated that both compounds **22d** and **SL** significantly enhanced the thermal stability of intracellular SKP2 protein in MV‐4‐11 cells compared to the DMSO control, providing further evidence for their SKP2 binding and similar activity (Figure 2E,F). To further investigate, we evaluated SKP2 stability in MV‐4‐11 cells heated to 55°C after treatment with a concentration gradient of compound **SL**. The thermal stabilization of SKP2 diminished significantly at concentrations below 1.1 µM (Figure 2G,H), implying a binding affinity near this value, which was closely corroborated by our subsequent surface plasmon resonance (SPR) data (Figure 3A,B).

To further confirm the binding interaction between **SL** and SKP2, we purified GST‐tagged SKP2 protein in vitro (Figure S2A,B). It is noteworthy that, based on prior literature and our preliminary expression studies, co‐expression of the SKP1 protein was confirmed to significantly enhance the stability and yield of SKP2, and was therefore incorporated into our expression construct [46]. SPR measurements revealed the binding affinity between SKP2 and **SL** (Figure 3A,B). The dissociation constant (*K*_D_) derived from kinetic analysis (*K*_D‐kinetic_) was 1.81 ± 0.42 µM, while that obtained from steady‐state equilibrium analysis (*K*_D‐affinity_) was 3.95 ± 0.22 µM. The binding kinetics of the SKP2‐**SL** interaction, as shown in Figure 3A, revealed a slow association rate (*k*_a_) and a moderate dissociation rate (*k*_d_). Thus, the SPR results directly quantify the affinity of the **SL**‐SKP2 interaction.

Based on these experimental results, we confirmed the binding of compound **SL** to the SKP2 protein. Consequently, the deprotected derivative **SL1** was selected as the SKP2 recruiter. The solvent‐exposed benzene ring of **SL1** presents an optimal attachment point for linking various target protein ligands via a linker, which will facilitate subsequent validation studies.

### 2.3 SKP2‐Recruiting BRD4‐Targeting PROTACs Show Potent Cell Growth Inhibition and BRD4 Degradation in MV‐4‐11 Cells

We designed and synthesized compounds **2‐1** to **2‐13** by conjugating **SL1** with the BRD4 inhibitor **JQ1** through various linkers, as summarized in Table 1. For our study, we selected MV‐4‐11 cells, which are sensitive to BRD4 and are widely used in investigating the degradation of BRD4 by PROTACs [47, 48]. Initially, we performed a bioinformatics analysis of BRD4 and SKP2 expression levels in MV‐4‐11 cells using the DepMap database (https://depmap.org/). The results indicate that both BRD4 and SKP2 proteins are expressed at moderately elevated levels in MV‐4‐11 cells (Figure S4A), making them suitable for our study. Furthermore, bioinformatics analysis confirmed a negative correlation between BRD4 knockout and MV‐4‐11 cell growth (Figure S4B).

**TABLE 1:** Structure and activity of compounds **2‐1** to **2‐13** in MV‐4‐11 cells.

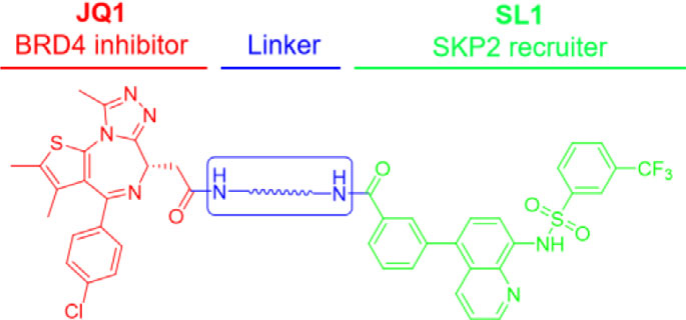				
Cpd.	Linker	IC_50_ (nM)^a^	D_max_ of BRD4 (%)^b^	
		MV‐4‐11 Cell	10 µM	1 µM
**2‐1**	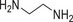	250 ± 9.8	99.6	94.1
**2‐2**	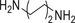	616 ± 37.2	96.9	74.1
**2‐3**	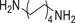	1594 ± 125	84.1	58.2
**2‐4**	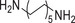	1693 ± 59	70.2	62
**2‐5**	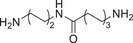	377 ± 18.5	78.5	57.4
**2‐6**	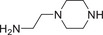	314 ± 17.1	95.6	89.7
**2‐7**	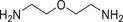	975 ± 42.6	99	82.8
**2‐8**	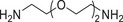	815 ± 51.5	95.4	81.7
**2‐9**	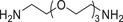	599 ± 36.8	96	60.3
**2‐10**	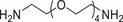	517 ± 44.4	96.1	65.4
**2‐11**	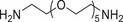	878 ± 25.1	92	83
**2‐12**	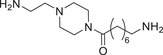	855 ± 21	68	60.5
**2‐13**	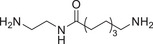	393 ± 19.1	95.3	62.5
**SL1**	—	6181 ± 181	—	—
**JQ1**	—	103 ± 9.4	—	—

^a^ The half maximal inhibitory concentration of compounds on the growth of MV‐4‐11 cells.

^b^ Maximum degradation rate of BRD4 in MV‐4‐11 cells by compounds.

Subsequently, we evaluated the antiproliferative effects of compounds **2‐1** to **2‐13** on MV‐4‐11 cells (Table 1). These compounds exhibited a significant cell growth inhibition, with compound **2‐1** showing the most potent activity with IC_50_ value of 250 nM, followed by compound **2‐6**, which also exhibited notable efficacy with a shorter linker. These results suggest that linker modifications significantly influence the activity of the compounds.

**FIGURE 4:**
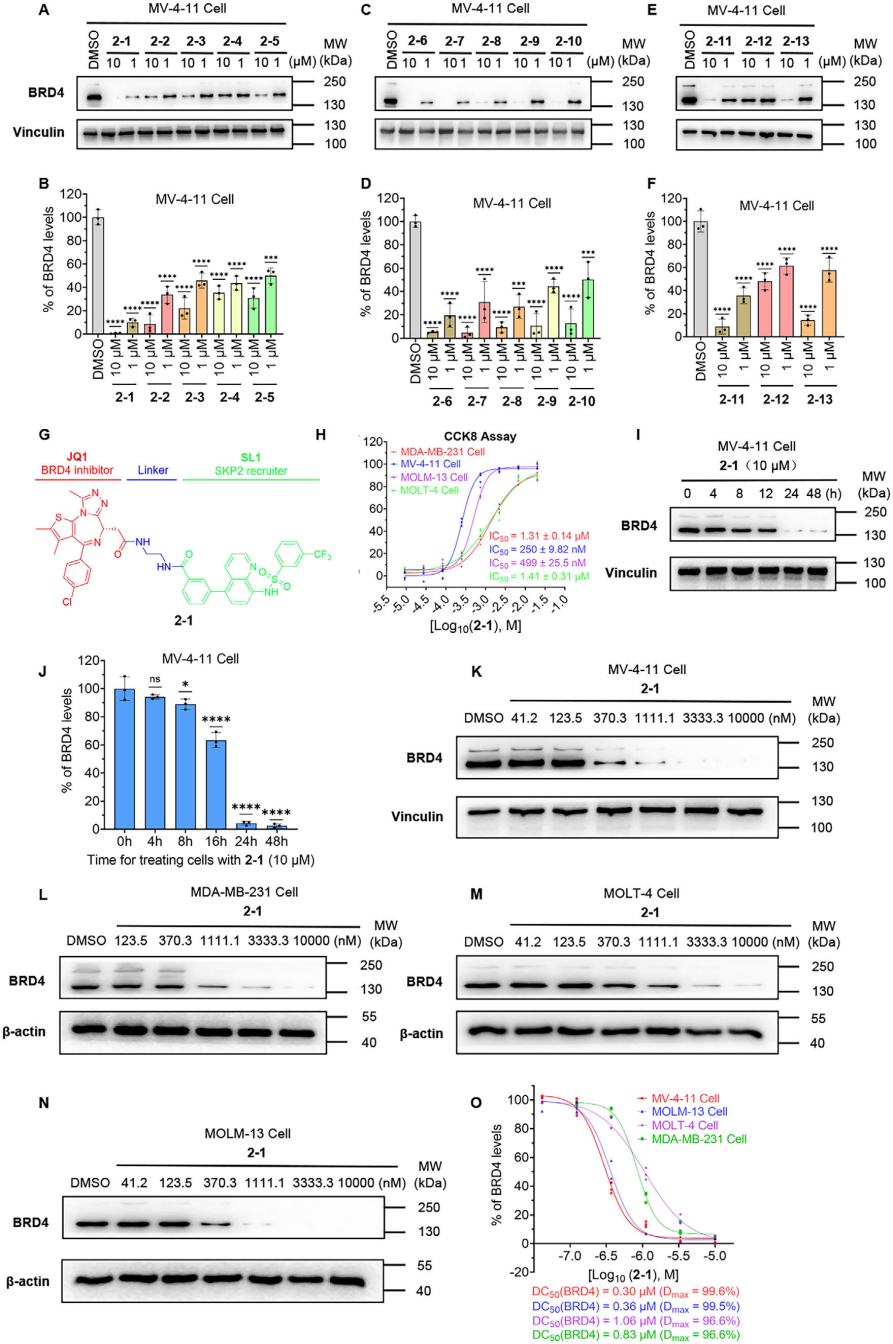
SKP2‐based PROTACs promote the degradation of BRD4. (A) After treating MV‐4‐11 cells with DMSO and **2‐1** to **2‐5** (10 µM and 1 µM) for 24 h, the levels of BRD4 and the loading control Vinculin were assessed by Western blotting, and the quantification (B) of BRD4 abundance in the indicated experimental groups was shown. (C) Western blots showing effects of DMSO and **2‐6** to **2‐10** (10 µM and 1 µM), and the quantification (D) of BRD4 abundance in the indicated experimental groups. (E) Western blots showing effects of DMSO and **2‐11** to **2‐13** (10 µM and 1 µM) and the quantification (F) of BRD4 abundance in the indicated experimental groups. (G) Structure of SKP2‐recruiting non‐covalent PROTAC **2‐1**. (H) After treating MV‐4‐11, MOLT‐4, MOLM‐13, and MDA‐MB‐231 cells with **2‐1** for 72 h, the cell proliferation inhibition was assessed using the CCK8 assay, with the data representing the mean ± SD from three independent experiments. (I) The levels of BRD4 and Vinculin detected by Western blotting after treating MV‐4‐11 cells with 10 µM of **2‐1** for different durations, along with (J) the corresponding quantification results. (K) Immunoblot analysis results after treating MV‐4‐11 cells with DMSO and varying doses of **2‐1** for 24 h, along with (O) quantification of BRD4 abundance, half‐maximal degradation concentration (DC_50_) values, and observed maximum degradation (D_max_). (L) Immunoblot analysis results after treating MDA‐MB‐231 cells with DMSO and varying doses of **2‐1** for 24 h, along with (O) quantification of BRD4 abundance, DC_50_ values, and D_max_. (M) Immunoblot analysis results after treating MOLT‐4 cells with DMSO and varying doses of **2‐1** for 24 h, along with (O) quantification of BRD4 abundance. (N) Immunoblot analysis results after treating MOLM‐13 cells with DMSO and varying doses of **2‐1** for 24 h, along with (O) quantification of BRD4 abundance, DC_50_ values, and D_max_. All data are expressed as mean ± SD, based on three independent experiments for each group. *P*‐values were determined by one‐way ANOVA with Dunnett's post hoc test for multiple comparisons. ns: *p* > 0.05, *p < 0.05, **p < 0.01, ***p < 0.001, ****p < 0.0001 as compared with the DMSO controls.

To evaluate the compounds’ ability to induce BRD4 protein degradation, we treated MV‐4‐11 cells with these compounds for 24 h. The results indicated that all compounds promoted BRD4 degradation to varying degrees (Table 1 and Figure 4). Importantly, we observed a potential correlation between degradation efficacy and antiproliferative potency. For instance, compounds **2‐1** and **2‐6**, which exhibited strong cellular activity, also demonstrated significant degradation capacity; whereas compound **2‐4**, with poorer cell viability, showed limited BRD4 degradation ability. Quantitative analysis revealed that compound **2‐1** achieved the strongest degradation effect among all tested compounds, with maximum degradation (D_max_) reaching 99.6% and 94.1% at 10 µM and 1 µM concentrations, respectively (Figure 4B,D,F). Meanwhile, compound **2‐6** also demonstrated promising degradation activity, leading us to conduct detailed degradation studies on both compounds in subsequent experiments.

Herein, we observed that the overall anti‐proliferative effect of BRD4 degraders designed based on SKP2 was inferior to that of **JQ1**. The efficacy of PROTACs depends on the efficient formation of the ternary “target protein (POI)‐PROTAC‐E3 ligase” complex and its recognition by the ubiquitin‐proteasome system. This process is influenced by multiple key parameters and cannot be solely attributed to the additive activity of the individual ligands. First, PROTAC molecules generally possess higher molecular weight and greater polar surface area, which can lead to poorer cellular permeability compared to smaller, more hydrophobic ligands. As a result, even at equivalent extracellular concentrations, the intracellular concentration of active PROTAC may be lower, leading to reduced bioactivity compared to the individual ligands. Moreover, the core function of a PROTAC is to induce target protein degradation; suboptimal degradation efficiency directly translates to diminished anti‐proliferative activity. As shown in Table 1, compound **2‐1**, which induced more efficient target protein degradation (the D_max_ of 1 µM is 94.1%), exhibited relatively stronger suppression of cell proliferation (IC_50_ = 250 ± 9.8 nM), whereas compound **2‐4**, with weaker degradation activity (the D_max_ of 1 µM is 62%), showed reduced anti‐proliferative potency (IC_50_ = 1693 ± 59 nM). This indicates that the anti‐tumor activity of these PROTACs is not solely determined by the inhibitory potency of their individual ligand moieties, but is closely correlated with their efficiency in degrading BRD4. Overall, the anti‐proliferative activity of PROTACs can be influenced by complex factors, including cellular uptake and degradation efficiency, which together explain the observed inferior performance of SKP2‐based BRD4 degraders compared to the standalone BRD4 inhibitor **JQ1**.

### 2.4 Compound 2‐1 Induces BRD4 Degradation in Various Tumor Cell Lines

After confirming that SKP2‐recruiting PROTACs induced BRD4 degradation in MV‐4‐11 cells, we further examined the time‐course degradation profile of compound **2‐1**. It induced near‐complete BRD4 degradation in MV‐4‐11 cells after 24 h of treatment (Figure 4I,J). Building on previous findings that both **2‐1** and **2‐6** exhibit promising degradation activity, we compared their degradation efficacy in MV‐4‐11 cells. Dose‐response studies revealed that compound **2‐1** exhibited a half‐maximal degradation concentration (DC_50_) of 298 nM with a maximal degradation effect (D_max_) of 99.6% (Figure 4K,O). while, compound **2‐6** showed a DC_50_ of 664 nM and a D_max_ of 98.5% (Figure S5C,D). We also evaluated the BRD4 degradation activity of **dBET1** (CRBN‐based) and **MZ1** (VHL‐based) in MV‐4‐11 cells [49, 50]. Dose‐response studies determined that **dBET1** and **MZ1** achieved maximal degradation (D_max_) of 99.3% and 99.5%, with DC_50_ values of 50.56 nM and 46.92 nM, respectively (Figure S6C,G). The results indicate that the degradation activity of both **dBET1** and **MZ1** was approximately six‐fold higher than that of compound **2‐1**. This difference may be attributed to the moderate affinity between SKP2 and its ligand. Therefore, future optimization of SKP2 ligands represents a key research direction for enhancing the degradation efficacy of SKP2‐recruiting PROTACs, suggesting considerable potential for further improvement in degradation activity. Based on its relatively superior degradation profile, compound **2‐1** was selected for further mechanistic studies.

We then evaluated the antiproliferative and degradation activities of the compound **2‐1** across various BRD4‐related tumor cell lines, including the leukemia cell lines MOLM‐13 and MOLT‐4, and the breast cancer cell line MDA‐MB‐231. Compound **2‐1** exhibited variable antiproliferative activity across these cell lines, with MOLM‐13 cells showing the highest sensitivity (IC_50_ = 499 nM) (Figure 4H). Further assessment of BRD4 degradation revealed that compound **2‐1** achieved potent degradation in MOLM‐13 cells (DC_50_ = 360 nM, D_max_ = 99.5%) (Figure 4N,O). While BRD4 degradation was also observed in MOLT‐4 and MDA‐MB‐231 cells, the activity was more moderate, with DC_50_ values of 1.06 µM and 0.83 µM, respectively (Figure 4L,M,O).

These findings indicate that compound 2‐1 can induce BRD4 degradation across various tumor cells, demonstrating its broad applicability. The varying degradation efficacies suggest that cell type‐specific factors may influence its mechanism of action.

### 2.5 Compound 2‐1 Promotes the Degradation of BET Family Proteins

While **JQ1** is known as a non‐selective inhibitor of the bromodomain and extra‐terminal (BET) family proteins by binding to the shared N‐terminal bromodomains of BRD2, BRD3, BRD4, and BRDT [51], we examined the degradation profile of compound **2‐1** across BET family proteins.

**FIGURE 5:**
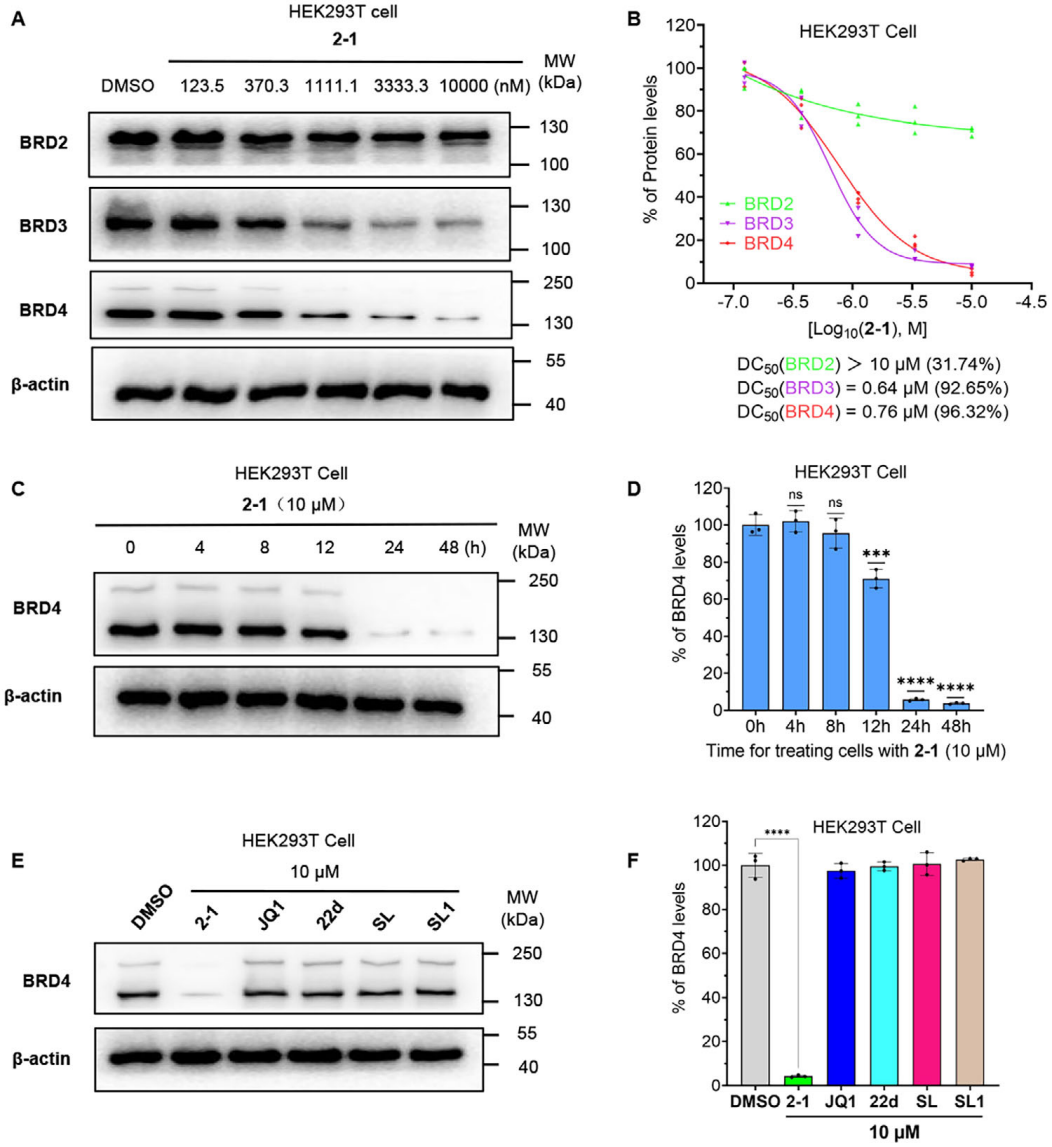
Compound **2‐1** promotes the degradation of BET family proteins. (A) After treating HEK293T cells with DMSO and various concentrations of **2‐1** for 24 h, the levels of BRD2, BRD3, BRD4 and the loading control β‐actin were assessed via Western blotting, with (B) the quantification of BRD4 abundance, DC_50_ values, and D_max_ in the experimental group are shown, data are derived from three independent experiments. (C) The levels of BRD4 and β‐actin detected by Western blotting after treating HEK293T cells with 10 µM of **2‐1** for different durations, along with (D) the corresponding quantification results. (E) After treating HEK293T cells with 10 µM of compound **2‐1**, **JQ1**, **22d**, **SL**, **SL1**, or an equal volume of DMSO for 24 h, the levels of BRD4 and β‐actin were detected by Western blot, along with (F) the corresponding quantification results. All data are expressed as mean ± SD, based on three independent experiments for each group. *P*‐values were determined by one‐way ANOVA with Dunnett's post hoc test for multiple comparisons. ns: *p* > 0.05, ***p < 0.001, ****p < 0.0001 as compared with the DMSO controls.

Initially, we observed that compound **2‐1** also promotes BRD3, BRD4 degradation in HEK293T cells, with a DC_50_ of 0.64 µM for BRD3 and a DC_50_ of 0.76 µM (D_max_ of 96.32%) for BRD4, respectively, while showing minimal effects on BRD2(Figure 5A,B). This degradation profile is somewhat distinct from those of the previously developed CRBN‐based degrader **dBET1** and the VHL‐based degrader **MZ1**, both of which promote the degradation of BRD2, BRD3, and BRD4 with comparable potency [49, 50] (Figure S6E,I). Given the absence of BRDT, another BET family member, in HEK293T cells, we evaluated the selectivity of compound **2‐1** in NCI‐H2009 cells, which endogenously express high levels of BRDT [52]. Consistent with our previous findings, **2‐1** significantly promoted the degradation of BRD3 and BRD4, but had a weaker effect on BRD2 degradation. Notably, it also promoted the degradation of BRDT (Figure S7A,B).

Subsequently, we also investigated the relationship between the treatment time of compound **2‐1** and BRD4 degradation. As shown in Figure 5C,D, minimal BRD4 degradation was observed after 8 and 12 h of treatment, while a significant reduction in BRD4 levels was evident after 24 h, demonstrating the time‐dependent nature of the degradation effect induced by **2‐1**.

To investigate whether small molecule inhibitors targeting BRD4 or SKP2 alone would promote BRD4 degradation, we first compared the effects of compound **2‐1** with those of individual BRD4 or SKP2 inhibitors **22d**, **SL** and **SL1**, on BRD4 protein levels. The results showed that, compared to compound **2‐1**, small molecules such as **JQ1**, **22d**, **SL** and **SL1** had no effect on the intracellular BRD4 protein levels (Figure 5E,F).

### 2.6 Mechanism of BRD4 Degradation by 2‐1 via Ubiquitination, Proteasome‐ and SKP2‐Dependent Pathway in HEK293T Cells

**FIGURE 6:**
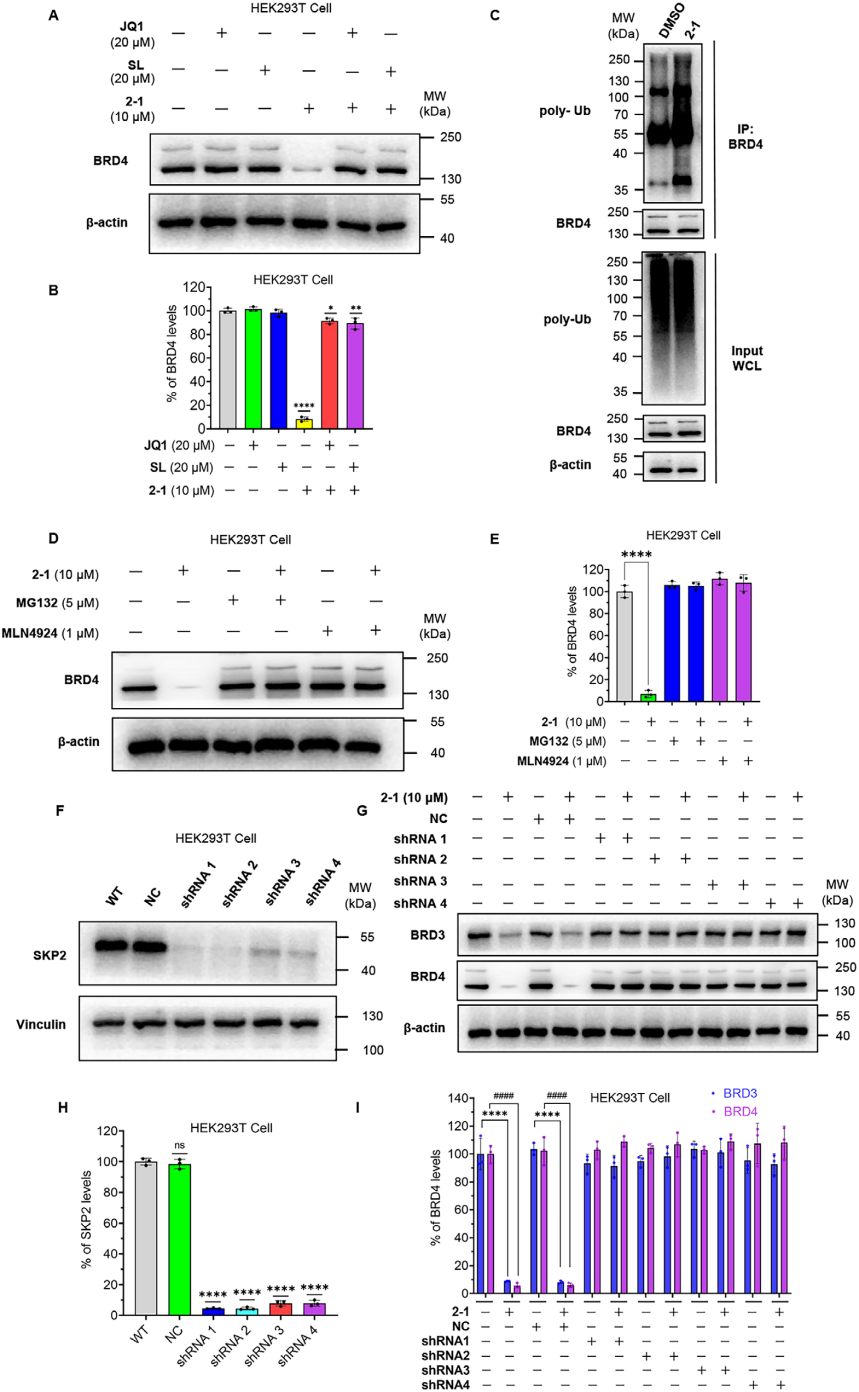
Compound **2‐1** induces ubiquitination and degradation of BRD4 in HEK293T cells. (A) Western blot analysis of BRD4 and β‐actin (loading control) in HEK293T cells pretreated with 20 µM **JQ1** or **SL** for 8 h and then exposed to 10 µM **2‐1** or DMSO for 24 h. (B) Quantitative data corresponding to the blot shown in (A). All data are expressed as mean ± SD, based on three independent experiments for each group. *P*‐values were determined by one‐way ANOVA with Dunnett's post hoc test for multiple comparisons. *p < 0.05, **p < 0.01, ****p < 0.0001 as compared with the DMSO controls. (C) **2‐1** induces polyubiquitination of BRD4 in HEK293T cells. After treating HEK293T cells with DMSO or 10 µM of **2‐1** for 18 h, 10 µM of the proteasome inhibitor **MG132** was added, and the cells were further treated for 6 h. BRD4 protein was then obtained through immunoprecipitation and analyzed by Western blotting to detect ubiquitination, with results shown in the figure. IP, immunoprecipitation; WCL, whole‐cell lysate. (D) The degradation of BRD4 mediated by **2‐1** is blocked by the proteasome inhibitor **MG132** and the neddylation inhibitor **MLN4924**. HEK293T cells were preincubated with 5 µM **MG132** or 1 µM MLN4924 for 6 h, followed by treatment with 10 µM **2‐1** along with 5 µM **MG132** or 1 µM **MLN4924** for 24 h, after which the levels of BRD4 and the loading control β‐actin were assessed by Western blotting, with (E) the corresponding quantification results shown in the figure. All data are expressed as mean ± SD, based on three independent experiments for each group. *P*‐values were determined by one‐way ANOVA with Dunnett's post hoc test for multiple comparisons. ****p < 0.0001 as compared with the DMSO controls. (F) HEK293T cells were infected with a lentiviral vector containing short hairpin RNA (shRNA) targeting SKP2 and selected using puromycin to obtain stable SKP2 knockdown cell lines. The efficacy of the knockdown was assessed by Western blotting, and (H) the corresponding quantification results are shown in the figure; the data are expressed as mean ± SD, based on three independent experiments for each group. *P*‐values were determined by one‐way ANOVA with Dunnett's post hoc test for multiple comparisons. ns: p > 0.05, ****p < 0.0001 as compared with the WT controls. (G) Wild‐type, negative control, and SKP2 stable knockdown cells were treated with DMSO or **2‐1** (10 µM) for 24 h, after which the levels of BRD3, BRD4, and the loading control β‐actin were assessed by Western blotting, with (I) the corresponding quantification results shown in the figure. WT: Wild‐type Cell, NC: Negative control cells, cells transfected with lentivirus packaged with blank plasmid. The data are reported as mean ± SD, reflecting three independent experiments conducted for each group. P‐values were determined by one‐way ANOVA with Dunnett's post hoc test for multiple comparisons. In the quantification results of BRD3, ****p < 0.0001 as compared with the DMSO controls. In the quantification results of BRD4, ^####^ *p* < 0.0001 as compared with the DMSO controls.

To assess the dual dependency of **2‐1**, HEK293T cells were pre‐treated with excess **SL** or **JQ1** prior to exposure to **2‐1**. This competition nearly abolished BRD4 degradation (Figure 6A,B), confirming that **2‐1**’s activity requires the engagement of both target protein binding sites.

We then investigated whether compound **2‐1** induced BRD4 ubiquitination and mediated degradation through the proteasomal pathway. Treatment with 10 µM of **2‐1** significantly enhanced BRD4 ubiquitination compared to the control group (Figure 6C). Moreover, co‐treatment with the proteasome inhibitor **MG132** prevented **2‐1** inducing BRD4 degradation, further confirming that the degradation process occurs through the ubiquitin‐proteasome pathway (Figure 6D,E).

Given that SKP2 functions as a component of the CRL1 E3 ligase complex, we examined the role of the Cullin‐RING ligase (CRL) system in **2‐1**‐mediated degradation. Neddylation, a post‐translational modification process, regulates protein function through the covalent attachment of the NEDD8 protein to target proteins via a cascade involving NEDD8‐activating enzyme (NAE), NEDD8‐conjugating enzyme (E2), and E3 ligase [53].

The Cullin family proteins (CUL1‐3, 4A, 4B, 5, 7, and 9) are primary physiological substrates of neddylation, and their neddylation activates CRL E3 ubiquitin ligases [54]. Pretreatment with **MLN4924**, an NAE inhibitor, significantly attenuated **2‐1**‐induced BRD4 degradation, providing evidence that compound **2‐1** operates through the CRL E3 ligase pathway (Figure 6D,E).

To further elucidate the role of SKP2 induced BRD4 degradation mediates by compound **2‐1**, we generated SKP2 knockdown HEK293T cells using short hairpin RNA (shRNA) (Figure 6F,H). As illustrated in Figure 6G, compound **2‐1** markedly induced BRD3 and BRD4 degradation in both wild‐type and negative control cells; however, this effect was significantly attenuated in SKP2 knockdown cells, confirming that the degradation mechanism is contingent upon SKP2. In addition, we generated SKP2 knockout 293T cells using the CRISPR‐Cas9 technology and treated these SKP2‐deficient cells with compound **2‐1**(Figure S8A–D). The results were consistent with previous studies, demonstrating that SKP2 knockout rescued the BRD4 degradation induced by **2‐1**, further corroborating the dependency of **2‐1**’s degradative activity on the presence of SKP2 protein.

In conclusion, our finding demonstrates that compound **2‐1** facilitates the ubiquitination of BRD4 and its related family proteins, leading to their subsequent proteasome‐dependent degradation in an SKP2‐dependent manner.

### 2.7 Significant Degradation Activity of SKP2‐Recruiting PROTACs Targeting AR Protein in 22RV1 Cells

To explore the broader applicability of SKP2‐recruiting PROTACs, we investigated their potential to degrade additional target proteins beyond BRD4. We focused on the androgen receptor (AR), a key oncogenic transcription factor in prostate cancer and a subject of interest in prior PROTACs research [55]. To evaluate the capability of our SKP2‐based degrader platform to induce AR degradation, we designed and synthesized compounds **3‐1** to **3‐10** by linking **SL1** with AR antagonists **AL** through various linkers, as detailed in Table 2.

**TABLE 2:** Structure and activity of compounds **3‐1** to **3–10**.

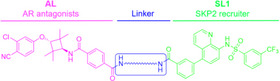			
Cpd	Linker	IC_50_(µM)^a^	D_max_ of AR (%)^b^
		22RV1 Cell	10 µM
**3‐1**	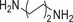	11.2 ± 1.8	46.2
**3‐2**	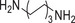	8.9 ± 1.4	49.4
**3‐3**	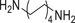	7.4 ± 0.9	58.5
**3‐4**	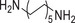	2.1 ± 0.2	95.1
**3‐7**	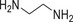	14.8 ± 3.1	50.0
**3‐6**	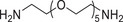	9.7 ± 1.5	60.3
**3‐5**	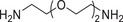	11.9 ± 2	54.1
**3‐8**	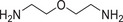	9.9 ± 1.6	55.6
**3‐9**	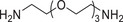	7.1 ± 1.1	68.8
**3‐10**	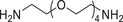	2.9 ± 0.3	91.7
**SL**	—	6.1 ± 0.4	—
**AL**	—	14.58 ± 1	33.7

^a^ The half maximal inhibitory concentration of compounds on the growth of 22RV1 cells.

^b^ Maximum degradation % of AR in 22RV1 cells by compounds.

**FIGURE 7:**
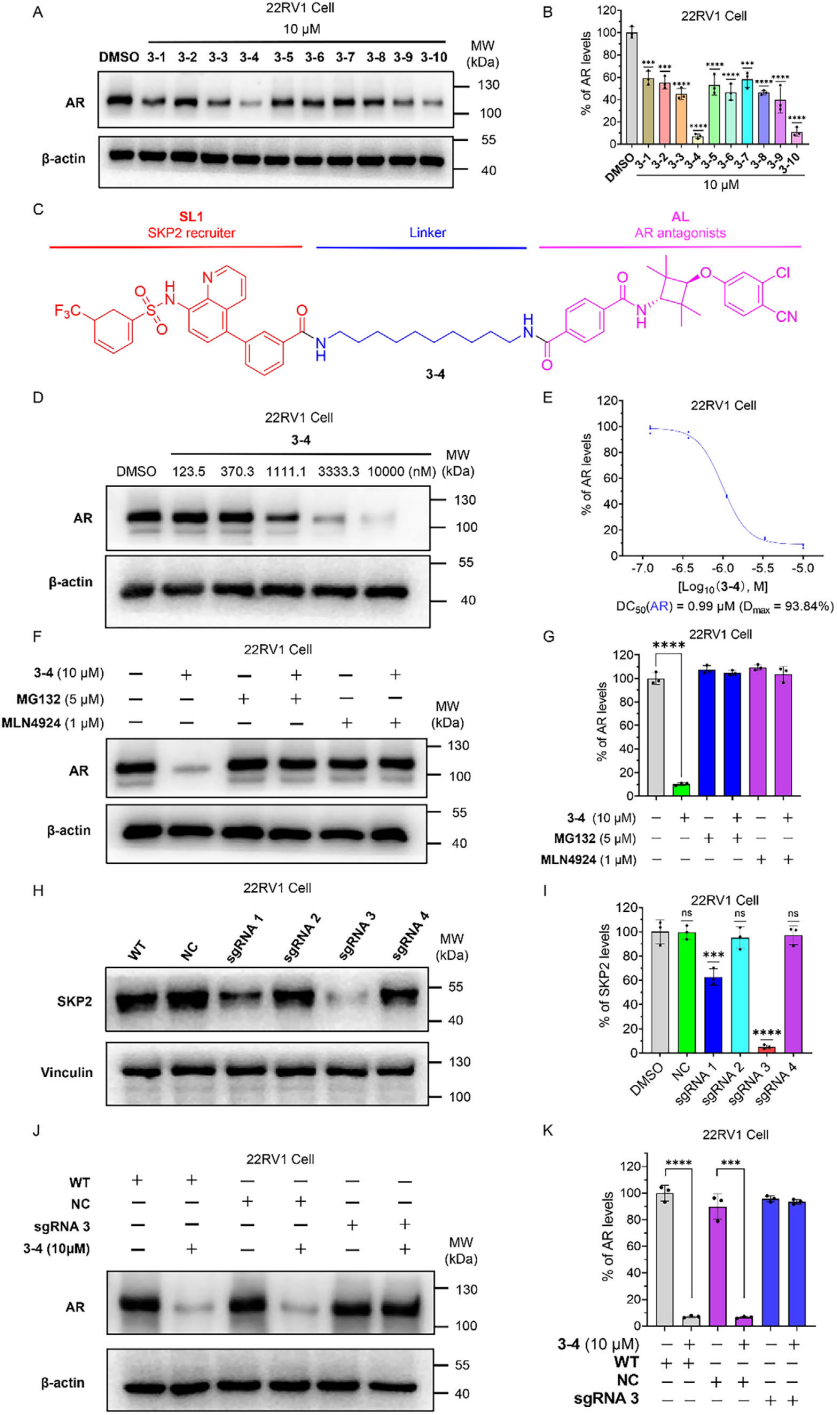
The SKP2‐recruiting PROTACs induce the degradation of AR in 22RV1 cells. (A) After treating 22RV1 cells with DMSO and **3‐1** to **3‐10** (10 µM) for 24 h, the levels of AR and the loading control β‐actin were assessed by Western blotting, and the quantification (B) of AR abundance in the indicated experimental groups is shown. All data are expressed as mean ± SD, based on three independent experiments for each group. *P*‐values were determined by one‐way ANOVA with Dunnett's post hoc test for multiple comparisons. ***p < 0.001, ****p < 0.0001 as compared with the DMSO controls. (C) Structure of the SKP2‐recruiting AR degrader **3‐4**. (D) Immunoblot analysis results after treating 22RV1 cells with DMSO and varying doses of **3‐4** for 24 h, along with (E) quantification of AR abundance, DC_50_ values, and D_max_. All data are presented as mean ± SD, reflecting three independent experiments conducted for each group. (F) The degradation of AR mediated by **3‐4** is blocked by the proteasome inhibitor **MG132** and the neddylation inhibitor **MLN4924**. 22RV1 cells were preincubated with 5 µM **MG132** or 1 µM **MLN4924** for 6 h, followed by treatment with 10 µM **3‐4** along with 5 µM **MG132** or 1 µM **MLN4924** for 24 h, after which the levels of AR and the loading control β‐actin were assessed by Western blotting, with (G) the corresponding quantification results shown in the figure. The data are reported as mean ± SD, reflecting three independent experiments conducted for each group, *P*‐values were determined by one‐way ANOVA with Dunnett's post hoc test for multiple comparisons. ns: *p* > 0.05, *p < 0.05, **p < 0.01, ***p < 0.001, ****p < 0.0001 as compared with the DMSO controls. (H) 22RV1 cells were transfected with LentiCRISPRv2‐CMV‐ZsGreen‐Puro plasmids expressing Cas9 and single‐guide RNAs (sgRNAs) targeting the SKP2 gene to generate stable SKP2 knockout cell lines. Positive clones were selected using puromycin. The efficiency of SKP2 knockout was confirmed by Western blotting, with (I) the corresponding quantification results shown in the figure H. Data are expressed as mean ± SD from three independent experiments per group. *P*‐values were determined by one‐way ANOVA with Dunnett's post hoc test for multiple comparisons. ns: *p* > 0.05, ***p < 0.001, ****p < 0.0001 as compared with the WT controls (J) Wild‐type, negative control, and SKP2 knockout cells were treated with either DMSO or **3‐4** (10 µM) for 24 h. The protein levels of AR, and the loading control β‐actin were then determined by Western blotting, and (K) the corresponding quantitative data are presented. WT: Wild‐type Cell, NC: Negative control cells, cells transfected with lentivirus packaged with blank plasmid. The data are reported as mean ± SD, reflecting three independent experiments conducted for each group. *P*‐values were determined by one‐way ANOVA with Dunnett's post hoc test for multiple comparisons. In the quantification results of AR, ***p < 0.001, ****p < 0.0001 as compared with the DMSO controls.

Initial screening in AR‐overexpressing 22RV1 cells at 10 µM revealed that linker composition significantly influenced degradation efficiency. Compound **3‐4** showed the highest activity, achieving 95.1% AR degradation (Figure 7A,B). Subsequently, we evaluated the anti‐proliferative activity of the AR antagonist **AL** and the novel SKP2‐based AR degraders (compounds **3‐1** to **3‐10**) against AR‐high 22RV1 cells (Table 2). Consistent with previous reports, the parent antagonist itself exhibited only modest activity (IC_50_ = 14.58 µM) [55]. In contrast, our corresponding AR‐based PROTACs demonstrated markedly improved potency. Furthermore, the enhanced anti‐proliferative activity strongly correlated with AR degradation efficiency, with the most effective degrader, compound **3‐4**, also yielding the best IC_50_ value (2.1 µM). Subsequent dose‐response studies with compound **3‐4** demonstrated a DC_50_ of 0.99 µM and a D_max_ of 93.8% (Figure 7D,E).

To elucidate the mechanism of compound **3‐4** mediates AR degradation, we performed mechanistic studies using proteasome and NAE inhibitors. Co‐treatment with the proteasome inhibitor **MG132** prevented **3‐4** induce AR degradation, confirming dependence on the ubiquitin‐proteasome pathway. Additionally, pretreatment with **MLN4924**, an NAE inhibitor, attenuated **3‐4** induced AR degradation, indicating that the compound operates through the CRL E3 ligase complex (Figure 7F,G).

Additionally, to investigate whether small molecule ligands targeting AR or SKP2 individually promote AR degradation, we compared the effects of compound **3‐4** with the AR antagonist **AL** and the SKP2 ligand **SL** on AR protein levels. The results revealed that compound SL did not induce AR degradation in 22RV1 cells, while the AR antagonist **AL** partially promoted AR degradation. However, its effect was markedly weaker than that of **3‐4** (Figure S9A,B).

To further elucidate whether the AR degradation induced by compound **3‐4** is dependent on SKP2, we generated SKP2 knockout 22RV1 cells using CRISPR‐Cas9 (Figure 7H,I). As shown in Figure 7J, compound **3‐4** robustly induced AR degradation in wild‐type and negative control cells, whereas this effect was significantly diminished in SKP2 knockout cells, confirming that the degradation mechanism relies on SKP2.

These findings indicate that non‐covalent SKP2‐recruiting PROTACs can effectively facilitate the degradation of multiple endogenous proteins, including BRD4 and AR, in cellular contexts. All of those validate SKP2‐recruiting PROTACs as a valuable strategy for TPD.

## 3 Conclusions

In this study, we report the development of potent non‐covalent PROTACs that target BRD4 and AR through SKP2 recruitment, demonstrating the feasibility of utilizing SKP2 as an E3 ligase in TPD and expanding the repertoire of E3 ligases capable of mediating ligand‐induced protein degradation.

The human genome encodes over 600 E3 ligases, yet only a few have been successfully utilized in TPD applications. This underscores a critical need to develop novel E3 ligases to advance this field. As the most prominent E3 ligase family, CRL encompasses most E3 ligases used in TPD, such as VHL and CRBN, which serve as substrate receptors for CRL2 and CRL4, respectively. Therefore, we focused on SKP2, the substrate receptor of CRL1, to explore its potential application in TPD. Through literature research, molecular docking, CO‐IP, CETSA, and SPR experiments, we confirmed the binding interaction between SKP2 and the ligand **SL**, ultimately identifying **SL1**, a deprotected derivative of **SL**, as a recruiter for SKP2. We then conjugated **SL1** with the BRD4 inhibitor **JQ1** via various linkers to synthesize a series of bifunctional degraders, which were shown to effectively induce BRD4 degradation in MV‐4‐11 cells. Among them, compound **2‐1** exhibited the highest potency, with a DC_50_ of 298 nM and a D_max_ of 99.6%. Further evaluation across multiple cancer cell lines confirmed that SKP2‐recruited PROTACs can facilitate BRD4 degradation in diverse tumor types, though with varying efficacy depending on cell type. This selectivity should be considered in future development and applications.

Mechanistic studies demonstrate that **2‐1** facilitates the ubiquitination and subsequent degradation of BRD4 in an SKP2‐dependent manner, confirming the feasibility of utilizing SKP2‐based PROTACs for TPD. We observed an intriguing phenomenon wherein **2‐1** significantly promoted the degradation of BRDT, BRD3, and BRD4 but showed a weaker effect on BRD2 degradation. Compound **2‐1**’s differential degradation of BET may involve target conformation specificity and cellular feedback mechanisms. As demonstrated by Hsia et al., conformational selectivity (such as that conferred by a single BD2 residue) can determine E3 recruitment and degradation characteristics [56]. Meanwhile, Riching et al. reported that BET degradation triggers compensatory upregulation of BRD2 synthesis, which may mask sustained degradation [57]. Therefore, the weak degradation of BRD2 by **2‐1** may result from the combined effects of its conformational preference and cellular feedback mechanisms, a specific interplay that requires further in‐depth investigation.

Ultimately, to assess the broader applicability of SKP2‐based PROTACs for protein degradation, we targeted AR and demonstrated that SKP2‐recruited non‐covalent PROTACs efficiently induced AR degradation, highlighting SKP2's potential as a versatile E3 ligase for future TPD applications.

In summary, this study marks the first introduction of SKP2 into the TPD field, filling a gap in the application of CRL1 family E3 ligases and enriching the E3 ligase arsenal for TPD. Previous studies have shown that CRBN, a non‐essential E3 ligase in tumor cells, can lead to resistance by promoting the loss of its copies and reducing its expression levels [58]. In contrast, SKP2, an oncoprotein that is overexpressed in various cancer cells, may help circumvent or at least delay the emergence of therapeutic resistance (Figure S10A,B). Given the tissue selectivity of SKP2, its application in TPD could enable tumor‐selective target protein degradation, significantly reducing PROTAC toxicity and enhancing biosafety in animal models.

However, this study still has some issues and limitations. Although **SL** was identified as a suitable SKP2 recruiter based on literature research and our findings, it may not be the optimal candidate. Given the high rigidity of the SKP2‐recruiting ligand **SL**, we assessed its apparent solubility using turbidimetry and found it to be relatively poor, at only 19.4 µM [59] (Figure S12). The PROTAC molecules **2‐1** to **2‐13**, designed based on **SL**, also exhibit this limitation, with solubility values ranging from 3 to 5 µM, which is suboptimal (Figures S11 and S12). Such solubility issues may hinder their efficacy in target protein degradation and pose significant challenges for their practical application at the animal level. Furthermore, the moderate affinity between SKP2 and **SL** exacerbates these limitations. Therefore, the development of new SKP2 ligands with stronger affinity and improved physicochemical properties is crucial to overcoming these obstacles and enhancing the therapeutic potential of SKP2‐ recruited PROTACs. Our future work will focus on the development of novel SKP2 ligands and the exploration of SKP2‐recruited PROTACs with enhanced degradation activity and improved pharmacokinetic properties, followed by further investigation of their antitumor efficacy and tissue selectivity in animal models. In addition, we are also developing ligands for other tissue‐selective (including Central Nervous System) E3 ligases and exploring their applications in TPD using similar strategies.

## 4 Materials and methods

The comprehensive materials and methods, along with the synthetic routes for the reported compounds, can be found in the Supplementary Information.

### 4.1 Common Reagents and Antibodies

The anti‐BRD4 (#13440) and anti‐BRDT (#93069) Rabbit monoclonal antibodies were purchased from Cell Signaling Technology. The anti‐BRD2 antibody (#49970), anti‐BRD3 antibody (#54675) and anti‐β‐action antibody (#21338) were purchased from Signalway Antibody LLC. The anti‐Ubiquitin antibody (BM4359) were purchased from BOSTER Biological Technology. The anti‐Vinculin antibody (RMAB50122) and HRP Goat Anti‐Rabbit IgG (H+L) (SAB48169) were purchased from Bioswamp Biological Technology. The anti‐AR antibody (22089‐1‐AP) was purchased from Proteintech Group, Inc. The anti‐SKP2 antibody (HA 500427) was purchased from Huabio Biological Technology. The anti‐SKP2 antibody (15010‐1‐AP) was purchased from Proteintech Group, Inc used for CO‐IP experiments. The Clarity and Clarity Max ECL Western Blotting Substrates (#1705061) was purchased from Bio‐Rad. Cell Counting Kit‐8 (CCK‐8) Kit (C0005) was purchased from TargetMol. Compound MG132 (HY‐13259), MLN4924 (HY‐70062), MZ1 (HY‐107425), dBET1 (HY‐101838) and Protein A/G Magnetic Beads (HY‐K0202) were purchased from MedChemExpress (MCE). PEI MAX‐Transfection Grade Linear Polyethylenimine Hydrochloride (MW 40000) was purchased from Polysciences, Inc. The 250 kDa plus protein marker (MP202) was purchased from Vazyme Biotech Co., Ltd. Puromycin (P8230) and BCA Protein Quantification Kit (PC0020) were purchased from Solarbio Science & Technology Co., Ltd. Coomassie brilliant blue solution (P0003S) was purchased from Beyotime Biotechnology.

### 4.2 Cell Culture

MV‐4‐11 cells (RRID: CVCL_0064) were purchased from the cell bank of the Chinese Academy of Sciences and cultured in IMDM medium (GIBCO) containing 10% (v/v) fetal bovine serum (Excell bio, FSP500) in a cell incubator at 37°C with 5% CO_2_. MOLT‐4 cells (RRID: CVCL_0013), MOLM‐13 cells (RRID: CVCL_2119) and 22RV1 cells (RRID: CVCL_1045) were purchased from Guangzhou ewell Bio‐technology Co. Ltd and cultured in 1640 medium containing 10% (v/v) fetal bovine serum (Excell bio, FSP500). NCI‐H2009 cells (RRID: CVCL_1514) was purchased from Guangzhou ewell Bio‐technology Co. Ltd and cultured in DMEM/F12 medium (GIBCO) containing 10% (v/v) FBS in a cell incubator at 37°C with 5% CO_2_. MDA‐MB‐231 (RRID: CVCL_0062) cells and HEK293T cells (RRID: CVCL_0063) were obtained from the research group of Professor Deng Wenbin, School of pharmacy, Sun Yat sen University (Shenzhen), and cultured in DMEM medium containing 10% (v/v) FBS. All cells were cultured in a cell incubator at 37°C with 5% CO_2_, with regular confirmation of sterility.

### 4.3 Protein Expression, Extraction, and Purification

The cDNA fragment encoding the human SKP2 (residues 95–419) was cloned into the pGEX‐4T‐1 expression vector downstream of the sequence for glutathione S‐transferase (GST) tag. To enhance the stability of the purified SKP2 protein, the human SKP1 gene sequence was inserted into the plasmid vector, as the co‐expression of SKP2 and SKP1 can improve SKP2 protein stability [46]. The plasmid vector was constructed by Tsingke Biotechnology Co., Ltd. The resulting recombinant plasmid was transformed into E. coli BL21 (DE3) competent cells for protein expression. A single positive colony was inoculated into LB medium containing 100 µg/mL ampicillin and cultured at 37°C with shaking until the OD_600_ reached 0.6‐0.9. Protein expression was then induced by adding IPTG to a final concentration of 0.4 mM, and the culture was continued at 18°C for 20 h. The cells were harvested by centrifugation at 3900 ×g for 30 min at 4°C. The cell pellet was resuspended in ice‐cold lysis buffer (1×PBS, 200 mM NaCl, 0.3% β‐Mercaptoethanol, 1 mM PMSF, pH 7.4) and lysed by sonication on ice (250 W, sonication 5 s, interval 3 s, 25 min). The cell lysate was clarified by centrifugation at 12500 ×g for 30 min at 4°C to remove cellular debris, and the SKP2 proteins containing the N‐terminal GST tag in the supernatant were purified using a GSTSep Glutathione 4FF Chromatography Column (Yeasen, 20510ES25). Recombinant GST‐SKP2 proteins were finally collected by elution buffer containing reduced glutathione (1 × PBS, 200 mM NaCl, 0.3% β‐mercaptoethanol, 1 mM PMSF, 30 mM reduced glutathione, pH 8.0). Following affinity chromatography, the eluted protein fractions were pooled and subjected to simultaneous concentration, desalting, and buffer exchange (1×PBS, 200 mM NaCl, pH 7.4) using Amicon Ultra centrifugal ultrafiltration tube, 30 kDa MWCO (Millipore, UFC9030). The protein's molecular weight and purity were analyzed by SDS‐PAGE with Coomassie Brilliant Blue staining. The final protein concentration was determined by the Bradford method (Beyotime, P0006C), and the purified protein was aliquoted, flash‐frozen in liquid nitrogen, and stored at ‐80°C for subsequent use.

### 4.4 Co‐Immunoprecipitation (CO‐IP) Assay

MV‐4‐11 cells were plated and treated with compound **22d**, **SL**, **SL1**, **SL2**, **SL3** or an equal volume of DMSO for 8 h. The cells were then lysed for 40 min on ice using RIPA lysis buffer (Beyotime, P0013B), followed by centrifugation at 12000 ×g for 10 min at 4°C to collect the supernatant. The cell lysate was incubated with SKP2 polyclonal antibody (Proteintech Group, Inc, 15010‐1‐AP, 1:30) at 4°C for 16 h. Subsequently, the complex was incubated with protein A/G magnetic beads (MCE, HY‐K0202) at 4°C for 6 h. The immunocomplex was washed four times with lysis buffer, and then 50 µL of 1× protein loading buffer was added. The samples were denatured at 95°C for 8 min before performing Western blotting to detect the levels of SKP2 and CKS1.

### 4.5 Cellular Thermal Shift Assay (CETSA)

MV‐4‐11 cells were seeded in 10 cm culture dishes and treated with the corresponding concentrations of compound **22d**, **SL**, or an equal volume of DMSO for 1 h. The cells were then collected and resuspended in 500 µL of PBS buffer, and evenly distributed into eight 1.5 mL microcentrifuge tubes (EP tubes), with 20 µL taken from each tube as the loading control. The cells were heated for 10 min at the following temperatures: 42°C, 43.9°C, 46.2°C, 49.9°C, 54.4°C, 58.9°C, 63.3°C, and 68.0°C. After heating, the cells were subjected to freeze‐thaw cycles in liquid nitrogen three times. The lysates were then centrifuged at 12 000 ×g at 4°C to collect the supernatant. Finally, 5 × protein loading buffer was added, and the samples were heated at 95°C for 10 min. Western blotting was performed to detect the SKP2 protein and the loading control β‐actin levels. In addition, MV‐4‐11 cells were treated with varying concentrations of **SL** for 1 h. After collection, the cells were heated at 55°C for 10 min and lysed on ice with RIPA lysis buffer for 40 min. The lysates were then centrifuged at 12 000 ×g and 4°C for 30 min. The resulting supernatants were collected, mixed with 5 × protein loading buffer, and boiled at 95°C for 10 min. Finally, SKP2 levels and the loading control β‐actin were assessed by Western Blot analysis.

### 4.6 Molecular Docking

The crystal structure of SKP1‐SKP2‐CKS1‐P27 (PDB 2AST) was used for molecular docking was downloaded from RCSB protein data bank (http://www.pdb.org) [60]. It was used as a template which was transformed to a PDBQT format file after the water and P27 were removed. Based on the binding positions of P27, we selected the seven amino acids W2265, R2294, D2319, R2344, S3041, E3040, and N3045 as the center of the docking box, with a pocket size of 20 Å [44]. The docking analysis was then conducted by Schrödinger Maestro software (Release 2019‐2, Schrödinger LLC, New York, NY, 2019) to predict the possible binding mode of compounds with SKP2 and the results were processed and illustrated using open‐source PyMOL software.

### 4.7 Preparation of Cell Lysates

After treating the cells with DMSO or compounds, adherent cells were digested with trypsin (GIBCO, 25200072), followed by termination of digestion with culture medium. The cells were then centrifuged at 5000 xg for 10 min. For suspended cells, direct centrifugation was performed. The cell pellets were washed twice with pre‐chilled PBS buffer, then lysed under ice bath conditions for 40 min using RIPA lysis buffer (Beyotime, P0013B) supplemented with a protease and phosphatase inhibitor cocktail (Beyotime, P1048) and PMSF (Beyotime, ST507). Following lysis, the samples were centrifuged at 15000 xg for 20 min at 4°C, and the supernatant was collected. Protein concentration was determined using a BCA protein assay kit, after which 5 × SDS‐PAGE sample buffer was added and the samples were denatured in a metal bath at 95°C for 10 min.

### 4.8 Western Blot Analysis

After preparing the protein samples, proteins were separated using 8% ∼ 12% SDS‐PAGE and transferred to a PVDF membrane. The membrane was then blocked with PBS buffer containing 5% non‐fat dry milk at room temperature for 2 h. Subsequently, the samples were incubated overnight at 4°C with the appropriate primary antibodies at suitable concentrations. After washing with PBST, the membrane was treated with HRP Goat Anti‐Rabbit IgG (H+L) at a dilution of 1/5000. Finally, the membrane was imaged using Clarity Max ECL Western Blotting Substrates and a chemiluminescence detection system, and the results were analyzed with ImageJ software.

### 4.9 CCK8 assay

To evaluate the inhibitory effects of the compounds on tumor cell proliferation, suspension cells were seeded at a density of 30000−50000 cells per well, while adherent cells were seeded at 3000−5000 cells per well in a 96‐well plate. Adherent cells were allowed to attach for 12 h before adding different concentrations of the compounds. The MV‐4‐11 cells were then incubated for 72 h in a 37°C incubator with 5% CO_2_. 22RV1 cells were plated at 800 cells / well and incubated in a 37°C incubator containing 5% CO_2_ for 7 days. The solution was changed every 3 days and the drug was added. Finally, 10 µL of CCK‐8 reagent was added to each well and incubated for 4 h, followed by measurement of absorbance at 450 nm using a microplate reader. Data were normalized to the DMSO‐treated cells, and half‐maximal inhibitory concentration (IC_50_) values were calculated and graphed using GraphPad Prism 9.

### 4.10 Immunoprecipitation and Ubiquitination Detection Protocol

To assess ubiquitination, after treating HEK293T cells with DMSO or 10 µM of **2‐1** for 18 h, 10 µM of the proteasome inhibitor **MG132** was added, and the cells were further treated for 6 h, followed by washing with cold PBS. The cells were then resuspended in a lysis buffer containing protease and phosphatase inhibitors and lysed under ice bath conditions for 40 min. After centrifugation at 15000 ×g for 20 min at 4°C, the supernatant was collected. To eliminate non‐specific binding, the lysate was incubated with protein A/G beads for 1 h at 4°C, after which the supernatant was collected again. A specific antibody (anti‐BRD4, 1: 50) was added to the supernatant and incubated gently overnight at 4°C, followed by an additional 2 h incubation with Protein A/G Magnetic Beads. The beads were washed three times with cold washing buffer to remove unbound proteins, and finally, Western blot analysis was performed to detect the levels of protein ubiquitination.

### 4.11 Generation of SKP2 Knockdown in HEK293T Cell Lines

Using PEI MAX—Transfection Grade Linear Polyethylenimine Hydrochloride (MW 40000), HEK293T cells were co‐transfected with shRNA‐containing vectors, PAX2, and VSVG to generate shRNA lentivirus. After 48 h of transfection, the culture medium containing the virus was collected and used to infect HEK293T cells for another 48 h. Following this, the cells were subjected to puromycin selection for 6 days. The proportion of successfully infected cells was observed under a fluorescence microscope, and Western blot analysis was performed to verify SKP2 degradation. The shRNA targeting human SKP2 was obtained from Tsingke Biotech in the pLKO.1‐CMV‐copGFP‐PURO vector, with the sequence described below.

shRNA 1:
CCGGGATAGTGTCATGCTAAAGAATCTCGAGATTCTTTAGCATGACACTATCTTTTTT
shRNA 2:
CCGGGCCTAAGCTAAATCGAGAGAACTCGAGTTCTCTCGATTTAGCTTAGGCTTTTTT
shRNA 3:
CCGGCCATTGCCAGGCCAACTATTGCTCGAGCAATAGTTGGCCTGGCAATGGTTTTTT
shRNA 4:
CCGGCCATTGTCAATACTCTCGCAACTCGAGTTGCGAGAGTATTGACAATGGTTTTTT

### 4.12 Turbidimetric Method for Apparent Solubility Determination

A series of dilutions for each test compound were prepared in PBS. The final DMSO concentration in all test solutions was maintained at a constant level of 0.5% (v/v) to ensure consistent solvent effects and prevent DMSO‐induced precipitation. A PBS solution containing 0.5% DMSO served as the blank control. Following dilution, the 96‐well plate was sealed and subjected to continuous shaking at a constant temperature (25°C) for 30 min to ensure thorough mixing and reach equilibrium. After the incubation period, the absorbance of each well was immediately measured at a wavelength of 650 nm using the microplate reader [59]. The apparent solubility of each compound was defined as the concentration at which precipitation occurred, indicated by an absorbance value exceeding that of the PBS (0.5% DMSO) blank control. To quantitatively determine this endpoint, the linear portion of the absorbance‐concentration curve was fitted, and the intersection point of this fitted line with the baseline absorbance (dashed line), as indicated by the arrow, was calculated as the solubility value. Data for each concentration are presented as the mean of three independent experiments.

### 4.13 SKP2‐SL Binding Assay by Surface Plasmon Resonance (SPR)

Surface plasmon resonance (SPR) assay was performed on the Biacore 1K system (Cytiva) at room temperature. Recombinant purified GST‐SKP2 was covalently immobilized on a CM5 sensor chip (Cytiva, 29104988) using an amine coupling kit (Cytiva, BR100050) according to the manufacturer's standard protocol in the Biacore 1K system. The protein immobilization level was 12506 RU. Immediately before SPR assay, compound SL was dissolved at 10 mM in dimethyl sulfoxide (DMSO) and then diluted into HBS‐EP running buffer (Cytiva, BR100826, pH 7.4, 5% DMSO, 0.05% P20). Interaction assay was performed using a flow rate of 35 µL/min. Contact time was set to 110 s, followed by a dissociation time of 120 s. A reference cell was used to subtract possible nonspecific binding to the chip surface. The raw SPR sensorgrams were collected and analyzed using the Biacore 1K Evaluation Software. The affinity (*K*_D_, dissociation constant), the association rate (*k*_a_), and the dissociation rate (*k*_d_) value were calculated using the Biacore 1K Evaluation Software built‐in module “Kinetic / Affinity” under the “1:1 ligand binding” setting with default setups.

### 4.14 Generation of SKP2 Knockout Cell Lines Using CRISPR/Cas9

SKP2 knockout cell lines were generated in both HEK293T and 22RV1 backgrounds. The 293T Cells were co‐transfected using PEI MAX transfection (MW 40000) reagent with the LentiCRISPRv2‐CMV‐ZsGreen‐Puro vector, which co‐expresses Streptococcus pyogenes Cas9 and a single‐guide RNA (sgRNA) targeting the human SKP2 gene, along with the packaging plasmids PAX2 and VSVG for lentivirus production. The viral supernatant was collected 48 h post‐transfection and used to infect fresh HEK293T and 22RV1 cells. Successfully transduced cells were selected with puromycin (2.5 µg/mL) for 6 days to establish stable polyclonal populations. The knockout efficiency of SKP2 was confirmed at the protein level by Western blot analysis. The target sequence of the sgRNA used is as follows:

sgRNA 1: caccgAAATGATCGTGGGCAGCGGA
sgRNA 2: caccgCCGCTGCCCACGATCATTTA
sgRNA 3: caccgAGCTGCTTAGCAAAGTCTGC
sgRNA 4: caccgAGAATCCAGAACACCCAGAA

### 4.15 Statistical Analysis

The dose‐response curve was generated by fitting the effect (Y) against the logarithmically transformed drug concentration (X) using a four‐parameter logistic nonlinear regression model. The x‐axis is plotted on a logarithmic (Log_10_) scale. The data were expressed as mean ± standard deviation (SD). Three individual experiments were performed for each data. Differences between two groups were analyzed by the two‐tailed Student's t‐test. For comparisons involving more than two groups, one‐way ANOVA was conducted, followed by Dunnett's post hoc test where applicable. All statistical data were calculated using the GraphPad Prism 9 (GraphPad Software Inc., La Jolla, CA, USA). The *p* values were indicated (ns: *p* > 0.05, *p < 0.05, **p < 0.01, ***p < 0.001 and ****p < 0.0001). *P* values less than 0.05 were considered statistically significant.

## Conflicts of Interest

Authors Jianwei Chen, Guanjun Dong, Aima Huang, Ziqing Zhao, Bikai Lai, Xiaohan Xu, and Huiyu Yang are inventors on the patent application (Application No. PCT/CN2025/135528) based on the research presented in this manuscript. The remaining authors declare that they have no competing interests.</m

## Supporting information




**Supporting File**: advs74177‐sup‐0001‐SuppMat.pdf.

## Data Availability

The data that support the findings of this study are available from the corresponding author upon reasonable request.

## References

[1] A. C. Lai and C. M. Crews , “Induced Protein Degradation: An Emerging Drug Discovery Paradigm,” Nature Reviews Drug Discovery 16 (2017): 101–114, 10.1038/nrd.2016.211. 27885283 PMC5684876

[2] S. J. Hughes and A. Ciulli , “Molecular Recognition of Ternary Complexes: A New Dimension in the Structure‐Guided Design of Chemical Degraders,” Essays in Biochemistry 61 (2017): 505–516. 29118097 10.1042/EBC20170041 PMC5869862

[3] G. Lu , R. E. Middleton , H. Sun , et al., “The Myeloma Drug Lenalidomide Promotes the Cereblon‐Dependent Destruction of Ikaros Proteins,” Science 343 (2014): 305–309, 10.1126/science.1244917. 24292623 PMC4070318

[4] J. Krönke , N. D. Udeshi , A. Narla , et al., “Lenalidomide Causes Selective Degradation of IKZF1 and IKZF3 in Multiple Myeloma Cells,” Science 343 (2014): 301–305. 24292625 10.1126/science.1244851 PMC4077049

[5] M. Békés , D. R. Langley , and C. M. Crews , “PROTAC Targeted Protein Degraders: The Past Is Prologue,” Nature Reviews Drug Discovery 21 (2022): 181–200. 35042991 10.1038/s41573-021-00371-6 PMC8765495

[6] Y. Fang , S. Wang , S. Han , et al., “Targeted Protein Degrader Development for Cancer: Advances, Challenges, and Opportunities,” Trends in Pharmacological Sciences 44 (2023): 303–317, 10.1016/j.tips.2023.03.003. 37059054

[7] K. Raina and C. M. Crews , “Targeted Protein Knockdown Using Small Molecule Degraders,” Current Opinion in Chemical Biology 39 (2017): 46–53, 10.1016/j.cbpa.2017.05.016. 28605671 PMC5584562

[8] D. P. Bondeson , A. Mares , I. E. D. Smith , et al., “Catalytic in Vivo Protein Knockdown by Small‐Molecule PROTACs,” Nature Chemical Biology 11 (2015): 611–617, 10.1038/nchembio.1858. 26075522 PMC4629852

[9] D. L. Buckley , I. Van Molle , P. C. Gareiss , et al., “Targeting the von Hippel–Lindau E3 Ubiquitin Ligase Using Small Molecules to Disrupt the VHL/HIF‐1α Interaction,” Journal of the American Chemical Society 134 (2012): 4465–4468. 22369643 10.1021/ja209924v PMC3448299

[10] M. Schapira , M. F. Calabrese , A. N. Bullock , and C. M. Crews , “Targeted Protein Degradation: Expanding the Toolbox,” Nature Reviews Drug Discovery 18 (2019): 949–963, 10.1038/s41573-019-0047-y. 31666732

[11] K. A. Donovan , F. M. Ferguson , J. W. Bushman , et al., “Mapping the Degradable Kinome Provides a Resource for Expedited Degrader Development,” Cell 183 (2020): 1714, 10.1016/j.cell.2020.10.038. 33275901 PMC10294644

[12] A. C. Lai , D. M. Toure , D. D. Hellerschmied , et al., “Modular PROTAC Design for the Degradation of Oncogenic BCR‐ABL,” Angewandte Chemie International Edition 55 (2016): 807–810, 10.1002/anie.201507634. 26593377 PMC4733637

[13] D. P. Bondeson , B. E. Smith , G. M. Burslem , et al., “Lessons in PROTAC Design From Selective Degradation With a Promiscuous Warhead,” Cell Chemical Biology 25 (2018): 78–87.e5, 10.1016/j.chembiol.2017.09.010. 29129718 PMC5777153

[14] H.‐T. Huang , D. Dobrovolsky , J. Paulk , et al., “A Chemoproteomic Approach to Query the Degradable Kinome Using a Multi‐Kinase Degrader,” Cell Chemical Biology 25 (2018): 88–99.e6, 10.1016/j.chembiol.2017.10.005. 29129717 PMC6427047

[15] T. Ishida and A. Ciulli , “E3 Ligase Ligands for PROTACs: How They Were Found and How to Discover New Ones,” SLAS Discovery 26 (2021): 484–502, 10.1177/2472555220965528. 33143537 PMC8013866

[16] Y. Itoh , M. Ishikawa , M. Naito , and Y. Hashimoto , “Protein Knockdown Using Methyl Bestatin−Ligand Hybrid Molecules: Design and Synthesis of Inducers of Ubiquitination‐Mediated Degradation of Cellular Retinoic Acid‐Binding Proteins,” Journal of the American Chemical Society 132 (2010): 5820–5826. 20369832 10.1021/ja100691p

[17] J. Wei , F. Meng , K.‐S. Park , et al., “Harnessing the E3 Ligase KEAP1 for Targeted Protein Degradation,” Journal of the American Chemical Society 143 (2021): 15073–15083, 10.1021/jacs.1c04841. 34520194 PMC8480205

[18] J. Hines , S. Lartigue , H. Dong , Y. Qian , and C. M. Crews , “MDM2‐Recruiting PROTAC Offers Superior, Synergistic Antiproliferative Activity via Simultaneous Degradation of BRD4 and Stabilization of p53,” Cancer Research 79 (2019): 251–262, 10.1158/0008-5472.CAN-18-2918. 30385614 PMC6318015

[19] M. Schröder , M. Renatus , X. Liang , et al., “DCAF1‐Based PROTACs With Activity Against Clinically Validated Targets Overcoming Intrinsic‐ and Acquired‐Degrader Resistance,” Nature Communications 15 (2024): 275, 10.1038/s41467-023-44237-4. PMC10766610 38177131

[20] M. F. Mabanglo , B. Wilson , M. Noureldin , et al., “Crystal Structures of DCAF1‐PROTAC‐WDR5 Ternary Complexes Provide Insight Into DCAF1 Substrate Specificity,” Nature Communications 15 (2024): 10165, 10.1038/s41467-024-54500-x. PMC11585590 39580491

[21] M. Zhao , W. Ma , J. Liang , et al., “Design, Synthesis, and Activity Evaluation of BRD4 PROTAC Based on Alkenyl Oxindole‐DCAF11 Pair,” Journal of Medicinal Chemistry 67 (2024): 19428–19447, 10.1021/acs.jmedchem.4c01767. 39475482

[22] S. C. C. Lucas , A. Ahmed , S. N. Ashraf , et al., “Optimization of Potent Ligands for the E3 Ligase DCAF15 and Evaluation of Their Use in Heterobifunctional Degraders,” Journal of Medicinal Chemistry 67 (2024): 5538–5566, 10.1021/acs.jmedchem.3c02136. 38513086

[23] P. Lu , Y. Cheng , L. Xue , et al., “Selective Degradation of Multimeric Proteins by TRIM21‐based Molecular Glue and PROTAC Degraders,” Cell 187 (2024): 7126–7142.e20, 10.1016/j.cell.2024.10.015. 39488207

[24] C. C. Ward , J. I. Kleinman , S. M. Brittain , et al., “Covalent Ligand Screening Uncovers a RNF4 E3 Ligase Recruiter for Targeted Protein Degradation Applications,” ACS Chemical Biology 14 (2019): 2430–2440, 10.1021/acschembio.8b01083. 31059647 PMC7422721

[25] J. N. Spradlin , X. Hu , C. C. Ward , et al., “Harnessing the Anti‐Cancer Natural Product Nimbolide for Targeted Protein Degradation,” Nature Chemical Biology 15 (2019): 747–755, 10.1038/s41589-019-0304-8. 31209351 PMC6592714

[26] Y. Tao , D. Remillard , E. V. Vinogradova , et al., “Targeted Protein Degradation by Electrophilic PROTACs That Stereoselectively and Site‐Specifically Engage DCAF1,” Journal of the American Chemical Society 144 (2022): 18688–18699, 10.1021/jacs.2c08964. 36170674 PMC10347610

[27] X. Zhang , L. M. Luukkonen , C. L. Eissler , et al., “DCAF11 Supports Targeted Protein Degradation by Electrophilic Proteolysis‐Targeting Chimeras,” Journal of the American Chemical Society 143 (2021): 5141–5149, 10.1021/jacs.1c00990. 33783207 PMC8309050

[28] X. Zhang , V. M. Crowley , T. G. Wucherpfennig , M. M. Dix , and B. F. Cravatt , “Electrophilic PROTACs That Degrade Nuclear Proteins by Engaging DCAF16,” Nature Chemical Biology 15 (2019): 737–746, 10.1038/s41589-019-0279-5. 31209349 PMC6592777

[29] M. Meyers , S. Cismoski , A. Panidapu , B. Chie‐Leon , and D. K. Nomura , “Targeted Protein Degradation Through Recruitment of the CUL4 Complex Adaptor Protein DDB1,” ACS Chemical Biology 19 (2024): 58–68, 10.1021/acschembio.3c00487. 38192078 PMC11003717

[30] S. H. Hong , A. Divakaran , A. Osa , O. W. Huang , I. E. Wertz , and D. K. Nomura , “Exploiting the Cullin E3 Ligase Adaptor Protein SKP1 for Targeted Protein Degradation,” ACS Chemical Biology 19 (2024): 442–450, 10.1021/acschembio.3c00642. 38305738 PMC10999000

[31] N. J. Henning , A. G. Manford , J. N. Spradlin , et al., “Discovery of a Covalent FEM1B Recruiter for Targeted Protein Degradation Applications,” Journal of the American Chemical Society 144 (2022): 701–708, 10.1021/jacs.1c03980. 34994556 PMC8928484

[32] S. Ramachandran , N. Makukhin , K. Haubrich , et al., “Structure‐Based Design of a Phosphotyrosine‐Masked Covalent Ligand Targeting the E3 Ligase SOCS2,” Nature Communications 14 (2023): 6345, 10.1038/s41467-023-41894-3. PMC10564737 37816714

[33] G. Bornstein , J. Bloom , D. Sitry‐Shevah , K. Nakayama , M. Pagano , and A. Hershko , “Role of the SCF^Skp2^ Ubiquitin Ligase in the Degradation of p21^Cip1^ in S Phase,” Journal of Biological Chemistry 278 (2003): 25752–25757, 10.1074/jbc.M301774200. 12730199

[34] A. C. Carrano , E. Eytan , A. Hershko , and M. Pagano , “SKP2 is Required for Ubiquitin‐Mediated Degradation of the CDK Inhibitor p27,” Nature Cell Biology 1 (1999): 193–199, 10.1038/12013. 10559916

[35] T. Kamura , T. Hara , S. Kotoshiba , et al., “Degradation of p57^Kip2^ Mediated by SCF^Skp2^‐Dependent Ubiquitylation,” Proceedings of the National Academy of Sciences 100 (2003): 10231–10236, 10.1073/pnas.1831009100. PMC193544 12925736

[36] C.‐H. Chan , J. K. Morrow , C.‐F. Li , et al., “Pharmacological Inactivation of Skp2 SCF Ubiquitin Ligase Restricts Cancer Stem Cell Traits and Cancer Progression,” Cell 154 (2013): 556–568. 23911321 10.1016/j.cell.2013.06.048 PMC3845452

[37] J. Liu , X. Zheng , W. Li , et al., “Anti‐Tumor Effects of Skp2 Inhibitor AAA‐237 on NSCLC by Arresting Cell Cycle at G0/G1 Phase and Inducing Senescence,” Pharmacological Research 181 (2022): 106259, 10.1016/j.phrs.2022.106259. 35577307

[38] E. Rico‐Bautista , C.‐C. Yang , L. Lu , G. P. Roth , and D. A. Wolf , “Chemical Genetics Approach to Restoring p27Kip1 Reveals Novel Compounds With Antiproliferative Activity in Prostate Cancer Cells,” BMC Biology 8 (2010): 153, 10.1186/1741-7007-8-153. 21182779 PMC3025922

[39] M. Oh , J. H. Lee , H. Moon , Y.‐J. Hyun , and H.‐S. Lim , “A Chemical Inhibitor of the Skp2/p300 Interaction that Promotes p53‐Mediated Apoptosis,” Angewandte Chemie International Edition 55 (2016): 602–606, 10.1002/anie.201508716. 26593157

[40] L. Wu , A. V. Grigoryan , Y. Li , B. Hao , M. Pagano , and T. J. Cardozo , “Specific Small Molecule Inhibitors of Skp2‐Mediated p27 Degradation,” Chemistry & Biology 19 (2012): 1515–1524, 10.1016/j.chembiol.2012.09.015. 23261596 PMC3530153

[41] K. Hu , X.‐J. Li , M. D. Asmamaw , X.‐J. Shi , and H.‐M. Liu , “Establishment of High‐Throughput Screening HTRF Assay for Identification Small Molecule Inhibitors of Skp2‐Cks1,” Scientific Reports 11 (2021): 21105, 10.1038/s41598-021-00646-3. 34702937 PMC8548536

[42] K. Zhang , K. Hu , Q. Li , et al., “Discovery of Novel 1,3‐Diphenylpyrazine Derivatives as Potent S‐Phase Kinase‐Associated Protein 2 (Skp2) Inhibitors for the Treatment of Cancer,” Journal of Medicinal Chemistry 66 (2023): 7221–7242, 10.1021/acs.jmedchem.2c01675. 37204466

[43] R. Singh , A. Sran , D. C. Carroll , et al., “Developing Structure–Activity Relationships From an HTS Hit for Inhibition of the Cks1–Skp2 Protein–Protein Interaction,” Bioorganic & Medicinal Chemistry Letters 25 (2015): 5199–5202. 26463131 10.1016/j.bmcl.2015.09.067

[44] R. J. Rowland , R. Heath , D. Maskell , et al., “Cryo‐EM Structure of SKP1‐SKP2‐CKS1 in Complex With CDK2‐cyclin A‐p27KIP1,” Scientific Reports 13 (2023): 10718, 10.1038/s41598-023-37609-9. 37400515 PMC10318019

[45] Z. Huang , Z. Chen , L. H. Lim , G. C. P. Quang , H. Hirao , and J. S. Zhou , “Weak Arene C─H⋅⋅⋅O Hydrogen Bonding in Palladium‐Catalyzed Arylation and Vinylation of Lactones,” Angewandte Chemie International Edition 52 (2013): 5807–5812, 10.1002/anie.201300481. 23610055

[46] B. A. Schulman , A. C. Carrano , P. D. Jeffrey , et al., “Insights Into SCF Ubiquitin ligases From the Structure of the Skp1–Skp2 Complex,” Nature 408 (2000): 381–386, 10.1038/35042620. 11099048

[47] M. Ding , Y. Shao , D. Sun , et al., “Design, Synthesis, and Biological Evaluation of BRD4 Degraders,” Bioorganic & Medicinal Chemistry 78 (2023): 117134, 10.1016/j.bmc.2022.117134. 36563515

[48] Q. He , L. Zhou , D. Yu , et al., “Near‐Infrared‐Activatable PROTAC Nanocages for Controllable Target Protein Degradation and On‐Demand Antitumor Therapy,” Journal of Medicinal Chemistry 66 (2023): 10458–10472, 10.1021/acs.jmedchem.3c00587. 37279091

[49] G. E. Winter , A. Mayer , D. L. Buckley , et al., “BET Bromodomain Proteins Function as Master Transcription Elongation Factors Independent of CDK9 Recruitment,” Molecular Cell 67 (2017): 5–18.e19, 10.1016/j.molcel.2017.06.004. 28673542 PMC5663500

[50] M. Zengerle , K.‐H. Chan , and A. Ciulli , “Selective Small Molecule Induced Degradation of the BET Bromodomain Protein BRD4,” ACS Chemical Biology 10 (2015): 1770–1777, 10.1021/acschembio.5b00216. 26035625 PMC4548256

[51] P. Filippakopoulos , J. Qi , S. Picaud , et al., “Selective Inhibition of BET Bromodomains,” Nature 468 (2010): 1067–1073, 10.1038/nature09504. 20871596 PMC3010259

[52] B. Zheng , M. Iwanaszko , S. H. A. Soliman , et al., “Ectopic Expression of Testis‐Specific Transcription Elongation Factor in Driving Cancer,” Science Advances 11 (2025): ads4200, 10.1126/sciadv.ads4200. PMC11908497 40085698

[53] L. Zhou , W. Zhang , Y. Sun , and L. Jia , “Protein Neddylation and Its Alterations in human Cancers for Targeted Therapy,” Cellular Signalling 44 (2018): 92–102, 10.1016/j.cellsig.2018.01.009. 29331584 PMC5829022

[54] A. M. Vogl , L. Phu , R. Becerra , et al., “Global Site‐Specific Neddylation Profiling Reveals That NEDDylated Cofilin Regulates Actin Dynamics,” Nature Structural & Molecular Biology 27 (2020): 210–220, 10.1038/s41594-019-0370-3. 32015554

[55] X. Han , C. Wang , C. Qin , et al., “Discovery of ARD‐69 as a Highly Potent Proteolysis Targeting Chimera (PROTAC) Degrader of Androgen Receptor (AR) for the Treatment of Prostate Cancer,” Journal of Medicinal Chemistry 62 (2019): 941–964, 10.1021/acs.jmedchem.8b01631. 30629437

[56] O. Hsia , M. Hinterndorfer , A. D. Cowan , et al., “Targeted Protein Degradation via Intramolecular Bivalent Glues,” Nature 627 (2024): 204–211, 10.1038/s41586-024-07089-6. 38383787 PMC10917667

[57] K. M. Riching , S. Mahan , C. R. Corona , et al., “Quantitative Live‐Cell Kinetic Degradation and Mechanistic Profiling of PROTAC Mode of Action,” ACS Chemical Biology 13 (2018): 2758–2770, 10.1021/acschembio.8b00692. 30137962

[58] X. Zuo and D. Liu , “Mechanism of Immunomodulatory Drug Resistance and Novel Therapeutic Strategies in Multiple Myeloma,” Hematology (Amsterdam, Netherlands) 27 (2022): 1110–1121, 10.1080/16078454.2022.2124694. 36121114

[59] Y. Fan and S. Castleberry , “High‐Throughput Kinetic Turbidity Analysis for Determination of Amorphous Solubility and Excipient Screening for Amorphous Solid Dispersions,” International Journal of Pharmaceutics 631 (2023): 122495, 10.1016/j.ijpharm.2022.122495. 36526147

[60] D. S. Goodsell , C. Zardecki , L. Di Costanzo , et al., “RCSB Protein Data Bank: Enabling Biomedical Research and Drug Discovery,” Protein Science 29 (2020): 52–65, 10.1002/pro.3730. 31531901 PMC6933845

