# Supplementary material for: Discovery of SKP2‐Recruiting PROTACs for Target Protein Degradation
Source: Adv Sci (Weinh). 2026 Feb 4;13(20):e15159. doi: 10.1002/advs.202515159 (PMC13067853; doi:10.1002/advs.202515159)
Supplement: Supplementary file 1 — Supporting File: advs74177‐sup‐0001‐SuppMat.pdf. [file ADVS-13-e15159-s001.docx]

**Supporting Information**

**Discovery of SKP2-recruiting PROTACs for Target Protein Degradation**

Guanjun Dong^1,2,3 ‡^, Aima Huang^1,2,3 ‡^, Ziqing Zhao^1,2,3 ‡^, Bikai Lai^1,2,3^, Xin Pan^1,2,3^, Huiyu Yang^1,2,3^, Xiaohan Xu^1,2,3^, Tianwei Wang^1,2,3^, Fangchen Zhao^4^, Zhimin Zhang^5^, Yongbo Xue^1,2,3^, Guanjun Deng^1,2,3^, Wenbin Deng^1,2,3^, Jianwei Chen^1,2,3*^

[1] G. Dong, A. Huang, Z. Zhao, B. Lai, X. Pan, H. Yang, X. Xu, T. Wang, Prof. Y. Xue, Prof. G. Deng, Prof. W. Deng, Prof. J. Chen

School of Pharmaceutical Sciences (Shenzhen), Shenzhen Campus of Sun Yat-sen University, Shenzhen 518107, P. R. China, E-mail: (chenjw353@mail.sysu.edu.cn)

[2] G. Dong, A. Huang, Z. Zhao, B. Lai, X. Pan, H. Yang, X. Xu, T. Wang, Prof. Y. Xue, Prof. G. Deng, Prof. W. Deng, Prof. J. Chen

Shenzhen Key Laboratory of Neural Cell Reprogramming and Drug Research, Shenzhen Campus of Sun Yat-sen University, Shenzhen 518107, P. R. China

[3] G. Dong, A. Huang, Z. Zhao, B. Lai, H. X. Pan, Yang, X. Xu, T. Wang, Prof. Y. Xue, Prof. G. Deng, Prof. W. Deng, Prof. J. Chen

School of Pharmaceutical Sciences (Shenzhen), Sun Yat-sen University, Shenzhen 518107, P. R. China

[4] F. Zhao

Sun Yat-sen University School of Medicine (Shenzhen), Sun Yat sen University Shenzhen Campus, Shenzhen 518107, P. R. China

[5] Prof. Z. Zhang

College of Pharmacy, Jinan University, Guangzhou, 510632, P. R. China

^‡^ These authors contributed equally.

^*^Corresponding author

Table of Contents

[1. Experimental Procedures 3](#_Toc6840)

[1.1 Synthesis 3](#_Toc31884)

[1.2 Common reagents and antibodies 47](#_Toc809)

[1.3 Cell culture 48](#_Toc21937)

[1.4 Protein expression, extraction, and purification 48](#_Toc10989)

[1.5 Co-Immunoprecipitation (CO-IP) assay 49](#_Toc1267)

[1.6 Cellular Thermal Shift Assay (CETSA) 50](#_Toc26916)

[1.7 Molecular Docking 50](#_Toc10099)

[1.8 Preparation of cell lysates 51](#_Toc9936)

[1.9 Western Blot analysis 51](#_Toc18699)

[1.10 CCK8 assay 51](#_Toc21460)

[1.11 Immunoprecipitation and ubiquitination detection protocol 52](#_Toc10172)

[1.12 Generation of SKP2 knockdown in HEK293T cell lines 52](#_Toc13617)

[1.13 Turbidimetric method for apparent solubility determination 53](#_Toc32630)

[1.14 SKP2-**SL** binding assay by surface plasmon resonance (SPR) 53](#_Toc24100)

[1.15 Generation of SKP2 knockout cell lines using CRISPR/Cas9 54](#_Toc2391)

[1.16 Statistical analysis 54](#_Toc29434)

[2. Supporting tables and figures 56](#_Toc29066)

[2.1 Supplementary Figure 1. Predicted binding models of compounds **14i**, **SYK-031** and **SKPin C1** with the SKP2 protein. 56](#_Toc3043)

[2.2 Supplementary Figure 2. Plasmid vector and SDS-PAGE analysis of the purified GST-SKP2 protein. 57](#_Toc24938)

[2.3 Supplementary Figure 3. SPR sensorgrams showing the interaction between **SL** and SKP2 proteins from three replicate assays. 59](#_Toc32527)

[2.4 Supplementary Figure 4. Bioinformatics analysis of SKP2 and BRD4 expression levels in MV-4-11 cells and the impact of BRD4 knockout on these cells. 60](#_Toc26532)

[2.5 Supplementary Figure 5. Compound **2-6** induces BRD4 degradation in MV-4-11 Cells. 61](#_Toc21704)

[2.6 Supplementary Figure 6. Compound **MZ1** and **dBET1** induces BRD4 degradation in MV-4-11 Cells. 62](#_Toc5729)

[2.7 Supplementary Figure 7. Compound **2-1** induces the bromodomain and extraterminal (BET) family of proteins degradation in NCI-H2009 Cells. 63](#_Toc32120)

[2.8 Supplementary Figure 8. Knockout of SKP2 in HEK293T cells rescued the degradation of BRD4 induced by **2-1**. 64](#_Toc12223)

[2.9 Supplementary Figure 9. AR Ligand **AL** treatment partially degrades AR in 22RV1 Cells. 65](#_Toc24756)

[2.10 Supplementary Figure 10. Bioinformatics analysis of SKP2 and CRBN expression levels in normal and tumor tissues. 66](#_Toc15144)

[2.11 Supplementary Figure 11. Apparent solubility analysis of compounds by the turbidimetric method. 67](#_Toc21954)

[2.12 Supplementary Figure 12. Apparent solubility analysis of compounds by the turbidimetric method. 69](#_Toc29253)

[3. The triplicate data and original bands from the Western blot experiments. 71](#_Toc22840)

[4. NMR spectra 99](#_Toc10909)

[5. References 183](#_Toc12450)

**1. Experimental Procedures**

**1.1 Synthesis**

**General information**

Unless otherwise stated, all reagents and solvents were purchased from commercial suppliers and used directly without further purification. Reactions were monitored by analytical thin layer chromatography (TLC) or Waters HPLC system. Analytical TLC was performed on silica gel GF254, and visualization was carried out with UV light or stained with KMnO_4_. Column chromatography was performed with silica gel (200–300 mesh). NMR spectra data were recorded on a Bruker Advance (^1^H NMR at 500 MHz and ^13^C NMR at 126 MHz) Spectrometer. Chemical shifts (δ) were given in parts per million (ppm) with reference to solvent signals [^1^H NMR: CDCl_3_ (7.26), DMSO-*d*_6_ (2.50); ^13^C NMR: CDCl_3_ (77.2), DMSO-*d*_6_ (39.5)]. The signals were described as br.s (broad singlet), d (doublet), dd (doublet of doublet), m (multiple), q (quarter), s (singlet), and t (triplet). Coupling constants (J values) were given in Hz. Low-resolution mass spectra (LRMS) were obtained by the ESI ionization sources using Agilent 1290 Infinity II-Infinity Lab LC/MSD G6125B or SHIMADZU LCMS-2020. High-resolution mass spectra (HRMS) were obtained by the ESI ionization sources using a Thermo Fisher Orbitrap Exploris 480 Mass Spectrometer.

**Scheme 1.** Ligands or inhibitors of SKP2 protein.

**Scheme 2.** Synthesis route of **SL1**.

**Scheme 3.** Synthesis route of **S5a ~ S5j** and **S7a ~** **S7c**.

**Scheme 4.** Synthesis route of **2-1 ~ 2-13**.

**Scheme 5.** Synthesis route of **AL**. The synthesis process refers to the previous method^[1]^.

**Scheme 6.** Synthesis route of **S12a ~ S12j**.

**Scheme 7.** Synthesis route of **3-1 ~ 3-10**.

**5-Bromoquinolin-8-amine (S1)**

To the solution of 8-Aminoquinoline (2.88 g, 20.0 mmol, 1.00 equiv.) in 20.0 mL of acetonitrile, a portion of NBS (1.78 g, 10.0 mmol, 1.05 equiv.) in acetonitrile was added at 0 ℃ under an argon atmosphere. The reaction mixture was stirred at rt for 15  min, then a second portion of NBS (1.95 g, 11.0 mmol) was added at 0 ℃. The reaction was stirred for 1  h at 25  °C. The solvent was evaporated under reduced pressure and the residue was dissolved with EtOAc and washed with saturated sodium chloride solution (3 × 50 mL). The organic layer was dried over anhydrous sodium sulfate, filtered and concentrated. The residue was purified by flash chromatography on silica gel to afford **S1** as a yellow solid (3.94 g, 88% yield). **^1^H NMR** (500 MHz, CDCl_3_) δ 8.76 (dd, *J* = 4.0, 2.0 Hz, 1H), 8.42 (dd, *J* = 8.5, 2.0 Hz, 1H), 7.56 (d, *J* = 8.5, 1H), 7.47 (dd, *J* = 8.5, 4.0 Hz, 1H), 6.79 (d, *J* = 8.5, 1H), 5.05 (s, 2H). **^13^C NMR** (126 MHz, CDCl_3_) δ 148.0, 144.2, 139.2, 135.6, 131.0, 127.9, ^[1]^22.6, 110.3, 107.5. **MS (ESI)**: m/z 223.0 [M + H]^+^. **HRMS (ESI)**: m/z [M + H]^+^ calcd for C_9_H_7_BrN_2_, 222.9866; found, 222.9865.

***N*-(5-Bromoquinolin-8-yl)-3-(trifluoromethyl)benzenesulfonamide (S2)**

To the solution of **S1** (3.94 g, 17.7 mmol, 1.00 equiv.) in dichloromethane (20 mL) at 0 °C was added 3-(Trifluoromethyl)benzenesulfonyl chloride (6.46 g, 26.5 mmol, 1.50 equiv.), TEA (5.0 mL, 35.3 mmol, 2.00 equiv.) and DMAP (64.7 mg, 0.53 mmol, 0.03 equiv.) in sequence. The reaction was allowed to warm to room temperature and stirred for 12 h under argon conditions. The mixture was extracted with DCM and washed with NaHCO_3_, water, and brine, and dried with Na_2_SO_4_, then concentrated under reduced pressure. The product was purified by flash column chromatography to afford **S2** (5.16 g, 68% yield). **^1^H NMR** (500 MHz, CDCl_3_) δ 9.23 (br.s, 1H), 8.78 (dd, *J* = 4.0, 1.5 Hz, 1H), 8.46 (dd, *J* = 8.5, 1.5 Hz, 1H), 8.17 (s, 1H), 8.04 (d, *J* = 8.0 Hz, 1H), 7.76 – 7.72 (m, 2H), 7.69 (d, *J* = 8.0 Hz, 1H), 7.54 (dd, *J* = 8.5, 4.5 Hz, 1H), 7.52 – 7.49 (m, 1H). **^13^C NMR** (126 MHz, CDCl_3_) δ 149.7, 140.4, 139.4, 136.3, 133.2, 131.8 (q, *J* = 34.0 Hz), 130.5, 130.5, 129.9, 129.9 (q, *J* = 3.8 Hz), 127.8, 124.5 (q, *J* = 3.8 Hz), 123.4, 123.2 (q, *J* = 273.4 Hz), 116.6, 116.2. **MS (ESI)**: m/z 430.9 [M + H]^+^. **HRMS (ESI)**: m/z [M + H]^+^ calcd for C_16_H_10_BrF_3_N_2_O_2_S, 430.9672; found, 430.9670.

***Tert*-butyl3-(8-((3-(trifluoromethyl)phenyl)sulfonamido)quinolin-5-yl)benzoate (S3)**

To a dried round-bottom flask was added **S2** (216 mg, 0.50 mmol, 1.00 equiv.), (3-(*tert*-butoxycarbonyl)phenyl)boronic acid (166 mg, 0.75 mmol, 1.50 equiv.), (Ph_3_P)_2_PdCl_2_ (17.5 mg, 0.025 mmol, 0.05 equiv.) and Na_2_CO_3_ (106 mg, 1.00 mmol, 2.00 equiv.) under argon condition. Then the 1, 4-dioxane : water (3:1) was added and the resulting mixture was stirred at 106 ℃ for 48 h. The reaction mixture was monitored by TLC, and after complete conversion, the mixture was diluted with EtOAc and washed with saturated brine solution. The organic layer was dried with anhydrous sodium sulfate and concentrated under reduced pressure. The residue was purified by silica gel column chromatography to obtain **S3** (151.8 mg, 57% yield). **^1^H NMR** (500 MHz, CDCl_3_) δ 9.36 (br.s, 1H), 8.77 (d, *J* = 4.0 Hz, 1H), 8.19 (s, 1H), 8.14 – 8.10 (m, 2H), 8.06 – 8.04 (m, 1H), 7.99 (s, 1H), 7.91 (d, *J* = 8.0 Hz, 1H), 7.70 (d, *J* = 8.0 Hz, 1H), 7.55 – 7.51 (m, 3H), 7.45 (d, *J* = 7.5 Hz, 1H), 7.40 (dd, *J* = 8.5, 4.0 Hz, 1H), 1.59 (s, 9H). **^13^C NMR** (126 MHz, CDCl_3_) δ 165.6, 149.0, 140.7, 138.9, 138.9, 135.1, 134.8, 134.0, 133.0, 132.7, 131.7 (q, *J* = 32.8 Hz), 130.9, 130.5, 129.9, 129.7 (q, *J* = 3.8 Hz), 128.9, 128.7, 127.6, 126.8, 124.6 (q, *J* = 3.8 Hz), 123.2 (q, *J* = 273.4 Hz), 122.4, 115.7, 81.6, 28.4 (3C). **MS (ESI)**: m/z 529.0 [M + H]^+^. **HRMS (ESI)**: m/z [M + H]^+^ calcd for C_27_H_23_F_3_N_2_O_4_S, 529.1404; found, 529.1401.

**3-(8-((3-(Trifluoromethyl)phenyl)sulfonamido)quinolin-5-yl)benzoic acid (SL1)**

To a solution of **S3** (152 mg, 0.28 mmol) in dichloromethane (4 mL) was added TFA (2 mL) at 0 ℃. The mixture was stirred at room temperature for 30 min, then extracted with CH_2_Cl_2_ (8 mL) and washed with NaHCO_3_ (4 mL). The organic layers were dried with anhydrous sodium sulfate and concentrated under vacuum. The product **SL1** (128.8 mg, 97% yield) was used directly without further purification. **^1^H NMR** (500 MHz, DMSO-*d*_6_) δ 8.82 (dd, *J* = 4.0, 1.5 Hz, 1H), 8.24 (s, 1H), 8.19 (d, *J* = 8.0 Hz, 1H), 8.12 (dd, *J* = 8.5, 1.5 Hz, 1H), 8.03 (dt, *J* = 7.5, 2.0 Hz, 1H), 7.94 – 7.92 (m, 2H), 7.82 (d, *J* = 8.0 Hz, 1H), 7.75 – 7.72 (m, 1H), 7.68 – 7.63 (m, 2H), 7.56 – 7.53 (m, 2H). **^13^C NMR** (126 MHz, DMSO-*d*_6_) δ 167.2, 149.4, 141.0, 139.8, 138.5, 135.0, 134.2, 134.0, 133.2, 131.4, 130.9, 130.7, 130.4, 129.7 (q, *J* = 32.8 Hz), 129.6 (q, *J* = 3.8 Hz), 129.2, 128.7, 127.5, 126.1, 123.8 (q, *J* = 3.8 Hz), 123.3 (q, *J* = 273.4 Hz), 122.7, 119.1. **MS (ESI)**: m/z 473.0 [M + H]^+^. **HRMS (ESI)**: m/z [M + H]^+^ calcd for C_23_H_15_F_3_N_2_O_4_S, 473.0778; found, 473.0775.

***Tert*-butyl4-(8-((3-(trifluoromethyl)phenyl)sulfonamido)quinolin-5-yl)benzoate (SL2)**

The compound **SL2** (12.0 mg, 45% yield) was synthesized according to the same procedures as the **S3** preparation. **^1^H NMR** (500 MHz, CDCl_3_) δ8.77 (dd, *J* = 4.5, 2.0 Hz, 1H), 8.19 (s, 1H), 8.16 (dd, *J* = 8.5, 2.0 Hz, 1H), 8.12 (d, *J* = 8.5 Hz, 1H), 8.09 (d, *J* = 8.5 Hz, 2H), 7.91 (d, *J* = 8.0 Hz, 1H), 7.70 (d, *J* = 8.0 Hz, 1H), 7.55 – 7.52 (m, 1H), 7.45 – 7.43 (m, 3H), 7.39 (dd, *J* = 8.5, 4.0 Hz, 1H), 1.63 (s, 9H). **^13^C NMR** (126 MHz, CDCl_3_) δ 165.6, 149.0, 142.9, 140.8, 138.9, 135.0, 134.7, 133.1, 131.8 (q, *J* = 32.8 Hz), 131.6, 130.5, 130.0 (2C), 129.9, 129.9(2C), 129.8 (q, *J* = 3.8 Hz), 127.5, 126.6, 124.6 (q, *J* = 3.8 Hz), 123.2 (q, *J* = 273.4 Hz), 122.4, 115.5, 81.5, 28.4 (3C). **MS (ESI)**: m/z 529.1 [M + H]^+^. **HRMS (ESI):** m/z [M + H]^+^ calcd for C_27_H_23_F_3_N_2_O_4_S, 529.1404; found, 529.1403.

**N-(5-(pyridin-3-yl)quinolin-8-yl)-3-(trifluoromethyl)benzenesulfonamide (22d)**

The compound **22d** (22.2 mg,40% yield) was synthesized according to the same procedures as the **S3** preparation. **^1^H NMR** (500 MHz, CDCl_3_) δ 9.39 (br.s, 1H), 8.80 (dd, *J* = 4.5, 2.0 Hz, 1H), 8.68 (dd, *J* = 4.5, 2.0 Hz, 1H), 8.65 (d, *J* = 2.0 Hz, 1H), 8.19 (s, 1H), 8.14 – 8.11 (m, 2H), 7.93 (d, *J* = 8.0 Hz, 1H), 7.73 – 7.71 (m, 2H), 7.57 – 7.52 (m, 1H), 7.45 – 7.42 (m, 3H). **^13^C NMR** (126 MHz, CDCl_3_) δ 150.6, 149.2, 149.2, 140.7, 138.9, 137.4, 134.6, 134.3, 133.5, 131.9, 131.8 (q, *J* = 34.0 Hz), 130.5, 130.0, 129.8 (q, *J* = 3.8 Hz), 128.0, 126.8, 124.6 (q, *J* = 3.8 Hz), 123.6, 123.2 (q, *J* = 273.4 Hz) 122.6, 115.4. **MS (ESI)**: m/z 430.0 [M + H]^+^. **HRMS (ESI)**: m/z [M + H]^+^ calcd for C_21_H_14_F_3_N_3_O_2_S, 430.0832; found, 430.0828.

***tert*-butyl(*S*)-(2-(2-(4-(4-chlorophenyl)-2,3,9-trimethyl-6*H*-thieno[3,2-*f*][1,2,4]triazolo[4,3-*a*][1,4]diazepin-6-yl)acetamido)ethyl)carbamate (S4a)**

To the mixture of **(+) JQ-1 carboxylic acid** (40.1 mg, 0.1 mmol, 1.00 equiv.), *tert*-butyl (2-aminoethyl)carbamate (17.6 mg, 0.11 mmol, 1.10 equiv.), EDCI (28.8 mg, 0.15 mmol, 1.50 equiv.), HOBT (27.0 mg, 0.2 mmol, 2.00 equiv.) in anhydrous DMF (1 mL) was added DIPEA (52 µL, 0.3 mmol, 3.00 equiv.). The resulting reaction mixture was stirred at rt for 12 h. The reaction was then extracted with ethyl acetate and a saturated brine solution (3 × 5 mL). The combined organic layers were dried over anhydrous Na_2_SO_4_ and then evaporated under vacuum. The residue was purified by flash column chromatography on silica gel (DCM/MeOH, 40:1 to 20:1) to obtain the product **S4a** (51.4 mg, 95% yield). **^1^H NMR** (500 MHz, CDCl_3_) δ 7.38 (d, *J* = 8.0 Hz, 2H), 7.31 (d, *J* = 9.0 Hz, 2H), 5.49 (br.s, 1H), 4.67 (t, *J* = 7.0 Hz, 1H), 3.56 (dd, *J* = 14.5, 7.5 Hz, 1H), 3.48 – 3.26 (m, 5H), 2.67 (s, 3H), 2.40 (s, 3H), 1.67 (s, 3H), 1.41 (s, 9H). **MS (ESI)**: m/z 565.1 [M + Na]^+^.

***tert*-butyl(*S*)-(4-(2-(4-(4-chlorophenyl)-2,3,9-trimethyl-6*H*-thieno[3,2-*f*][1,2,4]triazolo[4,3-*a*][1,4]diazepin-6-yl)acetamido)butyl)carbamate (S4b)**

The compound **S4b** (55.0 mg, 96% yield) was synthesized from **(+) JQ-1 carboxylic acid** and *tert*-butyl (4-aminobutyl)carbamate according to the same procedures as the **S4a** preparation. **^1^H NMR** (500 MHz, CDCl_3_) δ 7.39 (d, *J* = 8.5 Hz, 2H), 7.33 (d, *J* = 8.5 Hz, 2H), 6.72 (br.s, 1H), 4.74 (br.s, 1H), 4.61 (dd, *J* = 8.0, 6.0 Hz, 1H), 3.56 (dd, *J* = 14.0, 8.0 Hz, 1H), 3.34 – 3.26 (m, 3H), 3.13 – 3.09 (m, 2H), 2.66 (s, 3H), 2.40 (s, 3H), 1.66 (s, 3H), 1.57 – 1.48 (m, 4H), 1.43 (s, 9H). **MS (ESI)**: m/z 593.2 [M + Na]^+^.

***tert*-butyl(*S*)-(8-(2-(4-(4-chlorophenyl)-2,3,9-trimethyl-6*H*-thieno[3,2-*f*][1,2,4]triazolo[4,3-*a*][1,4]diazepin-6-yl)acetamido)octyl)carbamate (S4c)**

The compound **S4c** (56.4 mg, 90% yield) was synthesized from **(+) JQ-1 carboxylic acid** and *tert*-butyl (8-aminooctyl)carbamate according to the same procedures as the **S4a** preparation. **^1^H NMR** (500 MHz, CDCl_3_) δ 7.39 (d, *J* = 8.5 Hz, 2H), 7.32 (d, *J* = 9.0 Hz, 2H), 6.54 (br.s, 1H), 4.61 (dd, *J* = 7.5, 6.0 Hz, 1H), 4.57 (br.s, 1H), 3.55 (dd, *J* = 14.0, 7.5 Hz, 1H), 3.34 – 3.27 (m, 2H), 3.23 – 3.18 (m, 1H), 3.11 – 3.07 (m, 2H), 2.66 (s, 3H), 2.39 (s, 3H), 1.66 (s, 3H), 1.54 – 1.48 (m, 4H), 1.43 (s, 9H), 1.31 – 1.26 (m, 8H). **MS (ESI)**: m/z 649.2 [M + Na]^+^.

***tert*-butyl(*S*)-(10-(2-(4-(4-chlorophenyl)-2,3,9-trimethyl-6*H*-thieno[3,2-*f*][1,2,4]triazolo[4,3-*a*][1,4]diazepin-6-yl)acetamido)decyl)carbamate (S4d)**

The compound **S4d** (59.5 mg, 91% yield) was synthesized from **(+) JQ-1 carboxylic acid** and *tert*-butyl (10-aminodecyl)carbamate according to the same procedures as the **S4a** preparation. **^1^H NMR** (500 MHz, CDCl_3_) δ 7.38 (d, *J* = 8.5 Hz, 2H), 7.30 (d, *J* = 8.0 Hz, 2H), 6.65 (br.s, 1H), 4.61 (t, *J* = 7.5 Hz, 1H), 4.56 (br.s, 1H), 3.52 (dd, *J* = 14.0, 7.5 Hz, 1H), 3.35 – 3.26 (m, 2H), 3.22 – 3.16 (m, 1H), 3.10 – 3.06 (m, 2H), 2.65 (s, 3H), 2.38 (s, 3H), 1.65 (s, 3H), 1.54 – 1.47 (m, 4H), 1.42 (s, 9H), 1.29 – 1.23 (m, 12H). **MS (ESI)**: m/z 677.2 [M + Na]^+^.

***tert*-butyl(*S*)-(2-(2-(2-(4-(4-chlorophenyl)-2,3,9-trimethyl-6*H*-thieno[3,2-*f]*[1,2,4]triazolo[4,3-*a*][1,4]diazepin-6-yl)acetamido)ethoxy)ethyl)carbamate (S4e)**

The compound **S4e** (61.2mg, overweight) was synthesized from **(+) JQ-1 carboxylic acid** and *tert*-butyl (2-(2-aminoethoxy)ethyl)carbamate according to the same procedures as the **S4a** preparation. **^1^H NMR** (500 MHz, CDCl_3_) δ 7.39 (d, *J* = 9.0 Hz, 2H), 7.31 (d, *J* = 9.0 Hz, 2H), 7.14 (br.s, 1H), 5.52 (br.s, 1H), 4.61 (dd, *J* = 7.5, 6.0 Hz, 1H), 3.59 (dd, *J* = 14.5, 8.5 Hz, 1H), 3.54 – 3.50 (m, 4H), 3.46 – 3.45 (m, 2H), 3.35 – 3.30 (m, 3H), 2.65 (s, 3H), 2.39 (s, 3H), 1.66 (s, 3H), 1.43 (s, 9H). **MS (ESI)**: m/z 587.2 [M + H]^+^.

***tert*-butyl (*S*)-(2-(2-(2-(2-(4-(4-chlorophenyl)-2,3,9-trimethyl-6*H*-thieno[3,2-*f*][1,2,4]triazolo[4,3-*a*][1,4]diazepin-6-yl)acetamido)ethoxy)ethoxy)ethyl)carbamate (S4f)**

The compound **S4f** (48.8 mg, 76% yield) was synthesized from **(+) JQ-1 carboxylic acid** and *tert*-butyl (2-(2-(2-aminoethoxy)ethoxy)ethyl)carbamate according to the same procedures as the **S4a** preparation. **^1^H NMR** (500 MHz, CDCl_3_) δ 7.38 (d, *J* = 8.5 Hz, 2H), 7.30 (d, *J* =9.0 Hz, 2H), 7.18 (br.s, 1H), 5.39 (br.s, 1H), 4.62 (t, *J* = 7.0 Hz, 1H), 3.62 – 3.60 (m, 4H), 3.59 – 3.51 (m, 5H), 3.50 – 3.46 (m, 2H), 3.35 – 3.31 (m, 3H), 2.64 (s, 3H), 2.38 (s, 3H), 1.65 (s, 3H), 1.40 (s, 9H). **MS (ESI)**: m/z 653.2 [M + Na]^+^.

***tert*-butyl (*S*)-(1-(4-(4-chlorophenyl)-2,3,9-trimethyl-6*H*-thieno[3,2-*f*][1,2,4]triazolo[4,3-*a*][1,4]diazepin-6-yl)-2-oxo-6,9,12-trioxa-3-azatetradecan-14-yl)carbamate (S4g)**

The compound **S4g** (51.4 mg, 76% yield) was synthesized from **(+) JQ-1 carboxylic acid** and *tert*-butyl(2-(2-(2-(2-aminoethoxy)ethoxy)ethoxy)ethyl) carbamate according to the same procedures as the **S4a** preparation. **^1^H NMR** (500 MHz, CDCl_3_) δ 7.38 (d, *J* = 8.0 Hz, 2H), 7.30 (d, *J* = 8.0 Hz, 2H), 7.09 (br.s, 1H), 5.29 (br.s, 1H), 4.64 (t, *J* = 7.0 Hz, 1H), 3.65 – 3.60 (m, 8H), 3.59 – 3.56 (m, 2H), 3.54 – 3.51 (m, 3H), 3.50 – 3.47 (m, 2H), 3.36 (dd, *J* = 15.0, 7.5 Hz, 1H), 3.31 – 3.27 (m, 2H), 2.64 (s, 3H), 2.37 (s, 3H), 1.64 (s, 3H), 1.40 (s, 9H). **MS (ESI)**: m/z 697.2 [M + Na]^+^.

***tert*-butyl (*S*)-(1-(4-(4-chlorophenyl)-2,3,9-trimethyl-6*H*-thieno[3,2-*f*][1,2,4] triazolo[4,3-*a*][1,4]diazepin-6-yl)-2-oxo-6,9,12,15-tetraoxa-3-azaheptadecan-17-yl) carbamate (S4h)**

The compound **S4h** (53.4 mg, 74% yield) was synthesized from **(+) JQ-1 carboxylic acid** and *tert*-butyl (14-amino-3,6,9,12-tetraoxatetradecyl)carbamate according to the same procedures as the **S4a** preparation. **^1^H NMR** (500 MHz, CDCl_3_) δ 7.37 (d, *J* = 8.5 Hz, 2H), 7.28 (d, *J* = 8.0 Hz, 2H), 5.34 (br.s, 1H), 4.62 (t, *J* = 7.0 Hz, 1H), 3.66 – 3.61 (m, 8H), 3.60– 3.55 (m, 6H), 3.50– 3.44 (m, 5H), 3.36 (dd, *J* = 15.0, 8.0 Hz, 1H), 3.28 – 3.24 (m, 2H), 2.62 (s, 3H), 2.35 (s, 3H), 1.63 (s, 3H), 1.38 (s, 9H). **MS (ESI)**: m/z 719.2 [M + H]^+^.

***tert*-butyl (*S*)-(1-(4-(4-chlorophenyl)-2,3,9-trimethyl-6*H*-thieno[3,2-*f*][1,2,4] triazolo[4,3-*a*][1,4]diazepin-6-yl)-2-oxo-6,9,12,15,18-pentaoxa-3-azaicosan-20-yl)carbamate (S4i)**

The compound **S4i** (68.7 mg, 90% yield) was synthesized from **(+) JQ-1 carboxylic acid** and *tert*-butyl (17-amino-3,6,9,12,15-pentaoxaheptadecyl)carbamate according to the same procedures as the **S4a** preparation. **^1^H NMR** (500 MHz, CDCl_3_) δ 7.38 (d, *J* = 8.5 Hz, 2H), 7.29 (d, *J* = 8.5 Hz, 2H), 7.15 (br.s, 1H), 5.21 (br.s, 1H), 4.63 (t, *J* = 7.0 Hz, 1H), 3.65 – 3.61 (m, 12H), 3.61 – 3.55 (m, 6H), 3.50 – 3.46 (m, 5H), 3.37 (dd, *J* = 15.0, 7.5 Hz, 1H), 3.28 – 3.25 (m, 2H), 2.63 (s, 3H), 2.36 (s, 3H), 1.64 (s, 3H), 1.40 (s, 9H). **MS (ESI)**: m/z 785.2 [M + Na]^+^.

***tert*-butyl(*S*)-4-(2-(2-(4-(4-chlorophenyl)-2,3,9-trimethyl-6*H*-thieno[3,2-*f*][1,2,4]triazolo[4,3-*a*][1,4]diazepin-6-yl)acetamido)ethyl)piperazine-1-carboxylate (S4j)**

The compound **S4j** (42.0 mg, 69% yield) was synthesized from **(+) JQ-1 carboxylic acid** and *tert*-butyl 4-(2-aminoethyl)piperazine-1-carboxylate according to the same procedur as the **S4a** preparation. **^1^H NMR** (500 MHz, CDCl_3_) δ 7.36 (d, *J* = 8.5 Hz, 2H), 7.28 (d, *J* = 7.0 Hz, 2H), 4.62 (t, *J* = 7.0 Hz, 1H), 3.56 – 3.46 (m, 3H), 3.42 (t, *J* = 5.0 Hz, 4H), 3.34 (dd, *J* = 15.0, 6.5 Hz, 1H), 2.67 – 2.64 (m, 2H), 2.62 (s, 3H), 2.53 (t, *J* = 6.0 Hz, 4H), 2.36 (s, 3H), 1.64 (s, 3H), 1.42 (s, 9H). **MS (ESI)**: m/z 612.1 [M + H]^+^.

***tert*-butyl(*S*)-(8-((2-(2-(4-(4-chlorophenyl)-2,3,9-trimethyl-6*H*-thieno[3,2-*f*][1,2,4]triazolo[4,3-*a*][1,4]diazepin-6-yl)acetamido)ethyl)amino)-8-oxooctyl)carbamate (S6a)**

To the solution of **S4a** (40.1mg, 0.074 mmol) in re-distilled dichloromethane (0.5 mL) was added TFA (0.25 mL) slowly at 0 ℃, and the mixture was stirred for 0.5 h in an ice bath. Then the mixture was diluted with DCM and evaporated to obtain the **S5a** salt form for the next step without further purification.

To the solution of 8-((*tert*-butoxycarbonyl)amino)octanoic acid (17.1 mg, 0.066 mmol, 1.00 equiv.), **S5a** (40.1 mg, 0.07 mmol, 1.10 equiv.), EDCI (17.2 mg, 0.09 mmol, 1.50 equiv.), HOBT (16.2 mg, 0.12 mmol, 2.00 equiv.) in anhydrous DMF (0.5 mL) was added DIPEA (62 µL, 0.36 mmol, 6.00 equiv.). The resulting reaction mixture was stirred at rt for 12 h. The reaction was then extracted with ethyl acetate and a saturated brine solution (3 × 5 mL). The combined organic layers were dried over anhydrous Na2SO4 and then evaporated under vacuum. The residue was purified by flash column chromatography on silica gel (DCM/MeOH, 40:1 to 20:1) to obtain the product **S6a** (10.0 mg, 22% yield). **^1^H NMR** (500 MHz, CDCl_3_) δ 7.39 (d, *J* = 8.0 Hz, 2H), 7.32 (d, *J* = 8.5 Hz, 2H), 6.78 (br.s, 1H), 4.64 (dd, *J* = 8.0, 6.0 Hz, 1H), 4.60 (br.s, 1H), 3.54 (dd, *J* = 14.5, 8.5 Hz, 1H), 3.49 – 3.41 (m, 2H), 3.37 – 3.31 (m, 3H), 3.08 – 3.04 (m, 2H), 2.66 (s, 3H), 2.40 (s, 3H), 2.15 – 2.09 (m, 4H), 1.67 (s, 3H), 1.60 – 1.54 (m, 2H), 1.42 (s, 9H), 1.27 – 1.25 (m, 6H). **MS (ESI)**: m/z 706.2 [M + Na]^+^.

***tert*-butyl(*S*)-(8-((4-(2-(4-(4-chlorophenyl)-2,3,9-trimethyl-6*H*-thieno[3,2-*f*][1,2,4]triazolo[4,3-*a*][1,4]diazepin-6-yl)acetamido)butyl)amino)-8-oxooctyl)carbamate (S6b)**

The compound **S6b** (13.1 mg, 32% yield) was synthesized from **S5b** and 8-((*tert*-butoxycarbonyl)amino)octanoic acid according to the same procedures as the **S6a** preparation. **^1^H NMR** (500 MHz, CDCl_3_) δ 7.39 (d, *J* = 8.0 Hz, 2H), 7.32 (d, *J* = 8.0 Hz, 2H), 6.99 (br.s, 1H), 6.24 (br.s, 1H), 4.64 – 4.61 (m, 2H), 3.56 (dd, *J* = 14.0, 8.5 Hz, 1H), 3.35 – 3.28 (m, 2H), 3.25 – 3.20 (m, 3H), 3.09 – 3.04 (m, 2H), 2.65 (s, 3H), 2.39 (s, 3H), 2.15 (t, *J* = 7.5 Hz, 2H), 1.66 (s, 3H), 1.61– 1.57 (m, 4H), 1.54 – 1.51 (m, 4H), 1.42 (s, 9H), 1.29 – 1.26 (m, 6H). **MS (ESI)**: m/z 734.3 [M + Na]^+^.

***tert*-butyl(*S*)-(8-(4-(2-(2-(4-(4-chlorophenyl)-2,3,9-trimethyl-6*H*-thieno[3,2-*f*][1,2,4]triazolo[4,3-*a*][1,4]diazepin-6-yl)acetamido)ethyl)piperazin-1-yl)-8-oxooctyl)carbamate (S6c)**

The compound **S6c** (14.0 mg, 32% yield) was synthesized from **S5j** and 8-((*tert*-butoxycarbonyl)amino)octanoic acid according to the same procedures as the **S6a** preparation. **^1^H NMR** (500 MHz, CDCl_3_) δ 7.39 (d, *J* = 8.5 Hz, 2H), 7.32 (d, *J* = 8.5 Hz, 2H), 6.97 (br.s, 1H), 4.63 (dd, *J* = 7.5, 6.0 Hz, 1H), 4.53 (br.s, 1H), 3.63 – 3.55 (m, 3H), 3.46 – 3.44 (m, 2H), 3.42 – 3.32 (m, 3H), 3.11 – 3.06 (m, 2H), 2.65 (s, 3H), 2.55 – 2.47 (m, 6H), 2.39 (s, 3H), 2.29 (t, *J* = 7.5 Hz, 2H), 1.66 (s, 3H), 1.62 – 1.58 (m, 2H), 1.48 – 1.45 (m, 2H), 1.42 (s, 9H), 1.34 – 1.29 (m, 6H). **MS (ESI)**: m/z 753.3 [M + H]^+^.

**(*S*)-*N*-(2-(2-(4-(4-chlorophenyl)-2,3,9-trimethyl-6*H*-thieno[3,2-*f*][1,2,4]triazolo[4,3**

**-*a*][1,4]diazepin-6-yl)acetamido)ethyl)-3-(8-((3-(trifluoromethyl)phenyl)**

**sulfonamido)quinolin-5-yl)benzamide (2-1)**

To the solution of **S4a** (320 mg, 0.59 mmol) in re-distilled dichloromethane (0.5 mL) was added TFA (0.25 mL) slowly at 0 ℃, and the mixture was stirred for 0.5 h in an ice bath. Then the mixture was diluted with DCM and evaporated to obtain the **S5a** salt form for the next step without further purification.

To the solution of **SL1** (26.0 mg, 0.055 mmol, 1.00 equiv.) in anhydrous DMSO (1.5 mL) were added **S5a** (36.8 mg, 0.066 mmol, 1.20 equiv.), EDCI (15.8 mg, 0.083 mmol, 1.50 equiv.), HOBT (14.9 mg, 0.11 mmol, 2.00 equiv.) and DIPEA (48 μL, 0.28 mmol, 5.00 equiv.). After being stirred at rt overnight, the reaction solution was extracted with ethyl acetate, then washed with water, and a saturated brine solution. The organic layers were dried over anhydrous Na_2_SO_4_, and the solvent was evaporated. The product was purified by flash column chromatography on silica gel (DCM/MeOH, 30:1) to afford Compound **2-1** (21.7 mg, 44% yield). **^1^H NMR** (500 MHz, CDCl_3_) δ 8.65 (d, *J* = 4.0 Hz, 1H), 8.17 (s, 1H), 8.11 (dd, *J* = 8.5, 1.5 Hz, 1H), 8.04 (d, *J* = 8.0 Hz, 1H), 8.01 – 7.80 (m, 2H), 7.86 – 7.84 (m, 1H), 7.82 (d, *J* = 7.5 Hz, 1H), 7.76 – 7.74 (m, 1H), 7.67 (d, *J* = 7.5 Hz, 1H), 7.51 – 7.48 (m, 1H), 7.43 – 7.41 (m, 2H), 7.38 (d, *J* = 8.0 Hz, 1H), 7.32 (d, *J* = 8.5 Hz, 2H), 7.29 (dd, *J* = 8.5, 4.0 Hz, 1H), 7.23 (d, *J* = 8.5 Hz, 2H), 4.64 (t, *J* = 7.5 Hz, 1H), 3.71 – 3.67 (m, 1H), 3.63 – 3.58 (m, 1H), 3.54 – 3.46 (m, 3H), 3.42 (dd, *J* = 14.5, 6.5 Hz, 1H), 2.48 (s, 3H), 2.37 (s, 3H), 1.60 (s, 3H). **^13^C NMR** (126 MHz, CDCl_3_) δ 172.0, 167.4, 164.3, 155.8, 150,0 148.8, 140.6, 139.0, 138.8, 137.0, 136.5, 135.0, 134.9, 134.8, 132.7, 132.7, 132.1, 131.7 (q, *J* = 34.0 Hz), 131.3, 131.1, 130.6, 130.5, 129.9 (2C), 129.9, 129.7 (q, *J* = 3.8 Hz), 129.3, 128.8 (2C), 128.8, 127.6, 126.6, 126.4, 124.5 (q, *J* = 3.8 Hz), 123.2 (q, *J* = 273.4 Hz), 122.3, 115.5, 54.4, 41.1, 39.4, 39.2, 14.5, 13.2, 11.8. **MS (ESI)**: m/z 897.2 [M + H]^+^. **HRMS (ESI)**: m/z [M + H]^+^ calcd for C_44_H_36_ClF_3_N_8_O_4_S_2_, 897.2015; found, 897.2014.

**(*S*)-*N*-(4-(2-(4-(4-chlorophenyl)-2,3,9-trimethyl-6*H*-thieno[3,2-*f*][1,2,4]triazolo[4,3**

**-*a*][1,4]diazepin-6-yl)acetamido)butyl)-3-(8-((3-(trifluoromethyl)phenyl)**

**sulfonamido)quinolin-5-yl)benzamide (2-2)**

The compound **2-2** (63.6 mg, overweight) was synthesized from **SL1** and **S5b** according to the same standard procedures of the **2-1** preparation. **^1^H NMR** (500 MHz, CDCl_3_) δ 8.65 (d, *J* = 4.0 Hz, 1H), 8.11 (s, 1H), 8.07 (d, *J* = 9.0 Hz, 1H), 8.00 (d, *J* = 8.0 Hz, 1H), 7.86 – 7.85 (m, 2H), 7.79 (d, *J* = 8.0 Hz, 1H), 7.62 (d, *J* = 8.0 Hz, 1H), 7.46 – 7.38 (m, 3H), 7.35 (d, *J* = 8.0 Hz, 1H), 7.32 (d, *J* = 8.5 Hz, 2H), 7.30 – 7.21 (m, 4H), 6.99 – 6.96 (m, 1H), 4.55 (t, *J* = 6.5 Hz, 1H), 3.48 – 3.39 (m, 2H), 3.35 – 3.27 (m, 2H), 3.23 (dd, *J* = 14.5, 5.5 Hz, 1H), 3.19 – 3.12 (m, 1H), 2.47 (s, 3H), 2.31 (s, 3H), 1.61 – 1.52 (m, 7H). **^13^C NMR** (126 MHz, CDCl_3_) δ 170.7, 167.3, 164.1, 155.7, 150.0, 148.9, 140.5, 138.9, 138.8, 136.9, 136.6, 135.4, 135.1, 134.8, 132.7, 132.6, 132.1, 131.6 (q, *J* = 34.0 Hz), 131.1, 131.0, 130.5, 130.4, 129.9 (2C), 129.8, 129.7 (q, *J* = 3.8 Hz), 129.0, 128.8 (2C), 128.7, 127.5, 126.6, 126.5, 124.4 (q, *J* = 3.8 Hz), 123.1 (q, *J* = 273.4 Hz), 122.3, 115.6, 54.6, 39.8, 39.3, 39.0, 26.9, 26.5, 14.4, 13.1, 11.7. **MS (ESI)**: m/z 925.2 [M + H]^+^. **HRMS (ESI)**: m/z [M + H]^+^ calcd for C_46_H_40_ClF_3_N_8_O_4_S_2_, 925.2328; found, 925.2426.

**(*S*)-*N*-(8-(2-(4-(4-chlorophenyl)-2,3,9-trimethyl-6*H*-thieno[3,2-*f*][1,2,4]triazolo[4,3**

**-*a*][1,4]diazepin-6-yl)acetamido)octyl)-3-(8-((3-(trifluoromethyl)phenyl)**

**sulfonamido)quinoline-5-yl)benzamide (2-3)**

The compound **2-3** (36.8 mg, 68% yield) was synthesized from **SL1** and **S5c** according to the same procedures as the **2-1** preparation. **^1^H NMR** (500 MHz, CDCl_3_) δ 8.69 (d, *J* = 4.0 Hz, 1H), 8.12 (s, 1H), 8.07 (d, *J* = 8.5 Hz, 1H), 8.03 (d, *J* = 8.0 Hz, 1H), 7.82 (d, *J* = 7.5 Hz, 1H), 7.77 – 7.76 (m, 2H), 7.64 (d, *J* = 8.0 Hz, 1H), 7.48 – 7.43 (m, 2H), 7.40 (d, *J* = 7.5 Hz, 1H), 7.37 – 7.31 (m, 4H), 7.24 – 7.23 (m, 2H), 6.70 (t, *J* = 6.0, 1H), 6.66 (t, *J* = 6.0, 1H), 4.55 (t, *J* = 7.0 Hz, 1H), 3.47 (dd, *J* = 14.5, 7.5 Hz, 1H), 3.39 – 3.35 (m, 2H), 3.26 (dd, *J* = 14.5, 4.5 Hz, 1H), 3.22 – 3.14 (m, 2H), 2.58 (s, 3H), 2.33 (s, 3H), 1.60 (s, 3H), 1.56 – 1.51 (m, 2H), 1.46 – 1.42 (m, 2H), 1.31 – 1.23 (m, 8H). **^13^C NMR** (126 MHz, CDCl_3_) δ 170.5, 167.3, 164.0, 155.8, 150.0, 149.0, 140.6, 139.1, 138.8, 136.9, 136.7, 135.5, 135.0, 134.7, 132.9, 132.8, 132.2, 131.6 (q, *J* = 34.0 Hz) 131.1, 131.0, 130.6, 130.5, 130.0 (2C), 129.9, 129.7 (q, *J* = 3.8 Hz), 128.9, 128.8 (2C), 128.8, 127.5, 126.7, 126.3, 124.5 (q, *J* = 3.8 Hz), 123.2 (q, *J* = 273.4 Hz), 122.4, 115.6, 54.6, 40.3, 39.7, 39.5, 29.6, 29.5, 29.2, 29.1, 26.9, 26.8, 14.5, 13.2, 11.9. **MS (ESI)**: m/z 981.2 [M + H]^+^. **HRMS (ESI)**: m/z [M+H]^+^ calcd for C_50_H_48_ClF_3_N_8_O_4_S_2_, 981.2954; found, 981.2952.

**(*S*)-*N*-(10-(2-(4-(4-chlorophenyl)-2,3,9-trimethyl-6*H*-thieno[3,2-*f*][1,2,4]triazolo[4,**

**3-*a*][1,4]diazepin-6-yl)acetamido)decyl)-3-(8-((3-(trifluoromethyl)phenyl)**

**sulfonamido)quinolin-5-yl)benzamide (2-4)**

The compound **2-4** (22.7 mg, 41% yield) was synthesized from **SL1** and **S5d** according to the same procedures as the **2-1** preparation. **^1^H NMR** (500 MHz, CDCl_3_) δ 8.74 (d, *J* = 4.5 Hz, 1H), 8.17 (s, 1H), 8.11 (d, *J* = 8.5 Hz, 1H), 8.08 (d, *J* = 8.0 Hz, 1H), 7.87 (d, *J* = 8.0 Hz, 1H), 7.80 – 7.78 (m, 2H), 7.68 (d, *J* = 8.0 Hz, 1H), 7.52 – 7.48 (m, 2H), 7.46 (d, *J* = 7.5 Hz, 1H), 7.41 (d, *J* = 8.0 Hz, 1H), 7.38 – 7.35 (m, 3H), 7.29 (d, *J* = 8.0 Hz, 2H), 6.62 (br.s, 1H), 6.47 (br.s, 1H), 4.60 (t, *J* =7.0 Hz, 1H), 3.51 (dd, *J* = 14.5, 7.5 Hz, 1H), 3.44 – 3.40 (m, 2H), 3.34 – 3.25 (m, 2H), 3.23 – 3.16 (m, 1H), 2.64 (s, 3H), 2.38 (s, 3H), 1.65 (s, 3H), 1.61 – 1.55 (m, 2H), 1.53 – 1.47 (m, 2H), 1.37 – 1.24 (m, 12H). **^13^C NMR** (126 MHz, CDCl_3_) δ 170.5, 167.3, 164.0, 155.8, 150.0, 149.0, 140.6, 139.1, 138.8, 136.9, 136.7, 135.5, 135.0, 134.7, 132.9, 132.8, 132.2, 131.6 (q, *J* = 34.0 Hz) 131.0, 131.0, 130.6, 130.5, 130.0 (2C), 129.9, 129.7 (q, *J* = 3.8 Hz), 128.9, 128.8 (2C), 128.8, 127.6, 126.7, 126.2, 124.5 (q, *J* = 3.8 Hz), 123.2 (q, *J* = 273.4 Hz), 122.4, 115.6, 54.7, 40.4, 39.8, 39.6, 29.7, 29.6, 29.4, 29.4, 29.3, 29.3, 27.1, 26.9, 14.5, 13.2, 11.9. **MS (ESI)**: m/z 1009.3 [M + H]^+^. **HRMS (ESI)**: m/z [M+H]^+^ calcd for C_52_H_52_ClF_3_N_8_O_4_S_2_, 1009.3267;found,1009.3266.

**(*S*)-*N*-(8-((4-(2-(4-(4-chlorophenyl)-2,3,9-trimethyl-6*H*-thieno[3,2-*f*][1,2,4]triazolo**

**[4,3-*a*][1,4]diazepin-6-yl)acetamido)butyl)amino)-8-oxooctyl)-3-(8-((3-(**

**trifluoromethyl)phenyl)sulfonamido)quinolin-5-yl)benzamide (2-5)**

The compound **2-5** (20.5 mg, 35% yield) was synthesized from **SL1** and **S7b** according to the same procedures as the **2-1** preparation. **^1^H NMR** (500 MHz, CDCl_3_) δ 8.75 (d, *J* = 4.0 Hz, 1H), 8.17 (s, 1H), 8.11 (d, *J* = 8.5 Hz, 1H), 8.08 (d, *J* = 8.0 Hz, 1H), 7.87 (d, *J* = 7.5 Hz, 1H), 7.82 – 7.81 (m, 2H), 7.69 (d, *J* = 8.0 Hz, 1H), 7.53 – 7.49 (m, 2H), 7.46 (d, *J* = 7.5 Hz, 1H), 7.41 (d, *J* = 7.5 Hz, 1H), 7.39 – 7.36 (m, 3H), 7.31 (d, *J* = 8.5 Hz, 2H), 6.91 (t, *J* = 6.0 Hz, 1H), 6.66 (t, *J* = 6.0 Hz, 1H), 6.26 (t, *J* = 6.0 Hz, 1H), 4.60 (dd, *J* = 8.0, 5.5 Hz, 1H), 3.53 (dd, *J* = 14.5, 8.5 Hz, 1H), 3.43 – 3.38 (m, 2H), 3.35 – 3.25 (m, 2H), 3.23 – 3.15 (m, 3H), 2.63 (s, 3H), 2.39 (s, 3H), 2.15 (t, *J* = 7.5 Hz, 2H), 1.65 (s, 3H), 1.60 – 1.49 (m, 8H), 1.34 – 1.28 (m, 6H). **^13^C NMR** (126 MHz, CDCl_3_) δ 173.8, 170.8, 167.4, 164.2, 155.7, 150.1, 149.0, 140.5, 139.0, 138.8, 137.0, 136.6, 135.4, 135.0, 134.7, 132.8, 132.8, 132.1, 131.6 (q, *J* = 32.8 Hz), 131.3, 131.0, 130.6, 130.5, 130.0 (2C), 129.9, 129.8 (q, *J* = 3.8 Hz), 128.9, 128.8 (2C), 128.8, 127.5, 126.6, 126.4, 124.4 (q, *J* = 3.8 Hz), 123.2 (q, *J* = 273.4 Hz), 122.4, 115.5, 54.5, 40.2, 39.1, 39.1, 39.1, 36.6, 29.5, 29.0, 28.8, 26.9, 26.8, 26.7, 25.6, 14.5, 13.2, 11.9. **MS (ESI)**: m/z 1064.1 [M - H]^-^. **HRMS (ESI)**: m/z [M + H]^+^ calcd for C_54_H_55_ClF_3_N_9_O_5_S_2_, 1066.3481; found, 1066.3480.

**(*S*)-2-(4-(4-chlorophenyl)-2,3,9-trimethyl-6*H*-thieno[3,2-*f*][1,2,4]triazolo[4,3-*a*][1,**

**4]diazepin-6-yl)-*N*-(2-(4-(3-(8-((3-(trifluoromethyl)phenyl)sulfonamido)quinoline**

**-5-yl)benzoyl)piperazin-1-yl)ethyl)acetamide (2-6)**

The compound **2-6** (20.8 mg, 39 % yield) was synthesized from **SL1** and **S5j** according to the same procedures as the **2-1** preparation. **^1^H NMR** (500 MHz, CDCl_3_) δ8.76 (d, *J* = 4.0, Hz, 1H), 8.19 – 8.16 (m, 2H), 8.10 (d, *J* = 8.0 Hz, 1H), 7.90 (d, *J* = 8.0 Hz, 1H), 7.69 (d, *J* =7.5 Hz, 1H), 7.53 – 7.51 (m, 2H), 7.46 – 7.43 (m, 3H), 7.42 – 7.38 (m, 4H), 7.31 (d, *J* = 8.5 Hz, 2H), 6.96 (br.s, 1H), 4.62 (t, *J* = 7.0 Hz, 1H), 3.82 – 3.75 (m, 2H), 3.58 (dd, *J* = 14.5, 8.0 Hz, 1H), 3.53 – 3.45 (m, 2H), 3.44 – 3.31 (m, 3H), 2.63 (s, 3H), 2.59 – 2.46 (m, 6H), 2.39 (s, 3H), 1.66 (s, 3H). **^13^C NMR** (126 MHz, CDCl_3_) δ 170.6, 169.9, 164.2, 155.8, 149.0, 140.7, 139.2, 138.9, 137.0, 136.7, 136.4, 134.9, 134.8, 133.0, 132.2, 132.2, 131.7 (q, *J* = 34.0 Hz), 131.4, 131.1, 131.1, 130.6, 130.5, 130.0 (2C), 129.9, 129.8 (q, *J* = 3.8 Hz), 129.0, 128.9 (2C), 128.6, 127.6, 126.6, 126.5, 124.5 (q, *J* = 3.8 Hz), 123.2 (q, *J* = 273.4 Hz), 122.4, 115.6, 56.7, 54.6, 53.3, 52.7, 47.8, 42.2, 39.4, 36.2, 14.5, 13.2, 11.9. **MS (ESI)**: m/z 964.4 [M - H]^-^. **HRMS (ESI)**: m/z [M + H]^+^ calcd for C_48_H_43_ClF_3_N_9_O_4_S_2_, 966.2593; found, 966.2593.

**(*S*)-*N*-(2-(2-(2-(4-(4-chlorophenyl)-2,3,9-trimethyl-6*H*-thieno[3,2-*f*][1,2,4]triazolo[**

**4,3-*a*][1,4]diazepin-6-yl)acetamido)ethoxy)ethyl)-3-(8-((3-(trifluoromethyl)phenyl)sulfonamido)quinolin-5-yl)benzamide (2-7)**

The compound **2-7** (17.8 mg, 34% yield) was synthesized from **SL1** and **S5e** according to the same procedures as the **2-1** preparation. **^1^H NMR** (500 MHz, CDCl_3_) δ 8.66 (dd, *J* = 4.0, 1.5 Hz, 1H), 8.16 (s, 1H), 8.11 (dd, *J* = 8.5, 1.5 Hz, 1H), 8.04 (d, *J* = 8.0 Hz, 1H), 7.99 – 7.94 (m, 3H), 7.78 (d, *J* = 8.0 Hz, 1H), 7.68 (d, *J* = 8.0 Hz, 1H), 7.51 – 7.48 (m, 1H), 7.47 – 7.44 (m, 1H), 7.43 – 7.39 (m, 3H), 7.34 – 7.27 (m, 5H), 4.49 (dd, *J* = 9.5, 4.5 Hz, 1H), 3.80 – 3.76 (m, 1H), 3.73 – 3.69 (m, 1H), 3.66 – 3.62 (m, 1H), 3.58 – 3.52 (m, 3H), 3.51 – 3.37 (m, 3H), 3.17 (dd, *J* = 14.0, 4.5 Hz, 1H), 2.54 (s, 3H), 2.41 (s, 3H), 1.66 (s, 3H). **^13^C NMR** (126 MHz, CDCl_3_) δ 170.8, 167.8, 164.2, 155.8, 150.0, 148.9, 140.6, 138.9, 138.8, 137.2, 136.5, 135.3, 135.2, 134.9, 132.7, 132.7, 132.0, 131.7 (q, *J* = 32.8 Hz), 131.4, 131.1, 130.7, 130.5, 130.0 (2C), 129.9, 129.8 (q, *J* = 3.8 Hz), 129.3, 128.9 (2C), 128.8, 127.5, 126.8, 126.6, 124.5 (q, *J* = 3.8 Hz), 123.2 (q, *J* = 272.2 Hz), 122.3, 115.6, 70.0, 69.4, 54.8, 40.4, 39.8, 39.5, 14.5, 13.3, 11.8. **MS (ESI)**: m/z 941.2 [M + H]^+^. **HRMS (ESI)**: m/z [M + H]^+^ calcd for C_46_H_40_ClF_3_N_8_O_5_S_2_, 941.2277; found, 941.2278.

**(*S*)-*N*-(2-(2-(2-(2-(4-(4-chlorophenyl)-2,3,9-trimethyl-6*H*-thieno[3,2-*f*][1,2,4]**

**triazolo[4,3-*a*][1,4]diazepin-6-yl)acetamido)ethoxy)ethoxy)ethyl)-3-(8-((3-(trifluoromethyl)phenyl)sulfonamido)quinolin-5-yl)benzamide (2-8)**

The compound **2-8** (32.5 mg, 60% yield) was synthesized from **SL1** and **S5f** according to the same procedures as the **2-1** preparation. **^1^H NMR** (500 MHz, CDCl_3_) δ 8.73 (dd, *J* = 4.0, 1.5 Hz, 1H), 8.19 (s, 1H), 8.12 (dd, *J* = 8.5, 1.5 Hz, 1H), 8.07 (d, *J* = 8.0 Hz, 1H), 7.93 – 7.92 (m, 1H), 7.87 (dt, *J* = 8.0, 1.5 Hz, 1H), 7.84 (d, *J* = 8.0 Hz, 1H), 7.77 – 7.75 (m, 1H), 7.69 (d, *J* = 8.0 Hz, 1H), 7.53 – 7.50 (m, 1H), 7.47 – 7.44 (m, 1H), 7.40 – 7.38 (m, 3H), 7.35 (dd, *J* = 8.5, 4.0 Hz, 1H), 7.32 – 7.29 (m, 3H), 7.26 – 7.25 (m, 1H), 4.63 (dd, *J* = 8.5, 6.0 Hz, 1H), 3.75 – 3.62 (m, 8H), 3.59 – 3.51 (m, 3H), 3.49 – 3.37 (m, 2H), 3.27 (dd, *J* = 14.0, 5.5 Hz, 1H), 2.55 (s, 3H), 2.40 (s, 3H), 1.67 (s, 3H). **^13^C NMR** (126 MHz, CDCl_3_) δ 170.6, 167.6, 164.2, 155.7, 150.0, 148.9, 140.6, 139.0, 138.8, 137.0, 136.6, 135.3, 135.2, 134.9, 132.8, 132.8, 132.1, 131.7 (q, *J* = 32.8 Hz), 131.2, 131.1, 130.7, 130.5, 130.0 (2C), 129.9, 129.8 (q, *J* = 2.5 Hz), 129.2, 128.9 (2C), 128.8, 127.6, 126.7, 126.5, 124.5 (q, *J* = 3.8 Hz), 123.2 (q, *J* = 273.4 Hz), 122.4, 115.6, 70.5, 70.4, 70.3, 70.0, 54.7, 40.1, 39.5, 39.4, 14.6, 13.3, 11.8. **MS (ESI)**: m/z 985.2 [M + H]^+^. **HRMS (ESI)**: m/z [M + H]^+^ calcd for C_48_H_44_ClF_3_N_8_O_6_S_2_, 985.2539; found, 985.2537.

**(*S*)-*N*-(1-(4-(4-chlorophenyl)-2,3,9-trimethyl-6*H*-thieno[3,2-*f*][1,2,4]triazolo[4,3-*a*][1,4]diazepin-6-yl)-2-oxo-6,9,12-trioxa-3-azatetradecan-14-yl)-3-(8-((3-(trifluoromethyl)phenyl)sulfonamido)quinolin-5-yl)benzamide (2-9)**

The compound **2-9** (21.3 mg, 38% yield) was synthesized from **SL1** and **S5g** according to the same procedures as the **2-1** preparation. **^1^H NMR** (500 MHz, CDCl_3_) δ 8.74 (d, *J* = 4.5 Hz, 1H), 8.20 (s, 1H), 8.11 (d, *J* = 8.5 Hz, 1H), 8.08 (d, *J* = 8.0 Hz, 1H), 7.93 (s, 1H), 7.89 (d, *J* = 8.0 Hz, 1H), 7.86 (d, *J* = 8.0 Hz, 1H), 7.75 (s, 1H), 7.69 (d, *J* = 8.0 Hz, 1H), 7.53 – 7.50 (m, 1H), 7.48 – 7.45 (m, 1H), 7.43 – 7.35 (m, 5H), 7.29 (d, *J* = 8.0 Hz, 2H), 7.26 – 7.25 (m, 1H), 4.65 (t, *J* = 7.0 Hz, 1H), 3.75 – 3.66 (m, 7H), 3.64 – 3.59 (m, 5H), 3.56 – 3.49 (m, 3H), 3.40 – 3.30 (m, 3H), 2.50 (s, 3H), 2.40 (s, 3H), 1.66 (s, 3H). **^13^C NMR** (126 MHz, CDCl_3_) δ 170.6, 167.6, 164.1, 155.8, 150.0, 149.0, 140.7, 139.0, 138.8, 137.0, 136.7, 135.3, 135.2, 135.0, 132.8, 132.8, 132.2, 131.8 (q, *J* = 34.0 Hz), 131.2, 131.1, 130.7, 130.5, 130.0 (2C), 129.9, 129.8 (q, *J* = 2.5 Hz), 129.2, 128.9 (2C), 128.8, 127.6, 126.7, 126.5, 124.6 (q, *J* = 3.8 Hz), 123.2 (q, *J* = 273.4 Hz), 122.4, 115.6, 70.6, 70.6, 70.3 (2C), 70.0, 69.9, 54.6, 40.4, 39.5, 39.1, 14.6, 13.3, 11.8. **MS (ESI)**: m/z 1029.2 [M + H]^+^. **HRMS (ESI)**: m/z [M + H]^+^ calcd for C_50_H_48_ClF_3_N_8_O_7_S_2_, 1029.2801; found, 1029.2799.

***N*-(1-((*S*)-4-(4-chlorophenyl)-2,3,9-trimethyl-6*H*-thieno[3,2-*f*][1,2,4]triazolo[4,3-*a*][1,4]diazepin-6-yl)-2-oxo-6,9,12,15-tetraoxa-3-azaheptadecan-17-yl)-3-(8-((3-(trifluoromethyl)phenyl)sulfonamido)quinolin-5-yl)cyclohexa-1,5-diene-1-carboxamide (2-10)**

The compound **2-10** (23.9 mg, 41% yield) was synthesized from **SL1** and **S5h** according to the same procedures as the **2-1** preparation. **^1^H NMR** (500 MHz, CDCl_3_) δ 8.78 (d, *J* = 4.0 Hz, 1H), 8.28 (d, *J* = 8.0 Hz, 1H), 8.15 (s, 1H), 8.10 (d, *J* = 8.0 Hz, 1H), 7.96 – 7.94 (m, 2H), 7.89 (d, *J* = 8.0 Hz, 1H), 7.84 (br.s, 1H), 7.78 (br.s,1H), 7.69 (d, *J* = 7.5 Hz, 1H), 7.55 – 7.42 (m, 7H), 7.31 (d, *J* = 7.5 Hz, 2H), 4.78 (t, *J* = 7.0 Hz, 1H), 3.70 – 3.60 (m, 15H), 3.56 – 3.50 (m, 5H), 3.44 – 3.41 (m, 2H), 2.72 (s, 3H), 2.42 (s, 3H), 1.67 (s, 3H). **^13^C NMR** (126 MHz, CDCl_3_) δ 170.1, 167.4, 164.9, 155.3, 150.3, 148.3, 140.6, 138.6, 138.0, 137.8, 136.3, 135.6, 135.4, 132.7, 132.5, 132.2, 131.7, 131.6 (q, *J* = 32.8 Hz), 131.6, 131.0, 130.6, 130.5, 130.5, 130.0 (2C), 129.8 (q, *J* = 2.5 Hz), 129.2, 129.0 (2C), 128.9, 128.0, 126.9, 126.9, 124.5 (q, *J* = 3.8 Hz), 123.2 (q, *J* = 273.4 Hz), 122.4, 117.2, 70.6, 70.6, 70.6, 70.5, 70.3, 70.2, 70.0, 70.0, 53.9, 39.8, 39.5, 37.8, 14.6, 13.4, 11.7. **MS (ESI)**: m/z 1073.3 [M + H]^+^. **HRMS (ESI)**: m/z [M + H]^+^ calcd for C_52_H_52_ClF_3_N_8_O_8_S_2_, 1073.3063; found, 1073.3063.

***N*-(1-((*S*)-4-(4-chlorophenyl)-2,3,9-trimethyl-6*H*-thieno[3,2-*f*][1,2,4]triazolo[4,3-*a*][1,4]diazepin-6-yl)-2-oxo-6,9,12,15,18-pentaoxa-3-azaicosan-20-yl)-3-(8-((3-(trifluoromethyl)phenyl)sulfonamido)quinolin-5-yl)cyclohexa-1,5-diene-1-carboxamide (2-11)**

The compound **2-11** (30.7 mg, 50% yield) was synthesized from **SL1** and **S5i** according to the same procedures as the **2-1** preparation. **^1^H NMR** (500 MHz, CDCl_3_) δ 8.74 (dd, *J* = 4.0, 1.5 Hz, 1H), 8.19 (s, 1H), 8.14 (dd, *J* = 8.5, 2.0 Hz, 1H), 8.08 (d, *J* = 8.0 Hz, 1H), 7.91 – 7.89 (m, 2H), 7.87 (d, *J* = 7.5 Hz, 1H), 7.69 (d, *J* = 8.0 Hz, 1H), 7.57 (br.s, 1H), 7.53 – 7.48 (m, 2H), 7.45 (m, *J* = 7.5, 2.0 Hz, 1H), 7.43 (d, *J* = 8.0 Hz, 1H), 7.39 – 7.34 (m, 4H), 7.30 (d, *J* = 8.5 Hz, 2H), 4.65 (t, *J* = 7.0 Hz, 1H), 3.68 – 3.53 (m, 22H), 3.52 – 3.44 (m, 3H), 3.39 (dd, *J* = 14.5, 7.5 Hz, 1H), 2.59 (s, 3H), 2.39 (s, 3H), 1.65 (s, 3H). **^13^C NMR** (126 MHz, CDCl_3_) δ 170.8, 167.4, 163.9, 155.9, 149.9, 149.0, 140.6, 139.0, 138.8, 136.8, 136.8, 135.3, 135.1, 134.9, 132.8, 132.8, 132.2, 131.7 (q, *J* = 34.0 Hz), 131.1, 131.0, 130.6, 130.5, 130.0 (2C), 129.9, 129.8 (q, *J* = 2.5 Hz), 129.2, 128.8 (2C), 128.8, 127.6, 126.7, 126.6, 124.5 (q, *J* = 3.8 Hz), 123.2 (q, *J* = 273.4 Hz), 122.4, 115.6, 70.6 (2C), 70.6, 70.6 (2C), 70.5, 70.3, 70.2, 70.0 (2C) , 54.5, 40.0, 39.5, 39.0, 14.6, 13.2, 11.9. **MS (ESI)**: m/z 1117.3 [M + H]^+^. **HRMS (ESI)**: m/z [M + H]^+^ calcd for C_54_H_56_ClF_3_N_8_O_9_S_2_, 1117.3326; found, 1117.3318.

**(*S*)-*N*-(8-(4-(2-(2-(4-(4-chlorophenyl)-2,3,9-trimethyl-6*H*-thieno[3,2-*f*][1,2,4]**

**triazolo[4,3-*a*][1,4]diazepin-6-yl)acetamido)ethyl)piperazin-1-yl)-8-oxooctyl)-3-(8-((3-(trifluoromethyl)phenyl)sulfonamido)quinolin-5-yl)benzamide (2-12)**

The compound **2-12** (10.2 mg, 17% yield) was synthesized from **SL1** and **S7c** according to the same standard procedures of the **2-1** preparation. **^1^H NMR** (500 MHz,CDCl_3_) δ 8.75 (dd, *J* = 4.0, 1.5 Hz, 1H), 8.17 (s, 1H), 8.12 (d, *J* = 8.5 Hz, 1H), 8.08 (d, *J* = 8.0 Hz, 1H), 7.87 (d, *J* = 8.0 Hz, 1H), 7.81 – 7.79 (m, 2H), 7.69 (d, *J* = 8.0 Hz, 1H), 7.53 – 7.50 (m, 2H), 7.47 (d, *J* = 8.0 Hz, 1H), 7.42 – 7.36 (m, 4H), 7.30 (d, *J* = 8.5 Hz, 2H), 6.94 (br.s, 1H) , 6.44 (br.s, 1H), 4.62 (t, *J* = 7.0 Hz, 1H), 3.62 – 3.49 (m, 3H), 3.44 – 3.32 (m, 7H), 2.64 (s, 3H), 2.51 – 2.44 (m, 4H), 2.39 (s, 3H), 2.38 – 2.33 (m, 2H), 2.27 (t, *J* = 8.0 Hz, 2H), 1.66 (s, 3H), 1.62 – 1.57 (m, 4H), 1.38 – 1.32 (m, 6H). **^13^C NMR** (126 MHz, CDCl_3_) δ 171.6, 170.6, 167.3, 164.1, 155.8, 150.0, 149.0, 140.6, 139.2, 138.8, 137.0, 136.7, 135.4, 135.0, 134.8, 132.9, 132.8, 132.2, 131.6 (q, *J* = 32.8 Hz), 131.1, 131.1, 130.6, 130.5, 130.0 (2C), 129.9, 129.7 (q, *J* = 3.8 Hz), 128.9, 128.9 (2C), 128.7, 127.6, 126.7, 126.2, 124.5 (q, *J* = 3.8 Hz), 123.2 (q, *J* = 273.4 Hz), 122.4, 115.6, 56.7, 54.5, 53.2, 52.8, 45.6, 41.5, 40.3, 39.4, 36.3, 33.2, 29.6, 29.4, 29.1, 26.8, 25.2, 14.5, 13.2, 11.9. **MS (ESI)**: m/z 1107.3 [M + H]^+^. **HRMS (ESI)**: m/z [M + H]^+^ calcd for C_56_H_58_ClF_3_N_10_O_5_S_2_, 1107.3747; found, 1107.3749.

**(*S*)-*N*-(8-((2-(2-(4-(4-chlorophenyl)-2,3,9-trimethyl-6*H*-thieno[3,2-*f*][1,2,4]triazolo**

**[4,3-*a*][1,4]diazepin-6-yl)acetamido)ethyl)amino)-8-oxooctyl)-3-(8-((3-(trifluoro**

**methyl)phenyl)sulfonamido)quinolin-5-yl)benzamide (2-13)**

The compound **2-13** (22.9 mg, 40% yield) was synthesized from **SL1** and **S7a** according to the same procedures as the **2-1** preparation. **^1^H NMR** (500 MHz, CDCl_3_) δ 8.75 (dd, *J* = 4.5, 1.5 Hz, 1H), 8.18 (s, 1H), 8.12 (dd, *J* = 8.5, 1.5 Hz, 1H), 8.08 (d, *J* = 7.5 Hz, 1H), 7.87 (d, *J* = 8.0 Hz, 1H), 7.83 – 7.81 (m, 2H), 7.69 (d, *J* = 7.5 Hz, 1H), 7.53 – 7.49 (m, 2H), 7.46 (d, *J* = 7.5 Hz, 1H), 7.41 (d, *J* = 8.0 Hz, 1H), 7.39 – 7.33 (m, 4H), 7.30 (d, *J* = 8.0 Hz, 2H), 6.78 (br.s, 1H), 6.66 (br.s, 1H), 4.62 (dd, *J* = 8.0, 6.0 Hz, 1H), 3.52 – 3.45 (m, 2H), 3.43 – 3.36 (m, 3H), 3.35 – 3.26 (m, 3H), 2.63 (s, 3H), 2.40 (s, 3H), 2.13 (t, *J* = 7.5 Hz, 2H), 1.66 (s, 3H), 1.59 – 1.53 (m, 4H), 1.34 – 1.28 (m, 6H). **^13^C NMR** (126 MHz, CDCl_3_) δ 174.0, 171.4, 167.4, 164.4, 155.9, 150.1, 149.0, 140.6, 139.1, 138.8, 137.1, 136.6, 135.5, 135.0, 134.8, 132.9, 132.8, 132.2, 131.7 (q, *J* = 34.0 Hz), 131.3, 131.1, 130.6, 130.5, 130.0 (2C), 129.9, 129.8 (q, *J* = 2.5 Hz), 128.9 (2C), 128.9, 128.8, 127.6, 126.7, 126.4, 124.5 (q, *J* = 3.8 Hz), 123.3 (q, *J* = 273.4 Hz), 122.4, 115.6, 54.5, 40.2, 39.7, 39.7, 39.2, 36.5, 29.5, 29.0, 28.9, 26.8, 25.5, 14.6, 13.3, 12.0. **MS (ESI)**: m/z 1038.3 [M + H]^+^. **HRMS (ESI)**: m/z [M + H]^+^ calcd for C_52_H_51_ClF_3_N_9_O_5_S_2_, 1038.3168; found, 1038.3171.

**4-(((1*r*,3*r*)-3-(3-chloro-4-cyanophenoxy)-2,2,4,4-tetramethylcyclobutyl)carbamoyl) benzoic acid (AL)**

To the solution of 1,1-Dimethylethyl N-(trans-3-hydroxy-2,2,4,4-tetrame thylcyclobutyl)carbamate (2.43 g, 10.0 mmol, 1.00 equiv.) in dry DMF (20 mL) was added NaH (60% dispersion in mineral oil, 600 mg, 15.0 mmol, 1.50 equiv.) at 0 °C under argon atmosphere. After stirring the mixture at 0 °C for 20 min, 2-Chloro-4-fluorobenzonitrile (1.86 g, 12.0 mmol, 1.20 equiv.) was added. The mixture was stirred at rt for 4 h. After the complete conversion of starting materials, water (40 mL) was added, and the mixture was extracted with EtOAc (20 × 3 mL), the combined organic layers were washed with brine, and then dried over anhydrous Na_2_SO_4_. The solvent was removed using a rotary evaporator and purified by flash column chromatography on silica gel to get **S8** (2.87 g, 76% yield). Then the desired intermediate **S9** was obtained by deprotection with TFA (5 mL) in DCM (5 mL) at 0 ℃ for 3 h.

To a solution of compound **S9** (2.08 g, 7.50 mmol, 1.00 equiv.) in DMF (15 mL) was added DIPEA (4 mL, 22.5 mmol, 3.00 equiv.) and HATU (4.27 g, 11.3 mmol, 1.50 equiv.) at 0 ℃ and stirred for 30 min. Then the 1-(1,1-Dimethylethyl) 1,4-benzenedicarboxylate (2.50 g, 8.25 mmol, 1.10 equiv.) was added and reacted under anhydrous conditions for 2 h at rt. The mixture was extracted with ethyl acetate and saturated brine (3 × 10 mL). The combined ethyl acetate layers were dried over anhydrous Na2SO4 and then evaporated under vacuum. The residue was purified by flash column chromatography on silica gel to obtain the product **S10** (2.70 g, 75% yield). The target material **AL** (2.40 g) was obtained by deprotection with TFA (4 mL) in DCM (8 mL) for 3 h. **^1^H NMR** (500 MHz, DMSO-*d*_6_) δ 8.04 – 8.02 (dd, 3H), 7.92 (d, *J* = 8.0 Hz, 2H), 7.88 (d, *J* = 8.5 Hz, 1H), 7.19 (d, *J* = 2.0 Hz, 1H), 7.00 (dd, *J* = 9.0, 2.5 Hz, 1H), 4.32 (s, 1H), 4.08 (d, *J* = 9.0 Hz, 1H), 1.23 (s, 6H), 1.14 (s, 6H). **^13^C NMR** (126 MHz, DMSO-*d*6) δ 166.9, 166.8, 162.6, 138.6, 136.9, 136.1, 133.0, 129.2 (2C), 127.9 (2C), 116.9, 116.3, 114.7, 103.7, 83.9, 58.5, 40.3 (2C), 24.0 (2C), 23.2 (2C). **MS (ESI)**: m/z 427.1 [M + H]^+^. **HRMS (ESI)**: m/z [M + H]^+^ calcd for C_23_H_23_ClN_2_O_4_, 427.1420; found, 427.1418.

***tert*-butyl(2-(4-(((1*r*,3*r*)-3-(3-chloro-4-cyanophenoxy)-2,2,4,4-tetramethylcyclobutyl)carbamoyl)benzamido)ethyl)carbamate (S11a)**

To the solution of **AL** (42.6 mg, 0.10 mmol, 1.00 equiv.), *tert*-butyl (2-aminoethyl)carbamate (17.6 mg, 0.11 mmol, 1.10 equiv.), EDCI (28.8 mg, 0.15 mmol, 1.50 equiv.), HOBT (27.0 mg, 0.2 mmol, 2.00 equiv.) in anhydrous DMF (1 mL) was added DIPEA (52 µL, 0.3 mmol, 3.00 equiv.). After being stirred for 12 h at rt, the reaction solution was extracted with ethyl acetate, washed with water, and saturated brine solution. The combined organic layers were dried with anhydrous Na_2_SO_4_, then the solvent was evaporated. The residue was purified by flash column chromatography on silica gel (DCM/MeOH, 40:1 to 20:1) to obtain the product **S11a** (56.2 mg, 98% yield). **^1^H NMR** (500 MHz, CDCl_3_) δ 7.91 (d, *J* = 8.5 Hz, 2H), 7.80 (d, *J* = 8.5 Hz, 2H), 7.57 (d, *J* = 8.5 Hz, 1H), 7.53 (br.s, 1H), 6.97 (d, *J* = 2.5 Hz, 1H), 6.81 (dd, *J* = 8.5, 2.5 Hz, 1H), 6.27 (d, *J* = 8.0 Hz, 1H), 5.06 (br.s, 1H), 4.16 (d, *J* = 8.0 Hz, 1H), 4.07 (s, 1H), 3.58 – 3.55 (m, 2H), 3.44 – 3.41 (m, 2H), 1.43 (s, 9H), 1.28 (s, 6H), 1.23 (s, 6H). **MS (ESI)**: m/z 567.1 [M - H]^-^.

***tert*-butyl(4-(4-(((1*r*,3*r*)-3-(3-chloro-4-cyanophenoxy)-2,2,4,4-tetramethylcyclobutyl)carbamoyl)benzamido)butyl)carbamate (S11b)**

The compound **S11b** (55.0 mg, 92% yield) was synthesized from **AL** and *tert*-butyl (4-aminobutyl)carbamate according to the same procedures as the **S11a** preparation. **^1^H NMR** (500 MHz, CDCl_3_) δ 7.86 (d, *J* = 8.0 Hz, 2H), 7.76 (d, *J* = 8.5 Hz, 2H), 7.55 (d, *J* = 8.8 Hz, 1H), 7.04 (br.s, 1H), 6.96 (d, *J* = 2.5 Hz, 1H), 6.80 (dd, *J* = 9.0, 2.5 Hz, 1H), 6.41 (d, *J* = 8.5 Hz, 1H), 4.76 (br.s, c1H), 4.15 (d, *J* = 8.5 Hz, 1H), 4.07 (s, 1H), 3.49 – 3.45 (m, 2H), 3.16 – 3.12 (m, 2H), 1.67 – 1.61 (m, 2H), 1.59 – 1.54 (m, 2H), 1.42 (s, 9H), 1.28 (s, 6H), 1.22 (s, 6H). **MS (ESI)**: m/z 619.2 [M + Na]^+^.

***tert*-butyl(6-(4-(((1*r*,3*r*)-3-(3-chloro-4-cyanophenoxy)-2,2,4,4-tetramethylcyclobutyl)carbamoyl)benzamido)hexyl)carbamate (S11c)**

The compound **S11c** (54.7 mg, 87% yield) was synthesized from **AL** and *tert*-butyl (6-aminohexyl)carbamate according to the same procedures as the **S11a** preparation. **^1^H NMR** (500 MHz, CDCl_3_) δ 7.86 (d, *J* = 8.0 Hz, 2H), 7.78 (d, *J* = 8.5 Hz, 2H), 7.55 (d, *J* = 9.0 Hz, 1H), 6.96 (d, *J* = 2.5 Hz, 1H), 6.80 (dd, *J* = 8.5, 2.0 Hz, 1H), 6.38 (d, *J* = 8.0 Hz, 1H), 4.62 (br.s, 1H), 4.15 (d, *J* = 8.0 Hz, 1H), 4.07 (s, 1H), 3.45 – 3.41 (m, 2H), 3.12 – 3.08 (m, 2H), 1.64 – 1.59 (m, 2H), 1.50 – 1.45 (m, 2H), 1.41 (s, 9H), 1.38 – 1.32 (m, 4H), 1.28 (s, 6H), 1.22 (s, 6H). **MS (ESI)**: m/z 647.2 [M + Na]^+^.

***tert*-butyl(8-(4-(((1*r*,3*r*)-3-(3-chloro-4-cyanophenoxy)-2,2,4,4-tetramethylcyclobutyl)carbamoyl)benzamido)octyl)carbamate (S11d)**

The compound **S11d** (56.0 mg, 86% yield) was synthesized from **AL** and *tert*-butyl (8-aminooctyl)carbamate according to the same procedures as the **S11a** preparation. **^1^H NMR** (500 MHz, CDCl_3_) δ 7.79 (d, *J* = 8.5 Hz, 2H), 7.74 (d, *J* = 8.0 Hz, 2H), 7.54 (d, *J* = 8.5 Hz, 1H), 6.94 (d, *J* = 2.5 Hz, 1H), 6.79 (dd, *J* = 9.0, 2.5 Hz, 1H), 6.74 (br.s, 1H), 6.48 (d, *J* = 8.5 Hz, 1H), 4.61 (br.s, 1H), 4.14 (d, *J* = 8.0 Hz, 1H), 4.07 (s, 1H), 3.42 – 3.38 (m, 2H), 3.06 – 3.02 (m, 2H), 1.61 – 1.56 (m, 2H), 1.45 – 1.42 (m, 2H), 1.40 (s, 9H), 1.36 – 1.30 (m, 6H), 1.27 (s, 6H), 1.26 – 1.24 (m, 2H), 1.20 (s, 6H). **MS (ESI)**: m/z 675.3 [M + Na]^+^.

***tert*-butyl(10-(4-(((1*r*,3*r*)-3-(3-chloro-4-cyanophenoxy)-2,2,4,4-tetramethylcyclobutyl)carbamoyl)benzamido)decyl)carbamate (S11e)**

The compound **S11e** (58.1 mg, 85% yield) was synthesized from **AL** and *tert*-butyl (10-aminodecyl)carbamate according to the same procedures as the **S11a** preparation. **^1^H NMR** (500 MHz, CDCl_3_) δ 7.78 (d, *J* = 8.5 Hz, 2H), 7.74 (d, *J* = 8.5 Hz, 2H), 7.54 (d, *J* =9.0 Hz, 1H), 6.94 (d, *J* = 2.5 Hz, 1H), 6.79 (dd, *J* = 8.5, 2.5 Hz, 1H), 6.68 (t, *J* = 6.0 Hz, 1H), 6.46 (d, *J* = 8.5 Hz, 1H), 4.59 (br.s, 1H), 4.14 (d, *J* = 8.5 Hz, 1H), 4.07 (s, 1H), 3.43 – 3.39 (m, 2H), 3.07 – 3.03 (m, 2H), 1.62 – 1.56 (m, 2H), 1.44 – 1.42 (m, 2H), 1.40 (s, 9H), 1.35 – 1.31 (m, 4H), 1.27 (s, 6H), 1.25 – 1.22 (m, 8H), 1.20 (s, 6H). **MS (ESI)**: m/z 703.3 [M + Na]^+^.

***tert*-butyl(2-(2-(4-(((1*r*,3*r*)-3-(3-chloro-4-cyanophenoxy)-2,2,4,4-tetramethylcyclobutyl)carbamoyl)benzamido)ethoxy)ethyl)carbamate (S11f)**

The compound **S11f** (55.8 mg, 91% yield) was synthesized from **AL** and *tert*-butyl (2-(2-aminoethoxy)ethyl)carbamate according to the same procedures as the **S11a** preparation. **^1^H NMR** (500 MHz, CDCl_3_) δ 7.88 (d, *J* = 8.0 Hz, 2H), 7.80 (d, *J* = 8.0 Hz, 2H), 7.56 (d, *J* = 8.5 Hz, 1H), 6.96 (d, *J* = 2.0 Hz, 1H), 6.88 (br.s, 1H), 6.80 (dd, *J* = 8.5, 2.5 Hz, 1H), 6.31 (d, *J* = 8.0 Hz, 1H), 4.88 (br.s, 1H), 4.16 (d, *J* = 8.0 Hz, 1H), 4.07 (s, 1H), 3.67 – 3.64 (m, 4H), 3.56 – 3.54 (m, 2H), 3.34 – 3.30 (m, 2H), 1.41 (s, 9H), 1.28 (s, 6H), 1.23 (s, 6H). **MS (ESI)**: m/z 635.2 [M + Na]^+^.

***tert*-butyl(2-(2-(2-(4-(((1*r*,3*r*)-3-(3-chloro-4-cyanophenoxy)-2,2,4,4-tetramethylcyclobutyl)carbamoyl)benzamido)ethoxy)ethoxy)ethyl)carbamate (S11g)**

The compound **S11g** (46.3 mg, 70% yield) was synthesized from **AL** and *tert*-butyl (2-(2-(2-aminoethoxy)ethoxy)ethyl)carbamate according to the same standard procedures as the **S11a** preparation. **^1^H NMR** (500 MHz, CDCl_3_) δ 7.88 (d, *J* = 7.5 Hz, 2H), 7.82 (d, *J* = 8.0 Hz, 2H), 7.56 (d, *J* = 9.0 Hz, 1H), 6.96 (d, *J* = 2.5 Hz, 1H), 6.92 (br.s, 1H), 6.81 (dd, *J* = 8.5, 2.5 Hz, 1H), 6.33 (d, *J* = 8.0 Hz, 1H), 4.98 (br.s, 1H), 4.16 (d, *J* = 8.0 Hz, 1H), 4.07 (s, 1H), 3.69 – 3.64 (m, 8H), 3.55 – 3.53 (m, 2H), 3.29 – 3.25 (m, 2H), 1.42 (s, 9H), 1.28 (s, 6H), 1.23 (s, 6H). **MS (ESI)**: m/z 679.2 [M + Na]^+^.

***tert*-butyl(1-(4-(((1*r*,3*r*)-3-(3-chloro-4-cyanophenoxy)-2,2,4,4-tetramethylcyclobutyl)carbamoyl)phenyl)-1-oxo-5,8,11-trioxa-2-azatridecan-13-yl)carbamate (S11h)**

The compound **S11h** (62.8 mg, 90% yield) was synthesized from **AL** and *tert*-butyl (2-(2-(2-(2-aminoethoxy)ethoxy)ethoxy)ethyl)carbamate according to the same procedures as the **S11a** preparation. **^1^H NMR** (500 MHz, CDCl_3_) δ 7.88 (d, *J* = 7.5 Hz, 2H), 7.80 (d, *J* = 8.0 Hz, 2H), 7.55 (d, *J* = 9.0 Hz, 1H), 6.95 (d, *J* = 2.5 Hz, 1H), 6.80 (dd, *J* = 8.5, 2.0 Hz, 1H), 6.44 (d, *J* = 8.5 Hz, 1H), 5.13 (br.s, 1H), 4.16 (d, *J* = 6.5 Hz, 1H), 4.06 (s, 1H), 3.68 – 3.63 (m, 8H), 3.62 – 3.60 (m, 2H), 3.58 – 3.57 (m, 2H), 3.48 – 3.46 (m, 2H), 3.24 – 3.21 (m, 2H), 1.39 (s, 9H), 1.28 (s, 6H), 1.22 (s, 6H). **MS (ESI)**: m/z 723.2 [M + Na]^+^.

***tert*-butyl(1-(4-(((1*r*,3*r*)-3-(3-chloro-4-cyanophenoxy)-2,2,4,4-tetramethylcyclobutyl)carbamoyl)phenyl)-1-oxo-5,8,11,14-tetraoxa-2-azahexadecan-16-yl)carbamate (S11i)**

The compound **S11i** (60.9 mg, 82% yield) was synthesized from **AL** and *tert*-butyl (14-amino-3,6,9,12-tetraoxatetradecyl)carbamate according to the same procedures as the **S11a** preparation **^1^H NMR** (500 MHz, CDCl_3_) δ 7.94 (d, *J* = 8.0 Hz, 2H), 7.80 (d, *J* = 8.5 Hz, 2H), 7.56 (d, *J* = 9.0 Hz, 1H), 7.51 (br.s, 1H), 6.96 (d, *J* = 2.5 Hz, 1H), 6.80 (dd, *J* = 8.5, 2.5 Hz, 1H), 6.38 (d, *J* = 8.5 Hz, 1H), 4.17 (d, *J* = 8.5 Hz, 1H), 4.07 (s, 1H), 3.68 – 3.62 (m, 12H), 3.60 – 3.57 (m, 4H), 3.49 – 3.47 (m, 2H), 3.24 – 3.20 (m, 2H), 1.40 (s, 9H), 1.28 (s, 6H), 1.23 (s, 6H). **MS (ESI)**: m/z 767.2 [M + Na]^+^.

***tert*-butyl(1-(4-(((1*r*,3*r*)-3-(3-chloro-4-cyanophenoxy)-2,2,4,4-tetramethylcyclobutyl)carbamoyl)phenyl)-1-oxo-5,8,11,14,17-pentaoxa-2-azanonadecan-19-yl)carbamate (S11j)**

The compound **S11j** (73.5 mg, 93% yield) was synthesized from **AL** and *tert*-butyl (17-amino-3,6,9,12,15-pentaoxaheptadecyl)carbamate according to the same standard procedures as the **S11a** preparation. **^1^H NMR** (500 MHz, CDCl_3_) δ 7.93 (d, *J* = 8.0 Hz, 2H), 7.79 (d, *J* = 8.5 Hz, 2H), 7.55 (d, *J* = 9.0 Hz, 1H), 6.95 (d, *J* = 2.5 Hz, 1H), 6.79 (dd, *J* = 9.0, 2.5 Hz, 1H), 6.39 (d, *J* = 8.5 Hz, 1H), 5.22 (br.s, 1H), 4.15 (d, *J* = 8.5 Hz, 1H), 4.06 (s, 1H), 3.67 – 3.60 (m, 16H), 3.58 – 3.56 (m, 2H), 3.54 – 3.52 (m, 2H), 3.45 – 3.43 (m, 2H), 3.24 – 3.21 (m, 2H), 1.40 (s, 9H), 1.27 (s, 6H), 1.21 (s, 6H). **MS (ESI)**: m/z 811.2 [M + Na]^+^.

***N*^1^-((1*r*,3*r*)-3-(3-chloro-4-cyanophenoxy)-2,2,4,4-tetramethylcyclobutyl)-*N*^4^-(4-(3-(8-((3-(trifluoromethyl)phenyl)sulfonamido)quinolin-5-yl)benzamido)butyl)**

**terephthalamide (3-1)**

To the solution of **S11b** (71.1 mg, 0.12 mmol) in re-distilled dichloromethane (2 mL). was added TFA (1 mL) slowly at 0 ℃. The mixture was stirred for 30 min in an ice bath. Then the mixture was diluted with DCM and evaporated to obtain the **S12b** salt form for the next step without further purification.

To a solution of **SL1** (47.2 mg, 0.10 mmol, 1.00 equiv.) **S12b** (59.5 mg, 0.12 mmol, 1.20 equiv.), EDCI (28.7 mg, 0.15 mmol, 1.50 equiv.), HOBT (27.0 mg, 0.20 mmol, 2.00 equiv.) in anhydrous DMF (2 mL) was added DIPEA (87 μL, 0.50 mmol, 5.00 equiv.). After being stirred overnight at rt, the reaction solution was extracted with ethyl acetate and washed with water and saturated brine solution. The combined organic layers were dried with anhydrous Na_2_SO_4,_ and the solvent was evaporated. The product was purified by Preparative HPLC (50% to 99% MeOH, 0.05% HCl in H_2_O) to afford Compound **3-1** (28.7 mg, 30% yield). **^1^H NMR** (500 MHz, CDCl_3_) δ 8.75 (dd, *J* = 4.0, 1.5 Hz, 1H), 8.15 (d, *J* = 9.0 Hz, 1H), 8.06 (d, *J* = 8.0 Hz, 1H), 7.93 – 7.90 (m, 2H), 7.86 – 7.82 (m, 5H), 7.69 (d, *J* = 8.0 Hz, 1H), 7.57 – 7.49 (m, 5H), 7.39 (dd, *J* = 8.5, 4.0 Hz, 1H), 7.14 (t, *J* = 5.5 Hz, 1H), 6.98 (d, *J* = 2.5 Hz, 1H), 6.88 (t, *J* = 5.5 Hz, 1H), 6.82 (dd, *J* = 8.5, 2.0 Hz, 1H), 6.45 (d, *J* = 8.5 Hz, 1H), 4.17 (d, *J* = 8.0 Hz, 1H), 4.12 (s, 1H), 3.57 – 3.52 (m, 4H), 1.74 – 1.66 (m, 4H), 1.29 (s, 6H), 1.24 (s, 6H). **^13^C NMR** (126 MHz, CDCl_3_) δ 167.6, 167.1, 167.0, 162.8, 149.0, 140.5, 139.1, 138.8, 138.5, 137.3, 137.2, 135.3, 135.3, 135.1, 134.8, 133.0, 132.9, 131.7 (q, *J* = 32.8 Hz), 130.5, 130.0, 129.8 (q, *J* = 2.5 Hz), 128.9, 128.7, 127.8, 127.5 (2C), 127.4 (2C), 126.8, 126.7, 124.5 (q, *J* = 3.8 Hz), 123.2 (q, *J* = 272.2 Hz), 122.5, 117.0, 116.5, 115.6, 114.3, 105.3, 84.8, 59.0, 40.6 (2C), 39.9, 39.8, 27.3, 26.6, 23.9 (2C), 23.7 (2C). **MS (ESI)**: m/z 951.3 [M + H]^+^.**HRMS (ESI)**: m/z [M + H]^+^ calcd for C_50_H_46_ClF_3_N_6_O_6_S, 951.2913; found, 951.2916.

**N^1^-((1r,3r)-3-(3-chloro-4-cyanophenoxy)-2,2,4,4-tetramethylcyclobutyl)-N^4^-(6-(3-(8-((5-(trifluoromethyl)cyclohexa-1,3-diene)-1-sulfonamido)quinolin-5-yl)**

**benzamido)hexyl)terephthalamide (3-2)**

The compound **3-2** (46.0 mg, 47% yield) was synthesized from **AL** and **S12c** according to the same procedures as the **3-1** preparation. **^1^H NMR** (500 MHz, CDCl_3_) δ 8.73 (dd, *J* = 4.0, 1.5 Hz, 1H), 8.14 (s, 1H), 8.11 (dd, *J* = 8.5, 1.5 Hz, 1H), 8.06 (d, *J* = 8.0 Hz, 1H), 7.85 – 7.79 (m, 5H), 7.76 (d, *J* = 8.0, 2H), 7.68 (d, *J* = 8.0 Hz, 1H), 7.55 – 7.46 (m, 4H), 7.39 (d, *J* = 8.0 Hz, 1H), 7.36 (dd, *J* = 8.5, 4.0 Hz, 1H), 6.96 (d, *J* = 2.5 Hz, 1H), 6.85 – 6.79 (m, 3H), 6.44 (d, *J* = 8.5 Hz, 1H), 4.14 (d, *J* = 8.5 Hz, 1H), 4.08 (s, 1H), 3.45 – 3.38 (m, 4H), 1.62 – 1.59 (m, 4H), 1.41 – 1.39 (m, 4H), 1.28 (s, 6H), 1.22 (s, 6H). **^13^C NMR** (126 MHz, CDCl_3_) δ 167.5, 167.1, 166.8, 162.7, 149.0, 140.5, 139.1, 138.8, 138.4, 137.6, 137.0, 135.3, 135.2, 135.0, 134.7, 132.9, 132.8, 131.6 (q, *J* = 34.0 Hz), 130.5, 130.0, 129.8 (q, *J* = 3.8 Hz), 129.0, 128.7, 127.6, 127.5 (2C), 127.3 (2C), 126.6, 126.5, 124.4 (q, *J* = 3.8 Hz,), 123.2 (q, *J* = 273.4 Hz), 122.4, 116.9, 116.5, 115.4, 114.3, 105.2, 84.8, 59.0, 40.5 (2C), 39.6 (2C), 29.5, 29.5, 25.9, 25.9, 23.8 (2C), 23.6 (2C). **MS (ESI)**: m/z 979.3 [M + H]^+^. **HRMS (ESI)**: m/z [M + H]^+^ calcd for C_52_H_50_ClF_3_N_6_O_6_S, 979.3226; found, 979.3226.

***N*^1^-((1*r*,3*r*)-3-(3-chloro-4-cyanophenoxy)-2,2,4,4-tetramethylcyclobutyl)-*N*^4^-(8-(3-(8-((5-(trifluoromethyl)cyclohexa-1,3-diene)-1-sulfonamido)quinolin-5-yl)**

**benzamido)octyl)terephthalamide (3-3)**

The compound **3-3** (13.3 mg, 13% yield) was synthesized from **AL** and **S12d** according to the same procedures as the **3-1** preparation. **^1^H NMR** (500 MHz, CDCl_3_) δ 8.76 (d, *J* = 4.0 Hz, 1H), 8.17 (s, 1H), 8.12 (dd, *J* = 8.5, 2.0 Hz, 1H), 8.09 (d, *J* = 7.5 Hz, 1H), 7.87 (d, *J* = 7.5 Hz, 1H), 7.83 – 7.79 (m, 6H), 7.70 (d, *J* = 7.5 Hz, 1H), 7.56 (d, *J* = 8.5 Hz, 1H), 7.54 – 7.52 (m, 2H), 7.49 (d, *J* = 7.5 Hz, 1H), 7.41 (d, *J* = 8.0 Hz, 1H), 7.39 (dd, *J* = 8.5, 4.0 Hz, 1H), 6.96 (d, *J* = 2.5 Hz, 1H), 6.81 (dd, *J* = 9.0, 2.5 Hz, 1H), 6.37 – 6.35 (m, 2H), 6.31 (d, *J* = 8.0 Hz, 1H), 4.15 (d, *J* = 8.0 Hz, 1H), 4.07 (s, 1H), 3.46 – 3.41 (m, 4H), 1.64 – 1.58 (m, 4H), 1.34 (s, 8H), 1.28 (s, 6H), 1.23 (s, 6H). **^13^C NMR** (126 MHz, CDCl_3_) δ 167.4, 166.9, 166.6, 162.7, 149.0, 140.7, 139.3, 138.8, 138.5, 137.8, 137.0, 135.5, 135.3, 134.9, 134.7, 133.0, 133.0, 131.7 (q, *J* = 34.0 Hz), 130.5, 130.0, 129.8 (q, *J* = 3.8 Hz), 129.0, 128.7, 127.6, 127.5 (2C), 127.3 (2C), 126.7, 126.3, 124.5 (q, *J* = 3.8 Hz), 123.2 (q, *J* = 273.4 Hz), 122.5, 117.0, 116.5, 115.6, 114.3, 105.3, 84.9, 59.0, 40.5 (2C), 40.3, 40.3, 29.7, 29.6, 29.1 (2C), 26.9, 26.9, 23.8 (2C), 23.7 (2C). **MS (ESI)**: m/z 1007.3 [M + H]^+^. **HRMS (ESI)**: m/z [M + H]^+^ calcd for C_54_H_54_ClF_3_N_6_O_6_S, 1007.3539; found, 1007.3540.

**N^1^-((1r,3r)-3-(3-chloro-4-cyanophenoxy)-2,2,4,4-tetramethylcyclobutyl)-N4-(10-(3-(8-((5-(trifluoromethyl)cyclohexa-1,3-diene)-1-sulfonamido)quinolin-5-yl)benzamido)decyl)terephthalamide (3-4)**

The compound **3-4** (39.2 mg, 38 % yield) was synthesized from **AL** and **S12e** according to the same procedures as the **3-1** preparation. **^1^H NMR** (500 MHz, CDCl_3_) δ 8.76 (dd, *J* = 4.0, 1.5 Hz, 1H), 8.17 (s, 1H), 8.12 – 8.08 (m, 2H), 7.86 (d, *J* = 8.0 Hz, 1H), 7.82 – 7.78 (m, 6H), 7.70 (d, *J* = 8.0 Hz, 1H), 7.56 – 7.47 (m, 4H), 7.41 – 7.37 (m, 2H), 6.96 (d, *J* = 2.5 Hz, 1H), 6.80 (dd, *J* = 9.0, 2.5 Hz, 1H), 6.44 – 6.33 (m, 3H), 4.15 (d, *J* = 8.0 Hz, 1H), 4.07 (s, 1H), 3.45 – 3.40 (m, 4H), 1.62 – 1.57 (m, 4H), 1.36 – 1.29 (m, 10H), 1.28 (s, 6H), 1.26 – 1.25 (m, 2H), 1.22 (s, 6H). **^13^C NMR** (126 MHz, CDCl_3_) δ167.3, 167.0, 166.6, 162.7, 149.0, 140.6, 139.2, 138.8, 138.5, 137.8, 137.0, 135.5, 135.3, 135.0, 134.7, 133.0, 132.9, 131.7 (q, *J* = 34.0 Hz), 130.5, 130.0, 129.8 (q, *J* = 3.8 Hz), 129.0, 128.7, 127.6, 127.5 (2C), 127.3 (2C), 126.7, 126.3, 124.5 (q, *J* = 3.8 Hz), 123.2 (q, *J* = 273.4 Hz), 122.5, 116.9, 116.5, 115.5, 114.3, 105.3, 84.9, 59.0, 40.5 (2C), 40.4, 40.4, 29.7, 29.6, 29.4 (2C), 29.2, 29.2, 27.0, 27.0, 23.8 (2C), 23.6 (2C).  **MS (ESI)**: m/z 1035.3 [M + H]^+^. **HRMS (ESI)**: m/z [M + H]^+^ calcd for C_56_H_58_ClF_3_N_6_O_6_S, 1035.3852; found, 1035.3851.

***N*^1^-((1*r*,3*r*)-3-(3-chloro-4-cyanophenoxy)-2,2,4,4-tetramethylcyclobutyl)-*N*^4^-(2-(2-(2-(3-(8-((3-(trifluoromethyl)phenyl)sulfonamido)quinolin-5-yl)benzamido)**

**ethoxy)ethoxy)ethyl)terephthalamide (3-5)**

The compound **3-5** (18.2 mg, 18 % yield) was synthesized from **AL** and **S12g** according to the same procedures as the **3-1** preparation. **^1^H NMR** (500 MHz, CDCl_3_) δ 8.76 (dd, *J* = 4.5, 1,5Hz, 1H), 8.18 (s, 1H), 8.10 (d, *J* = 8.0 Hz, 2H), 7.86 (d, *J* = 8.0 Hz, 1H), 7.82 – 7.78 (m, 5H), 7.70 (d, *J* = 8.0 Hz, 1H), 7.57 – 7.48 (m, 5H), 7.42 – 7.38 (m, 2H), 6.96 (d, *J* = 2.5 Hz, 1H), 6.81 – 6.76 (m, 3H), 6.46 (d, *J* = 8.0 Hz, 1H), 4.16 (d, *J* = 8.0 Hz, 1H), 4.07 (s, 1H), 3.68 – 3.57 (m, 12H), 1.28 (s, 6H), 1.22 (s, 6H). **^13^C NMR** (126 MHz, CDCl_3_) δ 167.5, 167.1, 166.6, 162.7, 149.1, 140.7, 139.3, 138.8, 138.5, 137.3, 137.3, 135.3, 135.0, 134.8, 134.7, 133.2, 133.0, 131.7 (q, *J* = 34.0 Hz), 130.5, 130.0, 129.8 (q, *J* = 3.8 Hz), 129.0, 128.8, 127.6, 127.5 (2C), 127.4 (2C), 126.7, 126.3, 124.5 (q, *J* = 3.8 Hz), 123.2 (q, *J* = 272.2 Hz), 122.5, 116.9, 116.5, 115.5, 114.3, 105.3, 84.9, 70.4, 70.4, 69.8, 69.8, 59.0, 40.5 (2C), 40.0, 39.9, 23.8 (2C), 23.7 (2C). **MS (ESI)**: m/z 1011.3 [M + H]^+^. **HRMS (ESI)**: m/z [M + Na]^+^ calcd for C_52_H_50_ClF_3_N_6_O_8_S, 1033.2944; found, 1033.2941.

***N*^1^-((1*r*,3*r*)-3-(3-chloro-4-cyanophenoxy)-2,2,4,4-tetramethylcyclobutyl)-*N*^4^-(1-oxo-1-(3-(8-((3-(trifluoromethyl)phenyl)sulfonamido)quinolin-5-yl)phenyl)-5,7,10,13,16-pentaoxa-2-azaoctadecan-18-yl)terephthalamide (3-6)**

The compound **3-6** (14.0 mg, 12 % yield) was synthesized from **AL** and **S12j** according to the same procedures as the **3-1** preparation. **^1^H NMR** (500 MHz, CDCl_3_) δ 8.75 (dd, *J* = 4.5, 1.5 Hz, 1H), 8.18 (s, 1H), 8.12 (dd, *J* = 8.5, 1.5 Hz, 1H), 8.09 (d, *J* = 8.0 Hz, 1H), 7.90 (d, *J* = 7.5 Hz, 2H), 7.88 – 7.85 (m, 3H), 7.77 (d, *J* = 8.0 Hz, 2H), 7.69 (d, *J* = 7.5 Hz, 1H), 7.64 (br.s, 1H), 7.58 (br.s, 1H), 7.56 – 7.48 (m, 3H), 7.46 (d, *J* = 8.0 Hz, 1H), 7.42 (d, *J* = 7.5 Hz, 1H), 7.38 (dd, *J* = 8.5, 4.5 Hz, 1H), 6.95 (d, *J* = 2.5 Hz, 1H), 6.79 (dd, *J* = 8.5, 2.5 Hz, 1H), 6.47 (d, *J* = 8.5 Hz, 1H), 4.13 (d, *J* = 8.0 Hz, 1H), 4.07 (s, 1H), 3.66 – 3.55 (m, 20H), 3.53 – 3.50 (m, 4H), 1.28 (s, 6H), 1.22 (s, 6H). **^13^C NMR** (126 MHz, CDCl_3_) δ 167.2, 166.9, 166.4, 162.5, 148.8, 140.4, 138.8, 138.6, 138.2, 137.4, 136.6, 135.0, 135.0, 134.8, 134.6, 132.7, 132.6, 131.5 (q, *J* = 32.8 Hz), 130.3, 129.7, 129.6 (q, *J* = 3.8 Hz), 128.8, 128.6, 127.6 (2C), 127.4, 126.9 (2C), 126.4, 126.4, 124.3 (q, *J* = 3.8 Hz), 123.0 (q, *J* = 273.4 Hz), 122.2, 116.7, 116.2, 115.2, 114.1, 105.0, 84.6, 70.3 (2C), 70.3, 70.2, 70.2, 70.2, 69.9 (2C), 69.9, 69.8, 58.7, 40.3 (2C), 39.9, 39.8, 23.6 (2C), 23.4 (2C). **MS (ESI)**: m/z 1143.3 [M + H]^+^. **HRMS (ESI)**: m/z [M + Na]^+^ calcd for C_58_H_62_ClF_3_N_6_O_11_S, 1165.3731; found, 1165.3727.

***N*^1^-((1*r*,3*r*)-3-(3-chloro-4-cyanophenoxy)-2,2,4,4-tetramethylcyclobutyl)-*N*^4^-(2-(3-(8-((3-(trifluoromethyl)phenyl)sulfonamido)quinolin-5-yl)benzamido)ethyl)**

**terephthalamide (3-7)**

The compound **3-7** (29.3 mg, 32 % yield) was synthesized from **AL** and **S12a** according to the same procedures as the **3-1** preparation. **^1^H NMR** (500 MHz, CDCl_3_) δ 8.75 (dd, *J* = 4.5, 1.5 Hz, 1H), 8.17 (s, 1H), 8.12 (dd, *J* = 8.5, 1.5 Hz, 1H), 8.08 (d, *J* = 8.0 Hz, 1H), 7.89 – 7.84 (m, 5H), 7.78 (d, *J* = 8.5 Hz, 2H), 7.77 (br.s, 1H), 7.69 (d, *J* = 8.0 Hz, 1H), 7.66 (br.s, 1H), 7.56 (d, *J* = 9.0 Hz, 1H), 7.54 – 7.49 (m, 3H), 7.42 (d, *J* = 7.5 Hz, 1H), 7.37 (dd, *J* = 8.5, 4.5 Hz, 1H), 6.97 (d, *J* = 2.5 Hz, 1H), 6.81 (dd, *J* = 8.5, 2.5 Hz, 1H), 6.38 (d, *J* = 8.0 Hz, 1H), 4.15 (d, *J* = 8.5 Hz, 1H), 4.09 (s, 1H), 3.69 (s, 4H), 1.29 (s, 6H), 1.22 (s, 6H). **^13^C NMR** (126 MHz, CDCl_3_) δ 168.7, 167.9, 166.9, 162.7, 149.0, 140.6, 139.2, 138.8, 138.4, 137.3, 136.8, 135.3, 134.9, 134.7, 134.7, 133.2, 133.0, 131.7 (q, *J* = 32.8 Hz), 130.5, 130.0, 129.8 (q, *J* = 3.8 Hz), 129.0, 128.9, 127.7 (2C), 127.7, 127.3 (2C), 126.7, 126.6, 124.5 (q, *J* = 3.8 Hz), 123.2 (q, *J* = 273.4 Hz), 122.5, 117.0, 116.5, 115.6, 114.3, 105.3, 84.8, 59.0, 41.3, 41.2, 40.5 (2C), 23.8 (2C), 23.6 (2C). **MS (ESI)**: m/z 923.2 [M + H]^+^. **HRMS (ESI)**: m/z [M + H]+ calcd for C_48_H_42_ClF_3_N_6_O_6_S, 923.2600; found, 923.2599.

***N*^1^-((1*r*,3*r*)-3-(3-chloro-4-cyanophenoxy)-2,2,4,4-tetramethylcyclobutyl)-*N*^4^-(2-(2-(3-(8-((3-(trifluoromethyl)phenyl)sulfonamido)quinolin-5-yl)benzamido)ethoxy)**

**ethyl)terephthalamide (3-8)**

The compound **3-8** (39.4 mg, 41 % yield) was synthesized from **AL** and **S12f** according to the same procedures as the **3-1** preparation. **^1^H NMR** (500 MHz, CDCl_3_) δ 8.75 (dd, *J* = 4.0, 2.0 Hz, 1H), 8.16 (s, 1H), 8.10 (dd, *J* = 8.5, 2.0 Hz, 1H), 8.07 (d, *J* = 8.0 Hz, 1H), 7.84 – 7.81 (m, 5H), 7.73 (d, *J* = 8.0 Hz, 2H), 7.69 (d, *J* = 7.5 Hz, 1H), 7.56 (d, *J* = 9.0 Hz, 1H), 7.52 (d, *J* = 8.0 Hz, 1H), 7.50 – 7.48 (m, 2H), 7.40 (d, *J* = 8.0 Hz, 1H), 7.37 (dd, *J* = 8.5, 4.0 Hz, 1H), 6.98 – 6.96 (m, 3H), 6.80 (dd, *J* = 8.5, 2.5 Hz, 1H), 6.33 (d, *J* = 8.0 Hz, 1H), 4.14 (d, *J* = 8.0 Hz, 1H), 4.07 (s, 1H), 3.70 – 3.63 (m, 8H), 1.26 (s, 6H), 1.22 (s, 6H). **^13^C NMR** (126 MHz, CDCl_3_) δ 167.7, 166.9, 166.9, 162.7, 149.0, 140.6, 139.2, 138.8, 138.4, 137.3, 137.1, 135.3, 135.1, 134.9, 134.7, 133.1, 133.0, 131.7 (q, *J* = 34.0 Hz), 130.5, 130.0, 129.8 (q, *J* = 3.8 Hz), 129.0, 128.8, 127.6 (2C), 127.6, 127.2 (2C), 126.6, 126.5, 124.5 (q, *J* = 3.8 Hz), 123.2 (q, *J* = 273.4 Hz), 122.5, 117.0, 116.5, 115.5, 114.3, 105.3, 84.8, 70.0, 69.8, 59.0, 40.5 (2C), 40.1, 40.0, 23.8 (2C), 23.6 (2C). **MS (ESI)**: m/z 967.3 [M + H]^+^. **HRMS (ESI)**: m/z [M + H]^+^ calcd for C_50_H_46_ ClF_3_N_6_O_7_S, 967.2863; found, 967.2863.

***N*^1^-((1*r*,3*r*)-3-(3-chloro-4-cyanophenoxy)-2,2,4,4-tetramethylcyclobutyl)-*N*^4^-(1-oxo-1-(3-(8-((3-(trifluoromethyl)phenyl)sulfonamido)quinolin-5-yl)phenyl)-5,8,11-trioxa-2-azatridecan-13-yl)terephthalamide (3-9)**

The compound **3-9** (10.6 mg, 10% yield) was synthesized from **AL** and **S12h** according to the same procedures as the **3-1** preparation. **^1^H NMR** (500 MHz, CDCl_3_) δ 8.76 (d, *J* = 5.0 Hz, 1H), 8.18 (s, 1H), 8.11 – 8.08 (m, 2H), 7.87 (d, *J* = 8.0 Hz, 1H), 7.81 – 7.78 (m, 4H), 7.74 – 7.73 (m, 2H), 7.70 (d, *J* = 7.5 Hz, 1H), 7.56 – 7.52 (m, 2H), 7.49 – 7.45 (m, 2H), 7.41 – 7.38 (m, 2H), 7.20 (br.s, 1H), 7.09 (br.s, 1H), 6.96 (d, *J* = 2.5 Hz, 1H), 6.80 (dd, *J* = 8.5, 2.5 Hz, 1H), 6.64 (d, *J* = 8.5 Hz, 1H), 4.16 (d, *J* = 8.5 Hz, 1H), 4.09 (s, 1H), 3.64 – 3.53 (m, 16H), 1.29 (s, 6H), 1.21 (s, 6H). **^13^C NMR** (126 MHz, CDCl_3_) δ 167.4, 167.3, 166.6, 162.7, 149.0, 140.6, 139.0, 138.8, 138.4, 137.2, 137.2, 135.2, 134.9, 134.9, 134.7, 133.0, 132.9, 131.7 (q, *J* = 32.8 Hz), 130.5, 130.0, 129.8 (q, *J* = 3.8 Hz), 128.8, 128.9, 127.6 (2C), 127.5, 127.3 (2C), 126.6, 126.4, 124.5 (q, *J* = 3.8 Hz), 123.2 (q, *J* = 274.7 Hz), 122.5, 116.9, 116.5, 115.4, 114.3, 105.2, 84.9, 70.5, 70.4, 70.4, 70.2, 70.0, 69.9, 59.0, 40.6 (2C), 40.1, 39.8, 23.8 (2C), 23.6 (2C). **MS (ESI)**: m/z 1055.3 [M + H]^+^. **HRMS (ESI)**: m/z [M + H]^+^ calcd for C_54_H_54_ClF_3_N_6_O_9_S, 1055.3387; found, 1055.3384.

***N*^1^-((1*r*,3*r*)-3-(3-chloro-4-cyanophenoxy)-2,2,4,4-tetramethylcyclobutyl)-*N*^4^-(1-oxo-1-(3-(8-((3-(trifluoromethyl)phenyl)sulfonamido)quinolin-5-yl)phenyl)-5,8,11,14-tetraoxa-2-azahexadecan-16-yl)terephthalamide (3-10)**

The compound **3-10** (12.3 mg, 11 % yield) was synthesized from **AL** and **S12i** according to the same procedures as the **3-1** preparation. **^1^H NMR** (500 MHz, CDCl_3_) δ 8.76 (d, *J* = 4.5 Hz, 1H), 8.19 (s, 1H), 8.10 (d, *J* = 8.5 Hz, 2H), 7.86 (d, *J* = 7.5 Hz, 2H), 7.83 – 7.81 (m, 3H), 7.72 – 7.66 (m, 4H), 7.57 – 7.52 (m, 3H), 7.48 – 7.43 (m, 2H), 7.42 – 7.37 (m, 2H), 6.96 (d, *J* = 2.5 Hz, 1H), 6.80 (dd, *J* = 9.0, 2.5 Hz, 1H), 6.62 (d, *J* = 8.5 Hz, 1H), 4.15 (d, *J* = 8.0 Hz, 1H), 4.09 (s, 1H), 3.64 – 3.48 (m, 20H), 1.29 (s, 6H), 1.21 (s, 6H). **^13^C NMR** (126 MHz, CDCl_3_) δ 167.4, 167.4, 166.7, 162.7, 149.0, 140.7, 138.9, 138.8, 138.4, 137.4, 137.1, 135.2, 135.0, 135.0, 134.8, 132.9, 132.9, 131.7 (q, *J* = 34.0 Hz), 130.5, 130.0, 129.8 (q, *J* = 3.8 Hz), 129.0, 128.7, 127.7(2C), 127.6, 127.2 (2C), 126.7, 126.6, 124.5 (q, *J* = 3.8 Hz), 123.2 (q, *J* = 273.4 Hz), 122.5, 116.9, 116.5, 115.4, 114.3, 105.2, 84.9, 70.5, 70.5, 70.4, 70.3, 70.3, 70.3, 70.1, 70.1, 59.0, 40.6 (2C), 40.0, 39.9, 23.8 (2C), 23.7 (2C). **MS (ESI)**: m/z 1099.3 [M + H]^+^. **HRMS (ESI)**: m/z [M + Na]^+^ calcd for C_56_H_58_ClF_3_N_6_O_10_S, 1121.3468; found, 1121.3468.

**1.2 Common reagents and antibodies**

The anti-BRD4 (#13440) Rabbit mAb and anti-BRDT (#93069) Rabbit monoclonal antibody were purchased from Cell Signaling Technology. The anti-BRD2 antibody (#49970), anti-BRD3 antibody (#54675) and anti-β-action antibody (#21338) were purchased from Signalway Antibody LLC. The anti-Ubiquitin antibody (BM4359) were purchased from BOSTER Biological Technology. The anti-Vinculin antibody (RMAB50122) and HRP Goat Anti-Rabbit IgG (H+L) (SAB48169) were purchased from Bioswamp Biological Technology. The anti-AR antibody (22089-1-AP) was purchased from Proteintech Group, Inc. The anti-SKP2 antibody (HA 500427) was purchased from Huabio Biological Technology. The anti-SKP2 antibody (15010-1-AP) was purchased from Proteintech Group, Inc used for CO-IP experiments. The Clarity and Clarity Max ECL Western Blotting Substrates (#1705061) was purchased from Bio-Rad. Cell Counting Kit-8 (CCK-8) Kit (C0005) was purchased from TargetMol. Compound **MG132** (HY-13259), **MLN4924** (HY-70062) ,**MZ1** (HY-107425), **dBET1** (HY-101838) and Protein A/G Magnetic Beads (HY-K0202) were purchased from MedChemExpress (MCE). PEI MAX® - Transfection Grade Linear Polyethylenimine Hydrochloride (MW 40,000) was purchased from Polysciences, Inc. The 250 kDa plus protein marker (MP202) was purchased from Vazyme Biotech Co., Ltd. Puromycin (P8230) and BCA Protein Quantification Kit (PC0020) were purchased from Solarbio Science & Technology Co., Ltd. Coomassie brilliant blue solution (P0003S) was purchased from Beyotime Biotechnology.

**1.3 Cell culture**

MV-4-11 cells (RRID: CVCL_0064) were purchased from the cell bank of the Chinese Academy of Sciences and cultured in IMDM medium (GIBCO) containing 10% (v/v) fetal bovine serum (Excell bio, FSP500) in a cell incubator at 37 °C with 5% CO_2_. MOLT-4 cells (RRID: CVCL_0013), MOLM-13 cells (RRID: CVCL_2119) and 22RV1 cells (RRID: CVCL_1045) were purchased from Guangzhou ewell Bio-technology Co. Ltd and cultured in 1640 medium containing 10% (v/v) fetal bovine serum (Excell bio, FSP500). NCI-H2009 cells (RRID: CVCL_1514) was purchased from Guangzhou ewell Bio-technology Co. Ltd and cultured in DMEM/F12 medium (GIBCO) containing 10% (v/v) FBS in a cell incubator at 37 °C with 5% CO_2_. MDA-MB-231 (RRID: CVCL_0062) cells and HEK293T cells (RRID: CVCL_0063) were obtained from the research group of Professor Deng Wenbin, School of pharmacy, Sun Yat sen University (Shenzhen), and cultured in DMEM medium containing 10% (v/v) FBS. All cells were cultured in a cell incubator at 37 °C with 5% CO_2_, with regular confirmation of sterility.

**1.4 Protein expression, extraction, and purification**

The cDNA fragment encoding the human SKP2 (residues 95-419) was cloned into the pGEX-4T-1 expression vector downstream of the sequence for glutathione S-transferase (GST) tag. To enhance the stability of the purified SKP2 protein, the human SKP1 gene sequence was inserted into the plasmid vector, as the co-expression of SKP2 and SKP1 can improve SKP2 protein stability^[2]^. The plasmid vector was constructed by Tsingke Biotechnology Co., Ltd. The resulting recombinant plasmid was transformed into E. coli BL21 (DE3) competent cells for protein expression. A single positive colony was inoculated into LB medium containing 100 µg/mL ampicillin and cultured at 37°C with shaking until the OD_600_ reached 0.6-0.9. Protein expression was then induced by adding IPTG to a final concentration of 0.4 mM, and the culture was continued at 18°C for 20 hours. The cells were harvested by centrifugation at 3900 ×g for 30 minutes at 4°C. The cell pellet was resuspended in ice-cold lysis buffer (1 × PBS, 200mM NaCl, 0.3% β-Mercaptoethanol, 1 mM PMSF，pH 7.4) and lysed by sonication on ice (250 W, sonication 5 s, interval 3 s, 25 min). The cell lysate was clarified by centrifugation at 12500 ×g for 30 minutes at 4°C to remove cellular debris, and the SKP2 proteins containing the N-terminal GST tag in the supernatant were purified using a GSTSep Glutathione 4FF Chromatography Column (Yeasen, 20510ES25). Recombinant GST-SKP2 proteins were finally collected by elution buffer containing reduced glutathione (1 × PBS, 200 mM NaCl, 0.3% β-mercaptoethanol, 1 mM PMSF, 30 mM reduced glutathione, pH 8.0). Following affinity chromatography, the eluted protein fractions were pooled and subjected to simultaneous concentration, desalting, and buffer exchange (1 × PBS, 200 mM NaCl，pH 7.4) using Amicon^®^ Ultra centrifugal ultrafiltration tube, 30 kDa MWCO (Millipore, UFC9030). The protein's molecular weight and purity were analyzed by SDS-PAGE with Coomassie Brilliant Blue staining. The final protein concentration was determined by the Bradford method (Beyotime, P0006C), and the purified protein was aliquoted, flash-frozen in liquid nitrogen, and stored at -80 °C for subsequent use.

**1.5 Co-Immunoprecipitation (CO-IP) assay**

MV-4-11 cells were plated and treated with compound **22d**, **SL**, **SL1**, **SL2**, **SL3** or an equal volume of DMSO for 8 hour. The cells were then lysed for 40 minutes on ice using RIPA lysis buffer (Beyotime, P0013B), followed by centrifugation at 12000 ×g for 10 minutes at 4°C to collect the supernatant. The cell lysate was incubated with SKP2 polyclonal antibody (Proteintech Group, Inc, 15010-1-AP, 1:30) at 4°C for 16 hours. Subsequently, the complex was incubated with protein A/G magnetic beads (MCE, HY-K0202) at 4°C for 6 hours. The immunocomplex was washed four times with lysis buffer, and then 50 µL of 1× protein loading buffer was added. The samples were denatured at 95°C for 8 minutes before performing Western blotting to detect the levels of SKP2 and CKS1.

**1.6 Cellular Thermal Shift Assay (CETSA)**

MV-4-11 cells were seeded in 10 cm culture dishes and treated with the corresponding concentrations of compound **22d**, **SL**, or an equal volume of DMSO for 1 hour. The cells were then collected and resuspended in 500 µL of PBS buffer, and evenly distributed into eight 1.5 mL microcentrifuge tubes (EP tubes), with 20 µL taken from each tube as the loading control. The cells were heated for 10 minutes at the following temperatures: 42°C, 43.9°C, 46.2°C, 49.9°C, 54.4°C, 58.9°C, 63.3°C, and 68.0°C. After heating, the cells were subjected to freeze-thaw cycles in liquid nitrogen three times. The lysates were then centrifuged at 12000 ×g at 4°C to collect the supernatant. Finally, 5 × protein loading buffer was added, and the samples were heated at 95°C for 10 minutes. Western blotting was performed to detect the SKP2 protein and the loading control β-actin levels. In addition, MV-4-11 cells were treated with varying concentrations of **SL** for 1 hour. After collection, the cells were heated at 55°C for 10 minutes and lysed on ice with RIPA lysis buffer for 40 minutes. The lysates were then centrifuged at 12000 × g and 4°C for 30 minutes. The resulting supernatants were collected, mixed with 5 × protein loading buffer, and boiled at 95°C for 10 minutes. Finally, SKP2 levels and the loading control β-actin were assessed by Western Blot analysis.

**1.7 Molecular Docking**

The crystal structure of SKP1-SKP2-CKS1-P27 (PDB 2AST) was used for molecular docking was downloaded from RCSB protein data bank (http://www.pdb.org)^[3]^. It was used as a template which was transformed to a PDBQT format file after the water and P27 were removed. Based on the binding positions of P27, we selected the seven amino acids W2265, R2294, D2319, R2344, S3041, E3040, and N3045 as the center of the docking box, with a pocket size of 20 Å^[4]^. The docking analysis was then conducted by Schrödinger Maestro software (Release 2019-2, Schrödinger LLC, New York, NY, 2019) to predict the possible binding mode of compounds with SKP2 and the results were processed and illustrated using open-source PyMOL software.

**1.8 Preparation of cell lysates**

After treating the cells with DMSO or compounds, adherent cells were digested with trypsin (GIBCO, 25200072), followed by termination of digestion with culture medium. The cells were then centrifuged at 5000 xg for 10 minutes. For suspended cells, direct centrifugation was performed. The cell pellets were washed twice with pre-chilled PBS buffer, then lysed under ice bath conditions for 40 minutes using RIPA lysis buffer (Beyotime, P0013B) supplemented with a protease and phosphatase inhibitor cocktail (Beyotime, P1048) and PMSF (Beyotime, ST507). Following lysis, the samples were centrifuged at 15000 xg for 20 minutes at 4°C, and the supernatant was collected. Protein concentration was determined using a BCA protein assay kit, after which 5 × SDS-PAGE sample buffer was added and the samples were denatured in a metal bath at 95 °C for 10 minutes.

**1.9 Western Blot analysis**

After preparing the protein samples, proteins were separated using 8% ~ 12% SDS-PAGE and transferred to a PVDF membrane. The membrane was then blocked with PBS buffer containing 5% non-fat dry milk at room temperature for 2 hours. Subsequently, the samples were incubated overnight at 4°C with the appropriate primary antibodies at suitable concentrations. After washing with PBST, the membrane was treated with HRP Goat Anti-Rabbit IgG (H + L) at a dilution of 1/5000. Finally, the membrane was imaged using Clarity Max ECL Western Blotting Substrates and a chemiluminescence detection system, and the results were analyzed with ImageJ software.

**1.10 CCK8 assay**

To evaluate the inhibitory effects of the compounds on tumor cell proliferation, suspension cells were seeded at a density of 30000−50000 cells per well, while adherent cells were seeded at 3000−5000 cells per well in a 96-well plate. Adherent cells were allowed to attach for 12 hours before adding different concentrations of the compounds. The MV-4-11 cells were then incubated for 72 hours in a 37°C incubator with 5% CO₂. 22RV1 cells were plated at 800 cells / well and incubated in a 37°C incubator containing 5% CO_2_ for 7 days. The solution was changed every 3 days and the drug was added. Finally, 10 µL of CCK-8 reagent was added to each well and incubated for 4 hours, followed by measurement of absorbance at 450 nm using a microplate reader. Data were normalized to the DMSO-treated cells, and half-maximal inhibitory concentration (IC_50_) values were calculated and graphed using GraphPad Prism 9.

**1.11 Immunoprecipitation and ubiquitination detection protocol**

To assess ubiquitination, after treating HEK293T cells with DMSO or 10 μM of **2-1** for 18 hours, 10 μM of the proteasome inhibitor **MG132** was added, and the cells were further treated for 6 hours, followed by washing with cold PBS. The cells were then resuspended in a lysis buffer containing protease and phosphatase inhibitors and lysed under ice bath conditions for 40 minutes. After centrifugation at 15000 ×g for 20 minutes at 4°C, the supernatant was collected. To eliminate non-specific binding, the lysate was incubated with protein A/G beads for 1 hour at 4°C, after which the supernatant was collected again. A specific antibody (anti-BRD4, 1 : 50) was added to the supernatant and incubated gently overnight at 4°C, followed by an additional 2 hours incubation with Protein A/G Magnetic Beads. The beads were washed three times with cold washing buffer to remove unbound proteins, and finally, Western blot analysis was performed to detect the levels of protein ubiquitination.

**1.12 Generation of SKP2 knockdown in HEK293T cell lines**

Using PEI MAX^®^ - Transfection Grade Linear Polyethylenimine Hydrochloride (MW 40000), HEK293T cells were co-transfected with shRNA-containing vectors, PAX2, and VSVG to generate shRNA lentivirus. After 48 hours of transfection, the culture medium containing the virus was collected and used to infect HEK293T cells for another 48 hours. Following this, the cells were subjected to puromycin selection for 6 days. The proportion of successfully infected cells was observed under a fluorescence microscope, and Western blot analysis was performed to verify SKP2 degradation. The shRNA targeting human SKP2 was obtained from Tsingke Biotech in the pLKO.1-CMV-copGFP-PURO vector, with the sequence described below.

shRNA 1:

CCGGGATAGTGTCATGCTAAAGAATCTCGAGATTCTTTAGCATGACACTATCTTTTTT

shRNA 2:

CCGGGCCTAAGCTAAATCGAGAGAACTCGAGTTCTCTCGATTTAGCTTAGGCTTTTTT

shRNA 3:

CCGGCCATTGCCAGGCCAACTATTGCTCGAGCAATAGTTGGCCTGGCAATGGTTTTTT

shRNA 4:

CCGGCCATTGTCAATACTCTCGCAACTCGAGTTGCGAGAGTATTGACAATGGTTTTTT

**1.13 Turbidimetric method for apparent solubility determination**

A series of dilutions for each test compound were prepared in PBS. The final DMSO concentration in all test solutions was maintained at a constant level of 0.5% (v/v) to ensure consistent solvent effects and prevent DMSO-induced precipitation. A PBS solution containing 0.5% DMSO served as the blank control. Following dilution, the 96-well plate was sealed and subjected to continuous shaking at a constant temperature (25°C) for 30 minutes to ensure thorough mixing and reach equilibrium. After the incubation period, the absorbance of each well was immediately measured at a wavelength of 650 nm using the microplate reader^[5]^. The apparent solubility of each compound was defined as the concentration at which precipitation occurred, indicated by an absorbance value exceeding that of the PBS (0.5% DMSO) blank control. To quantitatively determine this endpoint, the linear portion of the absorbance-concentration curve was fitted, and the intersection point of this fitted line with the baseline absorbance (dashed line), as indicated by the arrow, was calculated as the solubility value. Data for each concentration are presented as the mean of three independent experiments.

**1.14** **SKP2-SL binding assay by surface plasmon resonance (SPR)**

Surface plasmon resonance (SPR) assay was performed on the Biacore 1K system (Cytiva) at room temperature. Recombinant purified GST-SKP2 was covalently immobilized on a CM5 sensor chip (Cytiva, 29104988) using an amine coupling kit (Cytiva, BR100050) according to the manufacturer's standard protocol in the Biacore 1K system. The protein immobilization level was 12506 RU. Immediately before SPR assay, compound **SL** was dissolved at 10 mM in dimethyl sulfoxide (DMSO) and then diluted into HBS-EP running buffer (Cytiva, BR100826, pH 7.4, 5% DMSO, 0.05% P20). Interaction assay was performed using a flow rate of 35 μL/min. Contact time was set to 110 s, followed by a dissociation time of 120 s. A reference cell was used to subtract possible nonspecific binding to the chip surface. The raw SPR sensorgrams were collected and analyzed using the Biacore 1K Evaluation Software. The affinity (*K*_D_, dissociation constant), the association rate (*k*_a_), and the dissociation rate (*k*_d_) value were calculated using the Biacore 1K Evaluation Software built-in module “Kinetic / Affinity” under the “1:1 ligand binding” setting with default setups.

**1.15 Generation of SKP2 knockout cell lines using CRISPR/Cas9**

SKP2 knockout cell lines were generated in both HEK293T and 22RV1 backgrounds. The 293T Cells were co-transfected using PEI MAX^®^ transfection (MW 40000) reagent with the LentiCRISPRv2-CMV-ZsGreen-Puro vector, which co-expresses Streptococcus pyogenes Cas9 and a single-guide RNA (sgRNA) targeting the human SKP2 gene, along with the packaging plasmids PAX2 and VSVG for lentivirus production. The viral supernatant was collected 48 hours post-transfection and used to infect fresh HEK293T and 22RV1 cells. Successfully transduced cells were selected with puromycin (2.5 µg/mL) for 6 days to establish stable polyclonal populations. The knockout efficiency of SKP2 was confirmed at the protein level by Western blot analysis. The target sequence of the sgRNA used is as follows:

sgRNA 1: caccgAAATGATCGTGGGCAGCGGA

sgRNA 2: caccgCCGCTGCCCACGATCATTTA

sgRNA 3: caccgAGCTGCTTAGCAAAGTCTGC

sgRNA 4: caccgAGAATCCAGAACACCCAGAA

**1.16 Statistical analysis**

The dose-response curve was generated by fitting the effect (Y) against the logarithmically transformed drug concentration (X) using a four-parameter logistic nonlinear regression model. The x-axis is plotted on a logarithmic (Log_10_) scale. The data were expressed as mean ± standard deviation (SD). Three individual experiments were performed for each data. Differences between two groups were analyzed by the two-tailed Student's t-test. For comparisons involving more than two groups, one-way ANOVA was conducted, followed by Dunnett's post hoc test where applicable. All statistical data were calculated using the GraphPad Prism 9 (GraphPad Software Inc., La Jolla, CA, USA). The *P* values were indicated (ns: *P* > .0.05, **P* < 0.05, ***P* < 0.01, ****P* < 0.001 and *****P* < 0.0001). *P* values less than 0.05 were considered statistically significant.

1. **Supporting tables and figures**

**
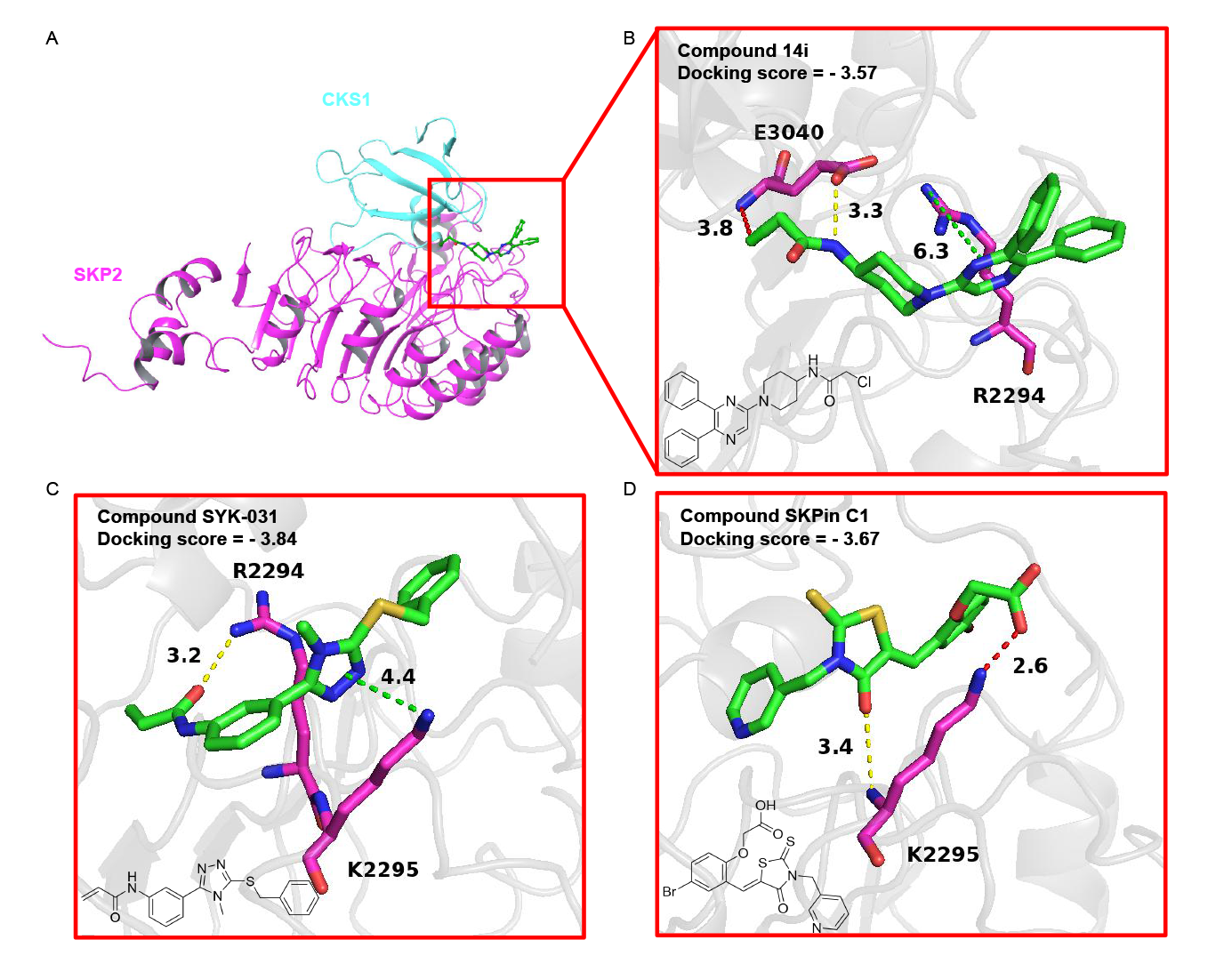
**

**2.1 Supplementary Figure 1.** Predicted binding models of compounds **14i**, **SYK-031** and **SKPin C1** with the SKP2 protein.

(A) Predicted binding models of compounds **14i** and SKP2-CKS1 (2AST). SKP2 and CKS1are presented in cyan and purple, respectively. (B) Predicted binding models of compounds **14i**. (C) Predicted binding models of compounds **SYK-031**. (D) Predicted binding models of compounds **SKPin C1**. Hydrogen bonds are represented by yellow dashed lines, cation-π interactions by green dashed lines, and salt bridges by red dashed lines. R2294 and K2295 are amino acids of SKP2 protein. E3040 is an amino acid of CKS1 protein. The docking analysis was then conducted by Schrödinger Maestro software (Release 2019-2, Schrödinger LLC, New York, NY, 2019) to predict the possible binding mode of compounds with SKP2 and the results were processed and illustrated using open-source PyMOL software.


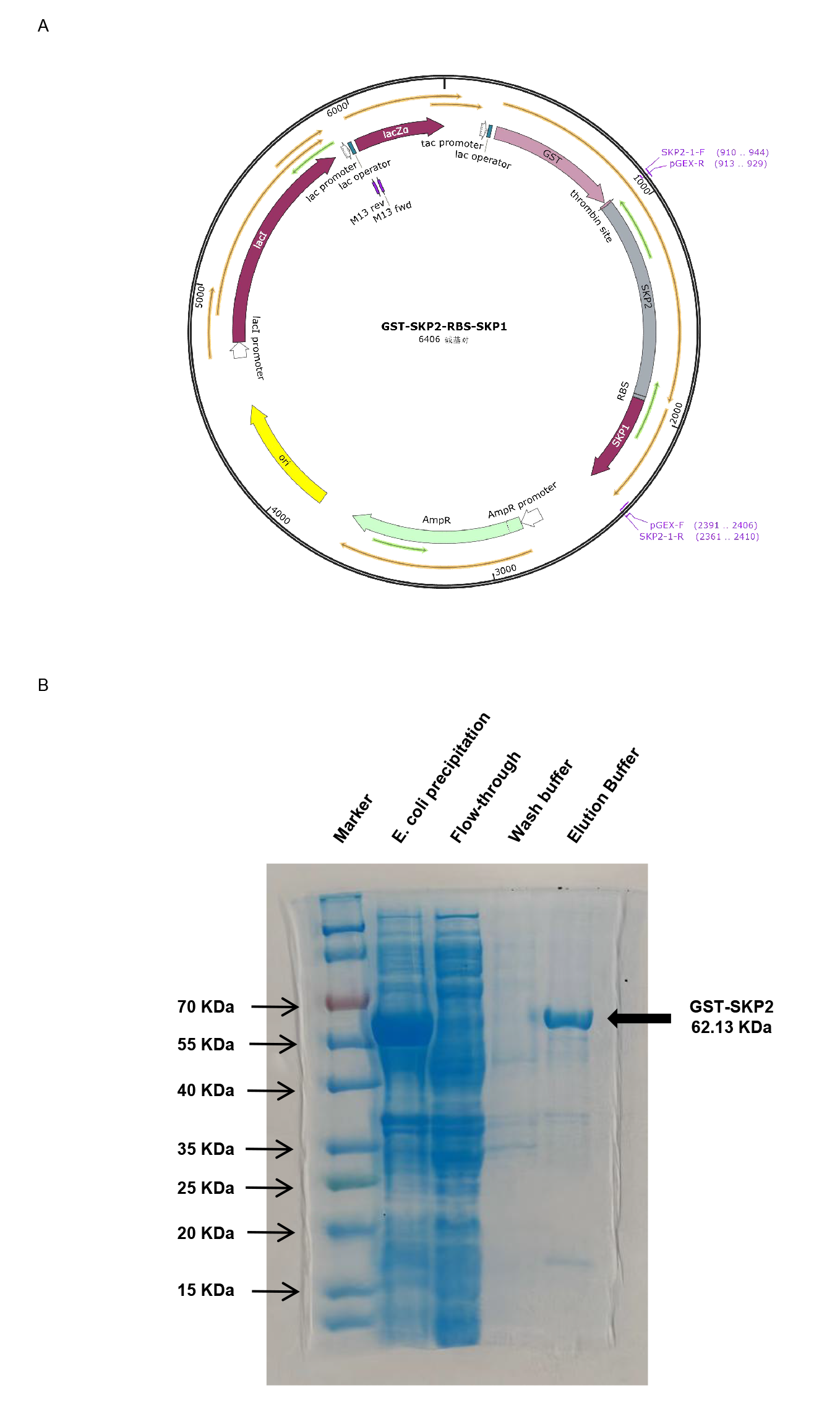


**2.2 Supplementary Figure 2.** Plasmid vector and SDS-PAGE analysis of the purified GST-SKP2 protein.

(A) Plasmid vector information for the GST-SKP2 fusion protein, the SKP2 (95-419) gene was ligated into the pGEX4T-1 plasmid vector. The SKP1 gene was also inserted into this vector, enabling the co-expression of SKP1 protein, which enhances the stability of the SKP2 protein. (B) Recombinant GST-SKP2 was expressed in E. coli and purified using Glutathione Sepharose affinity chromatography. Proteins were separated on a 12% SDS-PAGE gel and visualized with Commassie Blue Staining Solution. Each lane was loaded with 10 μg of protein. Lane Marker: prestained protein ladder. Lane E. coli precipitation: bacterial precipitation after lysis of Escherichia coli cells. Lane Flow-through: the flow-through fraction during the protein purification of the E. coli cell lysate supernatant. Lane Wash buffer: the wash fraction containing proteins that non-specifically bound to the resin. Lane Elution Buffer: purified GST-SKP2 after elution. The arrow marks the dominant band at ~62.13 kDa, consistent with the predicted molecular weight of the GST-SKP2 (GST: ~25.48 kDa + SKP2 fragment: ~36.65 kDa), indicating successful purification of the fusion protein.


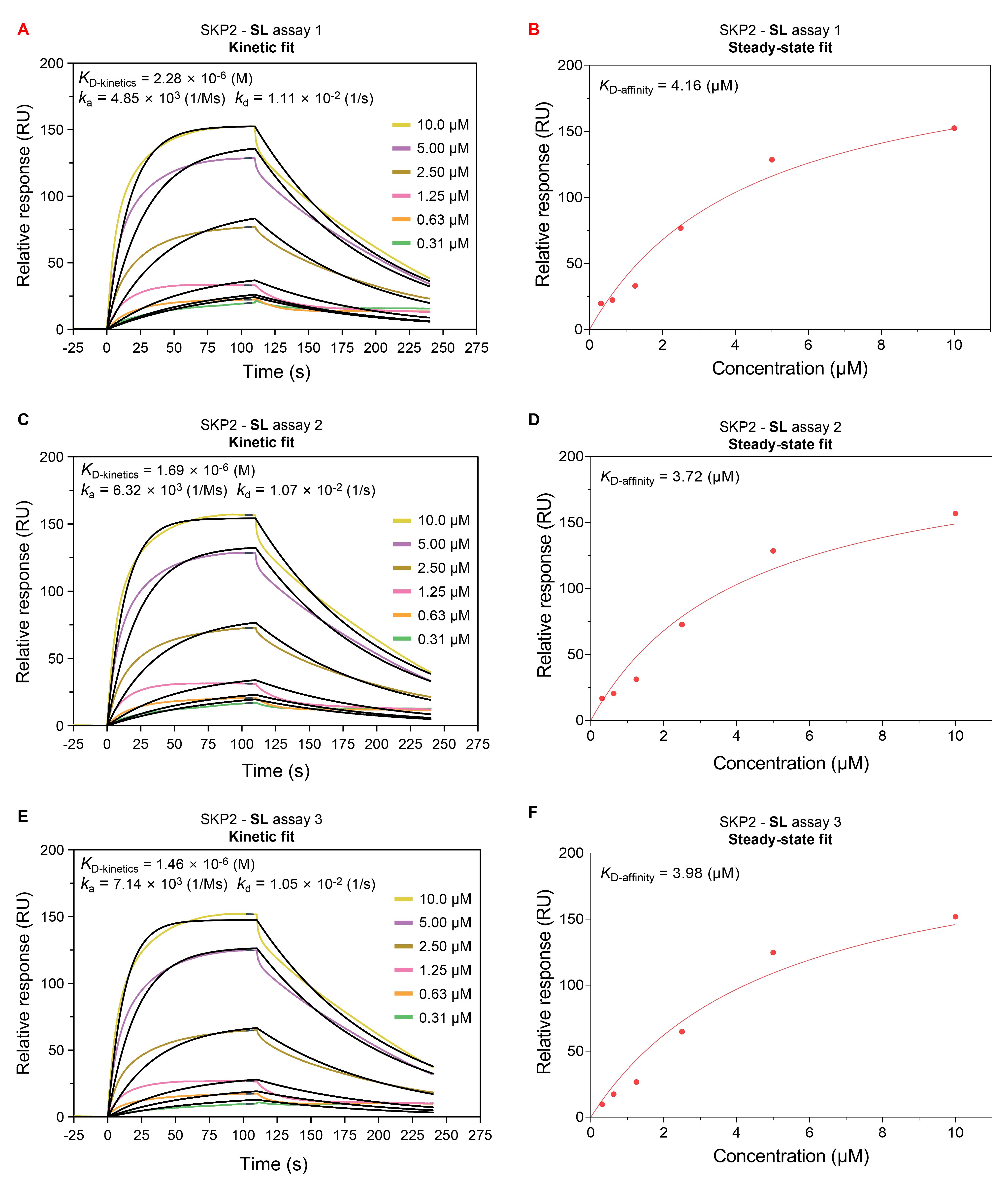


**2.3 Supplementary Figure 3.** SPR sensorgrams showing the interaction between **SL** and SKP2 proteins from three replicate assays.

(A, C, and E) Sensorgrams and fitting curve for the binding of **SL** to SKP2. GST-SKP2 protein was immobilized on a CM5 sensor chip, and then SPR analysis was performed on a Biacore 1K system (cytiva) instrument. The figure includes both experimental data (colored lines) and fitted curves (black lines). (B, D, and F) Steady state plots for the binding of **SL** to SKP2. The affinity (*K*_D_, dissociation constant) of SKP2 binding to **SL** obtained based on the “Kinetic” and “Steady-state” methods were shown in Figures above. The association rate (*k*_a_) and dissociation rate (*k*_d_) of the binding between SKP2 and **SL** were presented in Figure A, C, and E. Figure A and Figure B are presented in the main text as representatives.

**
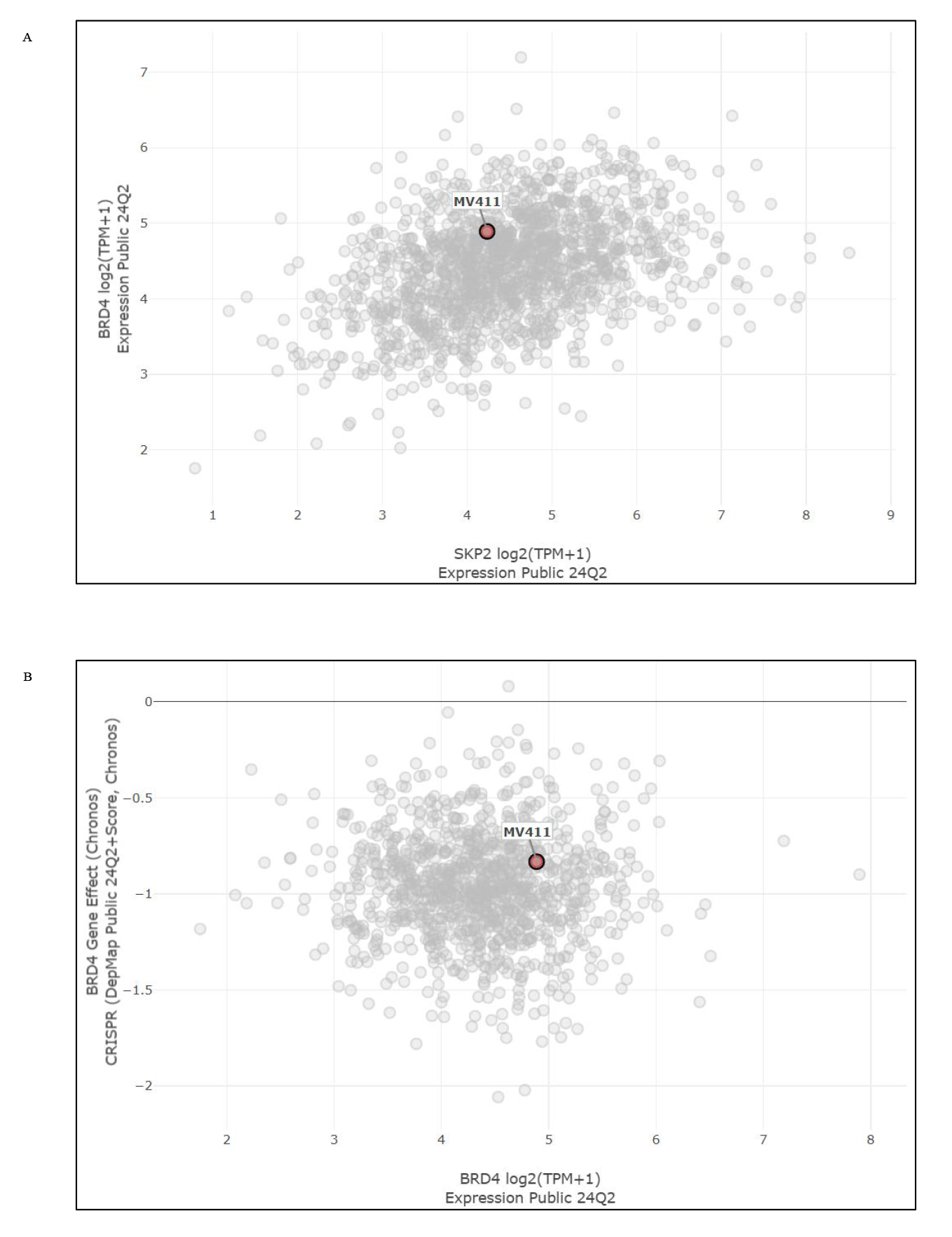
**

**2.4 Supplementary Figure 4.** Bioinformatics analysis of SKP2 and BRD4 expression levels in MV-4-11 cells and the impact of BRD4 knockout on these cells.

(A) Bioinformatics analysis of SKP2 and BRD4 expression levels in MV-4-11 cells, the x-axis represents the expression level of SKP2 protein, while the y-axis represents the expression level of BRD4. The red points indicate MV-4-11 cells, and the gray points represent other tumor cells. TPM, Transcripts Per Million. (B) Bioinformatics analysis of BRD4 expression and its impact on cellular effects upon knockout. The x-axis represents the level of BRD4 expression, while the y-axis indicates the impact of BRD4 knockout on cells, with smaller values reflecting greater effects. The red points denote MV-4-11 cells, and the gray points represent other tumor cells. All data are sourced from the DepMap database (https://depmap.org/).

**
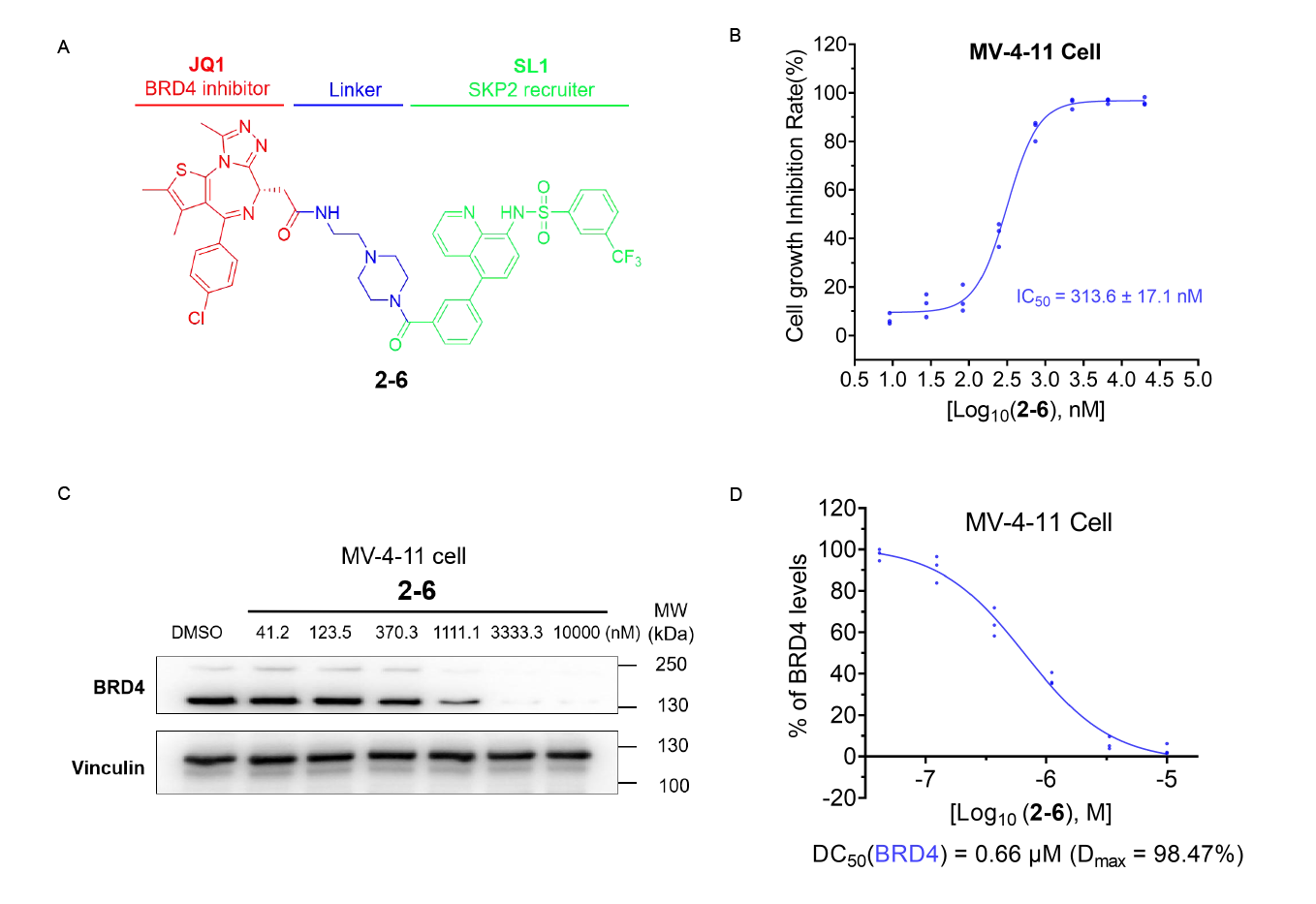
**

**2.5 Supplementary Figure 5.** Compound **2-6** induces BRD4 degradation in MV-4-11 Cells.

(A) Structure of SKP2-recruiting non-covalent PROTAC **2-6**. (B) After treating MV-4-11 cells with **2-6** for 72 hours, the cell proliferation inhibition was assessed using the CCK8 assay, with the data representing the mean ± SD from three independent experiments. (C) Immunoblot analysis results after treating MV-4-11 cells with DMSO and varying doses of **2-6** for 24 hours, along with (D) quantification of BRD4 abundance, half-maximal degradation concentration (DC_50_) values, and observed maximum degradation (D_max_). All data are based on three independent experiments for each group.


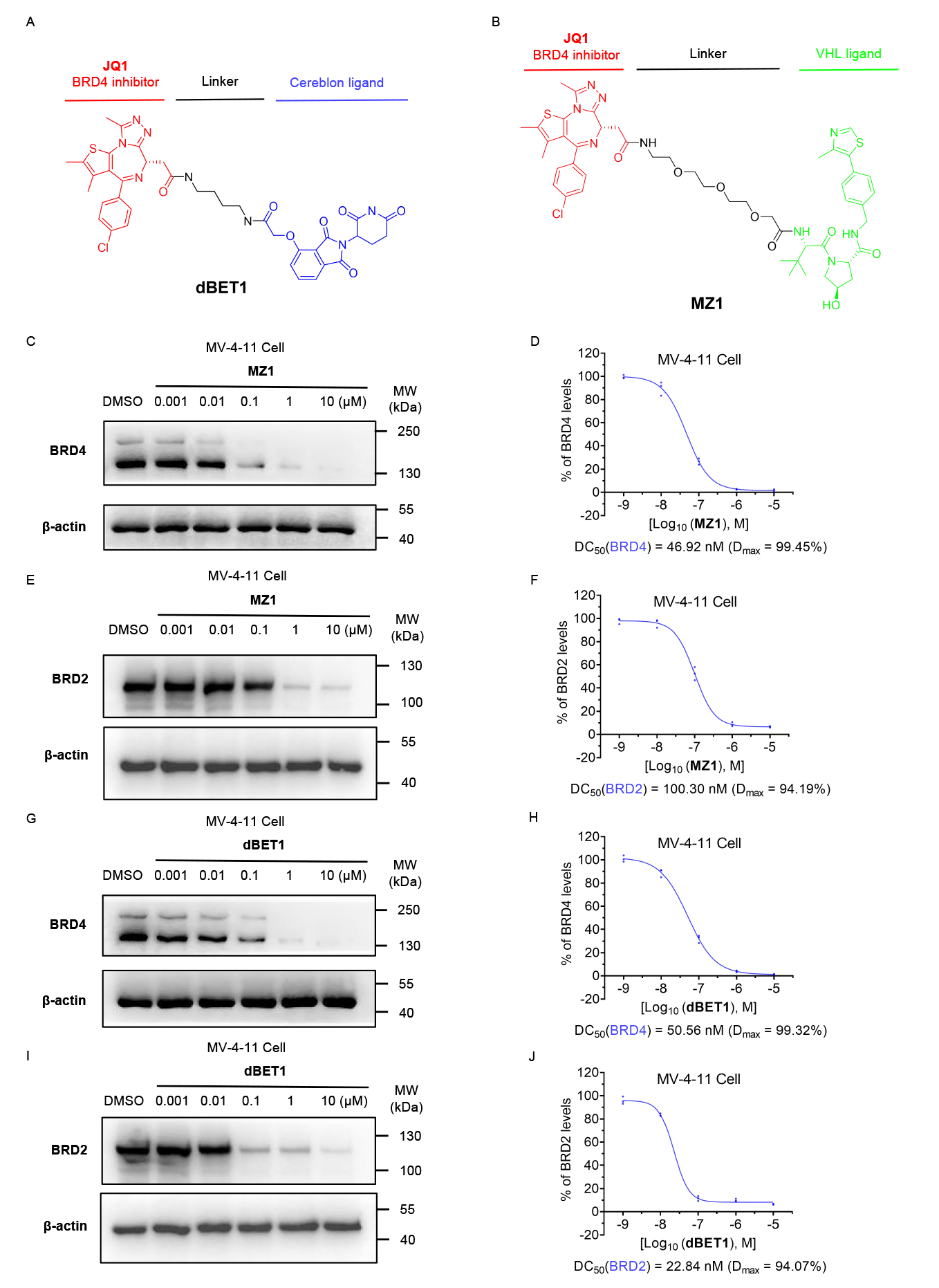


**2.6 Supplementary Figure 6.** Compound **MZ1** and **dBET1** induce BRD4 and BRD2 degradation in MV-4-11 Cells.

(A) Structure of **dBET1**. (B) Structure of **MZ1**. (C) Immunoblot analysis results after treating MV-4-11 cells with DMSO and varying doses of **MZ1** for 24 hours, along with (D) quantification of BRD4 abundance, half-maximal degradation concentration (DC_50_) values, and observed maximum degradation (D_max_). (E) Immunoblot analysis results after treating MV-4-11 cells with DMSO and varying doses of **MZ1** for 24 hours, along with (F) quantification of BRD2 abundance, DC_50_ values, and D_max_. (G) Immunoblot analysis results after treating MV-4-11 cells with DMSO and varying doses of **dBET1** for 24 hours, along with (H) quantification of BRD4 abundance, DC_50_ values, and D_max_. (I) Immunoblot analysis results after treating MV-4-11 cells with DMSO and varying doses of **dBET1** for 24 hours, along with (J) quantification of BRD2 abundance, DC_50_ values, and D_max_. All data are based on three independent experiments for each group.


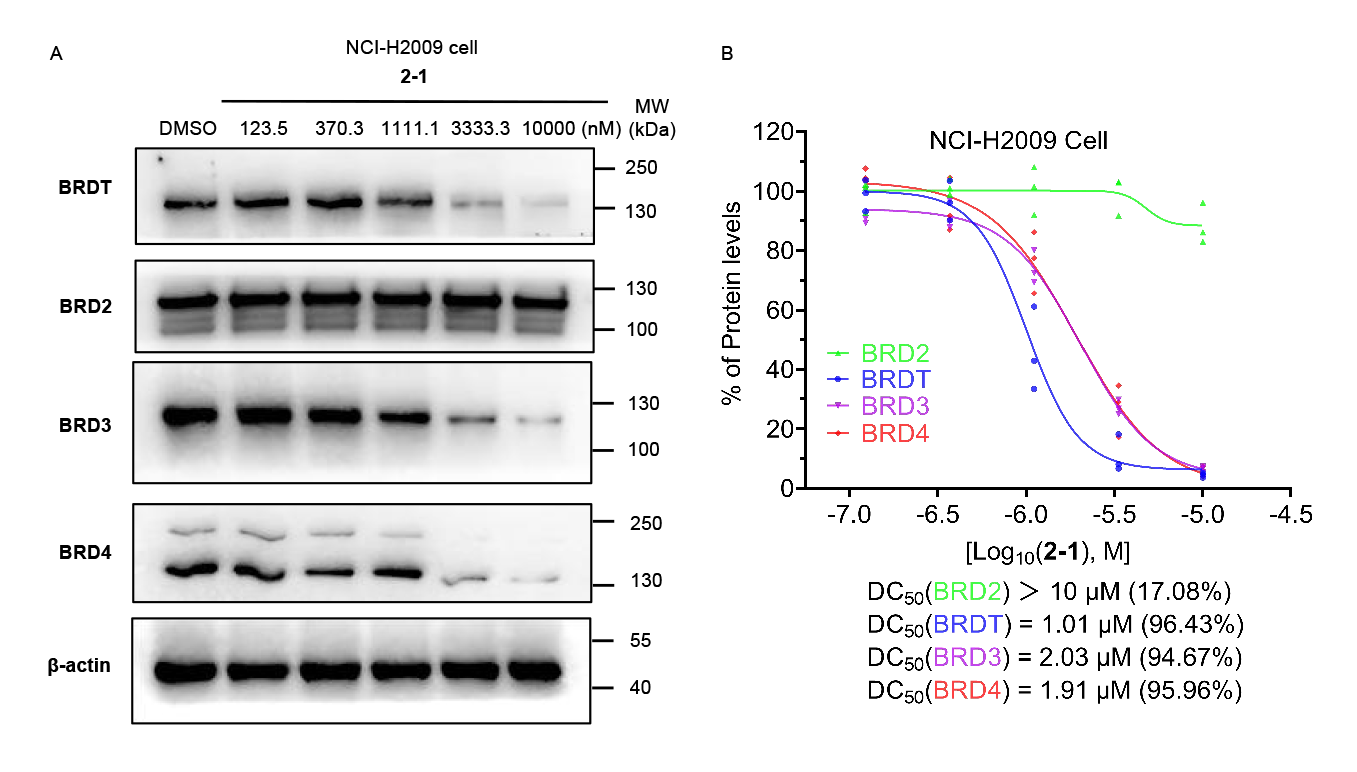


**2.7 Supplementary Figure 7.** Compound **2-1** induces the bromodomain and extraterminal (BET) family of proteins degradation in NCI-H2009 Cells.

(A) Immunoblot analysis results after treating NCI-H2009 cells with DMSO and varying doses of **2-1** for 24 hours, along with (B) quantification of BET family proteins abundance, DC_50_ values, and D_max_. All data are based on three independent experiments for each group.


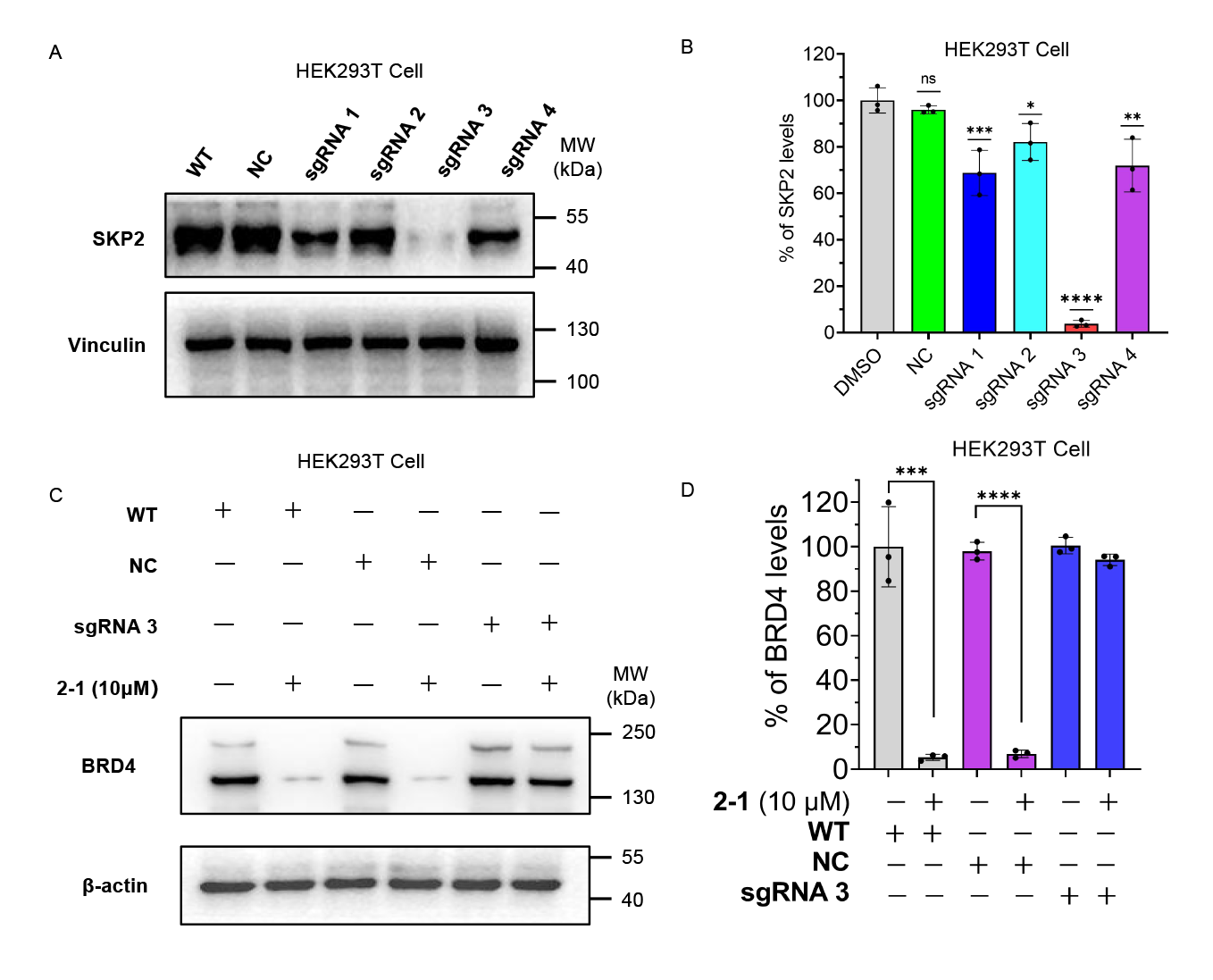


**2.8 Supplementary Figure 8.** Knockout of SKP2 in HEK293T cells rescued the degradation of BRD4 induced by **2-1**.

(A) HEK293T cells were transfected with LentiCRISPRv2-CMV-ZsGreen-Puro plasmids expressing Cas9 and single-guide RNAs (sgRNAs) targeting the SKP2 gene to generate stable SKP2 knockout cell lines. Positive clones were selected using puromycin. The efficiency of SKP2 knockout was confirmed by Western blotting, with (B) the corresponding quantification results shown in the figure A. Data are expressed as mean ± SD from three independent experiments per group. *P*-values were determined by one-way ANOVA with Dunnett's post hoc test for multiple comparisons. ns: *P* > 0.05, **P* < 0.05, ***P* < 0.01, ****P* < 0.001, *****P* < 0.0001 as compared with the WT controls (C) Wild-type, negative control, and SKP2 knockout cells were treated with either DMSO or **2-1** (10 µM) for 24 hours. The protein levels of BRD4, and the loading control β-actin were then determined by Western blotting, and (D) the corresponding quantitative data are presented. WT: Wild-type Cell, NC: Negative control cells, cells transfected with lentivirus packaged with blank plasmid. The data are reported as mean ± SD, reflecting three independent experiments conducted for each group. *P*-values were determined by one-way ANOVA with Dunnett's post hoc test for multiple comparisons. In the quantification results of BRD4, *****P* < 0.0001 as compared with the DMSO controls.


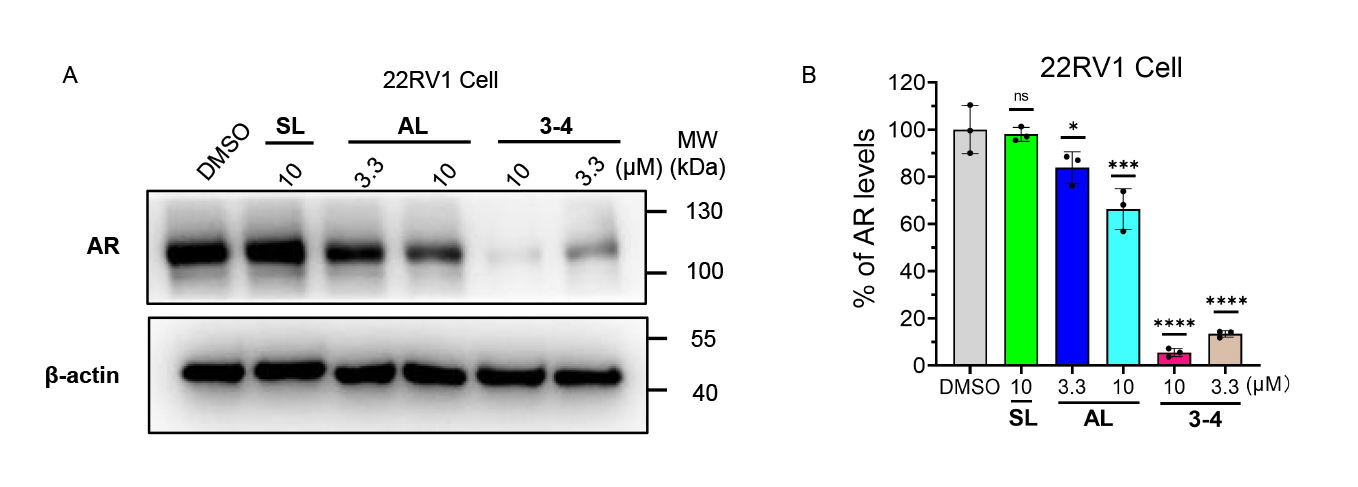


**2.9 Supplementary Figure 9.** AR Ligand **AL** Treatment Partially Degrades AR in 22RV1 Cells.

(A) Immunoblot analysis results after treating 22RV1 cells with DMSO, **SL**, **AL**, and **3-4** for 24 hours, along with (B) quantification of AR and β-actin proteins abundance. All data are based on three independent experiments for each group. The data are reported as mean ± SD, reflecting three independent experiments conducted for each group. *P*-values were determined by one-way ANOVA with Dunnett's post hoc test for multiple comparisons. In the quantification results of AR, ns: *P* > 0.05, **P* < 0.05, ****P* < 0.001, *****P* < 0.0001 as compared with the DMSO controls.


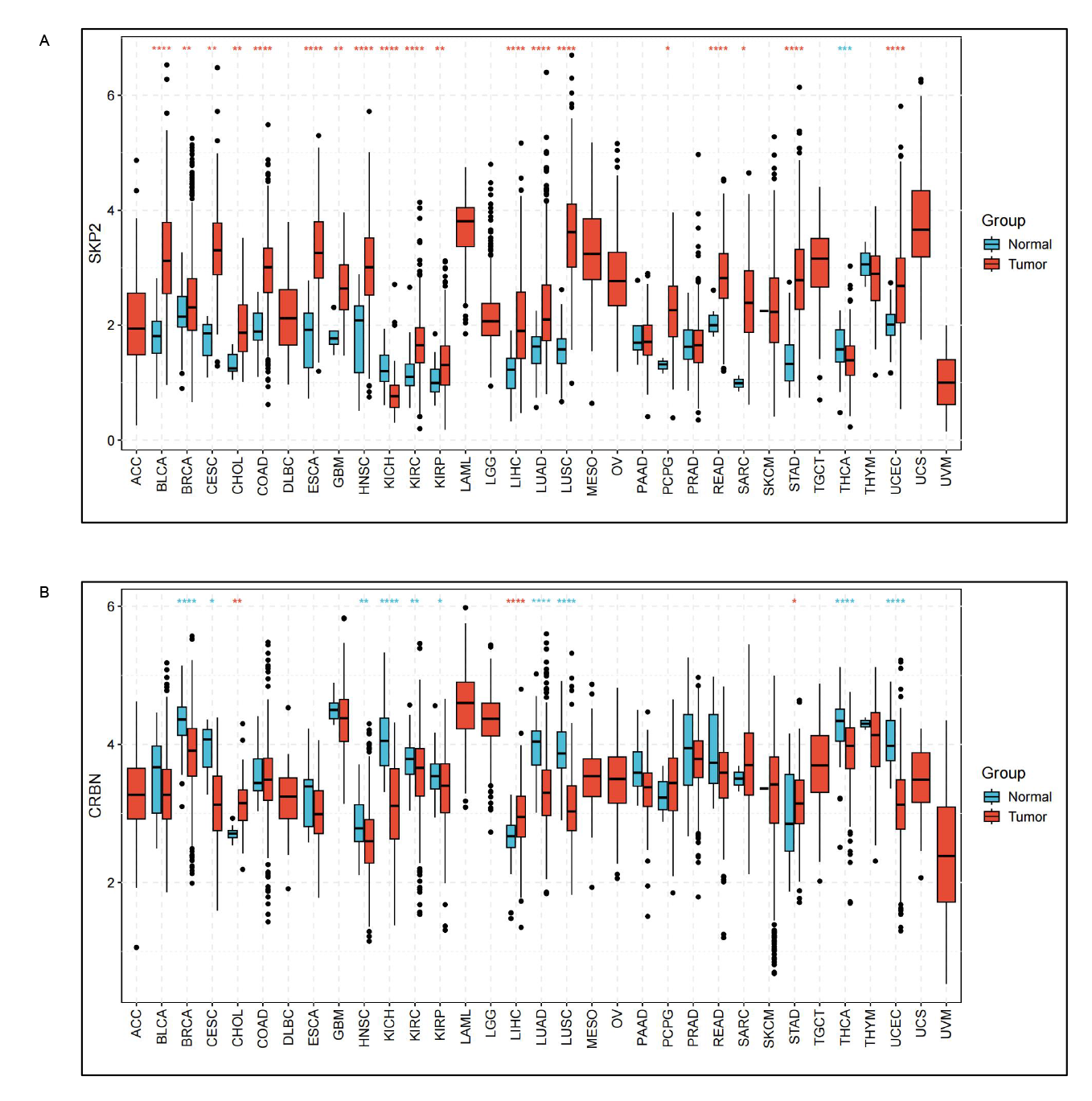


**2.10 Supplementary Figure 10.** Bioinformatics analysis of SKP2 and CRBN expression levels in normal and tumor tissues.

X-axis displays different tumor names. The Y axis represents the expression level. The red rectangle represents the expression level of corresponding proteins in tumors, and the green rectangle represents the expression level of corresponding proteins in normal tissues. The red asterisk represents genes that are significantly over-expressed in tumor tissues compared to normal tissues, the cyan asterisk indicates genes that are significantly under-expressed, and other symbols denote no significant difference. Bioinformatics analysis data are from the Cancer Genome Atlas program (TCGA) database (**https://portal.gdc.cancer.gov/)**.


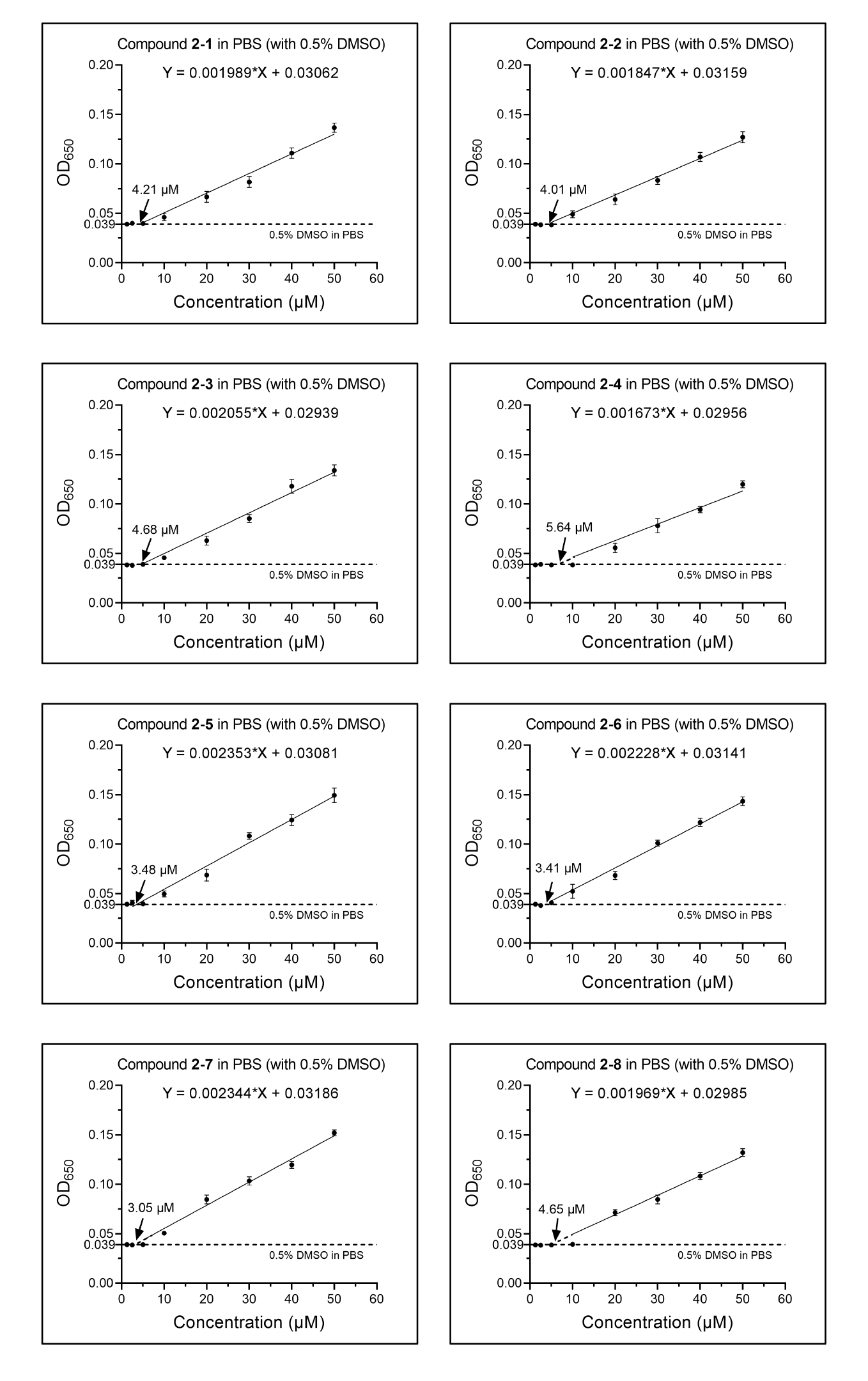


**2.11 Supplementary Figure 11.** Apparent solubility analysis of compounds by the turbidimetric method.

The apparent solubility of compounds **2-1** to **2-8** was determined by a turbidimetric method. Compounds were subjected to serial dilution in PBS (pH 7.4) containing 0.5% DMSO, the samples were shaken and incubated at room temperature for 30 minutes. The turbidity of each solution was monitored by measuring the absorbance at 650 nm using a microplate reader. The dashed line represents the baseline absorbance of the PBS (with 0.5% DMSO) control. The apparent solubility of each compound was defined as the concentration at which precipitation occurred, indicated by an absorbance value exceeding that of the PBS (0.5% DMSO) blank control. To quantitatively determine this endpoint, the linear portion of the absorbance-concentration curve was fitted, and the intersection point of this fitted line with the baseline absorbance (dashed line), as indicated by the arrow, was calculated as the solubility value. Data for each concentration are presented as the mean of three independent experiments.


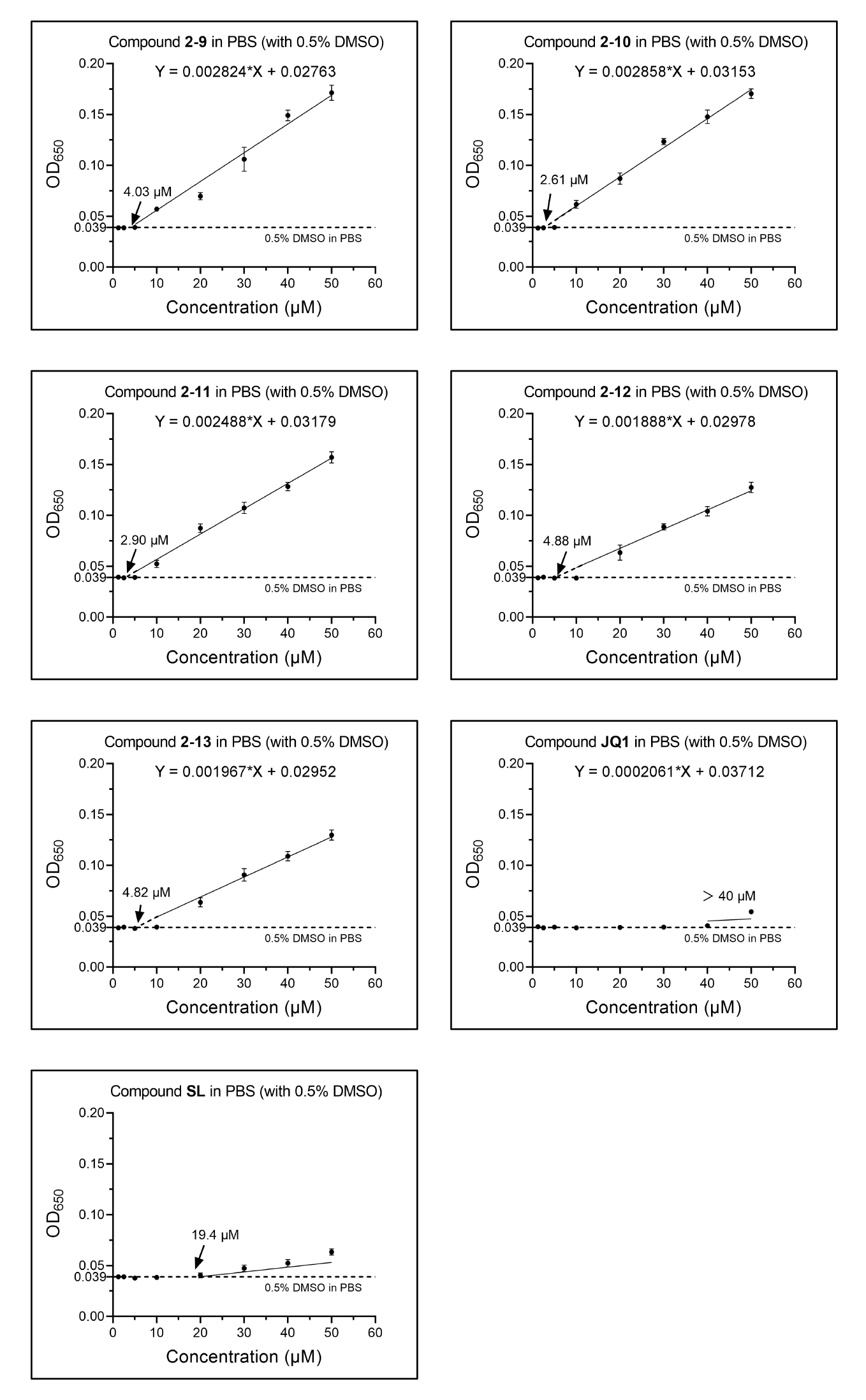


**2.12 Supplementary Figure 12.** Apparent solubility analysis of compounds by the turbidimetric method.

The apparent solubility of compounds **2-9** to **SL** was determined by a turbidimetric method. Compounds were subjected to serial dilution in PBS (pH 7.4) containing 0.5% DMSO, the samples were shaken and incubated at room temperature for 30 minutes. The turbidity of each solution was monitored by measuring the absorbance at 650 nm using a microplate reader. The dashed line represents the baseline absorbance of the PBS (with 0.5% DMSO) control. The apparent solubility of each compound was defined as the concentration at which precipitation occurred, indicated by an absorbance value exceeding that of the PBS (0.5% DMSO) blank control. To quantitatively determine this endpoint, the linear portion of the absorbance-concentration curve was fitted, and the intersection point of this fitted line with the baseline absorbance (dashed line), as indicated by the arrow, was calculated as the solubility value. Data for each concentration are presented as the mean of three independent experiments.

1. **The triplicate data and original bands from the Western blot experiments.**


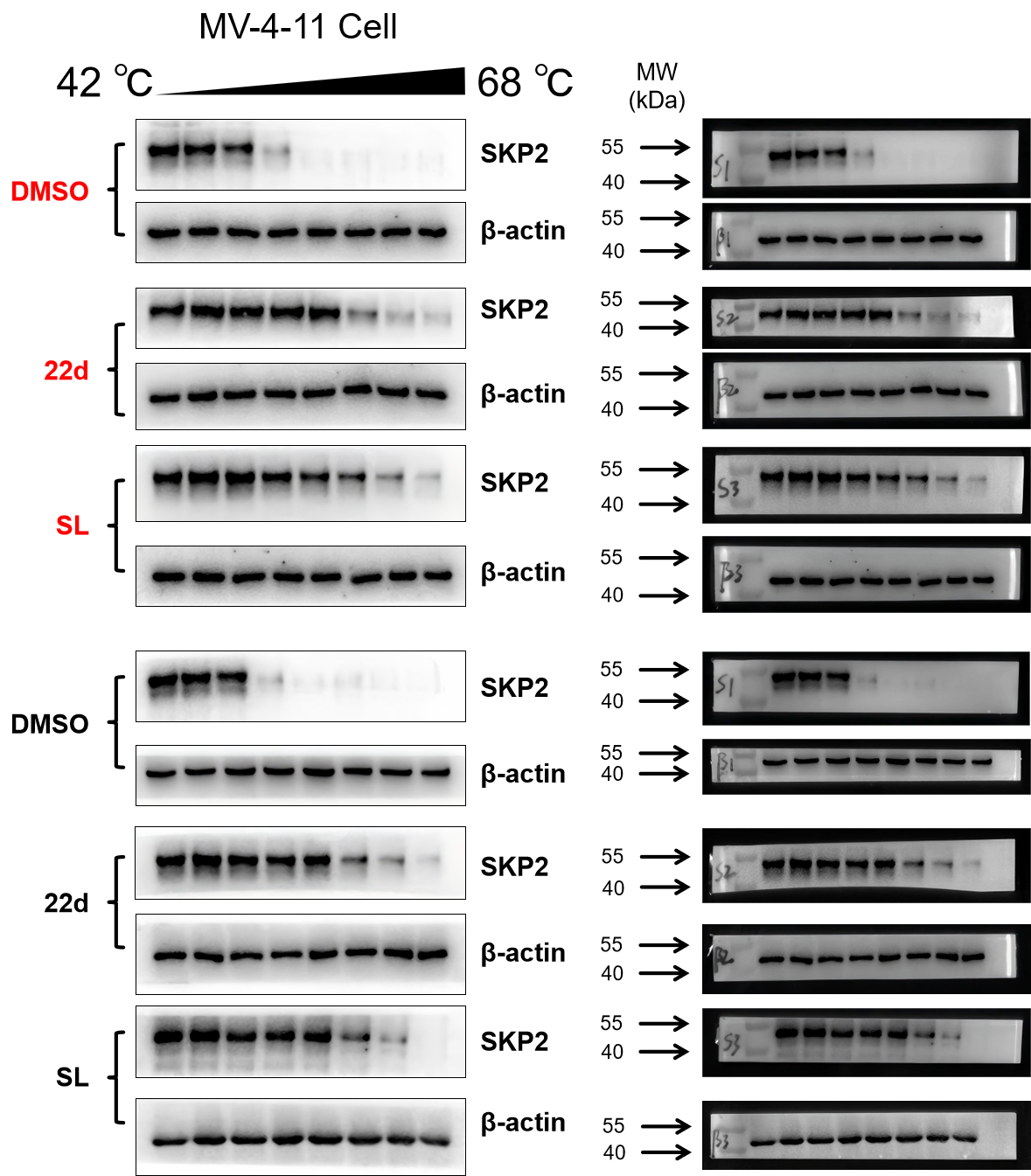


**Supplementary Figure 13.** Three replicates and original bands of Figure 2E (a). The bands highlighted in red are shown in the main figure.

**
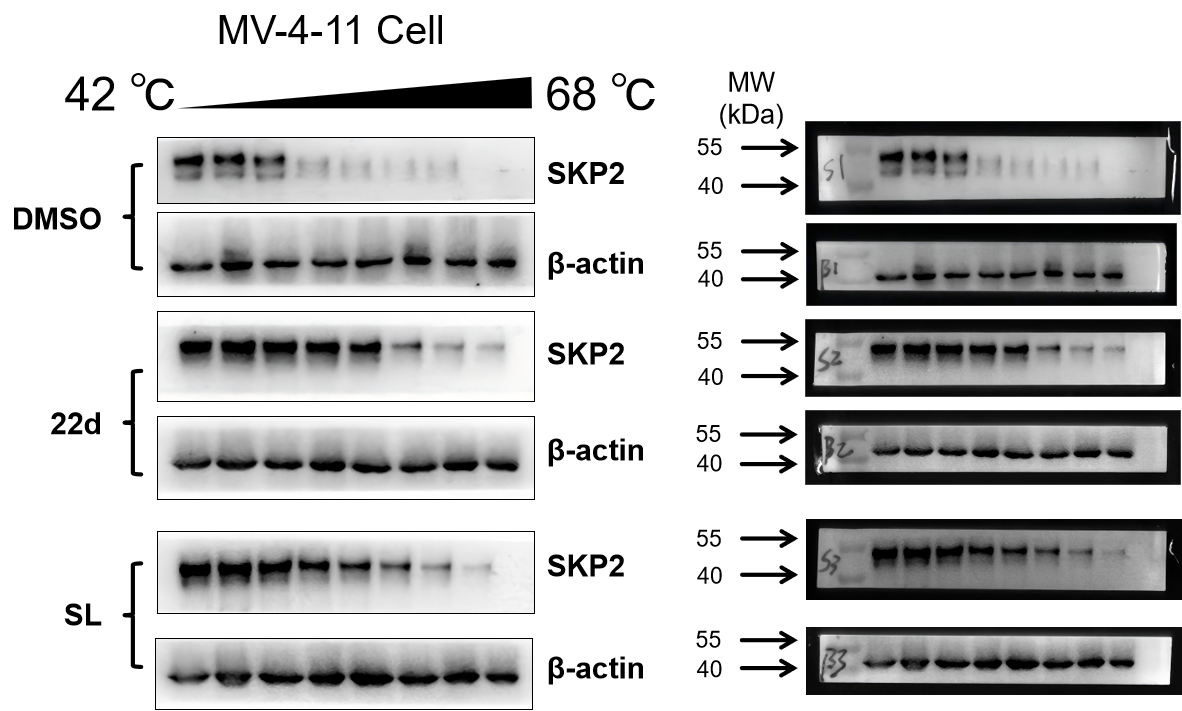
**

**Supplementary Figure 14.** Three replicates and original bands of Figure 2E (b). The bands highlighted in red are shown in the main figure.


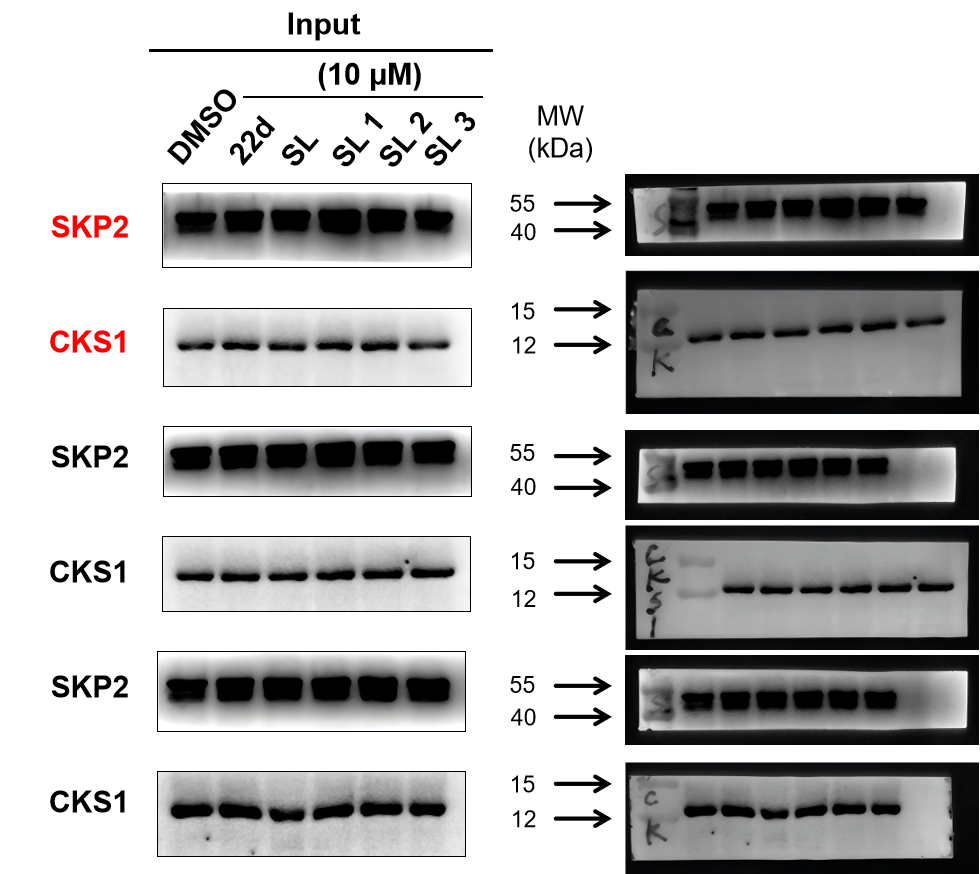


**Supplementary Figure 15.** Three replicates and original bands of Figure 2C (left panel). The bands highlighted in red are shown in the main figure.


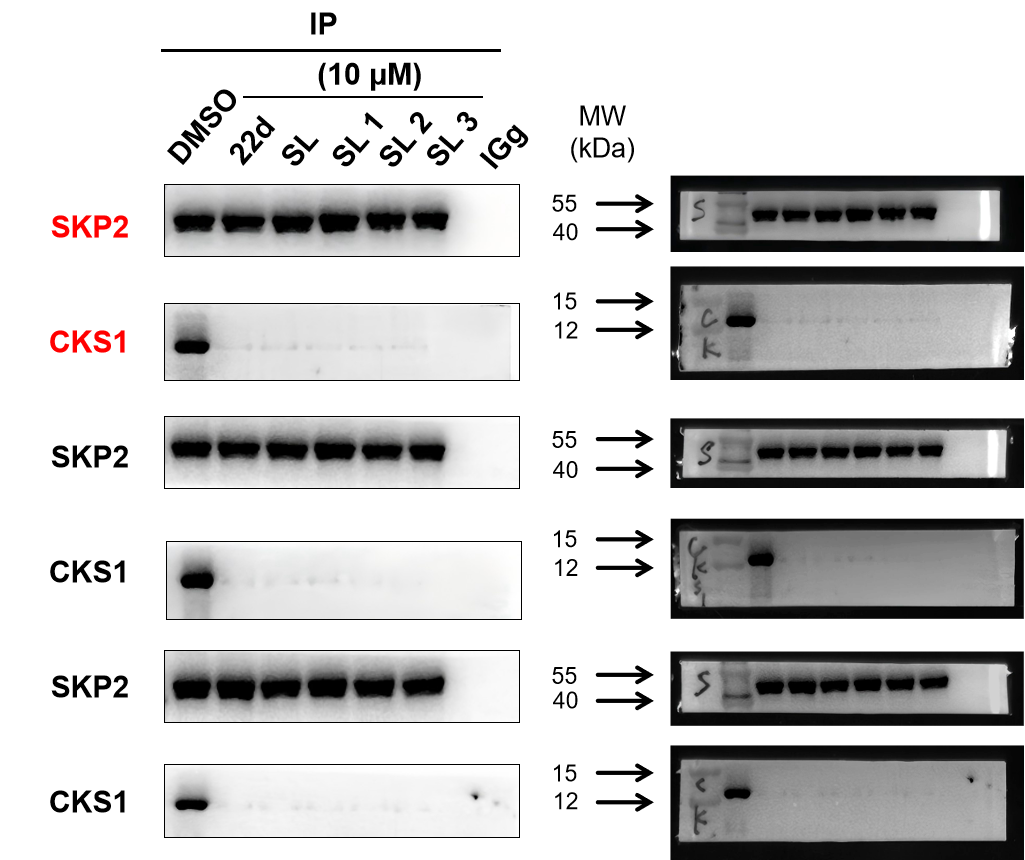


**Supplementary Figure 16.** Three replicates and original bands of Figure 2C (right panel). The bands highlighted in red are shown in the main figure.


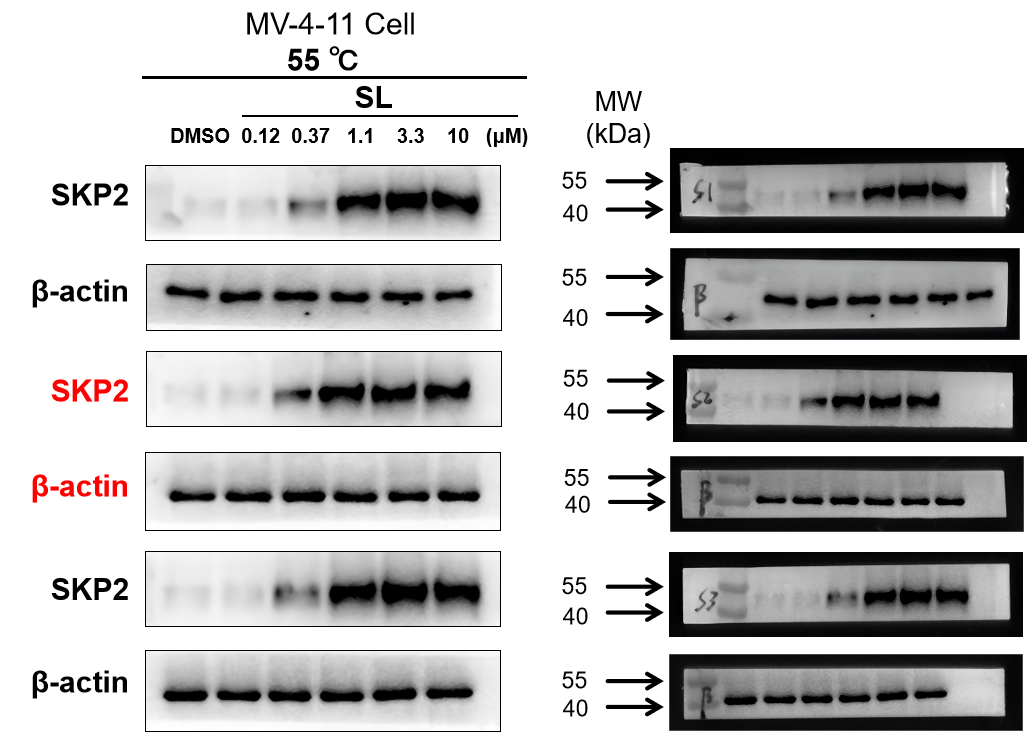


**Supplementary Figure 17.** Three replicates and original bands of Figure 2G. The bands highlighted in red are shown in the main figure.


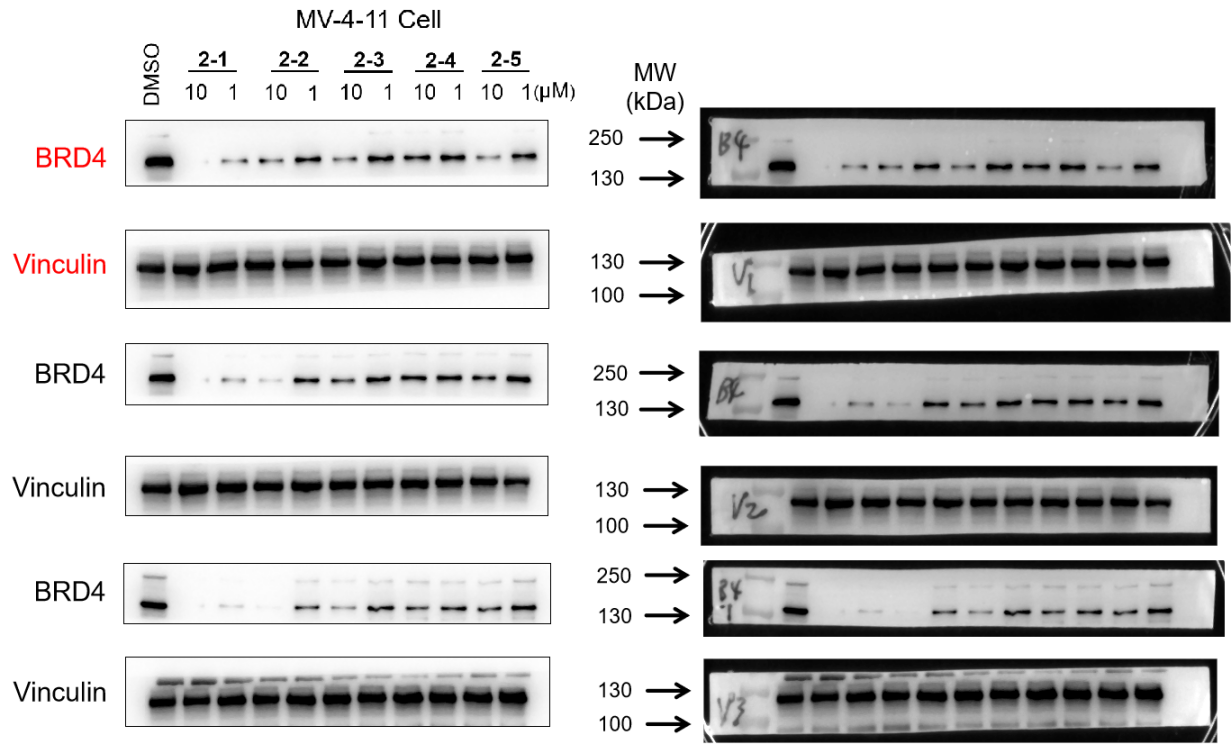


**Supplementary Figure 18.** Three replicates and original bands of Figure 4A. The bands highlighted in red are shown in the main figure.


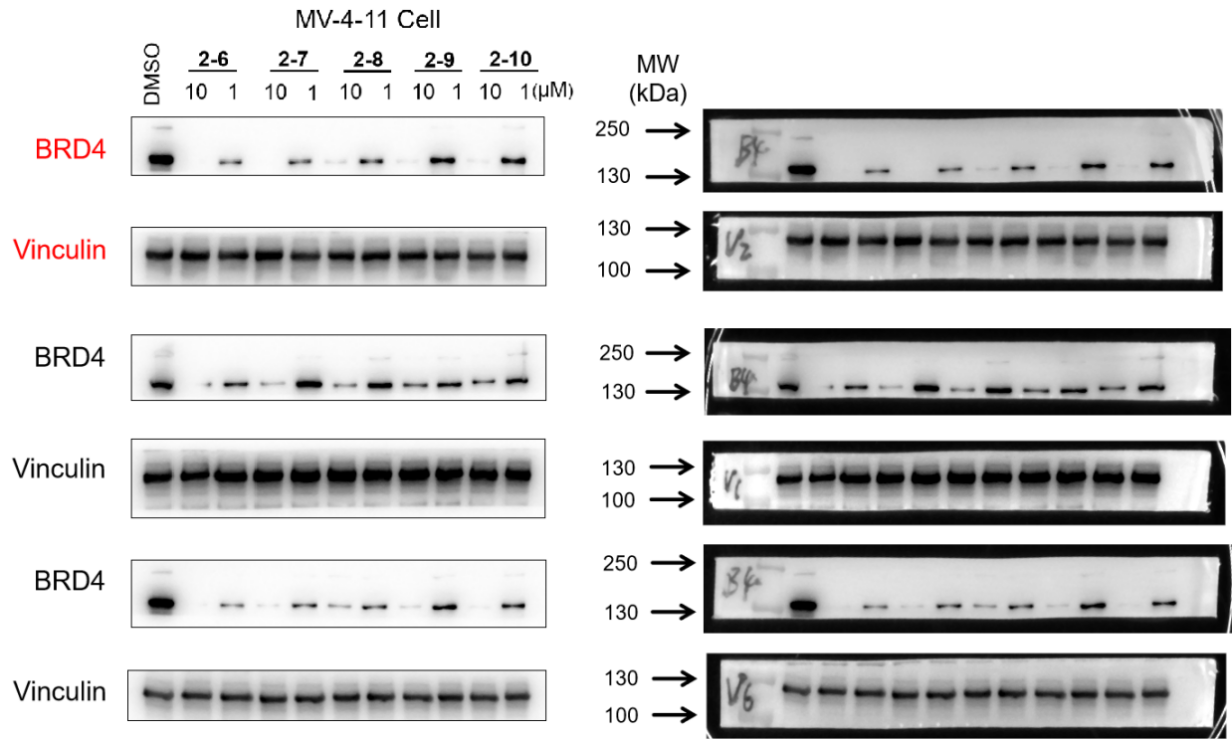


**Supplementary Figure 19.** Three replicates and original bands of Figure 4C. The bands highlighted in red are shown in the main figure.


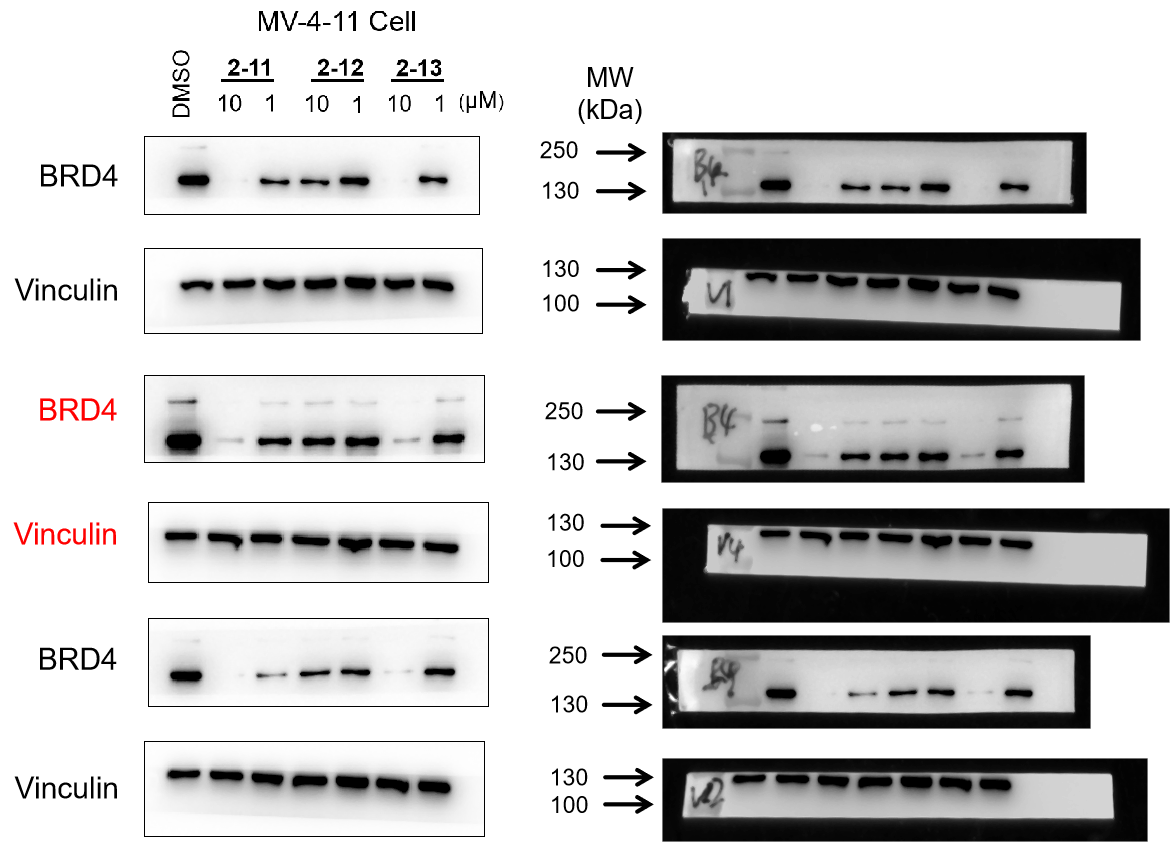


**Supplementary Figure 20.** Three replicates and original bands of Figure 4E. The bands highlighted in red are shown in the main figure.


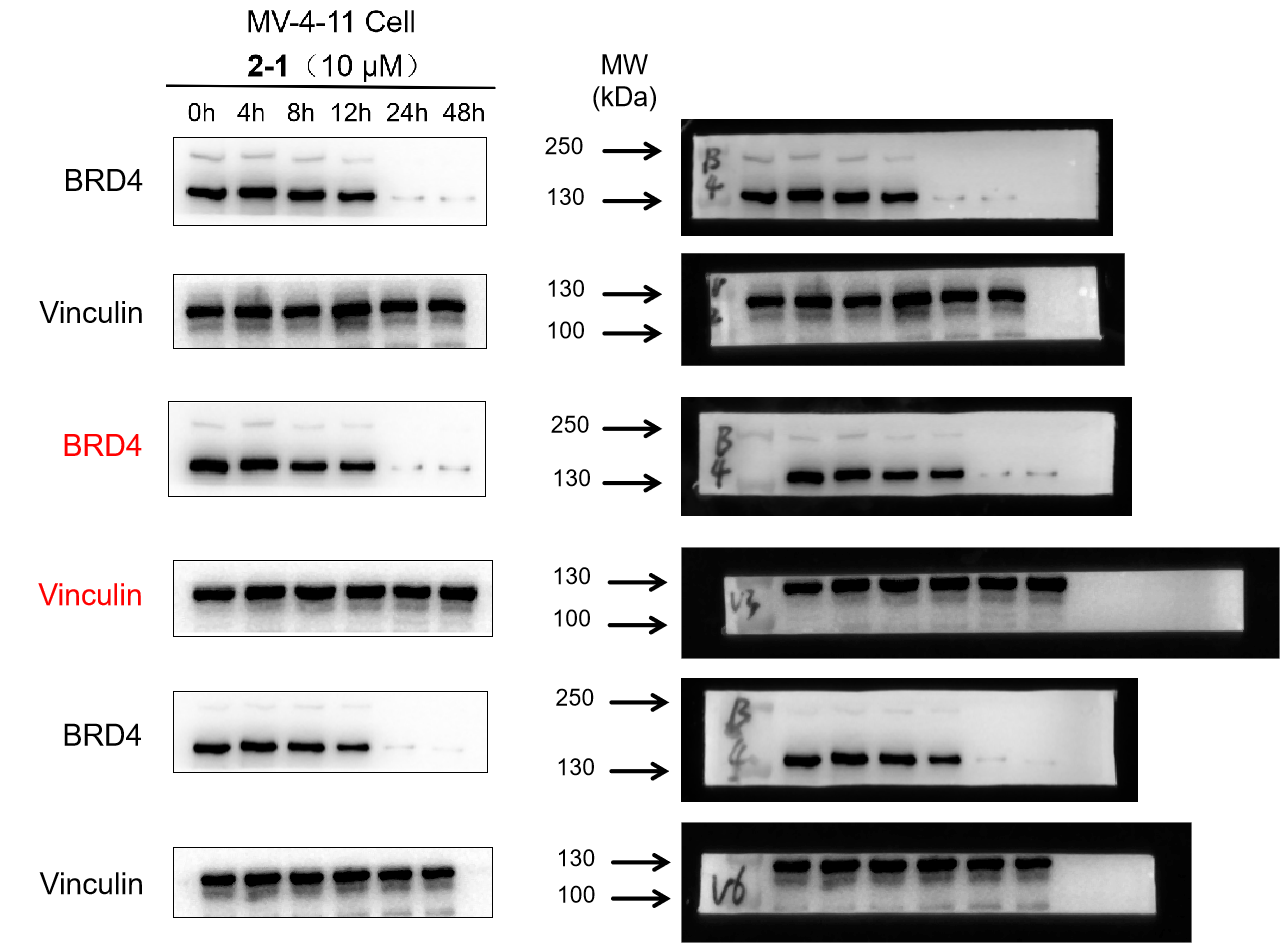


**Supplementary Figure 21.** Three replicates and original bands of Figure 4I. The bands highlighted in red are shown in the main figure.


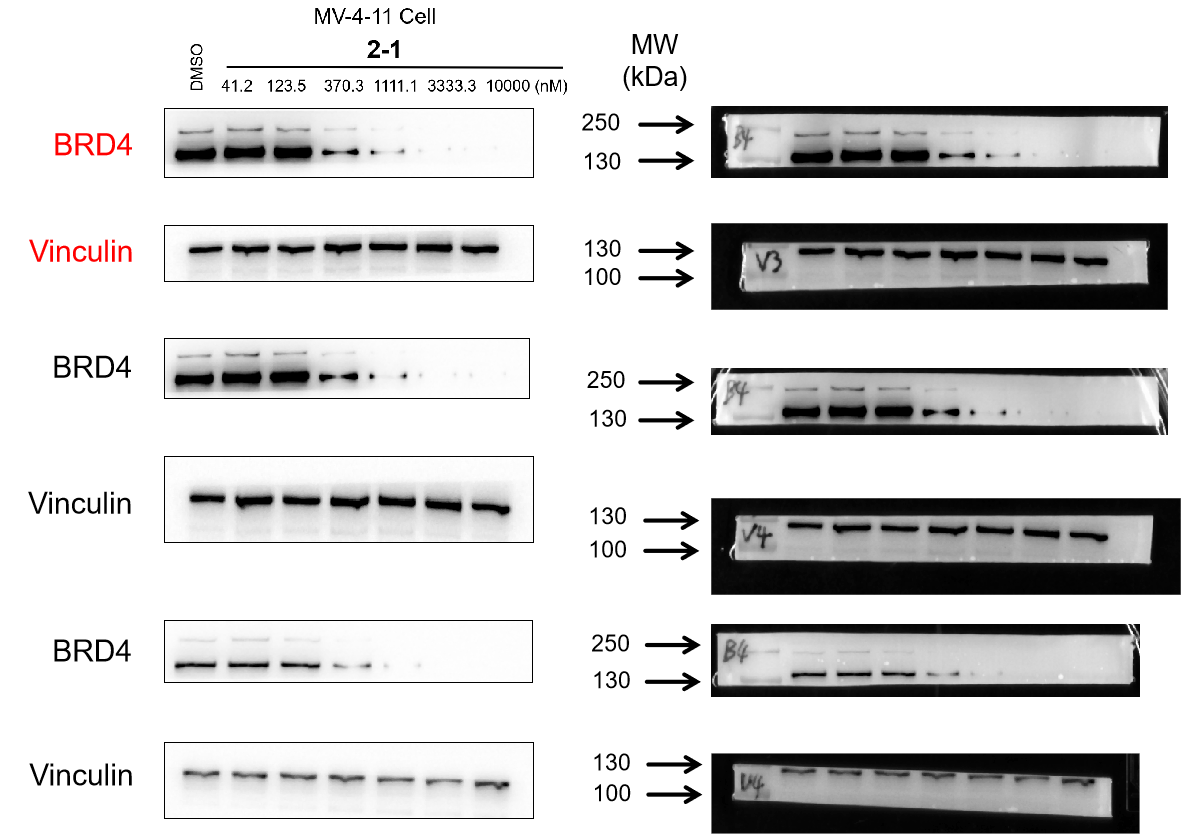


**Supplementary Figure 22.** Three replicates and original bands of Figure 4K. The bands highlighted in red are shown in the main figure.


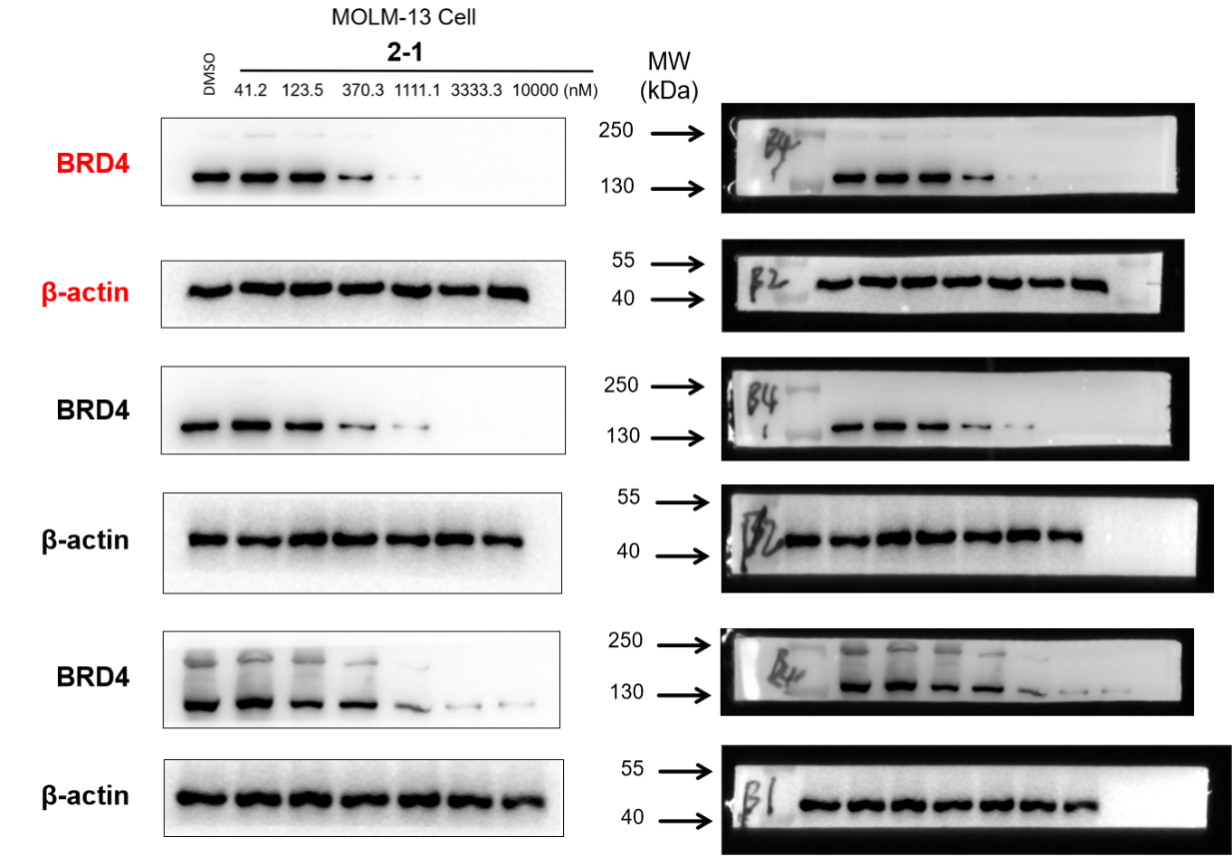


**Supplementary Figure 23.** Three replicates and original bands of Figure 4N. The bands highlighted in red are shown in the main figure.


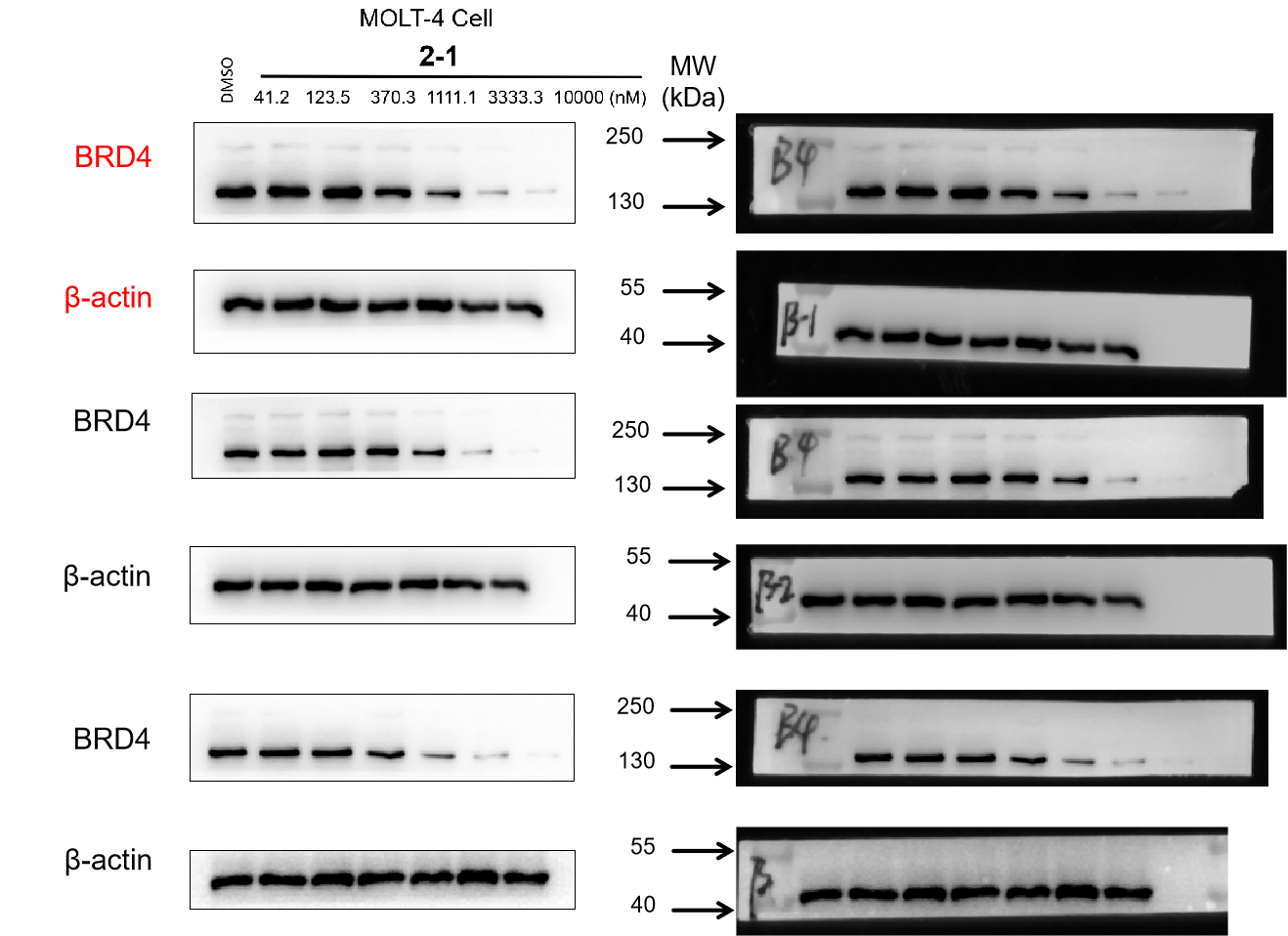


**Supplementary Figure 24.** Three replicates and original bands of Figure 4M. The bands highlighted in red are shown in the main figure.


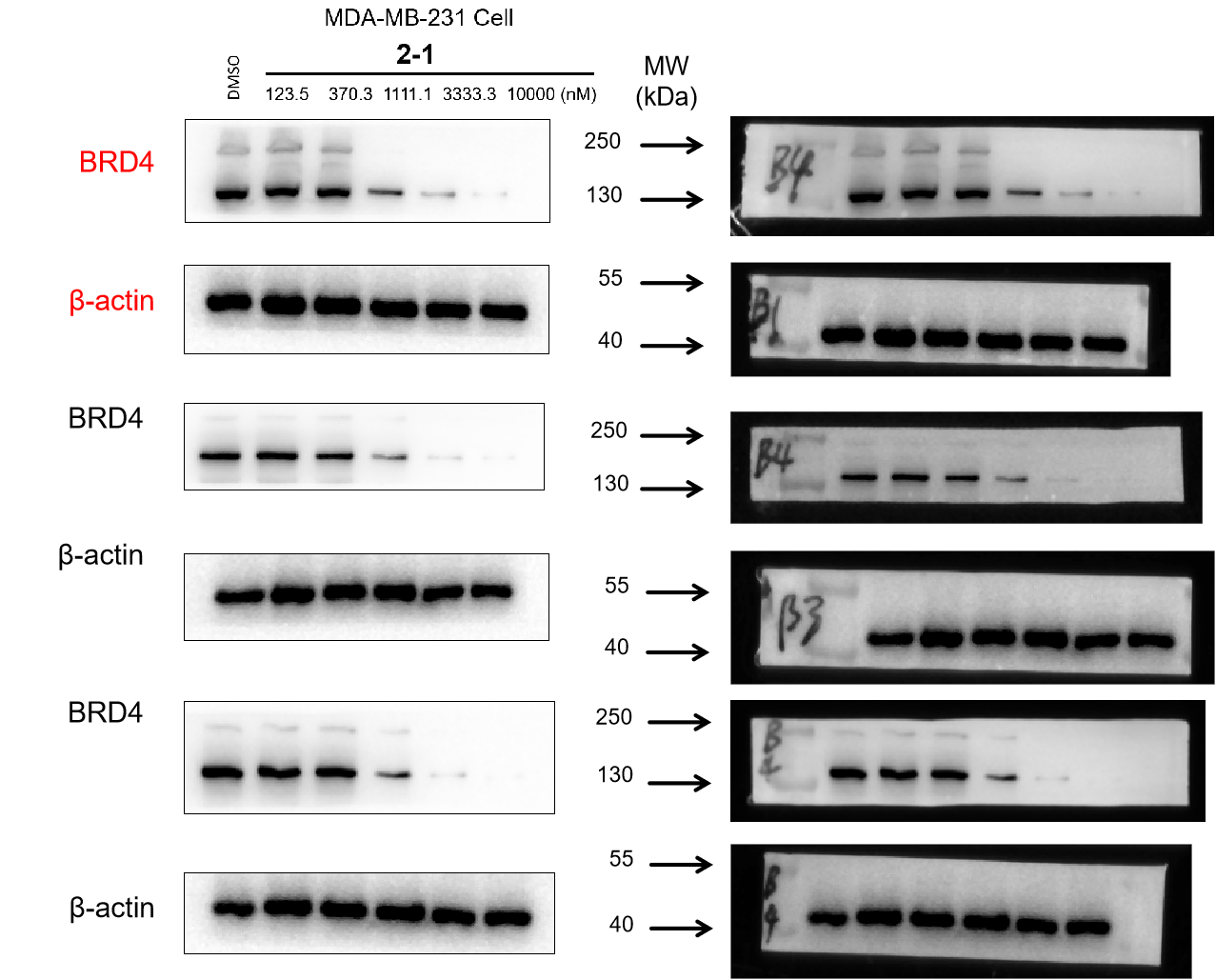


**Supplementary Figure 25.** Three replicates and original bands of Figure 4L. The bands highlighted in red are shown in the main figure.


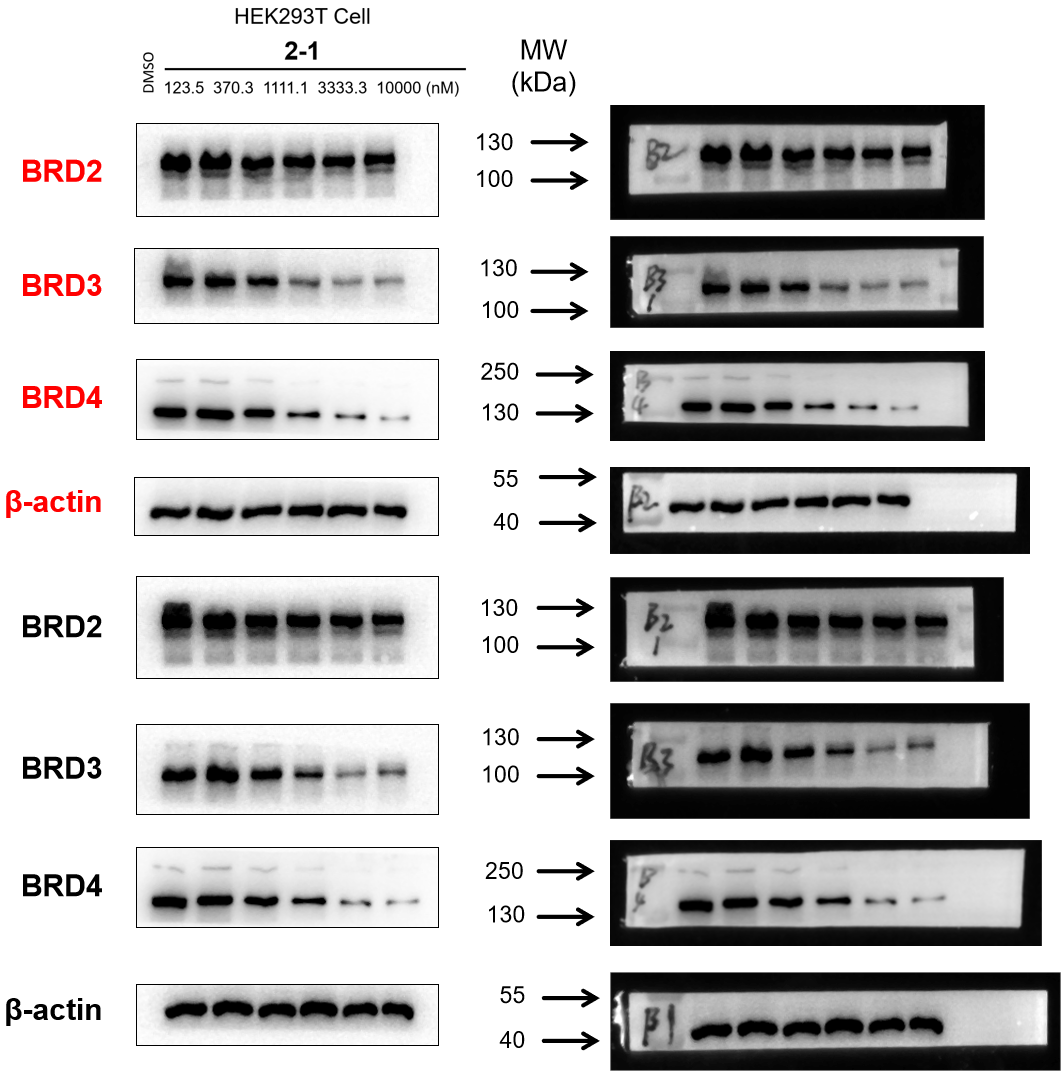


**Supplementary Figure 26.** replicates and original bands of Figure 5A (a). The bands highlighted in red are shown in the main figure.


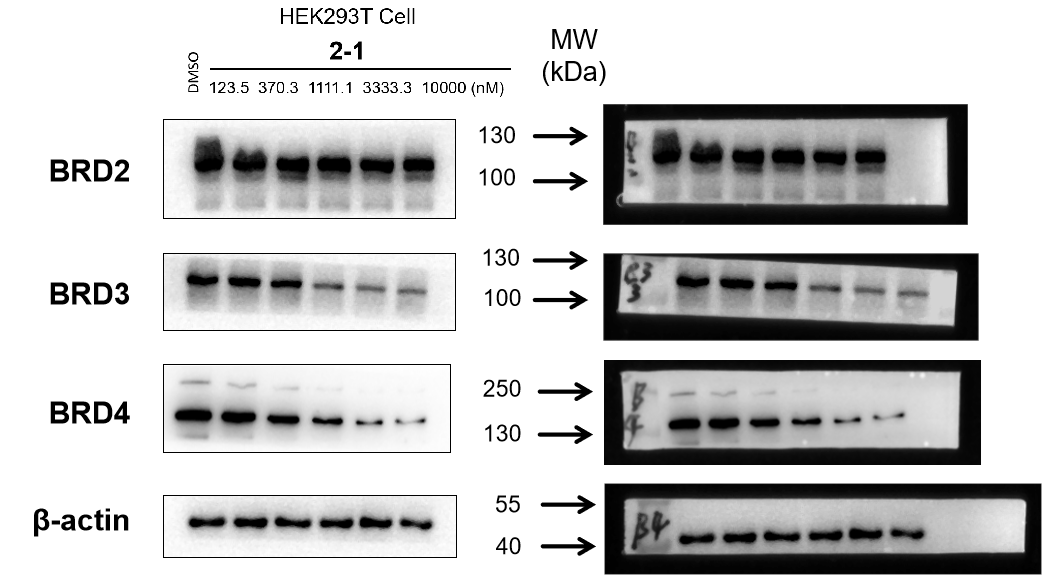


**Supplementary Figure 27.** Three replicates and original bands of Figure 5A (b). The bands highlighted in red are shown in the main figure.


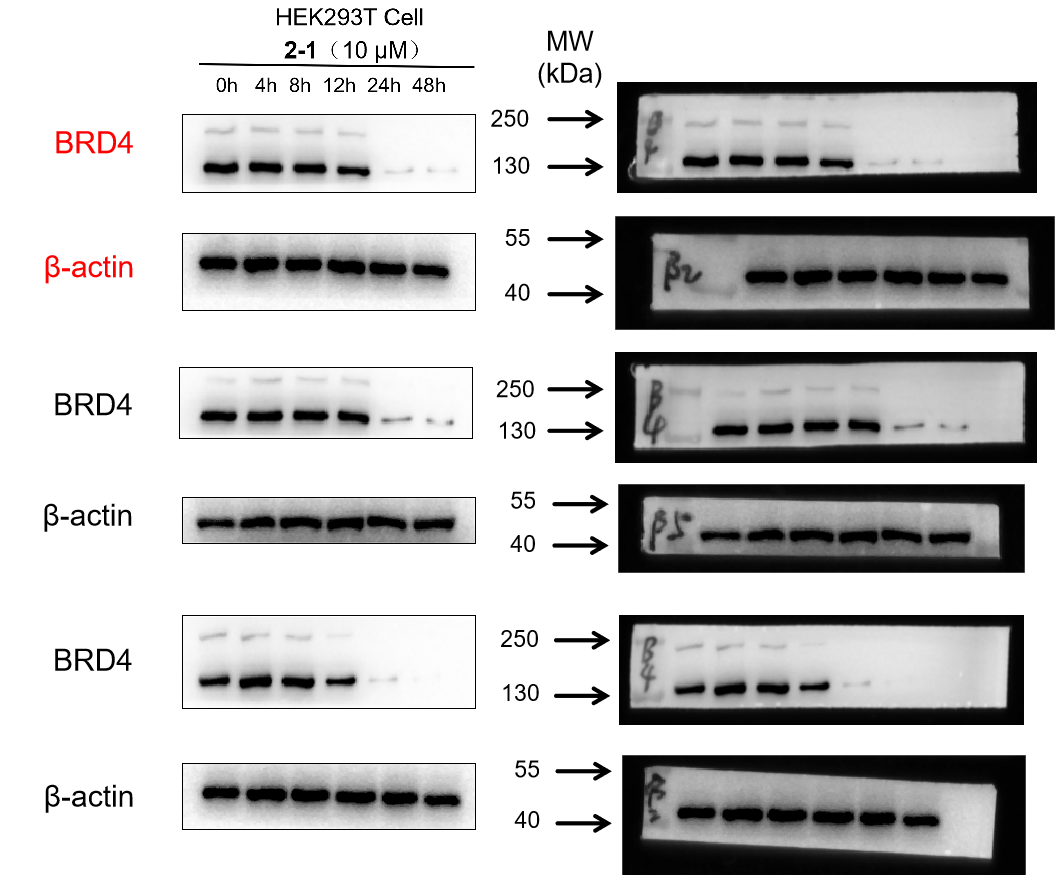


**Supplementary Figure 28.** Three replicates and original bands of Figure 5C. The bands highlighted in red are shown in the main figure.


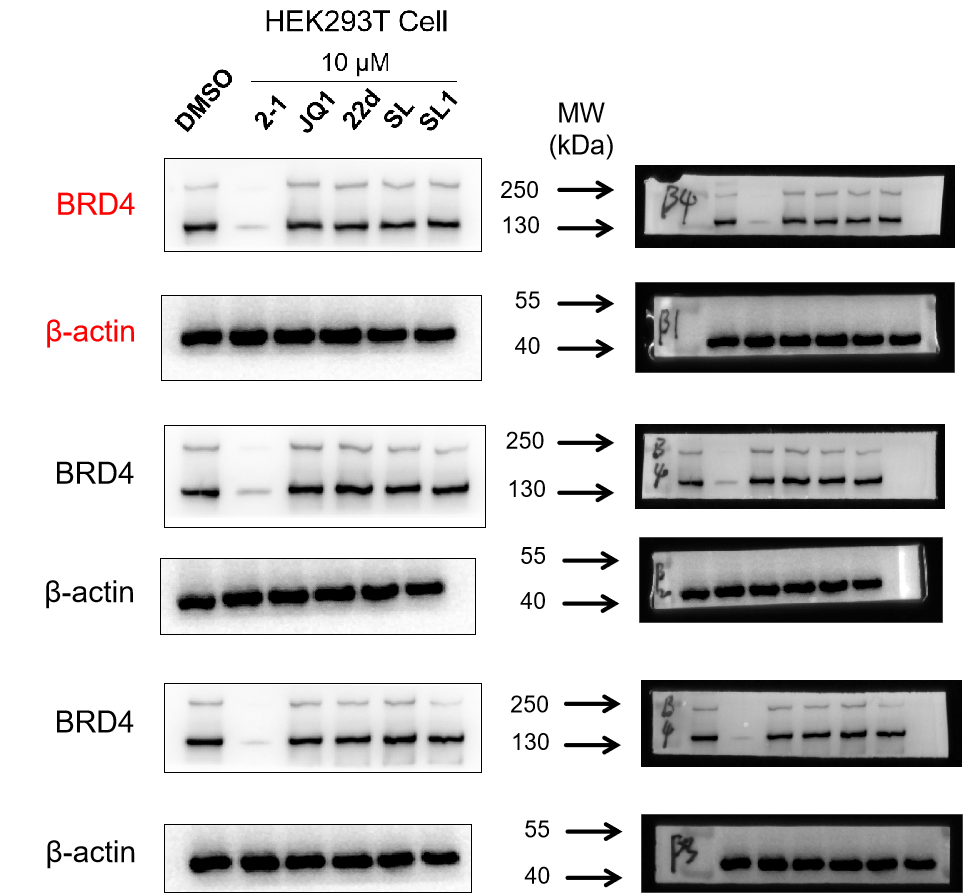


**Supplementary Figure 29.** Three replicates and original bands of Figure 5E. The bands highlighted in red are shown in the main figure.


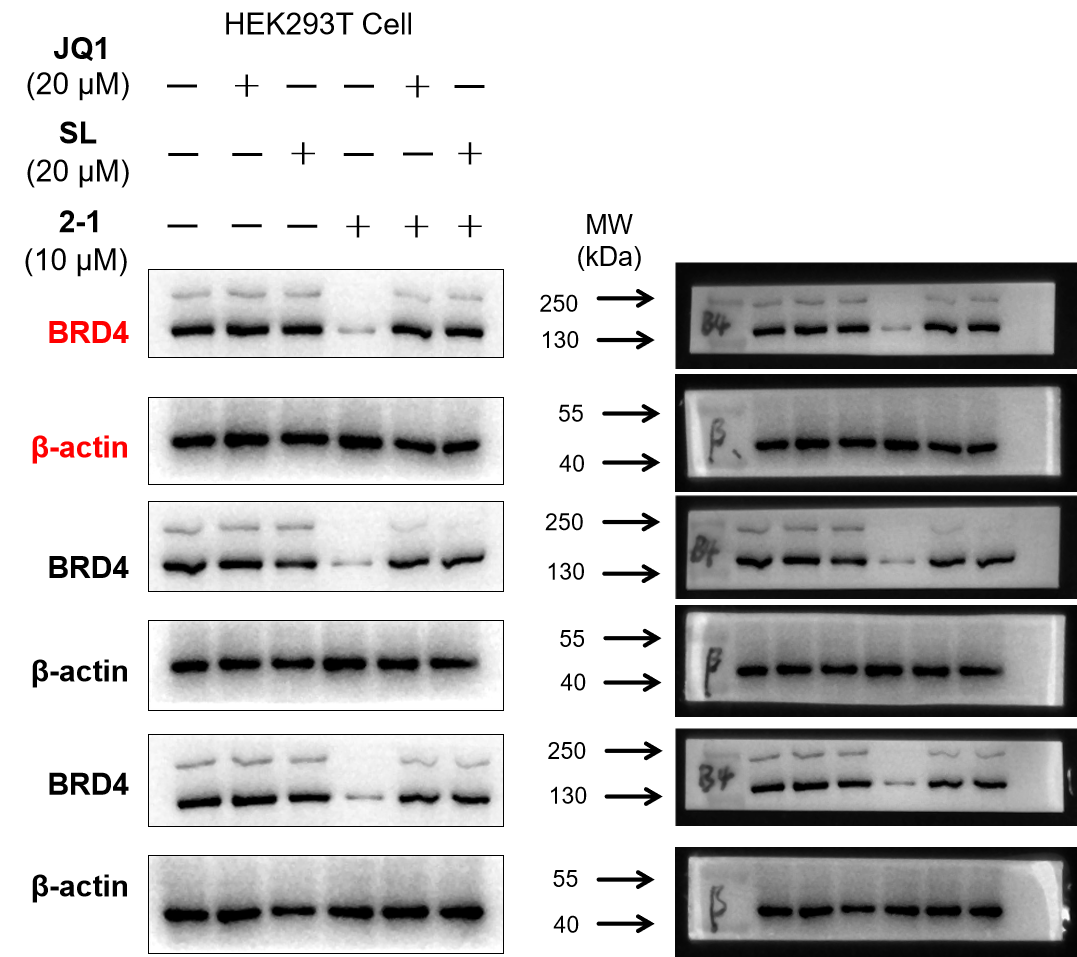


**Supplementary Figure 30.** Three replicates and original bands of Figure 6A. The bands highlighted in red are shown in the main figure.


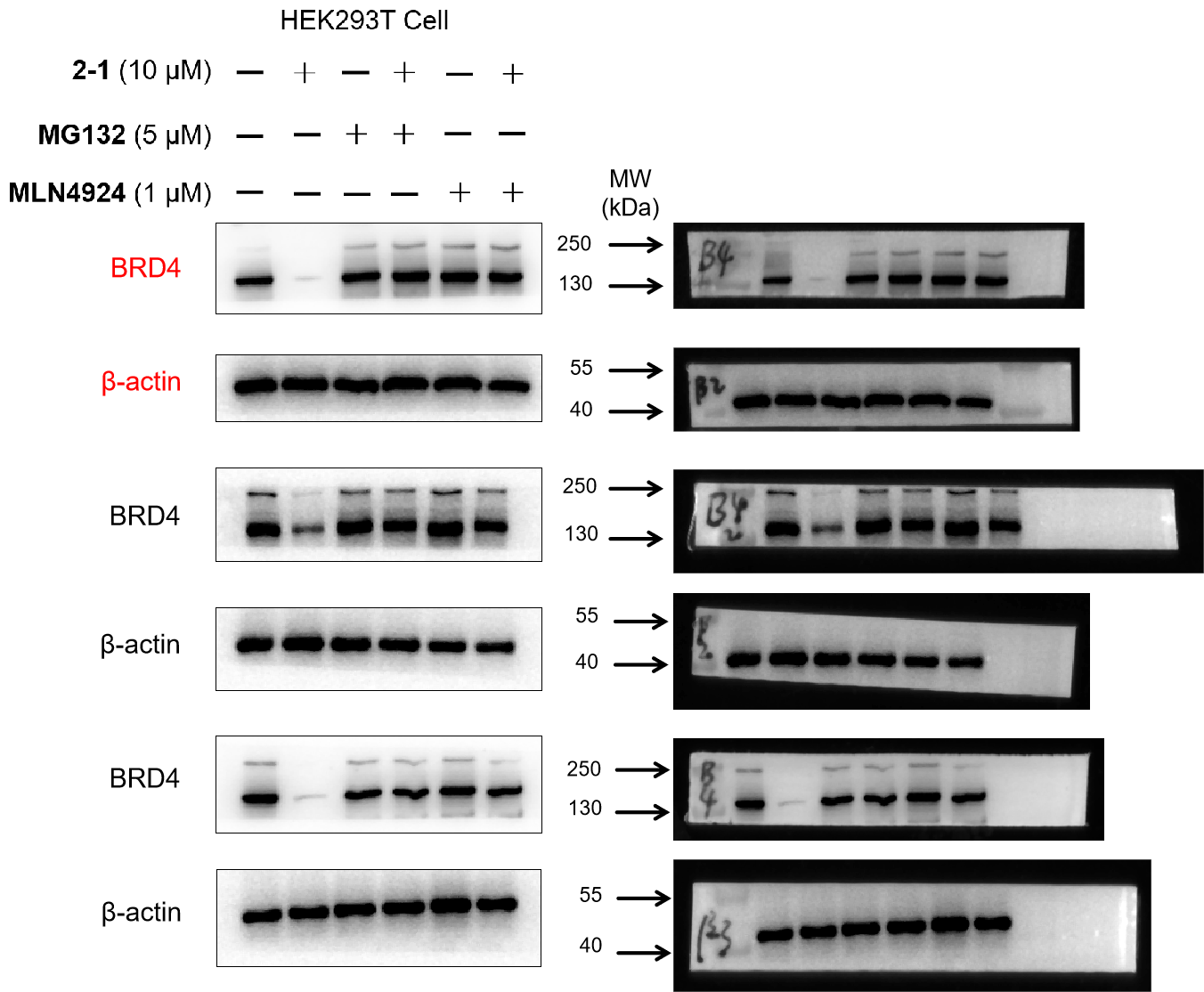


**Supplementary Figure 31.** Three replicates and original bands of Figure 6D. The bands highlighted in red are shown in the main figure.


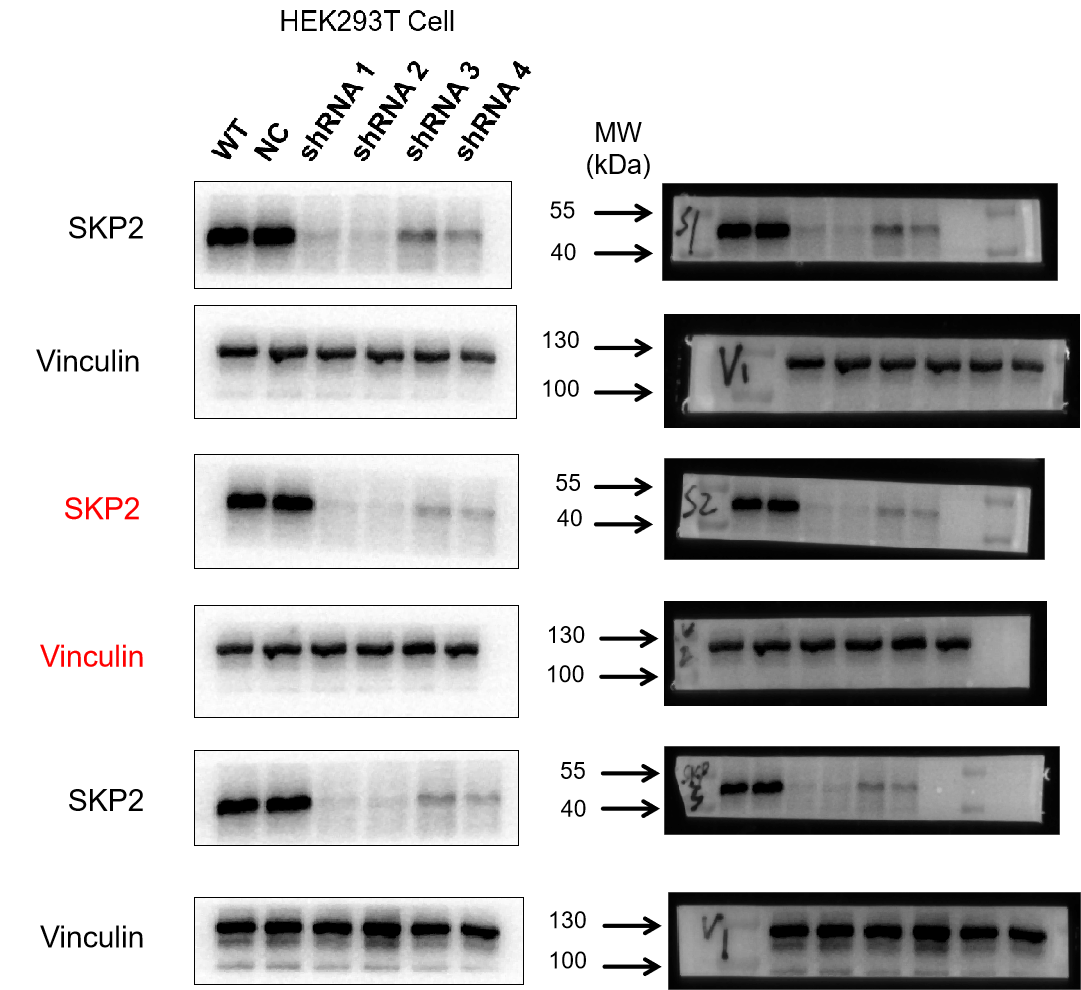


**Supplementary Figure 32.** Three replicates and original bands of Figure 6F. The bands highlighted in red are shown in the main figure.


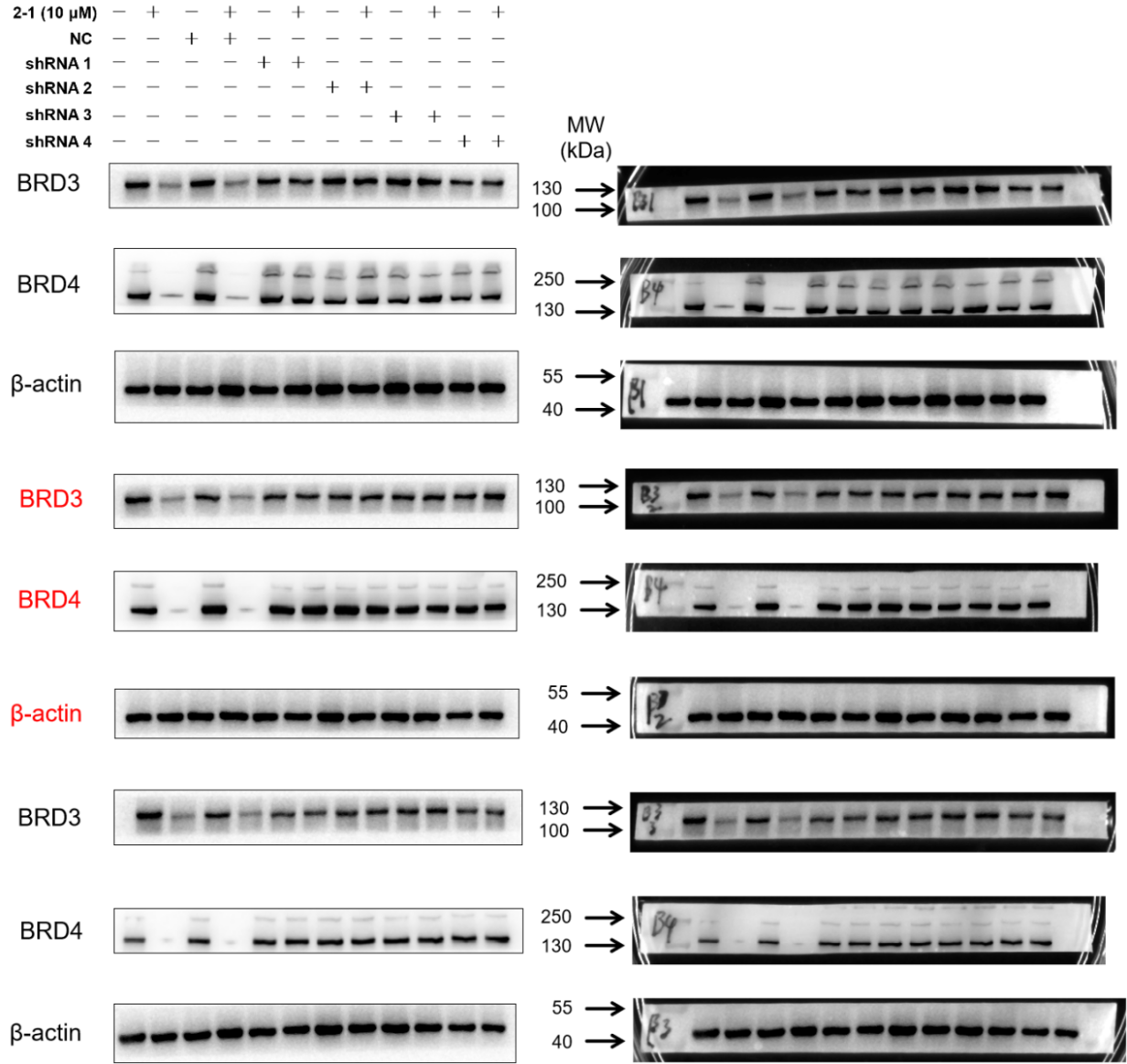


**Supplementary Figure 33.** Three replicates and original bands of Figure 6G. The bands highlighted in red are shown in the main figure.


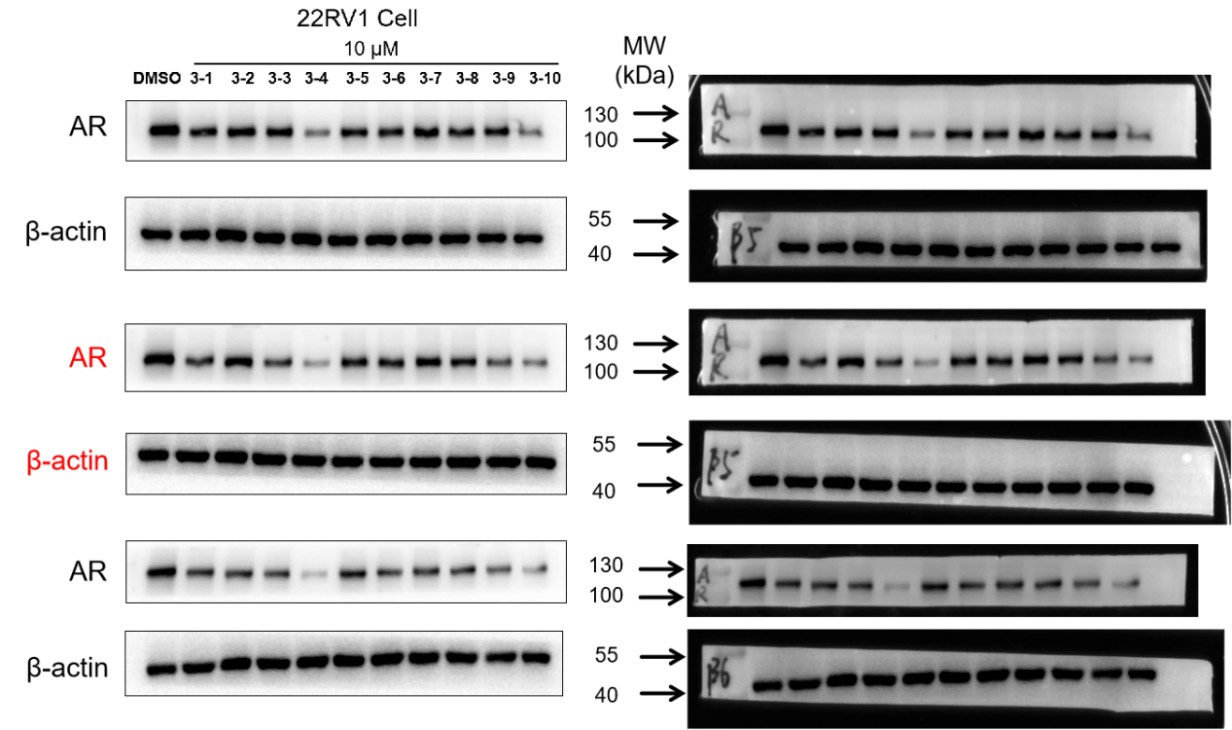


**Supplementary Figure 34.** Three replicates and original bands of Figure 7A. The bands highlighted in red are shown in the main figure.


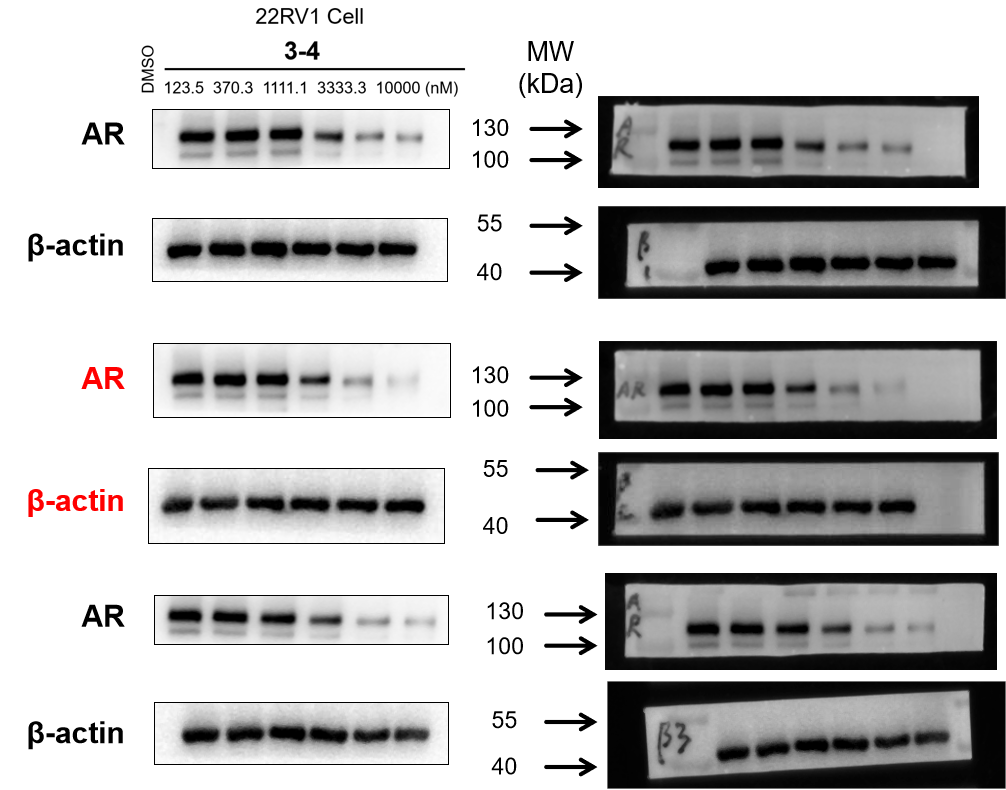


**Supplementary Figure 35.** Three replicates and original bands of Figure 7D. The bands highlighted in red are shown in the main figure.


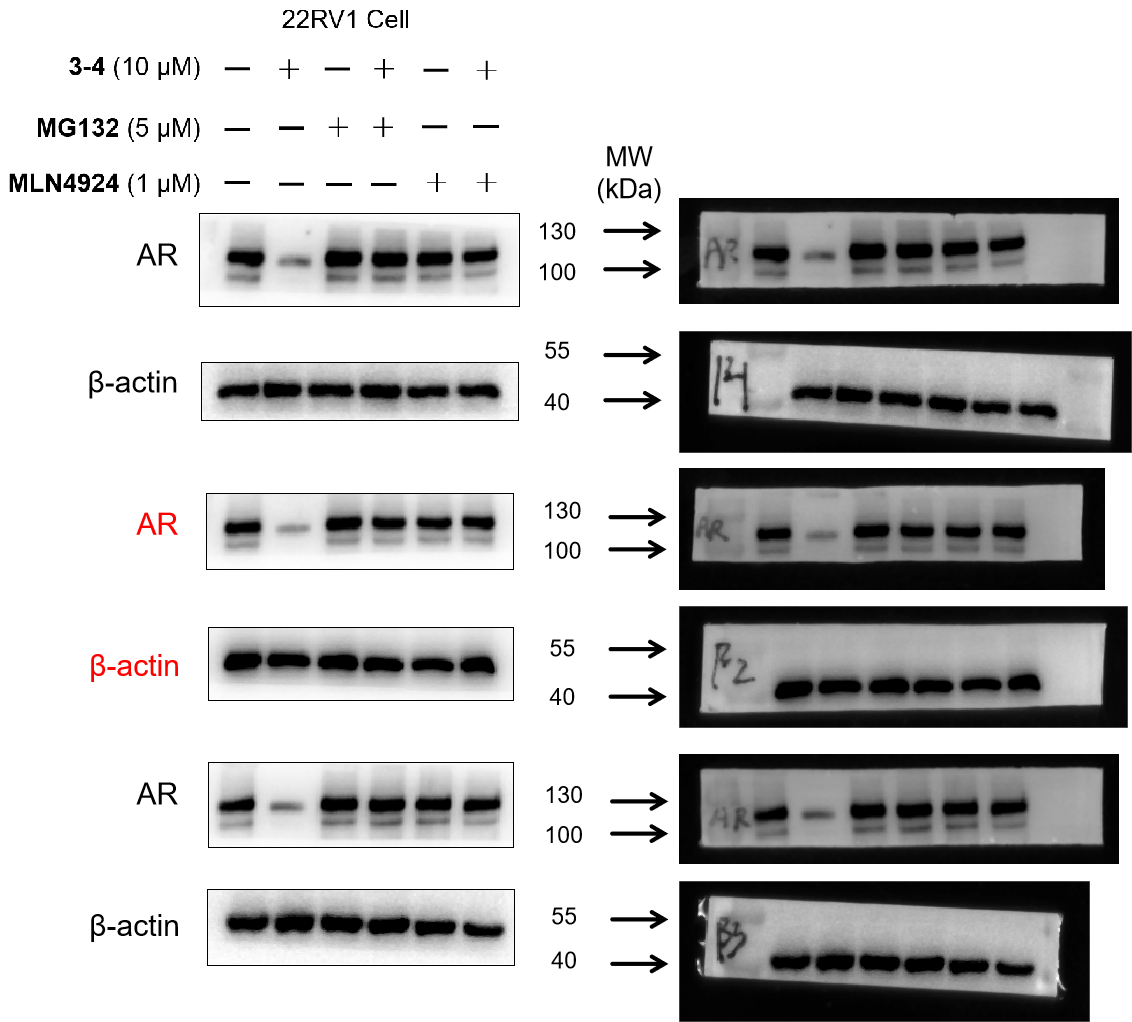


**Supplementary Figure 36.** Three replicates and original bands of Figure 7F. The bands highlighted in red are shown in the main figure.


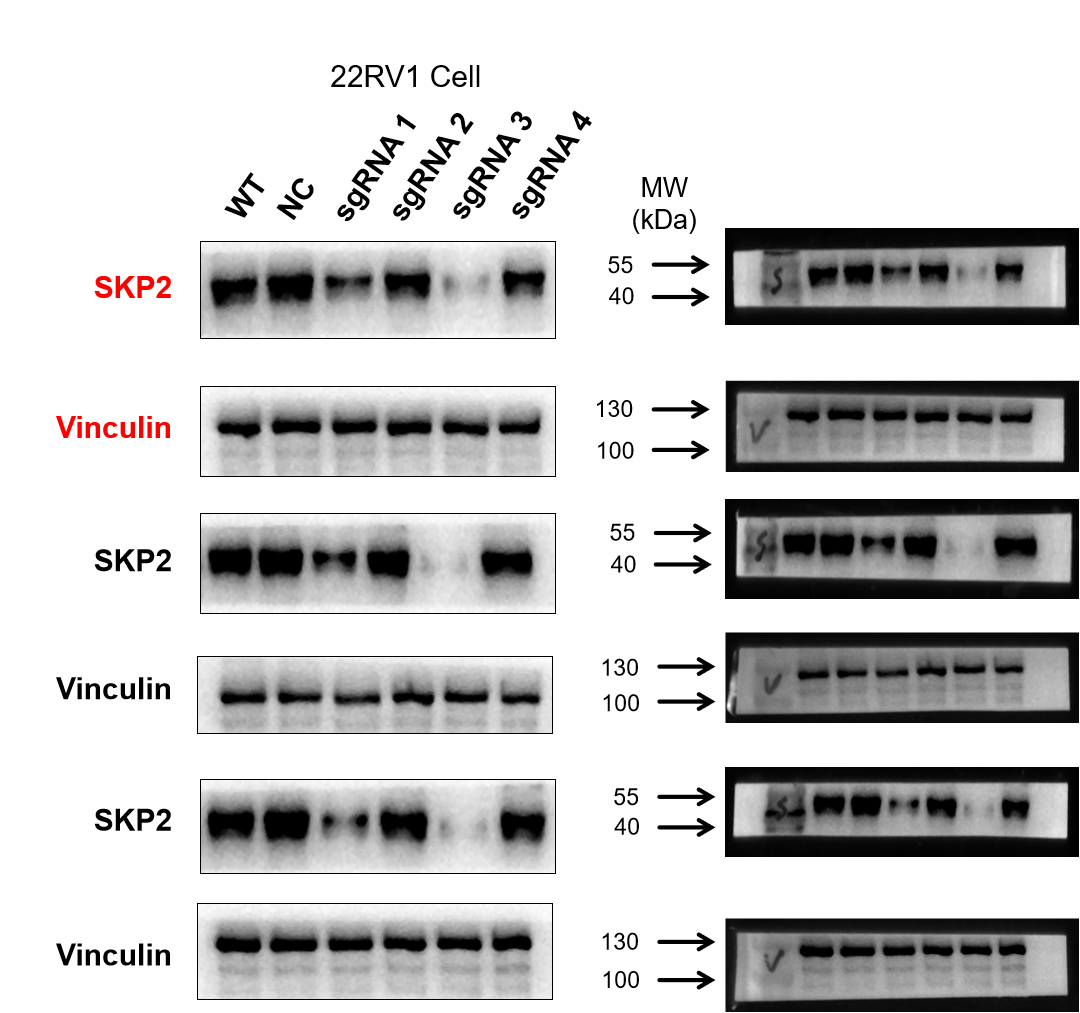


**Supplementary Figure 37.** Three replicates and original bands of Figure 7H. The bands highlighted in red are shown in the main figure.

**
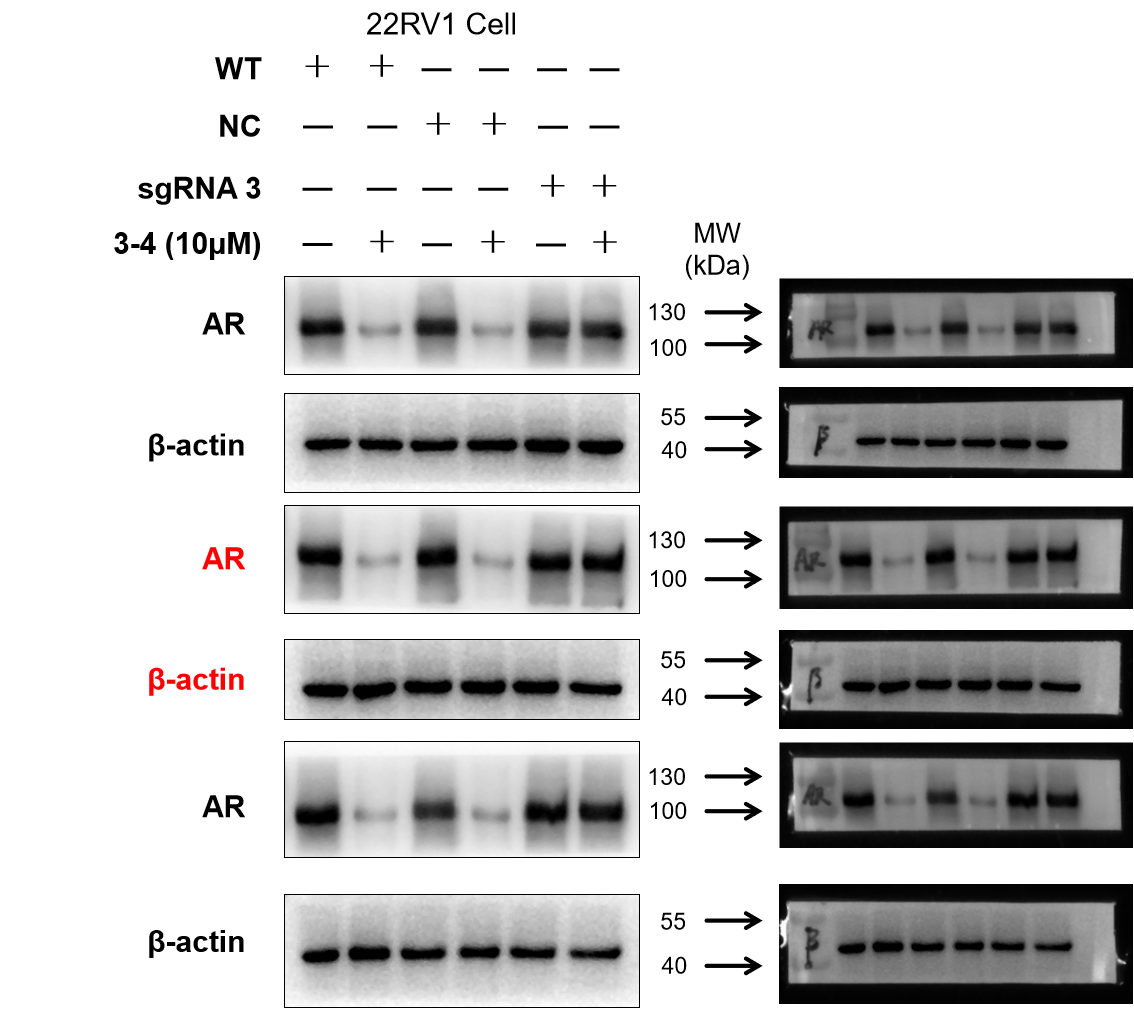
**

**Supplementary Figure 38.** Three replicates and original bands of Figure 7J. The bands highlighted in red are shown in the main figure.


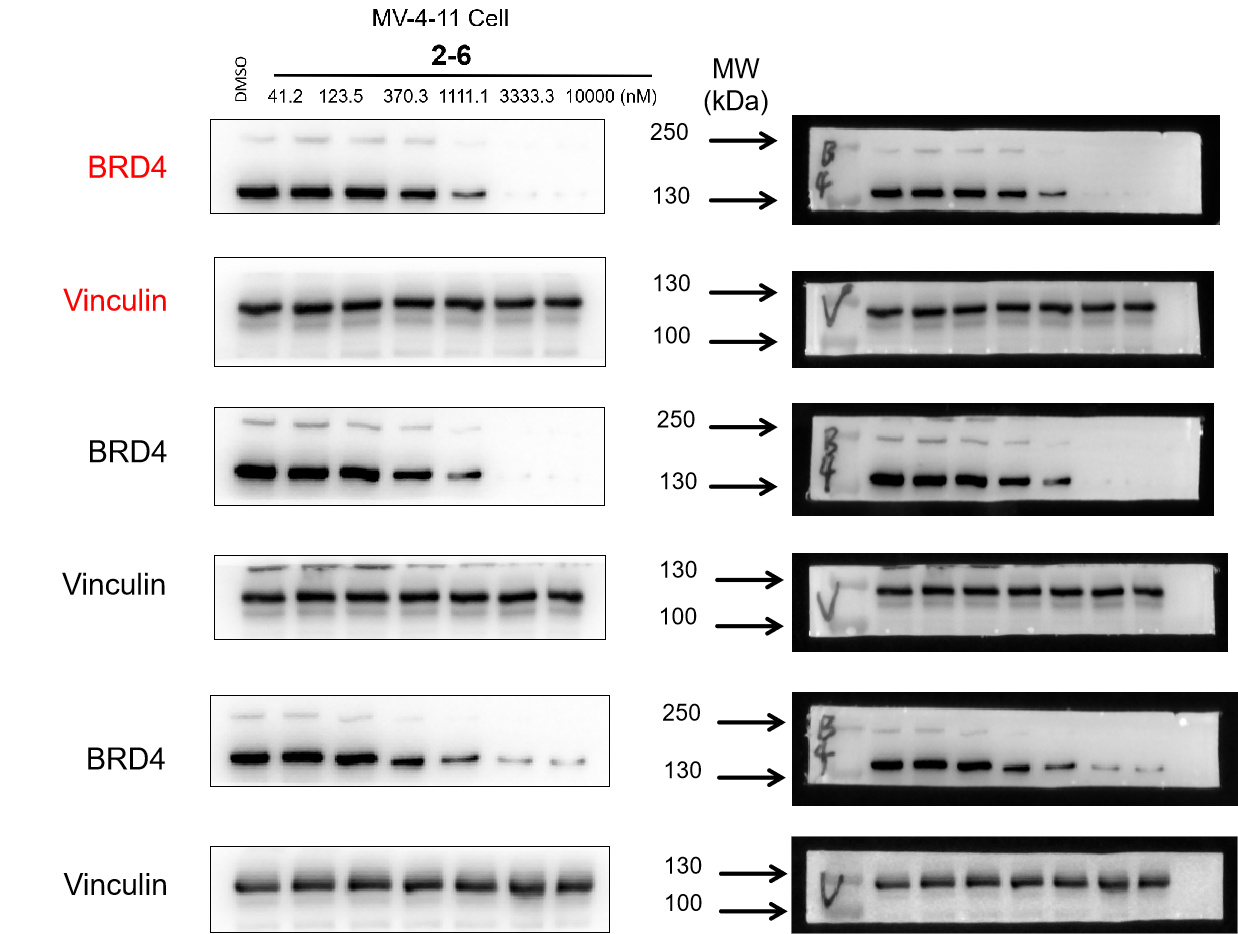


**Supplementary Figure 39.** Three replicates and original bands of Supplementary Figure 5C. The bands highlighted in red are shown in the main figure.


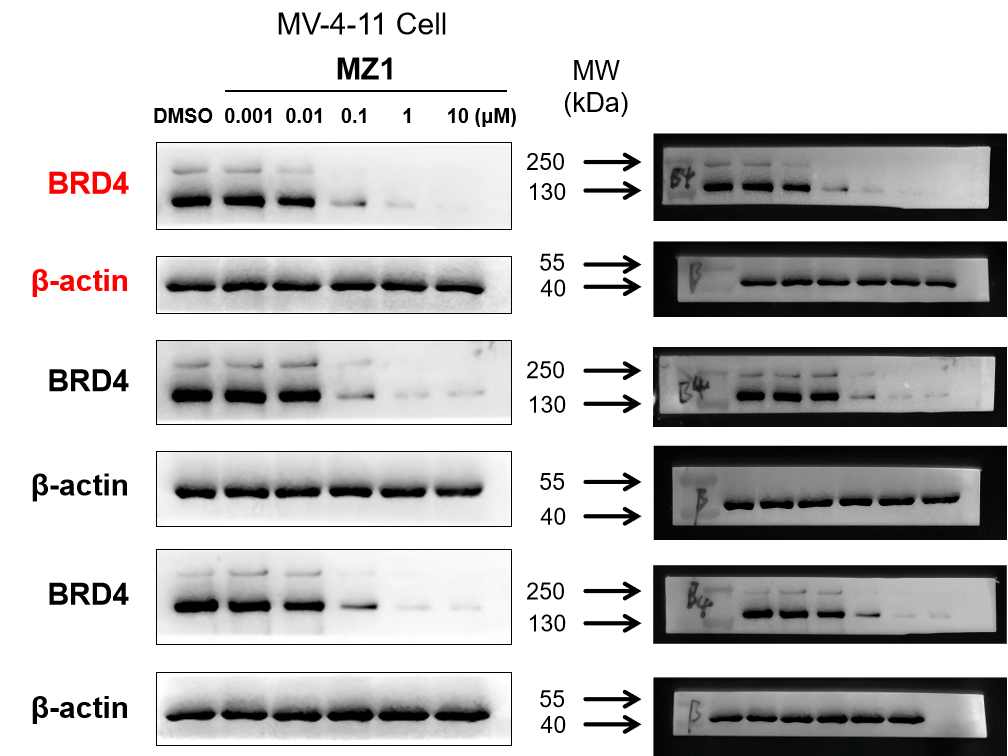


**Supplementary Figure 40.** Three replicates and original bands of Supplementary Figure 6C. The bands highlighted in red are shown in the main figure.


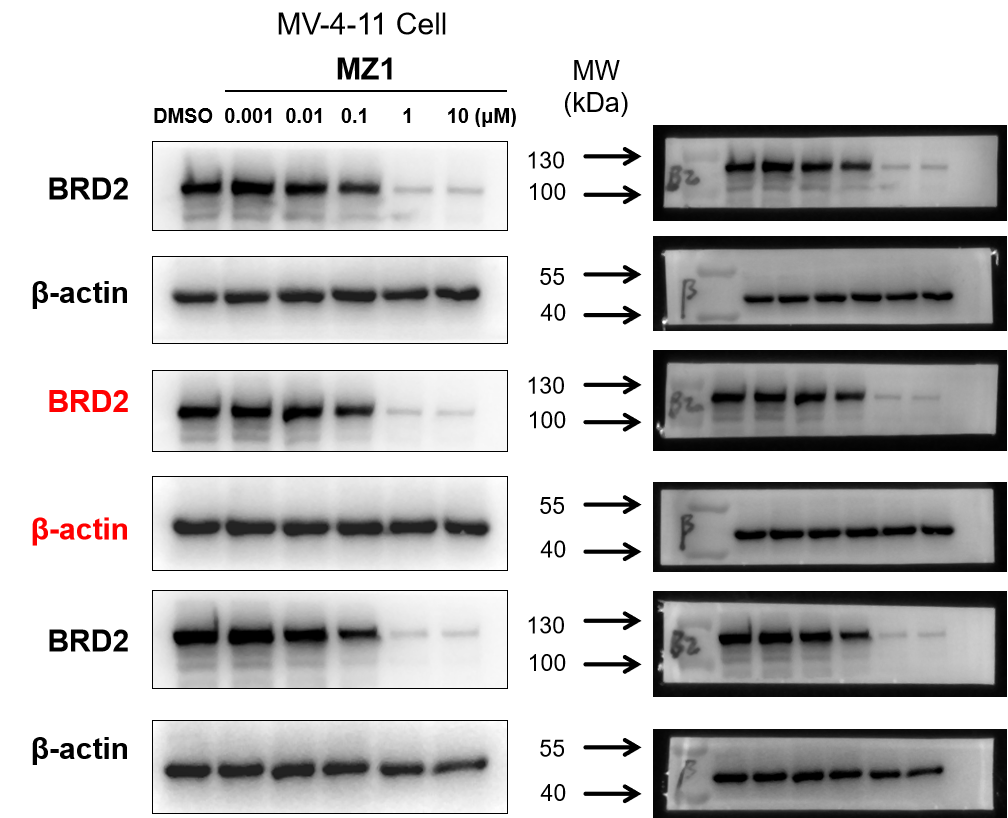


**Supplementary Figure 41.** Three replicates and original bands of Supplementary Figure 6E. The bands highlighted in red are shown in the main figure.


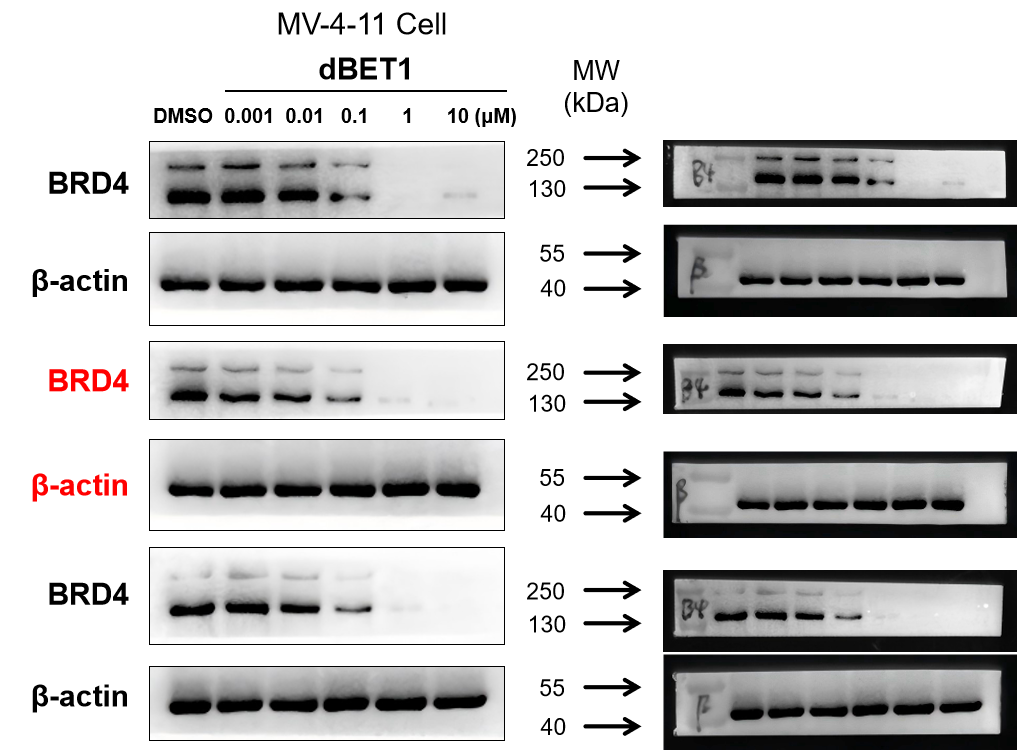


**Supplementary Figure 42.** Three replicates and original bands of Supplementary Figure 6G. The bands highlighted in red are shown in the main figure.


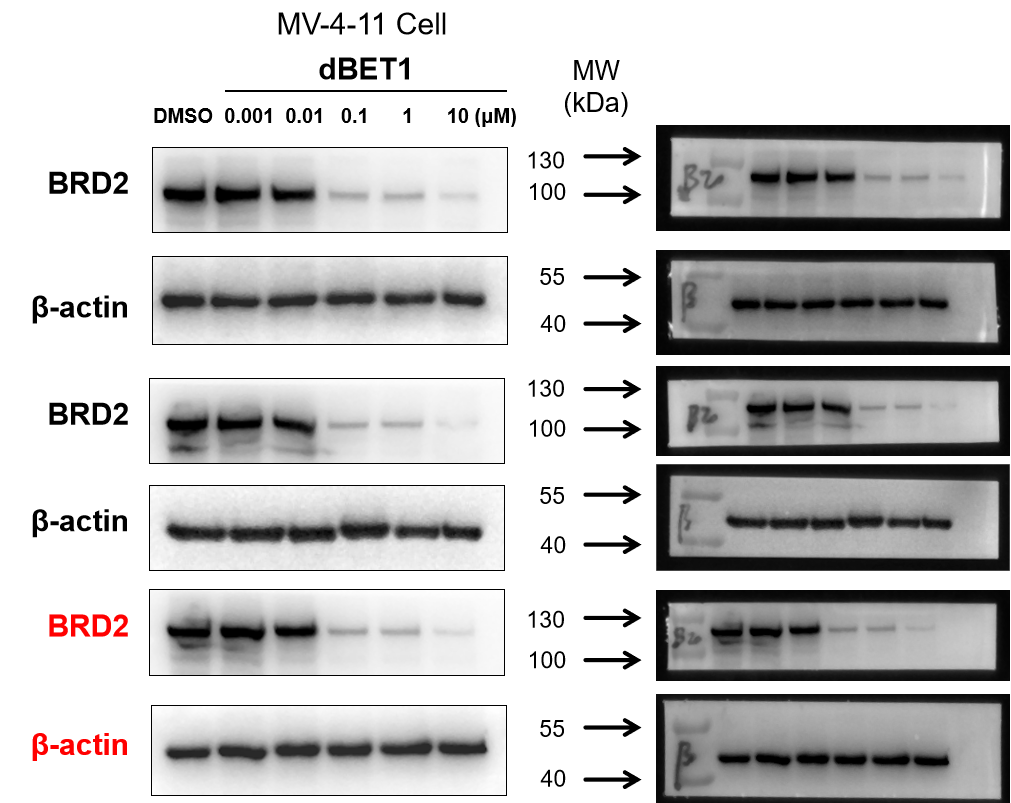


**Supplementary Figure 43.** Three replicates and original bands of Supplementary Figure 6I. The bands highlighted in red are shown in the main figure.


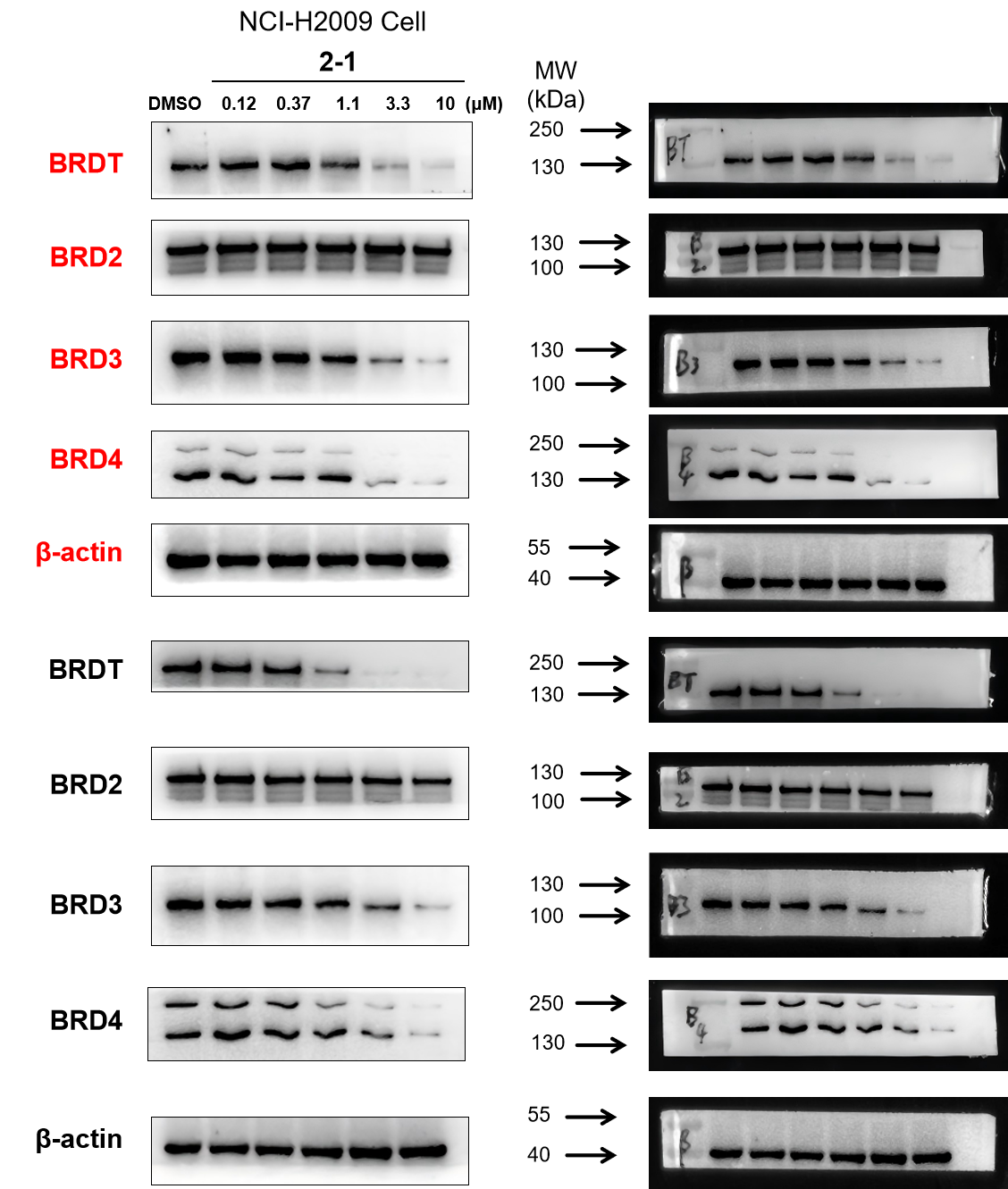


**Supplementary Figure 44.** Three replicates and original bands of Supplementary Figure 7A(a). The bands highlighted in red are shown in the main figure.


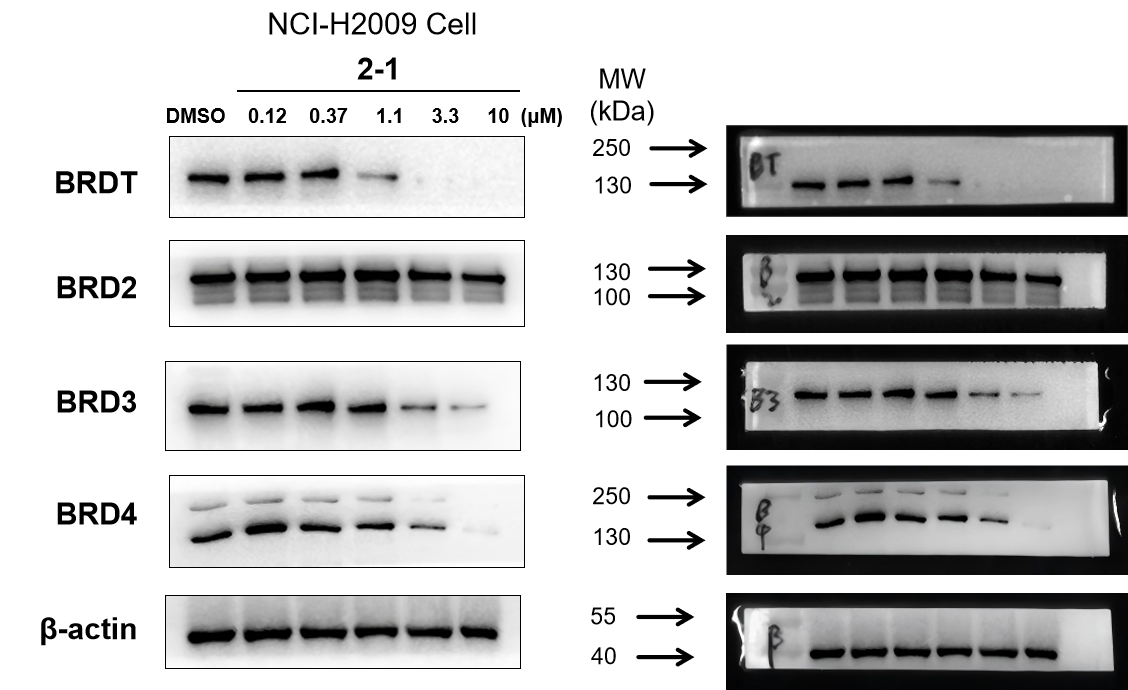


**Supplementary Figure 45.** Three replicates and original bands of Supplementary Figure 7A(b). The bands highlighted in red are shown in the main figure.


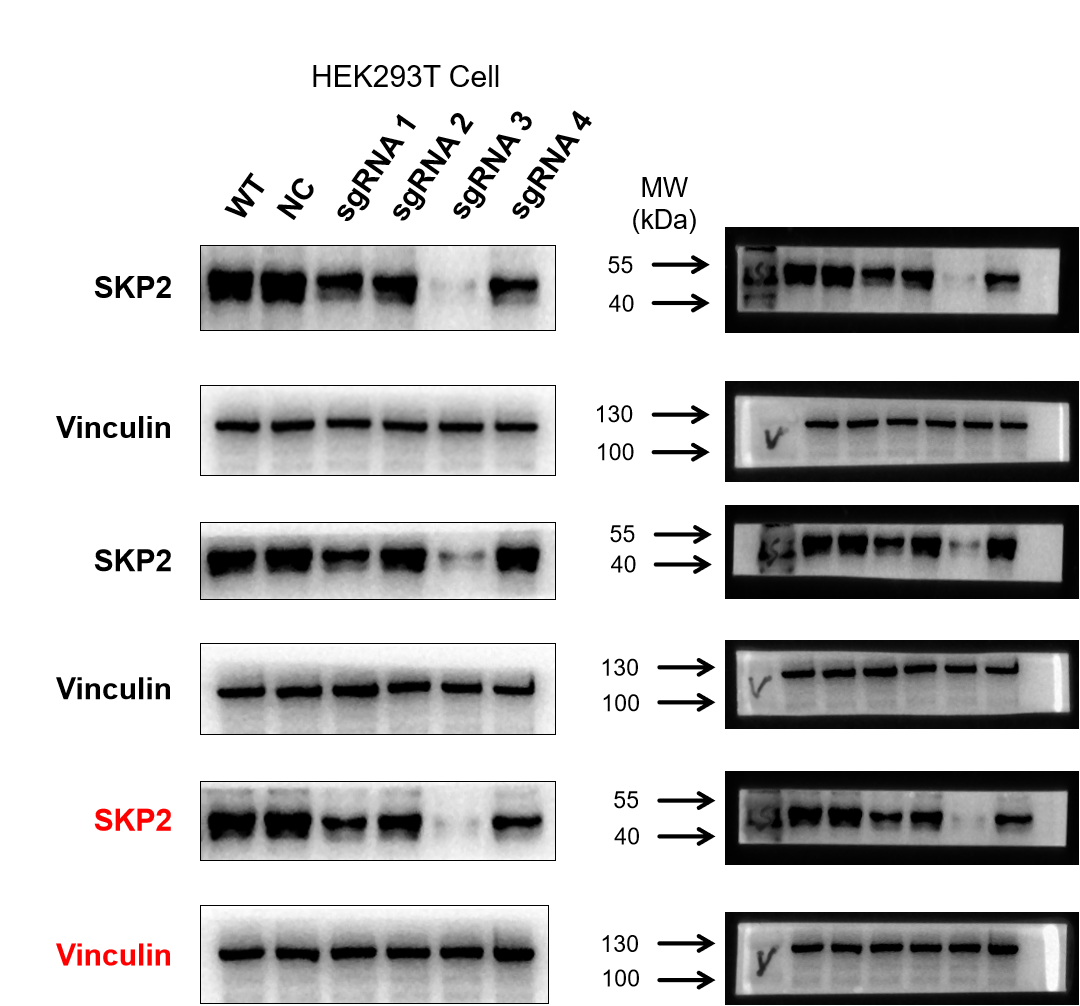


**Supplementary Figure 46.** Three replicates and original bands of Supplementary Figure 8A. The bands highlighted in red are shown in the main figure.


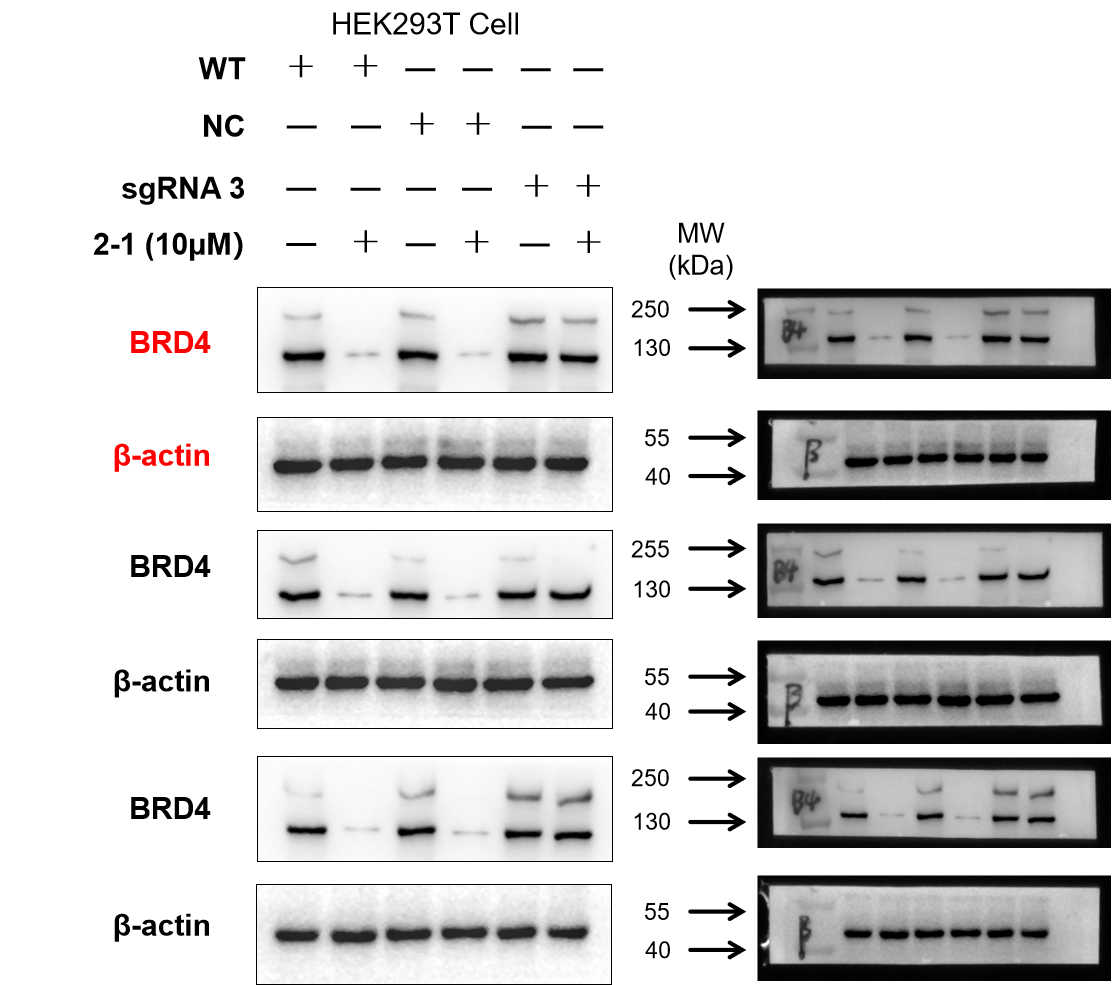


**Supplementary Figure 47.** Three replicates and original bands of Supplementary Figure 8C. The bands highlighted in red are shown in the main figure.


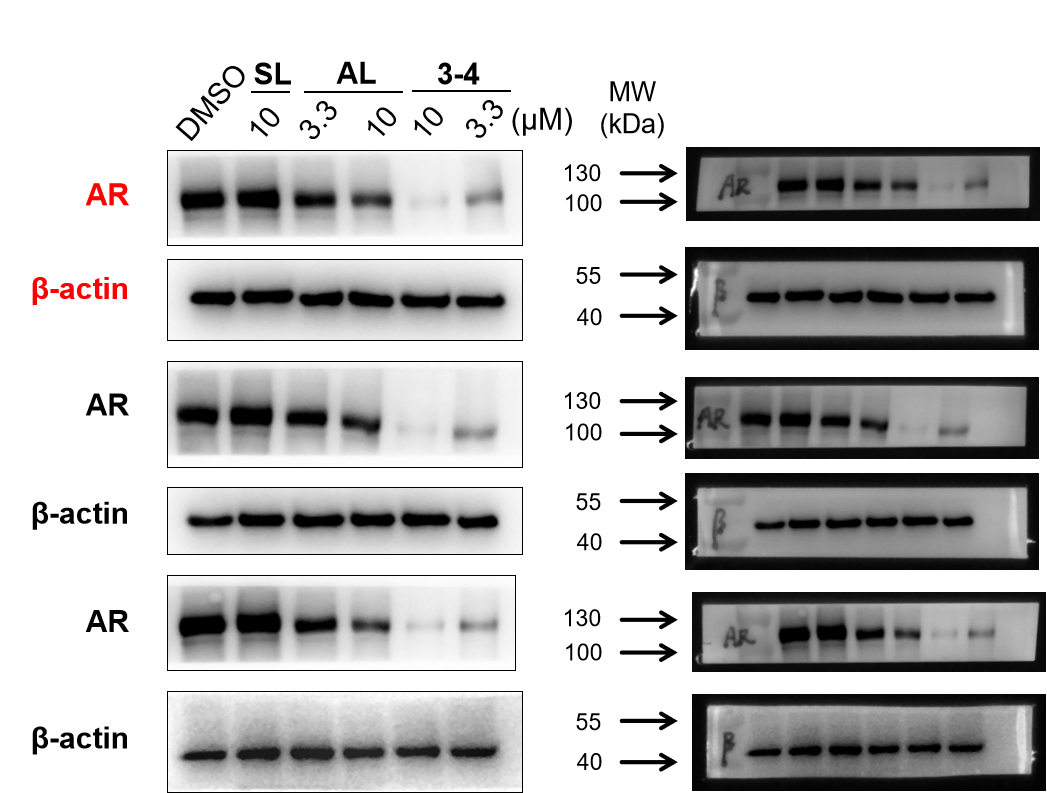


**Supplementary Figure 48.** Three replicates and original bands of Supplementary Figure 11A. The bands highlighted in red are shown in the main figure.

1. **NMR spectra**

**Supplementary Figure 49.** ^1^H NMR (500 MHz, CDCl_3_) of compound **S1.**

**Supplementary Figure 50.** ^13^C NMR (126 MHz, CDCl_3_) of compound **S1.**

**Supplementary Figure 51.** ^1^H NMR (500 MHz, CDCl_3_) of compound **S2.**

**Supplementary Figure 52.** ^13^C NMR (126 MHz, CDCl_3_) of compound **S2.**

**Supplementary Figure 53.** ^1^H NMR (500 MHz, CDCl_3_) of compound **S3.**

**Supplementary Figure 54.** ^13^C NMR (126 MHz, CDCl_3_) of compound **S3.**

**Supplementary Figure 55.** ^1^H NMR (500 MHz, DMSO-*d*_6_) of compound **SL1.**

**Supplementary Figure 56.** ^13^C NMR (126 MHz, DMSO-*d*_6_) of compound **SL1.**

**Supplementary Figure 57.** ^1^H NMR (500 MHz, CDCl_3_) of compound **SL2.**

**Supplementary Figure 58.** ^13^C NMR (126 MHz, CDCl_3_) of compound **SL2.**

**Supplementary Figure 59.** ^1^H NMR (500 MHz, CDCl_3_) of compound **22d.**

**Supplementary Figure 60.** ^13^C NMR (126 MHz, CDCl_3_) of compound **22d.**

**Supplementary Figure 61.** ^1^H NMR (500 MHz, CDCl_3_) of compound **S4a.**

**Supplementary Figure 62.** ^1^H NMR (500 MHz, CDCl_3_) of compound **S4b.**

**Supplementary Figure 63.** ^1^H NMR (500 MHz, CDCl_3_) of compound **S4c.**

**Supplementary Figure 64.** ^1^H NMR (500 MHz, CDCl_3_) of compound **S4d.**

**Supplementary Figure 65.** ^1^H NMR (500 MHz, CDCl_3_) of compound **S4e.**

**Supplementary Figure 66.** ^1^H NMR (500 MHz, CDCl_3_) of compound **S4f.**

**Supplementary Figure 67.** ^1^H NMR (500 MHz, CDCl_3_) of compound **S4g.**

**Supplementary Figure 68.** ^1^H NMR (500 MHz, CDCl_3_) of compound **S4h.**

**Supplementary Figure 69.** ^1^H NMR (500 MHz, CDCl_3_) of compound **S4i.**

**Supplementary Figure 70.** ^1^H NMR (500 MHz, CDCl_3_) of compound **S4j.**

**Supplementary Figure 71.** ^1^H NMR (500 MHz, CDCl_3_) of compound **S6a.**

**Supplementary Figure 72.** ^1^H NMR (500 MHz, CDCl_3_) of compound **S6b.**

**Supplementary Figure 73.** ^1^H NMR (500 MHz, CDCl_3_) of compound **S6c.**

**Supplementary Figure 74.** ^1^H NMR (500 MHz, CDCl_3_) of compound **2-1.**

**Supplementary Figure 75.** ^13^C NMR (126 MHz, CDCl_3_) of compound **2-1.**

**Supplementary Figure 76.** ^1^H NMR (500 MHz, CDCl_3_) of compound **2-2.**

**Supplementary Figure 77.** ^13^C NMR (126 MHz, CDCl_3_) of compound **2-2.**

**Supplementary Figure 78.** ^1^H NMR (500 MHz, CDCl_3_) of compound **2-3.**

**Supplementary Figure 79.** ^13^C NMR (126 MHz, CDCl_3_) of compound **2-3.**

**Supplementary Figure 80.** ^1^H NMR (500 MHz, CDCl_3_) of compound **2-4.**

**Supplementary Figure 81.** ^13^C NMR (126 MHz, CDCl_3_) of compound **2-4.**

**Supplementary Figure 82.** ^1^H NMR (500 MHz, CDCl_3_) of compound **2-5.**

**Supplementary Figure 83.** ^13^C NMR (126 MHz, CDCl_3_) of compound **2-5.**

**Supplementary Figure 84.** ^1^H NMR (500 MHz, CDCl_3_) of compound **2-6.**

**Supplementary Figure 85.** ^13^C NMR (126 MHz, CDCl_3_) of compound **2-6.**

**Supplementary Figure 86.** ^1^H NMR (500 MHz, CDCl_3_) of compound **2-7.**

**Supplementary Figure 87.** ^13^C NMR (126 MHz, CDCl_3_) of compound **2-7.**

**Supplementary Figure 88.** ^1^H NMR (500 MHz, CDCl_3_) of compound **2-8.**

**Supplementary Figure 89.** ^13^C NMR (126 MHz, CDCl_3_) of compound **2-8.**

**Supplementary Figure 90.** ^1^H NMR (500 MHz, CDCl_3_) of compound **2-9.**

**Supplementary Figure 91.** ^13^C NMR (126 MHz, CDCl_3_) of compound **2-9.**

**Supplementary Figure 92.** ^1^H NMR (500 MHz, CDCl_3_) of compound **2-10.**

**Supplementary Figure 93.** ^13^C NMR (126 MHz, CDCl_3_) of compound **2-10.**

**Supplementary Figure 94.** ^1^H NMR (500 MHz, CDCl_3_) of compound **2-11.**

**Supplementary Figure 95.** ^13^C NMR (126 MHz, CDCl_3_) of compound **2-11.**

**Supplementary Figure 96.** ^1^H NMR (500 MHz, CDCl_3_) of compound **2-12.**

**Supplementary Figure 97.** ^13^C NMR (126 MHz, CDCl_3_) of compound **2-12.**

**Supplementary Figure 98.** ^1^H NMR (500 MHz, CDCl_3_) of compound **2-13.**

**Supplementary Figure 99.** ^13^C NMR (126 MHz, CDCl_3_) of compound **2-13.**

**Supplementary Figure 100.** ^1^H NMR (500 MHz, DMSO-*d*6) of compound **AL.**

**Supplementary Figure 101.** ^13^C NMR (126 MHz, DMSO-*d*6) of compound **AL.**

**Supplementary Figure 102.** ^1^H NMR (500 MHz, CDCl_3_) of compound **S11a.**

**Supplementary Figure 103.** ^1^H NMR (500 MHz, CDCl_3_) of compound **S11b.**

**Supplementary Figure 104.** ^1^H NMR (500 MHz, CDCl_3_) of compound **S11c.**

**Supplementary Figure 105.** ^1^H NMR (500 MHz, CDCl_3_) of compound **S11d.**

**Supplementary Figure 106.** ^1^H NMR (500 MHz, CDCl_3_) of compound **S11e.**

**Supplementary Figure 107.** ^1^H NMR (500 MHz, CDCl_3_) of compound **S11f.**

**Supplementary Figure 108.** ^1^H NMR (500 MHz, CDCl_3_) of compound **S11g.**

**Supplementary Figure 109.** ^1^H NMR (500 MHz, CDCl_3_) of compound **S11h.**

**Supplementary Figure 110.** ^1^H NMR (500 MHz, CDCl_3_) of compound **S11i.**

**Supplementary Figure 111.** ^1^H NMR (500 MHz, CDCl_3_) of compound **S11j.**

**Supplementary Figure 112.** ^1^H NMR (500 MHz, CDCl_3_) of compound **3-1.**

**Supplementary Figure 113.** ^13^C NMR (126 MHz, CDCl_3_) of compound **3-1.**

**Supplementary Figure 114.** ^1^H NMR (500 MHz, CDCl_3_) of compound **3-2.**

**Supplementary Figure 115.** ^13^C NMR (126 MHz, CDCl_3_) of compound **3-2.**

**Supplementary Figure 116.** ^1^H NMR (500 MHz, CDCl_3_) of compound **3-3.**

**Supplementary Figure 117.** ^13^C NMR (126 MHz, CDCl_3_) of compound **3-3.**

**Supplementary Figure 118.** ^1^H NMR (500 MHz, CDCl_3_) of compound **3-4.**

**Supplementary Figure 119.** ^13^C NMR (126 MHz, CDCl_3_) of compound **3-4.**

**Supplementary Figure 120.** ^1^H NMR (500 MHz, CDCl_3_) of compound **3-5.**

**Supplementary Figure 121.** ^13^C NMR (126 MHz, CDCl_3_) of compound **3-5.**

**Supplementary Figure 122.** ^1^H NMR (500 MHz, CDCl_3_) of compound **3-6.**

**Supplementary Figure 123.** ^13^C NMR (126 MHz, CDCl_3_) of compound **3-6.**

**Supplementary Figure 124.** ^1^H NMR (500 MHz, CDCl_3_) of compound **3-7.**

**Supplementary Figure 125.** ^13^C NMR (126 MHz, CDCl_3_) of compound **3-7.**

**Supplementary Figure 126.** ^1^H NMR (500 MHz, CDCl_3_) of compound **3-8.**

**Supplementary Figure 127.** ^13^C NMR (126 MHz, CDCl_3_) of compound **3-8.**

**Supplementary Figure 128.** ^1^H NMR (500 MHz, CDCl_3_) of compound **3-9.**

**Supplementary Figure 129.** ^13^C NMR (126 MHz, CDCl_3_) of compound **3-9.**

**Supplementary Figure 130.** ^1^H NMR (500 MHz, CDCl_3_) of compound **3-10.**

**Supplementary Figure 131.** ^13^C NMR (126 MHz, CDCl_3_) of compound **3-10.**

1. **References**

[1] X. Han, C. Wang, C. Qin, W. Xiang, E. Fernandez-Salas, C.-Y. Yang, M. Wang, L. Zhao, T. Xu, K. Chinnaswamy, J. Delproposto, J. Stuckey, S. Wang, “Discovery of ARD-69 as a Highly Potent Proteolysis Targeting Chimera (PROTAC) Degrader of Androgen Receptor (AR) for the Treatment of Prostate Cancer” *J. Med. Chem.* **2019**, *62*, 941–964.

[2] B. A. Schulman, A. C. Carrano, P. D. Jeffrey, Z. Bowen, E. R. E. Kinnucan, M. S. Finnin, S. J. Elledge, J. W. Harper, M. Pagano, N. P. Pavletich, “Insights into SCF ubiquitin ligases from the structure of the Skp1–Skp2 complex” *Nature* **2000**, *408*, 381–386.

[3] D. S. Goodsell, C. Zardecki, L. Di Costanzo, J. M. Duarte, B. P. Hudson, I. Persikova, J. Segura, C. Shao, M. Voigt, J. D. Westbrook, J. Y. Young, S. K. Burley, “RCSB Protein Data Bank: Enabling biomedical research and drug discovery” *Protein Sci.* **2020**, *29*, 52–65.

[4] R. J. Rowland, R. Heath, D. Maskell, R. F. Thompson, N. A. Ranson, J. N. Blaza, J. A. Endicott, M. E. M. Noble, M. Salamina, “Cryo-EM structure of SKP1-SKP2-CKS1 in complex with CDK2-cyclin A-p27KIP1” *Sci. Rep.* **2023**, *13*, 10718.

[5] Y. Fan, S. Castleberry, “High-throughput kinetic turbidity analysis for determination of amorphous solubility and excipient screening for amorphous solid dispersions” *Int. J. Pharm.* **2023**, *631*, 122495.
